# Catellani-Inspired BN-Aromatic Expansion: A Versatile Tool toward π‑Extended 1,2-Azaborines with Tunable Photosensitizing Properties

**DOI:** 10.1021/jacs.5c19389

**Published:** 2026-01-12

**Authors:** Federica Rulli, Sergi Ordeix, Roger Bresolí-Obach, Santi Nonell, Josep Saurí, Cristina Ribas-Font, Alexandr Shafir, Raimon Puig de la Bellacasa, Ana B. Cuenca

**Affiliations:** † Institut Químic de Sarrià, 82824 Universitat Ramon Llull, Vía Augusta 390, 08017 Barcelona, Spain; ‡ Institut de Química Avançada de Catalunya, IQAC−CSIC, c/Jordi Girona 20, 08034 Barcelona, Spain; § Centro de Innovación en Química Avanzada (ORFEO−CINQA), 08017 Barcelona, Spain; ∥ Catalan Institution for Research & Advanced Studies, ICREA, Pg. Lluís Companys 23, 08010 Barcelona, Spain

## Abstract

BN-isosterism, the replacement of carbon–carbon units with boron–nitrogen pairs in organic frameworks, offers a powerful means to create novel compounds, yet methods to access larger BN-containing polyaromatic cores remain scarce. Leveraging our recently developed multigram-scale synthesis of BN-naphthalene, we now combine it with a Catellani-type arene extension (Pd­(OAc)_2_/P­(2-furyl)_3_, norbornene) to rapidly access diverse extended BN-embedded polyaromatic cores. This strategy delivers BN-embedded benzo­[*c*]­phenanthridines and curved 8- and 7-membered ring-fused derivatives, as well as BN-embedded benzofluorenones in both normal and inverse BN-vector orientations. Importantly, the ability to access both directional BN isomers, in addition to the parent C=C core, provides a rare opportunity to directly interrogate the effect of the presence and sense of the BN moiety. Most notably, light-induced singlet oxygen (^1^O_2_) generation promoted by the benzofluorenone core shows a more than 10-fold enhancement in the “boron-up” BN isostere, while dropping to negligible levels upon inversion of the BN unit. This work thus offers a blueprint for experimental electronic tuning of optically responsive organic materials through BN-mapping.

## Graphical abstract

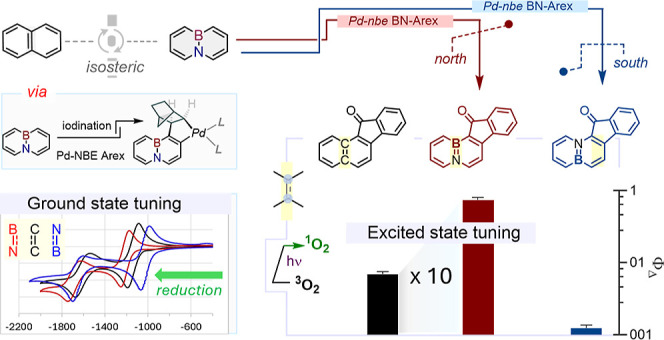

## Introduction

The replacement of one or more carbon–carbon fragments with isoelectronic boron–nitrogen pairs has emerged as a powerful strategy to modulate the electronic and photophysical properties of polycyclic aromatic hydrocarbons (PAHs).^1^ While largely preserving the geometry of the parent core, BN-isosteric substitution introduces a highly polarized B–N unit (Figure 1A) that markedly alters the molecular orbitals. As a result, considerable efforts are directed toward incorporating 1,2-azaborine motifs into biomedical,^2^ materials,^1d,1g,1i,1j,3^ and catalysis applications.^4^ Progress, however, is hindered by the limited availability of systematic research on accessing BN-isosteres of even the smallest extended aromatic cores, particularly for targets in which the BN unit is embedded rather than located peripherally. Not surprisingly, this methodological scarcity is currently driving intensive efforts toward both the from-scratch construction and late-stage modification of azaborines.^5,1k^

**Figure 1:**
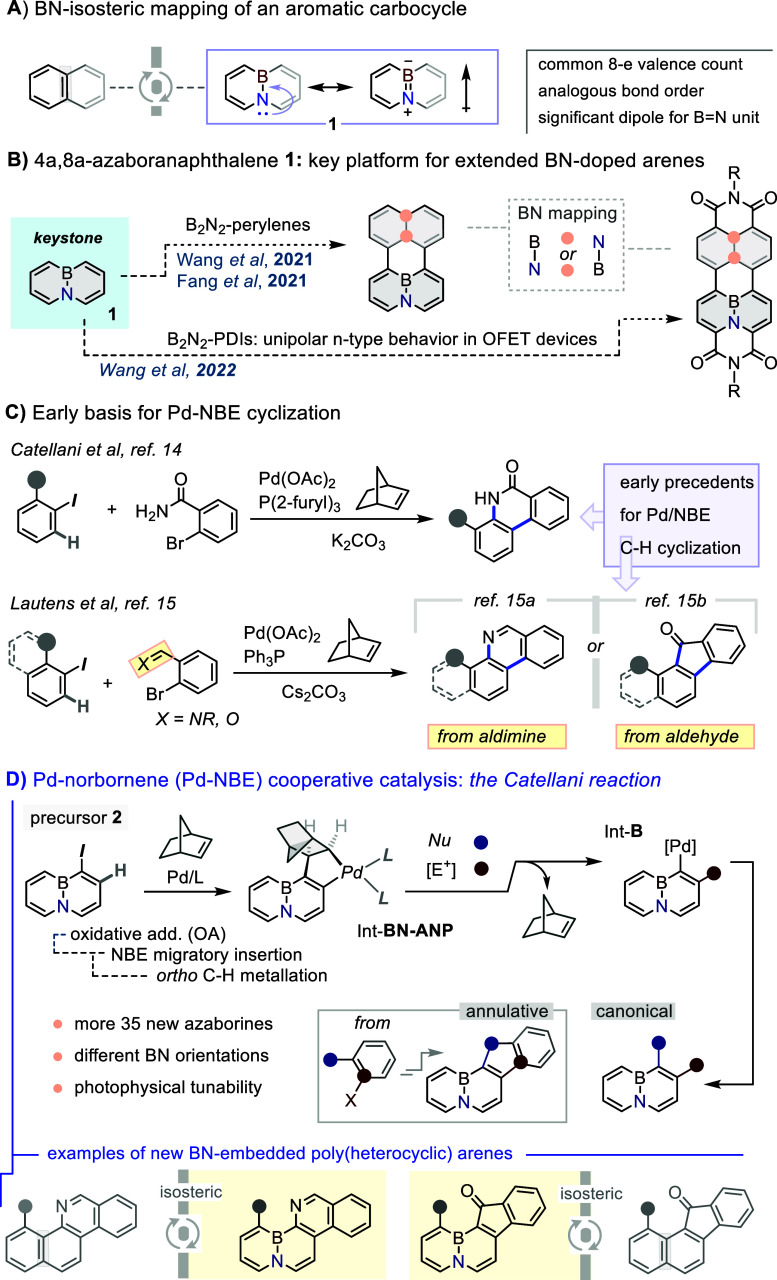
(A) BN-isosterism concept. (B) BN-naphthalene **1** as gateway to larger aromatic BN systems. (C) Early precedent for Pd-NBE cyclizative difunctionalization. (D) General scheme and main steps of the Catellani-type Pd-NBE cycle, and examples of annulative ring extension of BN-naphthalene presented herein.

As part of our own efforts in this area, we recently described a unified strategy for accessing BN-embedded polycyclic cores enabled by the formation of aminoboranes through quantitative aminolysis of B­(allyl)_3_ with *N*,*N*-diallyl and dipropargylamines.^6^ While the strategy could potentially enable BN-mapping of larger PAH, such as anthracene and tetracene, it was also used in a robust multigram synthesis of the smaller 4*a*,8*a*-azaboranaphthalene **1**, a BN-embedded isostere of naphthalene. This ready access to **1** might be expected to enable the synthesis of other BN-embedded PAH through downstream elaboration. Indeed, while various simpler functionalizations of **1** are known,^7^ its use as a building block to form tri- and tetra-cyclic cores has remained scarce.^8^ Nevertheless, the potential of **1** for accessing electronically versatile polyaromatic systems was recently illustrated in the synthesis of BN-doped perylenes,^9^ perylenes-diimides^10^ and terylene-diimides (Figure 1B).^11^ The electronic tuning resulting from the isosteric replacement in these prototypical discrete organic semiconductors enabled, for example, the observation of a high unipolar n-type behavior through their inclusion in single-crystal OFETs.^10^

Seeking to further build on the naphthalene-like **1**, we envisaged that metal-catalyzed annulative methodologies, such as a Catellani-type Pd/norbornene catalyzed annulative functionalization of **1**, could potentially provide access to a large collection of new BN-doped PAHs, if the catalytic cycle proves compatible with the presence of a BN unit. This approach would employ an iodinated derivative of **1**, such as 1-iodo-4*a*,8*a*-azaboranaphthalene **2**, and would engage the C–H position *ortho* to iodine via norbornene (NBE)-promoted shuttling of the Pd center between adjacent carbons.^12,13^ Specifically, we were inspired by annulative variants of the Catellani reaction using tethered nucleophile–electrophile partners, an avenue elegantly pioneered by the Catellani^14^ and Lautens laboratories^15^ and subsequently extended by others.^16^ Hence, Ferraccioli, Catellani and co-workers described the synthesis of phenanthridinones using 2-bromobenzamide as a dual coupling partner in combination with the Pd­(OAc)_2_/tri­(2-furyl)­phosphine/NBE catalyst system (Figure 1C-top).^14a^ Furthermore, Lautens and co-workers demonstrated that employing 2-halo-aldimines and 2-halo-aldehydes as coupling partners with a related Pd/L/NBE system enables a modular synthesis of phenanthridine^15a^ and fluorenone cores (Figure 1C-bottom).^15a^

Considering the far-reaching potential of this approach for BN-mapping key polyaromatic cores, the latter two manifolds were selected as starting point in the annulative ring expansion of BN-naphthalene **1**. Hence, following an initial validation of the approach, this study integrates the multigram synthesis of **1** with a Pd/NBE-catalyzed arene expansion (ArEx) to deliver a range of 5, 6, 7 or 8-fused BN-π-extended polyarenes, notably benzo­[*c*]­phenanthridines and benzofluorenones. Among other advances, the method is shown to enable isosteric mapping in both orientations of the BN vector, thus allowing for systematic evaluation of the influence of this directional parameter on the electronic properties of the target cores. As an example, BN-mapping of the benzofluorenone core reveals a drastically enhanced singlet oxygen generation by the BN-embedded “boron-up”, but not by the “boron-down” isostere.

The mechanistic framework for our proposal is outlined in Figure 1D. Based on literature precedents, we envisage a sequence that begins with oxidative addition of the halo-derivative (e.g., **2**) to a Pd(0) center, followed by olefin (NBE) insertion and *ortho*-C–H palladation via concerted metalation–deprotonation (CMD), yielding the key BN-naphthyl–norbornyl palladacycle Int-**BN-ANP**.^17^ At this stage, incorporation of the electrophilic component at the *ortho* (C2) position occurs through oxidative addition of the E–Y reagent to give a Pd­(IV) intermediate, followed by reductive elimination and NBE extrusion, which returns the metal center to the original C1 (*ipso*-Pd) position (intermediate **B)**. The cycle is closed by reaction with Nu–X to regenerate Pd(0), ultimately leading to the vicinal incorporation of an electrophile and a nucleophile (Figure 1D).

## Results and Discussion

### First Results and Hypothesis Validation

Given the requirement for a halogenated BN-arene as entry point, we leveraged our earlier synthesis of the BN-naphthalene **1** ^6^ to secure gram quantities of the iodo-azaborinine **2**. The nonaromatic BN-bicycle **1-H**_**4**_ was procured through a one-pot process involving a solventless proto-deallylative condensation of triallylborane with *N*,*N*-diallylamine, followed by double ring-closing olefin metathesis (Table 1A). The subsequent aromatization to give **1** was achieved with Pd/C and norbornene as sacrificial H_2_ acceptor uniquely capable of preventing detrimental saturation of one of the two rings due to inefficient H_2_ removal.^6^ While the iodination of **1** was previously achieved using the *N*-iodosuccinimide/AlCl_3_ combination,^7a^ in our hands this led to non-negligible amounts of the chlorinated side product, complicating product purification. Instead, the use of I_2_ activated with AgOTs provided **2** in a clean 85% yield (Table 1A).

**Table 1:**
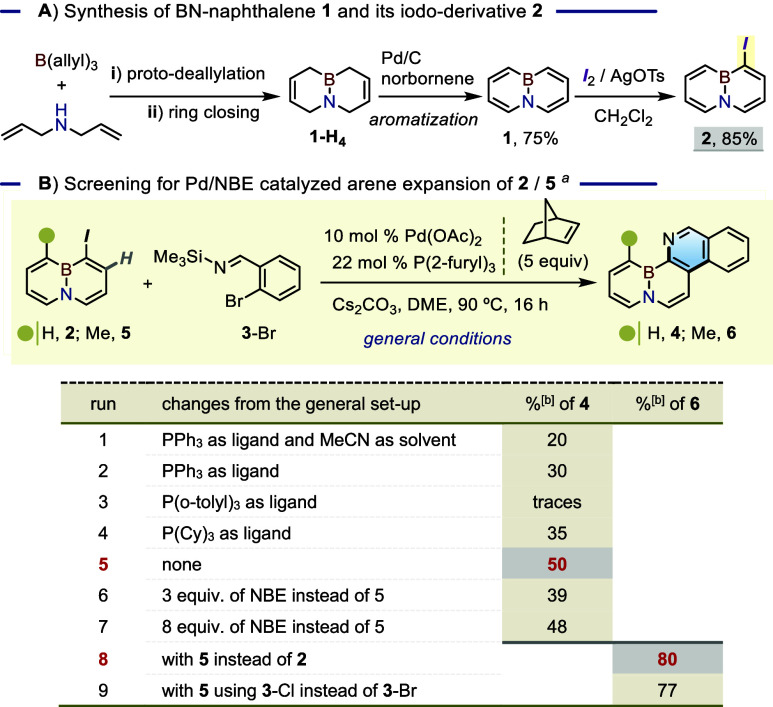
Synthesis of 2 and Initial Shaping of the General Conditions for the (4 + 2)-BN-Arex Method^c^

a Reaction conditions: **2** or **5** (0.2 mmol), **3** (0.3 mmol), Pd­(OAc)_2_ (0.01 mmol), ligand (0.022 mmol), NBE (1 mmol), and Cs_2_CO_3_ (0.6 mmol) in DME (1 mL), 90 °C, 16 h.

b Isolated yield.

c Part A. (i) Heating a neat 1:1 mixture of *N*,*N*-diallylamine and B­(allyl)_3_ to 65 °C; (ii) applying 3 mol % Grubbs I catalyst for 16 h at room temp. Part B.

Inspired by the arene expansion precedent from the Lautens laboratory,^15a^ the formation of the BN-phenanthridine scaffold was attempted by exposing a mixture of **2** and 2-bromophenyl-*N*-silyl-aldimine, **3**-Br, in acetonitrile to the combination of Pd­(OAc)_2_/PPh_3_ with norbornene (NBE, 5 equiv) in the presence of Cs_2_CO_3_ (Table 1B). After 16 h at 90 °C, the GCMS analysis revealed the formation of a mixture of new species, including a peak consistent with the target BN-embedded phenanthridine **4** (*m*/*z* 230). Upon its isolation in 20% yield (Table 1B, entry 1), the structure of **4** was confirmed by ^1^H, ^13^C, ^11^B NMR spectroscopy (see Supporting Information); the yield was increased to 30% by switching to 1,2-dimethoxyethane (DME) as solvent (entry 2). While no significant improvement was gained by switching the ligand to tri­(*o*-tolyl)­phosphine or tricyclohexylphosphine (entries 3 and 4), the use of tri­(2-furyl)­phosphine resulted in a synthetically meaningful 50% yield (entry 5). The norbornene loading of 5 equiv proved optimal, with lower efficiencies observed when going below or above this amount (entries 6 and 7). This GCMS analysis also showed the reaction balance made up of BN-naphthalene side products containing a norbornyl fragment,^18^ indicating hurdles in reaching or completing the norbornene extrusion stage. Considering that the well-documented *ortho* effect^19^ might help alleviate some of these hurdles through additional steric bulk at the substrate’s complementary *ortho* position, we synthesized the iodo-BN naphthalene **5** bearing a *peri*-Me group (see Supporting Information for details). To our delight, the use of **5** under the previously optimized conditions led to the corresponding BN-phenanthridine **6** in 80% yield (Table 1B, entry 8); interestingly, the use of the chlorinated silyl imine **3**-Cl proved similarly effective (77%, entry 9).

### New BN-Phenanthridine Skeletons

The new (4 + 2)-BN-Arex variant was subsequently extended to other 2-bromoaryl-*N*-silyl-aldimines (Table 2). In this way, the extension of **2** with the CF_3_-substituted aldimine **7** afforded the BN-phenanthridine **8** in 53% yield; a more efficient reaction with the methylated precursor **5** afforded the *peri*-Me derivative **9** in 79%. The coupling between **5** and the heliotropin-derived aldimine afforded the pentacyclic BN-target **10** in a 47% yield. As an important synthetic feature, the process also provided rapid access to new family of poly heteroatom doped polycyclic cores. Hence, the use of the thiophene-based *N*-silyl aldimine **11** led to the smooth formation of the tetracyclic BN-benzo-thieno-[2,3-*c*]-quinolines **12** and **13** in 72% and 88% yields, respectively. Furthermore, the use of the nicotine-derived aldimine **14** led to the poly heterocyclic BN-naphthyridine cores **15** and **16** in 47% and 82% yield, respectively, albeit using an increased catalyst loading. Incidentally, while primarily targeting the poly heteroarenes **12–13** and **15–16** for their potential optoelectronic behavior, we note that both the thienoquinoline and naphthyridine units are also structural components of known pharmacophores, including those involved in kinase inhibition,^20^ as well as anticancer and antiviral scaffolds.^21^

**Table 2:**
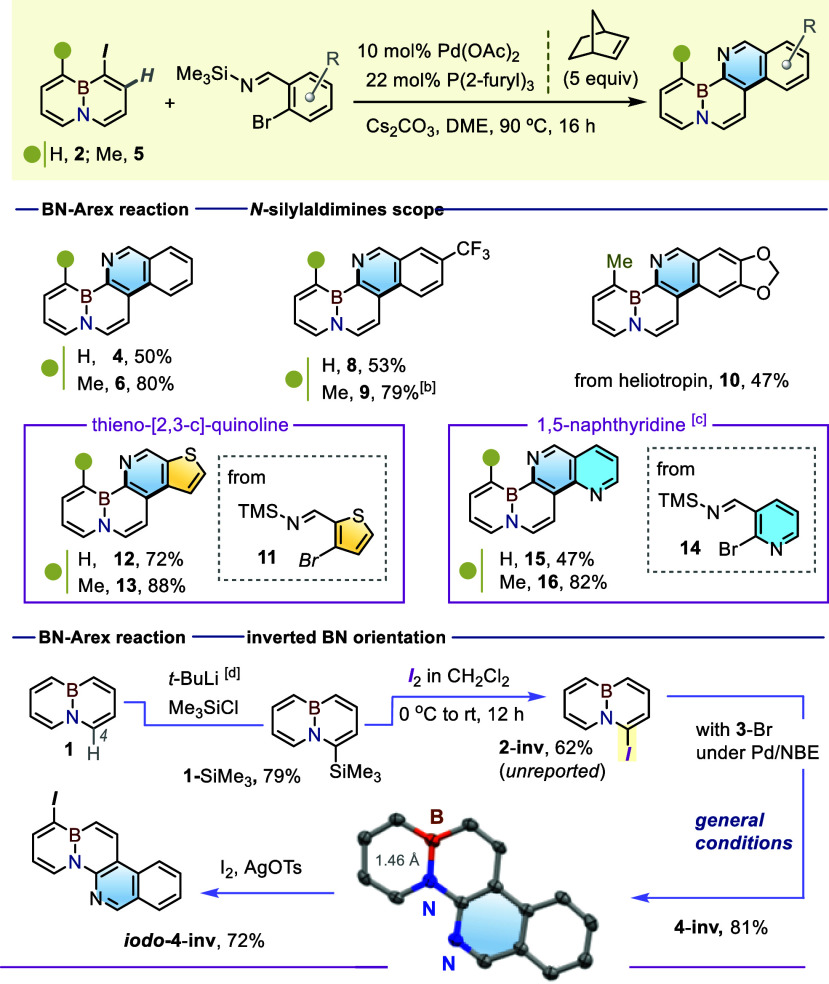
New BN-Embedded Phenanthridines Accessed by the (4 + 2)-BN-Arex Reaction^a^

a Reaction conditions: iodo-BN substrate (0.2 mmol), *N*-silylated imine (0.3 mmol), Pd­(OAc)_2_ (0.010 mmol), tri­(2-furyl)­phosphine (0.022 mmol), NBE (1 mmol), and Cs_2_CO_3_ (0.6 mmol) in DME (4 mL), 90 °C, 16 h.

b 0.02 mmol of Pd­(OAc)_2_ were used.

c 0.03 mmol of Pd­(OAc)_2_ were utilized.

d *t*-BuLi, THF, −78 °C, 30 min, and then, Me_3_SiCl −78 °C to rt, 16 h.

Given the importance of the v⃗(B–N) vector sense in the context of BN-isosterism,^22^ we also sought an analogue of **4** with an inverted BN unit. The functionalization of the “south side” of the BN-naphthalene **1**, i.e. positions α to N, is less developed due to the inability of this site to engage in EAS reaction. Still, we hoped to exploit the selective deprotonation of **1** with strong bases such as *t*-BuLi^7c,9a^ to prepare the new iodinated precursor **2-inv**. Since direct lithiation/iodination proved challenging, the target was obtained instead via the trimethylsilyl intermediate **1**-SiMe_3_, readily accessible by quenching **1**-(4-Li) with Me_3_SiCl (79%), followed by Si-to-I *ipso* substitution with I_2_ in CH_2_Cl_2_ (Table 2, bottom panel).

The resulting **2**-**inv** underwent smooth BN-Arex transformation to the target **4**-**inv** in 81% yield^23^ under the standard conditions (Table 2, bottom panel). The crystal structure of **4**-**inv** confirms the inverted B–N vector with a B–N distance of ≈1.46 Å. The **4**-**inv** remains susceptible to EAS reactivity through its α-boron C-H positions, as confirmed by the synthesis of the iodinated derivative *iodo-* **4-inv**, a compound potentially suitable for further synthetic elaborations.

Following the successful BN-Arex process using aldimines, we proceeded to test the *N*-silyl ketimines. Gratifyingly, a reaction between the *peri*-Me substrate **5** and ketimines **17-Me** and **17-Bu** led to the corresponding C7-alkyl BN-phenanthridines **18** and **19** in 87% and 71% yield, respectively (Scheme 1A); a somewhat lower yield was obtained for the C7-Ph derivative **20**. Curiously, the structural outcome showed a marked dependence on the substituent at the *peri* position of the iodinated precursor. Indeed, switching from **5** to the nonmethylated precursor **2** in a reaction with **17-Me** produced a roughly equimolar mixture of the target BN-phenanthridine **21** and a new species with a molecular mass (*m*/*z* = 338), a value consistent with a product bearing an additional norbornene unit (Scheme 1B). Analogous outcome was also observed for reactions between **2** and the other two ketimines (see Supporting Information, Section 6.1, page S21). Despite having an *R*_f_ value virtually identical with that of **21**, the new species could be isolated in a 32% yield, allowing for its structural determination by NMR.

**Scheme 1:**
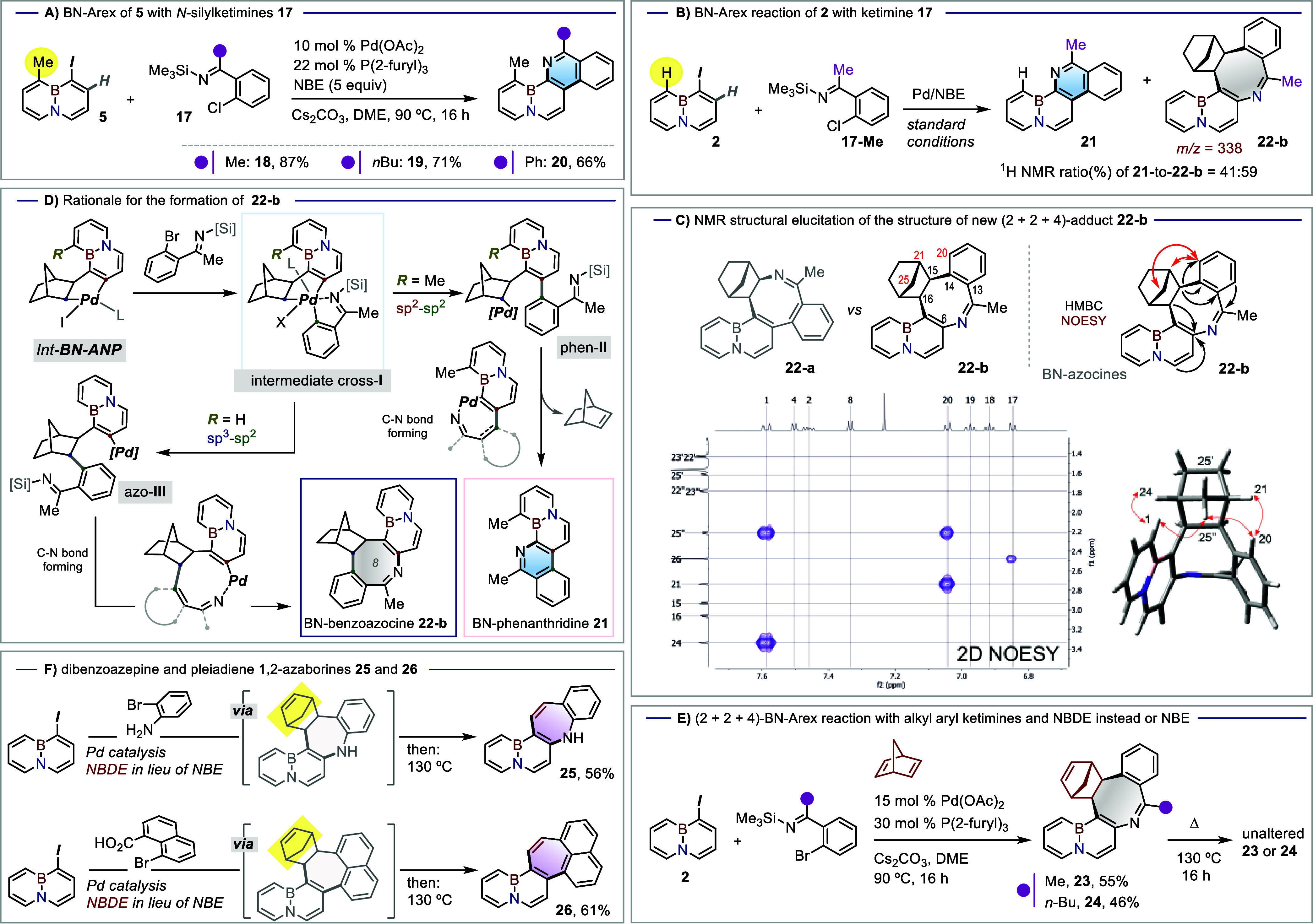
(A) (4 + 2)-BN-Arex Reaction with *N*-Silyl Ketimines; (B) Expansion Reaction between Ketimine **17**-**Me** and Substrate **2**; (C) Spectroscopic Investigation to Elucidate the Structure of the 8-Membered Ring Adduct **22**-**b**; Key HMBC and NOESY Correlations Are Highlighted in the Planar Structure of **22-b**, whilst NOESY Correlations Are Also Highlighted in the 3D Structure; (D) Mechanistic Rationale for the Formation of **22**-**b**; (E) NBDE-mediated (2 + 2 + 4)-BN-Arex Reaction with Alkyl Aryl *N*-Silyl Ketimines **17-Me** and **17-Bu**; Formation of New 8-Membered BN Pentacyclic Structures **23** and **24**; (F) Access to BN-Dibenzoazepine **25** and BN-Pleiadiene **26**

Preliminary analysis led to two possible isomeric 8-membered azocine structures **22**-**a** and **22**-**b** differing in the orientation of their ketamine-derived C–C–C–N fragment. Definitive structural assignment as **22-b** was supported by key NOESY and HMBC correlations (Scheme 1C). HMBC correlations involving the norbornene and the benzoazocine moieties (black arrows), were pivotal, as their relative positioning distinguishes **22-b** from **22-a**. Long-range HMBC correlations from the norbornane proton H-15 to C-14, C-13, and C-20 were consistent with direct connectivity between the norbornane and benzoazocine units in **22-b**. Additionally, a correlation from H-16 to a quaternary carbon resonating at 158.8 ppm, assigned as C-6, was observed. This carbon also showed HMBC correlations from protons from the BN-naphthalene, further supporting structure **22-b** exclusively. Furthermore, the NOESY correlations (red arrows) between the aromatic benzoazocine proton H-20 and the norbornane bridgehead methylene protons H-25 and methine H-21 were also fully consistent with **22-b** and effectively rule out **22-a**. The NOESY data also confirmed the expected *cis* stereochemistry at the newly formed norbornane junction, in line with 3D model depicted in Scheme 1C. A unique predominant conformer was found from the DFT geometry optimization as shown in the 3D structure in Scheme 1C.^24^ Interestingly, compound **22**-**b** is a formal reduced Diels–Alder adduct of an 8-membered azocine ring^25^ that adopts a pseudo boat-like geometry, thus inducing a clear curve shape to the molecule.

The selective conversion of compound **5** to phenanthridines **18**–**20**, as opposed to the divergent conversion of **2** to the phenanthridine/azocine mixture (**21** and **22**-**b**), can be rationalized within a unified mechanistic framework that bifurcates depending on the nature of the *peri* substituent (methyl vs hydrogen). A common Catellani-type Pd/NBE sequence, beginning with oxidative addition of the Nap_BN_–I bond to Pd(0), would thus be followed by norbornene insertion and subsequent oxidative addition of the Ar–Br precursor. This sequence leads to a shared Pd­(IV) intermediate,^26^ cross-**I**, in which an N···Pd interaction with the imine group is postulated^27^—especially considering that desilylation of the imine could already have occurred by this point. As shown in Scheme 1D, this octahedral Pd­(IV) intermediate functions as a mechanistic crossroads, from which the reaction can diverge into two distinct C–C bond-forming pathways: (a) a C­(sp^2^)–C­(sp^2^) reductive elimination that connects the C2 position of the BN-naphthalene core with the *ortho*-ketimine carbon, thus forming intermediate phen-**II**, or (b) a C­(sp^2^)–C­(sp^3^) bond formation leading to the norbornene-containing intermediate azo-**III**. Both of these intermediates are expected to undergo intramolecular Buchwald–Hartwig amination, ultimately yielding either BN-phenanthridine **21** or BN-azocine **22-b**, respectively. In this framework, the *peri*-methyl substituent in compound **5** exerts an enhanced *ortho* effect,^19^ biasing the system toward phen-**II** and, ultimately, the phenanthridine formation. The diminished steric demand in **2** relaxes this tendency, allowing for a portion of the cross-**I** population to evolve via azo-**III** to azocine **22**-**b**. We note that prior mechanistic research highlights the ability of the chelation phenomenon to override the *ortho* effect, thus enabling the appearance of norbornane-containing derivatives, for example via azo-**III**.^28,29^ In case of substrate **5**, however, the *peri*-Me group appears to create a super *ortho*-effect that brings the reactivity back to the canonical Ar–Ar coupling via phen-**II**, thus providing the expected phenanthridine targets.

Incidentally, the same reaction conditions also allow for the use of norbornadiene in lieu of norbornene. This enables the coupling between **2** and ketimines **17-Me** or **17-Bu** to afford BN-azocines **23** and **24** in moderate yields (Scheme 1E). Despite the potential for these ring systems to undergo a retro-Diels–Alder reaction,^30^ no cyclopentadiene extrusion was observed upon heating **23** and **24** to 130 °C for 16 h. Nevertheless, a related Catellani-style sequence involving norbornadiene incorporation and subsequent cyclopentadiene extrusion, with norbornadiene thus acting as a masked acetylene, was successfully implemented within a (2 + 2 + 3)-BN-Arex process to access new 7-membered derivatives of the BN-naphthalene core. The task was inspired by the scarcity of heptagon rings fused with BN-embedded cores,^31^ which stands in stark contrast with the great potential of such ring system in the design of organic electronics, including through the induction of negative curvatures.^32^ Inspired by an interesting report by Catellani, Derat and co-workers on the synthesis of dibenzoazepine core,^29a^ subjecting a mixture of **2** and 2-bromoaniline under catalytic conditions led to the corresponding NBE intermediate, as observed by an ^1^H NMR sampling; further heating to 130 °C enabled smooth evolution of this species to BN-dibenzoazepine **25** via a retro-DA reaction (Scheme 1F, top). In a related experiment inspired by the work of Kwong et al.,^33^ a decarboxylative coupling between **2** and 8-bromo-1-naphthoic acid initially led to a NBE-containing intermediate that readily evolved to the new BN-extended pleiadiene **26** (61%) upon heating at 130 °C (Scheme 1F, bottom).

### Preparation of New BN-Fluorenone Cores

Seeking to further expand the chemical space of BN-embedded poly heteroarenes via Pd/NBE-enabled *ortho*/*ipso* ring fusion, and motivated by the broad utility of fluorenone frameworks^34^ in biological,^35^ photonic, and organic electronic applications,^36^ our next goal was to access fluorenone-type cores through the reaction of **2** or **5** with 2-bromo­(hetero)­aryl aldehydes.^15b^ We were pleased to find that heating a mixture of **2** and 2-bromobenzaldehyde under our standard conditions at 90 °C for 17 h afforded the BN-benzo­[*a*]­fluorenone **27** in 43% yield (Scheme 2A),^37^ along with side products that include 1-norbornyl-BN-naphthalene (see compound **S1** in Supporting Information). Interestingly, the earlier improvement in efficiency noted during the formation of the phenanthridine core upon switching from **2** to the methylated iodo BN-naphthalene **5** was also mirrored in this case, with **5** converted to 1-methyl BN-fluorenone **28** in 71% yield. The reaction worked well with bromo-benzaldehyde coupling partners bearing the electron-withdrawing 3-trifluoromethyl group (comp. **29**, 50% yield) and the 3-aldehyde group (comp. **30**, 66% yield). The process was also extended to the piperonal-derived BN-embedded fluoren-11-one **31** obtained in a 68% yield. The solid-state structure of this compound (see ORTEP drawing in Scheme 2, center right) revealed a B–N bond length of 1.48 Å, in line with a partially delocalized B=N fragment;^38^ the packing diagram also revealed extensive π-stacking of the polyaromatic cores (Figure S13). Given the promise of heteroarene-containing π-cores in the development of organic optoelectronic materials^39^ and new bioactive cores,^40^ the Pd/NBE ArEx reaction was also applied to prepare the BN-embedded tetracyclic benzo­[*a*]­thieno-fluorenone **32**, as well as nicotinic and picolinic-derived aza-benzo-[*a*]-fluororenones **33–36** in moderate to good yields (Scheme 2, A).

**Scheme 2:**
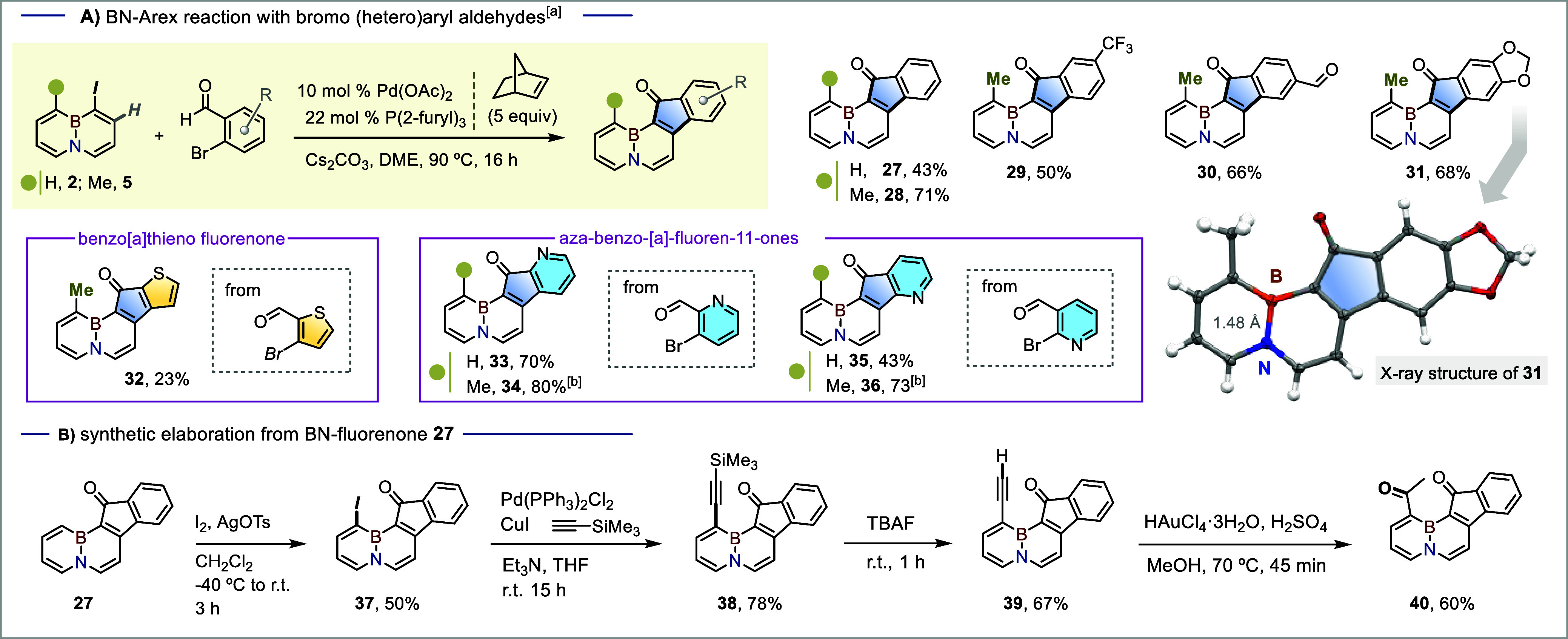
(A) (3 + 2)-BN-Arex Reaction with 2-bromo Aryl (or Heteroaryl) Aldehydes: [a] Reaction Conditions: Iodo-BN Substrate (0.2 mmol), 2-bromo Aryl (or Heteroaryl) Aldehyde (0.3 mmol), Pd­(OAc)_2_ (0.010 mmol), Tri­(2-furyl)­phosphine (0.022 mmol), NBE (1 mmol), and Cs_2_CO_3_ (0.6 mmol) in DME (4 mL), 90 °C, 17 h. [b] 0.03 mmol of Pd­(OAc)_2_ Were Utilized; (B) Illustrative Synthetic Elaboration from BN-Fluorenone **27**

The newly formed BN-embedded tetracycles derived from **2** preserve a C–H site α to the boron center, rendering them amenable to further functionalization via EAS. This feature is showcased for the BN-fluorenone **27**, readily transformed into iodinated derivative **37** in 50% yield (unoptimized) upon exposure to I_2_/AgOTs in CH_2_Cl_2_ (Scheme 2B). This further enables Pd-catalyzed cross-coupling, with a Sonogashira reaction followed by deprotection furnishing the alkynyl derivatives **38** and **39** in 78% and 67% yields, respectively. The gold­(III)-catalyzed hydration of the latter provided the diketone BN-fluorenone **40** in a 60% yield.

### How Consequential is the BN-Mapping on Photophysical Properties?

Having secured access to BN isosteres of benzo­[*c*]­phenanthridine and benzo­[*a*]­fluorenone, we were now well positioned to probe the photophysical consequences of CC-for-BN replacement, including the influence of BN vector orientation. For the benzo­[*c*]­phenanthridine framework, the absorption spectrum of the parent (non-BN) **carbo-4** (for preparation, see Supporting Information, Section 5) is dominated by two high-intensity bands peaking at 254 and 262 nm, with additional lower-intensity bands with maxima at 342 and 359 nm (Figure 2A-top, solid black). The fluorescence spectrum mirrors the lowest-energy bands with a negligible Stokes shift, suggesting that the molecule is very rigid both in the ground and in the first excited singlet state. From the crossing point of the overlapping absorption and fluorescence bands, the energy of the first excited singlet state is calculated as 334 kJ mol^–1^. Interestingly, striking spectral differences are immediately apparent for the boron-up BN isostere **4**. The highest-energy absorption bands now appear at longer wavelengths and with smaller absorption coefficients, by a remarkable factor of ∼ 2 for the red-most band, while the lowest-energy band shifts to the blue (Figure 2A, middle, solid red). At the same time, the fluorescence spectrum shifts to the red, resulting in a large Stokes shift (dashed red line). Furthermore, a striking contrast exists between the clearly structured emission observed for **carbo-4** and the structureless fluorescence spectrum of **4**, suggesting a loss of rigidity in the excited state. The calculated energy of the singlet excited state is now 346 kJ mol^–1^, a value higher than for the parent structure **carbo-4**. Finally, flipping the orientation of the BN-vector (the boron-down form **4-inv**) results in intermediate behavior. A comparison with the all-carbon parent structure **carbo-4** showed that inverse-sense BN doping has modest to negligible impact on the lowest-energy absorption bands, the Stokes shift, and the excited singlet state energy. In contrast, the highest-energy absorption region is more similar to that of the boron-up BN isomer **4**, and the fluorescence spectrum is also broadened and noticeably less structured. Consistent with the above trends, the fluorescence lifetime (τ_F_) is 5.0 ns for **carbo-4**, a value that decreases to 3.1 ns for **4**, and shows an intermediate lifetime of 4.2 ns for **4-inv** (see Table S1). The complete photophysical characterization of the remaining BN-phenanthridines **6**, **8–10**, **12**, **13**, **15**, **16** and **18–21**, as well as new BN-doped 7- and 8-membered cyclic cores **22-b** and **23–26** are shown in the Supporting Information (Figures S2–S5).

**Figure 2:**
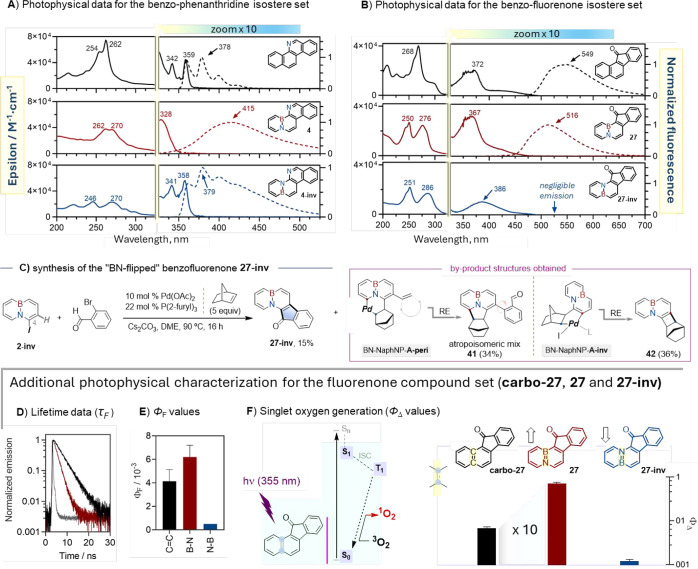
(A) Absorption (solid lines) and fluorescence emission (dashed lines) spectra of carbonaceous phenanthridine (**carbo-4**; black), boron-up BN-phenanthridine (**4**; dark red), and boron-down BN-phenanthridine (**4-inv**; blue) derivatives in acetonitrile. (B) Absorption (solid lines) and fluorescence (dashed lines) spectra of fluorenone **carbo-27** (black), BN fluorenone **27** (red), and **27-inv** fluorenone (blue) derivatives in acetonitrile. (C) BN-Arex reaction to prepared **27-inv**. Two principal BN-containing byproducts are also depicted. (D) Time-resolved fluorescence kinetic traces of **carbo-27** (black), **27** (red) and **27-inv** (blue). (E) Fluorescence quantum yield (Φ_F_) of **carbo-27**, **27** and **27-inv**. (F) ^1^O_2_ formation quantum yield (Φ_Δ_) induced by **carbo-27**, **27** and **27-inv**. As a visual aid, the Φ_Δ_ values for **carbo-27**, **27**, and **27-inv** are plotted on logarithmic scale due to significant differences in their values.

Analogously, the benzo­[*a*]­fluorenone compound set was also characterized from the optical and photophysical point of view. Hence, the boron-up BN-fluorenone **27** exhibited absorption and fluorescence spectra quite similar to those of the parent C=C compound **carbo-27**, albeit with a slight blue shift apparent in both spectra of **27** (Figure 2B, top and middle, solid lines for absorption and dashed for emission). In turn, the singlet excited state lifetime of the BN isostere is significantly shorter (τ_F_ = 1.6 ns for **27** vs 3.5 ns for **carbo-27**; see Figure 2D), while the fluorescence quantum yield Φ_F_ is comparable (0.006 vs 0.004; Figure 2E).

Undoubtedly, the ultimate goal of electronic tuning, including through BN mapping, is to obtain molecules with enhanced target function. Cognizant of the general ability of aryl ketones to undergo rapid and efficient singlet-to-triplet intersystem crossing (ISC), we decided to compare the ability of benzofluorenones **27** and **carbo-27** to induce the generation of singlet oxygen (^1^O_2_), which occurs from the triplet excited state. Beyond its chemical and biological roles, singlet oxygen production reflects efficient population of the triplet excited state. Indeed, the specific energy ordering of nπ* and ππ* excited states in aromatic ketones strongly affects the fate of the excited states.^41^

The simplest fluorenone molecule has been shown to exhibit efficient intersystem crossing (ISC) to the triplet state in nonpolar solvents, while this process is highly inefficient in polar solvents.^42^ In fact, literature data and our own measurements indicate that the ISC in **carbo-27** is limited in both nonpolar and polar solvents, with internal conversion dominating, particularly in polar solvents.^43^ Not surprisingly, we have found that the quantum yield of singlet oxygen production (Φ_Δ_), an energy-transfer process that occurs from long-lived excited triplet states, is only 0.05 (Figure 2F, black bar), confirming the triplet state formation being a minor pathway. Remarkably, the Φ_Δ_ value of BN-fluorenone **27** is ∼10-fold higher (0.77; Figure 2F, red bar), indicating that the singlet-to-triplet ISC pathway is strongly favored by the BN substitution. Assuming that the triplet quantum yield is equal to Φ_Δ_, the intersystem crossing rate constant (*k*_ISC_) is calculated as 4.7 × 10^8^ s^–1^ for **27**, 23-fold higher than for **carbo-27** (2.0 × 10^7^ s^–1^, see Table S3). Of note, the discrepancy between the enhancement of *k*_ISC_ and Φ_Δ_ indicates that internal conversion must be slower for **27**, consistent with the higher energy of its singlet excited state (Figure 2B-top and middle), on account of the energy-gap law.^44^ As far as we are aware, this is the first case in which enhanced conversion of ^3^O_2_ into ^1^O_2_ is achieved via direct BN-mapping of an all-carbon sensitizer candidate.^45^

Intrigued by the effect of inverting the orientation of the BN unit on the ^1^O_2_ generation capacity, we sought the “B-down” isomer **27-inv**, a species not prepared during the initial synthetic campaign. Although the (3 + 2)-BN-Arex coupling of the BN-inverted iodo precursor **2-inv** proved less efficient, the reaction did afford quantities of the target fluorenone **27-inv** (Figure 2C) sufficient for subsequent photophysical characterization. This lower efficiency was partially explained by the formation of an atropoisomeric mixture of BN structure **41** (34%) and the cyclobutyl-fused **42** (36%), the latter likely arising from a direct C–C reductive elimination in the BN-naphthalene-norbornyl-palladacycle BN-NaphNP-**A-inv** (Figure 2C, right). In contrast, species **41** might well arise as a consequence of a second metalation event, followed by a reductive elimination, after the successful incorporation of the benzaldehyde electrophile (late intermediate BN-NaphNP**-A-peri**).

The absorption spectrum of **27-inv** exhibits essentially the same overall profile as that of the boron-up isomer **27**, except that the band system centered at ∼370 nm disappears, leaving only the underlying broader structureless absorption, a band clearly observed in the spectrum of **27**. Strikingly, compound **27-inv** showed no detectable fluorescence and an almost negligible singlet oxygen Φ_Δ_ value of 0.01 (Figure 2E, blue bar), even lower than the carbonaceous **carbo-27**. Thus, the change in the orientation of the boron–nitrogen unit in **27-inv** has a dramatic effect on the photophysical properties of the fluorenone core, stripping it of any photochemical activity. The photophysical characterization of the rest of substituted BN-fluorenones (**28–40**) is shown in the Supporting Information (Figures S6–S9). Φ_Δ_ values range from 0.07 to 0.80, reflecting the nature and position of the substituents in a way consistent with the effects on ^1^O_2_ generation described for substituted fluorenones.^46^

In this context, interesting insights can be gained from DFT modeling of benzofluorenones **carbo-27**, **27** and **27-inv** at the CAM-b3lyp/Def2TZVP level. As we expected, all three compounds display similarly shaped frontier orbitals, with the HOMO (bottom) and LUMO (top) electron densities illustrated in Figure 3A. Despite these similarities, the BN doping alters orbital energies, with the −1.56 eV LUMO energy of **carbo-27** raised to −1.47 eV in the boron-up BN isostere **27**, and lowered to −1.69 eV in the boron-down derivative **27-inv**. Given that the LUMO orbital is responsible for accepting an electron upon reduction, this energy perturbation was found to directly translate into the compounds’ reduction potentials.^47^ Hence, the cyclic voltammetry measurements in CH_3_CN for all three species revealed two pseudoreversible reduction waves, presumably signaling the formation of the radical anion **[Benzofluorenone]** ^**•–**^ and a second doubly reduced species. For the reference **carbo-27**, the first reduction wave was found at *E*_1/2_ = −1.14 V vs the Standard Calomel Electrode (SCE). In line with the computed LUMO energy ordering (Figure 3B) this wave shifts the lower potential of *E*_1/2_ = −1.22 V for **27**, but in contrast moves up to the higher potential of *E*_1/2_ = −1.03 V for **27-inv** (Figure 3C). Illustrating once again electronic tuning afforded by BN-mapping, these measurements reveal how the redox potential of an organic core can be adjusted - up and down- in a ∼190 mV span by playing with the presence and sense of the BN vector (for full data, see Supporting Information).

**Figure 3:**
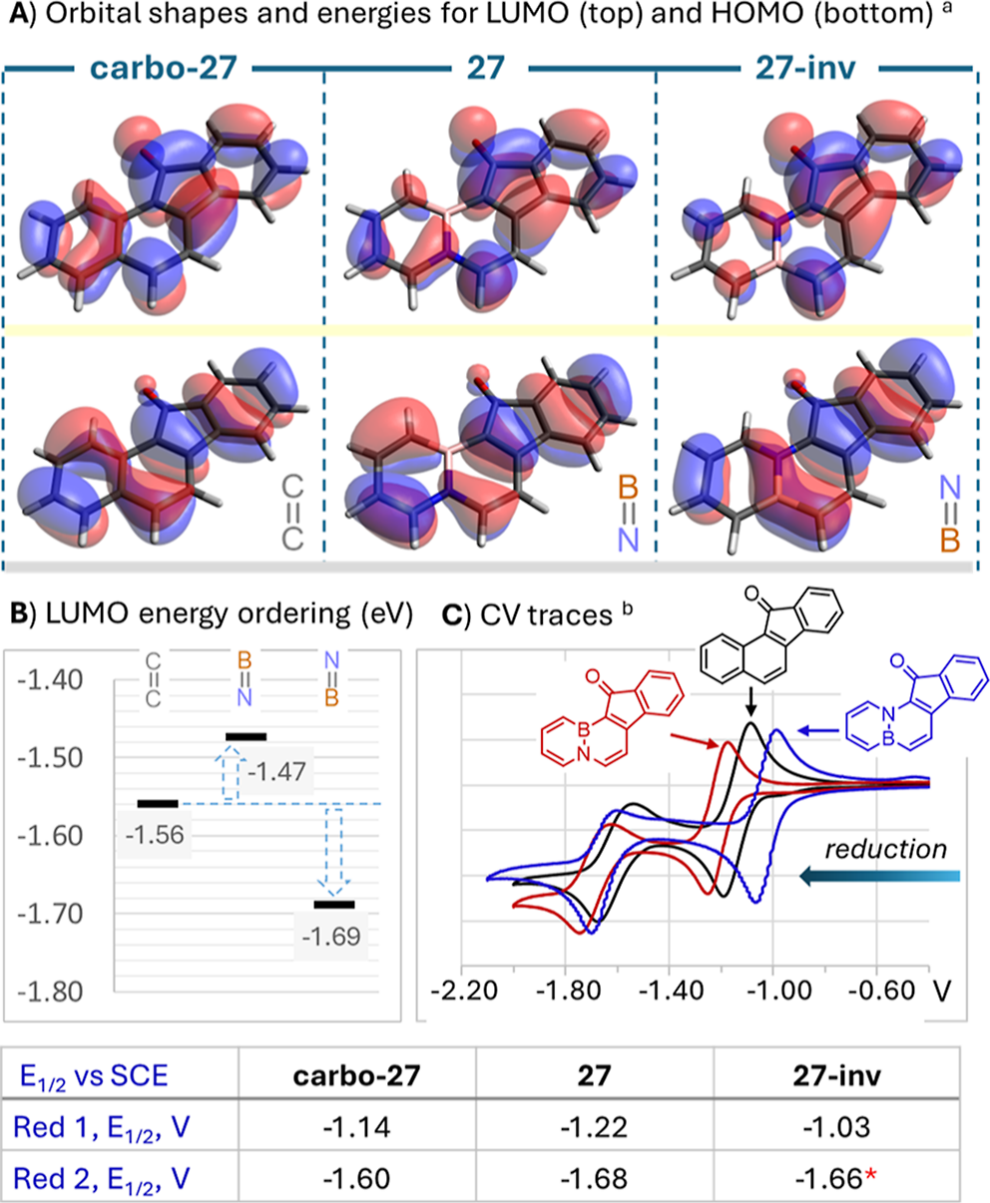
(A) Frontier orbitals shapes and energies (eV) of the benzofluorenone set obtained by DFT calculations at CAM-b3lyp/Def2TZVP level. (B) LUMO energy ordering for **carbo-27**, **27** and **27-inv**. (C) Cyclic voltammograms (reduction region) measured in CH_3_CN with TBAPF_6_ electrolyte at 100 mV/s scan rate and referenced to SCE. *Partially irreversible wave.

Ultimately, orbital perturbations both in the ground and excited states appear to culminate in **27** significantly outperforming its carbonaceous and boron-down isostere as a triplet sensitizer in ^1^O_2_ production, as was shown in Figure 2F. Despite some preliminary exploration by TD-DFT,^48^ the exact origin of this phenomenon remains under investigation.

## Conclusion

In conclusion, this study shows that earlier advances in BN-naphthalene synthesis pave the way for the construction of larger BN-doped polyarenes via Pd/norbornene-promoted core extension. A Catellani-type aryl extension (ArEx) process is thus configured to engage iodo-BN-naphthalene substrates, affording a plethora of new BN-isosteric architectures, including BN-doped benzo­[*c*]­phenanthridines, curved 7- and 8-membered ring-fused derivatives, and BN-embedded benzofluorenones. The newly accessed structural diversity underscores the profound effect of the presence and the directionality of the BN unit on the photophysical properties of the polyarene framework. Most notably, light-induced singlet oxygen (^1^O_2_) generation promoted by the benzofluorenone core shows a 10-fold enhancement in the “boron-up” BN isostere, while dropping to negligible levels upon inversion of the BN unit. These findings could contribute to the development of a new generation of singlet oxygen photosensitizers with altered properties derived from the BN-isosteric replacement.

## Supplementary Material



## Notes

^49^ Details on the synthetic procedures and product characterization, including NMR and MS data. Detailed photophysical characterization. Details on single crystal X-ray structural characterization are also included (DOCX)
