# Supplementary material for: Catellani-Inspired BN-Aromatic Expansion: A Versatile Tool toward π‑Extended 1,2-Azaborines with Tunable Photosensitizing Properties
Source: J Am Chem Soc. 2026 Jan 12;148(3):3614–25. doi: 10.1021/jacs.5c19389 (PMC12856902; doi:10.1021/jacs.5c19389)
Supplement: Supplementary file 1 [file ja5c19389_si_001.docx]

Catellani-Inspired BN-Aromatic Expansion: A Versatile Tool Towards π‑Extended 1,2-Azaborines with Tunable Photosensitizing Properties

Federica Rulli,^a^ Sergi Ordeix,^a^ Roger Bresolí-Obach,^a^ Santi Nonell,^a,d^ Josep Saurí,^a^ Cristina Ribas-Font,^a^ Alexandr Shafir,^b,c^* Raimon Puig de la Bellacasa^a^* and Ana B. Cuenca^a,c^*

^a^ Institut Químic de Sarrià, Universitat Ramon Llull, Vía Augusta 390, 08017 Barcelona, Spain

^b^ Institut de Química Avançada de Catalunya, IQAC-CSIC, c/ Jordi Girona 20, 08034, Barcelona, Spain

^c^ Centro de Innovación en Química Avanzada (ORFEO-CINQA), Spain

d Catalan Institution for Research & Advanced Studies, ICREA, Pg. Lluís Companys 23, 08010 Barcelona, Spain

**Table of Contents**

[1. Materials and Methods S3](#_Toc216774469)

[2. Synthesis of BN-naphthalene substrates S4](#_Toc216774470)

[2.1 Synthesis of 1-iodo-[1,2]azaborinino[1,2-*a*][1,2]azaborinine (2) S4](#_Toc216774471)

[2.2 Synthesis of 1,9-dibromo-[1,2]azaborinino[1,2-*a*][1,2]azaborinine (1-Br_2_) S4](#_Toc216774472)

[2.3 Synthesis of 1-bromo-9-methyl-[1,2]azaborinino[1,2-*a*][1,2]azaborinine (1-Me-Br) S5](#_Toc216774473)

[2.4 Synthesis of 1-iodo-9-methyl-[1,2]azaborinino[1,2-*a*][1,2]azaborinine (5) S6](#_Toc216774474)

[2.5 Synthesis of 4-(trimethylsilyl)-[1,2]azaborinino[1,2-*a*][1,2]azaborinine (1-SiMe_3_) S6](#_Toc216774475)

[2.6 Synthesis of 4-iodo-[1,2]azaborinino[1,2-*a*][1,2]azaborinine (2-inv) S7](#_Toc216774476)

[3. Synthesis of imine and carbonyl substrates S8](#_Toc216774477)

[3.1 Silyl aldimines 3-Br, 3-Cl, 7, 3-helio, 11 and 14. General procedure A. S8](#_Toc216774478)

[3.2 Silyl ketimines 17-Me, 17-Bu and 17-Ph. General procedure B. S10](#_Toc216774479)

[3.3 Procedure for the preparation of 2-bromoterephthalaldehyde S11](#_Toc216774480)

[4. Study of the reaction parameters for the [4,2]-BN-Arex reaction S11](#_Toc216774481)

[5*.* BN-phenanthridines from halo(hetero)aryl silyl aldimines. General procedure C S13](#_Toc216774482)

[6. Synthesis of BN*-*phenanthridines using aryl silyl ketimines. General procedure D S18](#_Toc216774483)

[6.1 Reaction between 2 and 17-Me in the presence of norbornene (NBE) S20](#_Toc216774484)

[7. [2+2+4]-BN*-Arex* reaction between 2 and 17-Me or 17-Bu and norbornadiene (NBDE) S21](#_Toc216774485)

[8. Synthesis of BN-dibenzoazepine derivative (25) S22](#_Toc216774486)

[9. Synthesis of pleiadiene 1,2-azaborine (26) S23](#_Toc216774487)

[10. Study of the [3+2]-BN*-Arex* reaction with bromo (hetero)aryl aldehydes S24](#_Toc216774488)

[11. Synthesis of BN-containing fluorenones. General procedure E S25](#_Toc216774489)

[12. Post-synthetic modification of new BN-containing structures S31](#_Toc216774490)

[12.1 Iodination of BN-phenanthridine 4-inv and BN-fluorenone 27. General procedure F S31](#_Toc216774491)

[13. Synthetic elaboration of 1-iodo-BN-fluorenone 37 S32](#_Toc216774492)

[14. Synthesis of inverted BN-doped fluorenone species (27-inv) S34](#_Toc216774493)

[15. Characterization data S38](#_Toc216774494)

[16. Photophysical characterization S120](#_Toc216774495)

[17. DFT calculations S133](#_Toc216774496)

[18. Cyclic voltammetry measurements S138](#_Toc216774497)

[19. Chemical shift calculations for compound 22-b S140](#_Toc216774498)

[20. X-Ray diffraction structural determination details S142](#_Toc216774499)

[21. References S147](#_Toc216774500)

# Materials and Methods

*Reagents*. All commercially acquired reagents were used as received unless indicated otherwise.

*Reaction conditions****.*** Reactions requiring inert atmosphere were conducted under Argon atmosphere using standard Schlenk-line techniques. When indicated, reactions were conducted in standard tube-shaped Schlenk flasks sealable with a screw-on Teflon stopper valve. All other reactions were performed employing standard organic synthesis protocols.

*Chromatography.* Thin layer chromatography (TLC) was performed using Merck aluminum-backed plates of TLC Silica gel 60 F254; the plates were revealed using UV light at 254 nm or by staining using potassium permanganate. Standard flash column chromatography was accomplished using silica gel (60 Å pore size, 230-400 μm mesh size). Also, automated flash chromatography was used when necessary, employing Büchi Pure C-815 Flash with Büchi FlashPure EcoFlex silica cartridges (55-75 Å pore size, 40-63 μm particle size).

*Gas Chromatography coupled to Low Resolution Mass Spectrometry.* Routine GC-MS measurements were recorded on an Agilent 7890B chromatograph equipped with an Agilent 5977A Network MS detector and using a 30 m-long HP-5MS column.

*Gas Chromatography coupled to High Resolution Mass Spectrometry.* Analyses were carried out at the IQAC Mass Spectrometry Facility, using a Thermo Scientific Trace 1310 Gas Chromatograph equipped with MS/MS Q Exactive GC Orbitrap.

*Liquid Chromatography coupled to High-Resolution Mass Spectrometry.* Analyses were carried out at Institut Químic de Sarrià IQS-SCIEX DEMO LAB Facility, using a EXION LC (Sciex) chromatograph coupled to a QTOF X500B (Hybrid LC/MS/Ms quadrupole time-of-flight mass spectrometer). Direct injections were performed using as mobile phase A Milli-Q water (w/o 0.1% of formic acid) and as mobile phase B CH_3_CN (w/o 0.1% of formic acid) (50:50).

*Nuclear Magnetic Resonance.* Spectroscopic experiments for the characterization were carried out at the Structural Determination facility in the Organic and Pharmaceutical Chemistry Department at IQS School of Engineering both on a Varian Mercury 400 MHz (9.3950 T) instrument (400 MHz for ^1^H, 100.5 MHz for ^13^C, 376 MHz for ^19^F and 128 MHz for ^11^B) and a ECZL-R 600 MHz equipped with a 5 mm broadband Double Resonance N_2_ cryogenic SuperCool probe instrument (600 MHz for ^1^H, 151 MHz for ^13^C, 193 MHz for ^11^B) . The ^1^H and ^13^C chemical shifts (δ) are quoted in parts per million (ppm) and referenced to the appropriate NMR solvent peak(s), which for ^1^H measurements would correspond to the residual *proteo* component of the deuterated solvent. 2D-NMR experiments COSY, HSQC and HMBC were used where necessary in assigning NMR spectra. Spin-spin coupling constants (*J*) are reported in Hertz (Hz). Signal multiplicities are represented by s (singlet), d (doublet), t (triplet), q (quartet), dd (doublet of doublets), td (triplet of doublets), ddd (doublet of doublet of doublets), m (multiplet).

*Infrared spectra* were recorded both at the IQAC Infrared Spectrometry Facility on a Thermo Scientific Nicolet Avatar 360 FT-IR spectrophotometer and at the Structural Determination facility in the Organic and Pharmaceutical Chemistry Department at IQS School of Engineering on a Thermo Scientific Nicolet iS10 FT-IR equipped with Smart iTR window.

# 2. Synthesis of BN-naphthalene substrates

2.1 Synthesis of 1-iodo-[1,2]azaborinino[1,2-*a*][1,2]azaborinine (2)

An oven dried Schlenk tube equipped with a magnetic stir bar was purged with argon and then charged with grinded molecular iodine (1.30 g, 5.12 mmol, 2.2 equiv.) and anhydrous silver tosylate (715 mg, 2.56 mmol, 1.1 equiv.). Anhydrous dichloromethane (6 mL) was added, and the mixture was stirred for 30 minutes at room temperature. After cooling down to 0 °C, compound **1**^[1]^ (300 mg, 2.33 mmol, 1 equiv.) was added to the mixture. Additional 0.5 mL of dichloromethane were employed to rinse the walls of the reactor Schlenk tube. The mixture was stirred at this temperature for 30 minutes and then at room temperature for 2 hours protected from light. Subsequently, the mixture was diluted with dichloromethane and filtered through a pad of Celite^®^. The liquid organic mixture was then washed with a saturated solution of sodium sulphite to quench the excess of iodine (2x20 mL). The combined organic phase was dried over anhydrous magnesium sulphate, filtered, and evaporated under reduced pressure. Finally, the resulting residue was purified by automated flash chromatography (12 g cartridge, cyclohexane) affording product **2** as a light-yellow solid (505 mg, 1.98 mmol, 85% yield).

 (**2**) ^1^H NMR (400 MHz, CDCl_3_) δ 8.21 (d, *J* = 7.0 Hz, 1H**a**), 7.81 (d, *J* = 6.9 Hz, 1H**c**), 7.75 (dd, *J* = 11.1, 6.4 Hz, 1H**f**), 7.71 (d, *J* = 6.9 Hz, 1H**d**), 7.49 (d, *J* = 11.2 Hz, 1H**g**), 6.77 (td, *J* = 6.7, 1.3 Hz, 1H**e**), 6.47 (t, *J* = 7.0 Hz, 1H**b**).^13^C NMR (100.5 MHz, CDCl_3_) δ 146.0, 140.2, 134.4, 133.7, 115.3, 115.1 (signals for two carbons next to boron atom are not observed). ^11^B NMR (128 MHz, CDCl_3_) δ 29.0. FTIR (ATR) cm^-1^: 2922, 2853, 1602, 1486, 1404, 1330, 1231, 773, 737, 697. Data in agreement with literature values.^[2a]^

2.2 Synthesis of 1,9-dibromo-[1,2]azaborinino[1,2-*a*][1,2]azaborinine (1-Br_2_)

An oven dried Schlenk tube equipped with a magnetic stir bar was purged with argon, and then charged with aluminum bromide (3.64 g, 13.64 mmol, 2.2 equiv.) and previously recrystallized *N*-bromosuccinimide (NBS) (2.43 g, 13.64 mmol, 2.2 equiv.). Then, anhydrous dichloromethane (150 mL) was added, and the mixture was stirred for 30 minutes at room temperature. After cooling down to -35 °C, the mixture was added to a solution of **1** (800 mg, 6.20 mmol, 1.0 equiv.) in anhydrous dichloromethane (100 mL). The mixture was stirred at this temperature for 1 hour and then at room temperature for 4 hours. Then, water was added to the mixture (40 mL) and extractions with dichloromethane were performed (3x80 mL). The combined organic phase was dried over anhydrous MgSO_4_, filtered, and evaporated under reduced pressure. The resulting residue was purified by automated flash chromatography (25 g cartridge, cyclohexane) affording the dibrominated product (**1**-Br_2_) as a white solid (1.03 g, 3.59 mmol, 58% yield).

 (**1**-Br_2_) ^1^H NMR (400 MHz, CDCl_3_) δ 8.04 (d, *J* = 7.4 Hz, 2H**a**), 7.77 (d, *J* = 6.9 Hz, 2H**c**), 6.58 (t, *J* = 7.2 Hz, 2H**b**). Data in agreement with literature values.^[2a,b]^

2.3 Synthesis of 1-bromo-9-methyl-[1,2]azaborinino[1,2-*a*][1,2]azaborinine (1-Me-Br)

Dibrominated BN-naphthalene (**1**-Br_2_) (890 mg, 3.10 mmol, 1.0 equiv.) was dissolved in anhydrous tetrahydrofuran (27 mL) in an oven dried Schlenk tube equipped with a magnetic stir bar. Then, the solution was cooled to -78 °C in a dry ice/acetone bath and *t*-BuLi (1.7 M in pentane, 3.6 mL, 6.12 mmol, 2.0 equiv.) was added dropwise. The resulting solution was stirred vigorously at -78 °C for 30 minutes. After that time, iodomethane (1.9 mL, 31.0 mmol, 10 equiv.) was slowly added at this temperature *via* syringe and the resulting mixture was allowed to reach slowly room temperature. The mixture was left stirring at room temperature for additional 12 hours. Next, a saturated solution of NH_4_Cl was added and the mixture was stirred for 5 minutes. The organic layer was collected using a separatory funnel, and the aqueous layer was extracted with ethyl acetate (4x60 mL). The combined organic phase was dried over anhydrous magnesium sulphate, filtered, and evaporated under reduced pressure. The resulting residue was purified by automated flash chromatography (12 g cartridge, cyclohexane) affording the product **1**-Me-Br as a light brown solid (632 mg, 2.85 mmol, 92% yield).

 (**1**-Me-Br) ^1^H NMR (400 MHz, CDCl_3_) δ 7.95 (dd, *J* = 7.3, 1.0 Hz, 1H**f**), 7.76 (d, *J* = 7.0 Hz, 1H**d**), 7.64 (d, *J* = 6.9 Hz, 1H**c**), 7.44 (d, *J* = 6.8 Hz, 1H**a**), 6.63 (t, *J* = 6.9 Hz, 1H**b**), 6.51 (t, *J* = 7.1 Hz, 1H**e**), 2.77 (s, 3H**g**). ^13^C NMR (100.5 MHz, CDCl_3_) δ 141.3, 138.7, 134.4, 132.5, 114.4, 113.1, 24.9 (signals for quaternary carbons next to boron atom are not observed). ^11^B NMR (128 MHz, CDCl_3_) δ 27.7. GC-HRMS (orbitrap) *m*/*z* calcd. for C_9_H_9_BBrN: [M]^+•^ 221.0006, found 221.0005.

2.4 Synthesis of 1-iodo-9-methyl-[1,2]azaborinino[1,2-*a*][1,2]azaborinine (5)

An oven dried Schlenk tube equipped with a magnetic stir bar was charged with copper(I) iodide (35 mg, 0.18 mmol, 5 mol%), 1-bromo-9-methyl-[1,2]azaborinino[1,2-*a*][1,2]azaborinine (**1**-Me-Br) (800 mg, 3.60 mmol, 1 equiv.), sodium iodide (1.08 g, 7.21 mmol, 2.0 equiv.) and briefly evacuated and backfilled with argon. Then, degassed 1,4-dioxane (2 mL) and trans-*N*,*N*′-dimethylcyclohexane-1,2-diamine (57 μL, 0.36 mmol, 10 mol%) were added under argon. The reaction mixture was stirred at 110 °C for 24 hours. After that time, the mixture was allowed to reach room temperature, filtered through a pad of Celite^®^, and the pad was further washed with dichloromethane (3x20 mL). The combined organic phase was evaporated, and the resulting residue was purified by automated flash chromatography (12 g cartridge, cyclohexane) affording the product **5** as a light-yellow solid (793 mg, 2.95 mmol, 82% yield).

(**5**) ^1^H NMR (400 MHz, CDCl_3_) δ 8.37 (d, *J* = 7.2 Hz, 1H**f**), 7.79 (d, *J* = 6.9 Hz, 1H**d**), 7.63 (d, *J* = 6.9 Hz, 1H**c**), 7.46 (d, *J* = 6.9 Hz, 1H**a**), 6.62 (t, *J* = 6.9 Hz, 1H**b**), 6.36 (t, *J* = 7.0 Hz, 1H**e**), 2.83 (s, 3H**g**). ^13^C NMR (100.5 MHz, CDCl_3_) δ 149.0, 138.8, 135.3, 132.9, 114.5, 114.4, 25.6 (signals for quaternary carbons next to boron atom are not observed). ^11^B NMR (128 MHz, CDCl_3_) δ 27.8. GC-HRMS (orbitrap) *m*/*z* calcd. for C_9_H_9_BIN: [M]^+•^ 268.9867, found 268.9869.

2.5 Synthesis of 4-(trimethylsilyl)-[1,2]azaborinino[1,2-*a*][1,2]azaborinine (1-SiMe_3_)

An oven dried 50 mL Schlenk tube equipped with a magnetic stir bar was charged with BN-naphthalene (**1**) (100 mg, 0.78 mmol, 1.0 equiv.) and then the reactor was briefly evacuated and backfilled with argon. Then, anhydrous tetrahydrofuran (13 mL) was added to the flask, and the mixture was cooled to -78 °C. At this temperature, a solution of *t-*BuLi (1,7 M in pentane, 1.8 mL, 3.10 mmol, 4.0 equiv.) was added dropwise, and the mixture was left stirring at this temperature for 30 minutes before the addition of freshly distilled chlorotrimethylsilane (0.50 mL, 3.9 mmol, 5.0 equiv.). The mixture was stirred for another 1 hour at -78 °C and then left stirring overnight at room temperature. After, water was added to the flask to quench the reaction, and the aqueous phase was extracted with ethyl acetate (3x30 mL). Finally, the organic layers were combined, dried over anhydrous magnesium sulphate and filtered. The solvent was evaporated under reduced pressure, and the residue was purified by flash column chromatography (cyclohexane) to afford **1**-SiMe_3_ as a colorless oil (124 mg, 0.62 mmol, 79% yield).

(**1**-SiMe_3_) ^1^H NMR (400 MHz, CDCl_3_) δ 8.18 (d, *J* = 8.2 Hz, 1H**d**), 7.71 (dd, *J* = 11.6, 5.7 Hz, 1H**b**), 7.64 (dd, *J* = 11.0, 6.4 Hz, 1H**f**), 7.53 – 7.44 (m, 2H**a,g**), 7.03 (dd, *J* = 6.4, 1.6 Hz, 1H**e**), 6.74 (ddd, *J* = 7.1, 6.2, 1.7 Hz, 1H**c**), 0.49 (s, 9H**h**). ^13^C NMR (100.5 MHz, CDCl_3_) δ 146.6, 138.0, 137.2, 134.4, 124.1, 113.7, -0.0 (signals for two carbons next to boron atom are not observed). ^11^B NMR (128 MHz, CDCl_3_) 28.8. HRMS (ESI) *m*/*z* calcd. for C_11_H_17_BNSi: [M+H]^+^ 202.1218, found 202.1219.

2.6 Synthesis of 4-iodo-[1,2]azaborinino[1,2-*a*][1,2]azaborinine (2-inv)

An oven dried Schlenk tube equipped with a magnetic stir bar was charged with trimethylsilyl-BN-naphthalene (**1**-SiMe_3_) (166 mg, 0.826 mmol, 1.0 equiv.) and anhydrous dichloromethane (6 mL). Then, the solution was cooled to 0 °C and molecular iodine (195 mg, 1.156 mmol, 1.4 equiv.) was added to the flask. The mixture was left stirring at this temperature for 15 minutes and then at room temperature for 12 hours. After that time the mixture was diluted with dichloromethane, and the excess of iodine was quenched with a saturated solution of sodium thiosulphate. The combined organic phase was dried over anhydrous magnesium sulphate, filtered, and evaporated under reduced pressure. Finally, the resulting residue was purified by automated flash chromatography (4 g cartridge**,** cyclohexane) affording product **2-inv** as a light-yellow oil (130 mg, 0.510 mmol, 62% yield).

(**2-inv**) ^1^H NMR (400 MHz, CDCl_3_) δ 8.31 (d, *J* = 7.3 Hz, 1H**d**), 7.72 (dd, *J* = 11.0, 6.4 Hz, 1H**b**), 7.53 (dd, *J* = 6.7, 1.6 Hz, 1H**f**), 7.48 (d, *J* = 11.0 Hz, 1H**g**), 7.40 – 7.33 (m, 2H**a,** **e**), 6.85 (ddd, *J* = 7.3, 6.3, 1.6 Hz, 1H**c**). ^13^C NMR (100.5 MHz, CDCl_3_) δ 139.6, 138.8, 138.7, 132.9 (broad signal, adjacent to the boron atom), 129.2, 115.5, 101.3 (signal for one carbon next to boron atom is not observed). ^11^B NMR (128 MHz, CDCl_3_) δ 30.0. GC-HRMS (orbitrap) *m*/*z* calcd. for C_8_H_7_BIN: [M]^+•^ 254,9711, found 254.9711.

# 3. Synthesis of imine and carbonyl substrates

3.1 Silyl aldimines 3-Br, 3-Cl, 7, 3-helio, 11 and 14. General procedure A.

Hexamethyldisilazane (462 mg, 0.60 mL, 2.86 mmol, 1.1 equiv.) was transferred to an oven dried 25 mL round bottomed flask with magnetic stir bar and septum under argon atmosphere. Once the flask was cooled to 0 °C, *n*-BuLi in hexanes (1.6 M, 1.8 mL, 2.86 mmol, 1.1 equiv.) was added dropwise. The solution was allowed to reach room temperature and then was stirred for 15 minutes. Then, the solution was cooled to 0 °C followed by the addition of the corresponding bromo aryl -or heteroaryl- aldehyde (2.60 mmol, 1.0 equiv.) which results in an immediate color change. The solution was then allowed to reach room temperature and stirred for 18 hours. The solvent was removed by evaporation, and the crude reaction mixture was purified either by Kugelrohr distillation, or by anhydrous hexane solid-liquid fast extraction.

(**3**-Br) Following the *General procedure A* using 2-bromobenzaldehyde (481 mg, 303 μL, 2.60 mmol, 1.0 equiv.) as carbonyl derivative. The crude reaction mixture was purified *via* Kugelrohr distillation (90 °C/0.5 mmHg) affording 413 mg (1.61 mmol, 62% yield) of **3**-Br as a yellow oil.

^1^H NMR (400 MHz, CDCl_3_) δ 9.26 (s, 1H), 8.01 (dt, *J* = 7.5, 1.4 Hz, 1H), 7.56 (d, *J* = 8.0 Hz, 1H), 7.34 (t, *J* = 7.4 Hz, 1H), 7.30 – 7.22 (m, 1H), 0.28 (s, 9H). Data in agreement with literature values.^[3]^

(**3**-Cl) Following the *General procedure A* using 2-chlorobenzaldehyde (366 mg, 293 μL, 2.60 mmol, 1.0 equiv.) as carbonyl derivative. The crude reaction mixture was purified *via* Kugelrohr distillation (100 °C/0.5 mmHg) affording 409 mg (1.61 mmol, 69% yield) of **3**-Cl as a yellow oil.

^1^H NMR (400 MHz, CDCl_3_) δ 9.37 (s, 1H), 8.08 – 7.99 (m, 1H), 7.42 – 7.28 (m, 3H), 0.28 (s, 9H). Data in agreement with literature values.^[3]^

(**7**) Following the *General procedure A* using 2-bromo-5-(trifluoromethyl)benzaldehyde (385 μL, 658 mg, 2.60 mmol, 1.0 equiv.) as carbonyl derivative. The crude reaction mixture was purified *via* Kugelrohr distillation (90 °C/0.5 mmHg) affording 632 mg (1.95 mmol, 75% yield) of **7** as a yellow oil which becomes a wax at 5-10 °C.

^1^H NMR (400 MHz, CDCl_3_) δ 9.24 (s, 1H), 8.28 (dd, ^4^*J_H-F_* = 2.5, ^4^*J_H-H_* 0.6 Hz, 1H), 7.70 (d, *J* = 8.4 Hz, 1H), 7.52 (ddd, *J* = 8.4, ^4^*J_H-F_* = 2.4, ^4^*J_H-H_* = 0.7 Hz, 1H), 0.29 (s, 9H). Data in agreement with literature values.^[3]^

(**3**-helio) Following the *General procedure A* using 6-bromo-1,3-benzodioxole-5-carboxaldehyde (595 mg, 2.60 mmol, 1 equiv.) as carbonyl derivative. The crude reaction mixture was purified *via* hexane extraction: first, the solvent was removed by evaporation and then the residue was extracted with anhydrous hexane (3 x 5 mL). The extracted organic phases were evaporated affording 744 mg (2.48 mmol, 95% yield) of **3**-helio as a light pink solid.

^1^H NMR (400 MHz, CDCl_3_) δ 9.11 (s, 1H), 7.52 (s, 1H), 7.00 (s, 1H), 6.00 (s, 2H), 0.25 (s, 9H). ^13^C NMR (100.5 MHz, CDCl_3_) δ 166.8, 151.0, 148.0, 130.8, 119.1, 112.7, 107.8, 102.3, -0.9.

(**11**) Following the *General procedure A* using 3-bromothiophene-2-carbaldehyde (497 mg, 2.60 mmol, 1.0 equiv.) as carbonyl derivative. The crude reaction mixture was purified *via* Kugelrohr distillation (110 °C/0.5 mmHg) affording 273 mg (1.04 mmol, 40% yield) of **11** as a yellow oil.

^1^H NMR (400 MHz, CDCl_3_) δ 8.96 (d, *J* = 1.1, 1H), 7.41 (dd, *J* = 5.2, 1.1 Hz, 1H), 7.04 (d, *J* = 5.3 Hz, 1H), 0.25 (s, 9H). ^13^C NMR (100.5 MHz, CDCl_3_) δ 159.9, 140.6, 131.2, 130.0, 115.0, -1.0.

(**14**) Following the *General procedure A* albeit in a smaller scale. Hence, after the addition of *n*-BuLi (0.9 mL, 1.43 mmol, 1.1 equiv.) to hexamethyldisilazane (0.3 mL, 1.43 mmol, 1.1 equiv.) at 0 °C the mixture was left stirring 15 minutes at room temperature. Then, the solution was cooled to -78 °C and it turned into a cloudy white suspension. At this point 2-bromonicotinaldehyde (242 mg, 1.3 mmol, 1.0 equiv.) was added observing no color change. The suspension was left stirring 2 hours at -60 °C and then color changes into light yellow when warmed up to -35 °C. The solution was allowed to warm up slowly to room temperature and was left stirring for 18 hours. The resulting dark purple solution was evaporated, and the crude reaction mixture was purified *via* anhydrous hexane extraction (3 x 3 mL) affording 251 mg (0.98 mmol, 75% yield) of **14** as a dark brown solid.

^1^H NMR (400 MHz, CDCl_3_) δ 9.15 (s, 1H), 8.40 (dd, *J* = 4.7, 2.1 Hz, 1H), 8.26 (dd, *J* = 7.6, 2.1 Hz, 1H), 7.31 (dd, *J* = 7.6, 4.7 Hz, 1H), 0.26 (s, 9H). ^13^C NMR (100.5 MHz, CDCl_3_) δ 165.9, 152.0, 145.0, 137.0, 133.5, 123.3, -1.1.

3.2 Silyl ketimines 17-Me, 17-Bu and 17-Ph. General procedure B.

In an oven dried 25 mL round bottomed flask with magnetic stir bar and septum 2-chlorobenzonitrile (500 mg, 3.63 mmol, 1.0 equiv.) was dissolved in anhydrous tetrahydrofuran (7.3 mL, 0.5 M) under argon atmosphere. Once the flask was cooled to -78 °C, the desired organolithium reagent was added dropwise (3.99 mmol, 1.1 equiv.) resulting in a clear color change which persisted throughout the addition. The solution was stirred at this temperature for 4 hours. After this time, freshly distilled trimethylsilyl chloride (591 mg, 690 μL, 5.44 mmol, 1.5 equiv.) was added *via* syringe and the solution was allowed to reach room temperature. The resulting mixture was stirred at room temperature for 18 hours. After this time, the salts were decanted, and the supernatant was transferred *via* cannula into another flask. Then, the solvent was removed by evaporation leaving a residue which was purified by Kugelrohr distillation.

(**17-Me**, mixture of tautomers) Following the *General procedure B* using 2-chlorobenzonitrile and methyl lithium (1.6 M in diethyl ether, 2.5 mL). The crude reaction mixture was purified *via* Kugelrohr distillation (85-90 °C/0.5 mmHg) affording 697 mg (3.09 mmol, 85% yield) of a colorless oil which corresponds to a mixture of two isomers (~1:1 NMR ratio of imine:enamine).

^1^H NMR (400 MHz, CDCl_3_) δ 7.42 – 7.18 (m, 8H, enamine + ketimine), 4.25 (s, 1H, enamine), 3.87 (d, *J* = 2.1 Hz, 1H, enamine), 2.91 (broad signal, 1H, enamine), 2.38 (s, 3H, ketimine), 0.22 (s, 9H, enamine), 0.03 (s, 9H, ketimine). ^13^C NMR (100.5 MHz, CDCl_3_, enamine + ketimine) δ 175.8, 148.4, 144.0, 141.5, 132.3, 130.8, 129.6, 129.2, 129.0, 127.3, 126.7, 88.4, 31.3, 0.3, -0.3. ^1^H NMR data of silyl enamine in agreement with literature values.^[3]^

(**17-Bu**) Following the *General procedure B* using 2-chlorobenzonitrile and *n*-BuLi (1.6 M in hexanes, 3.99 mmol, 2.5 mL). The crude reaction mixture was purified *via* Kugelrohr distillation (90-95 °C/0.5 mmHg) affording 778 mg (2.90 mmol, 80% yield, purity 88%) of **17-Bu** as a colorless oil lightly contaminated by E/Z silyl enamine isomers (12% of the product, ~1:1 NMR ratio).

^1^H NMR (400 MHz, CDCl_3_) δ 7.33 – 7.29 (m, 1H), 7.23 – 7.19 (m, 2H), 7.03 – 6.98 (m, 1H), 2.62 – 2.55 (m, 2H), 1.59 – 1.49 (m, 2H), 1.41 – 1.27 (m, 2H), 0.88 (t, *J* = 7.3 Hz, 3H), -0.06 (s, 9H). ^13^C NMR (100.5 MHz, CDCl_3_) δ 178.5, 143.4, 129.8, 129.6, 128.9, 127.7, 126.6, 44.1, 28.2, 22.7, 14.1, 0.2.

(**17-Ph**) Phenyl lithium was synthesized in a Schlenk tube by adding *n-*BuLi (1.6 M in hexanes, 3.15 mL, 5.04 mmol, 1.2 equiv.) at 0 °C to a solution of bromobenzene (659 mg, 442 μL, 4.20 mmol, 1 equiv.) in anhydrous diethyl ether (14 mL, 0.36 M). The mixture was left stirring for 30 minutes at this temperature. Then, 2/3 of the mixture was evaporated and cooled to -78 °C. Following the scale of the *General procedure B* described above, a solution of chlorobenzonitrile in tetrahydrofuran was added to the phenyl lithium solution at this temperature *via* cannula and left stirring for 4 hours before the addition of trimethylsilyl chloride. The crude reaction mixture was purified *via* Kugelrohr distillation (110-120 °C/0.5 mmHg) affording 910 mg (3.16 mmol, 75% yield over two steps) of **17-Ph** as a yellow oil.

^1^H NMR (400 MHz, CDCl_3_) δ 7.71 – 7.67 (m, 2H), 7.43 – 7.29 (m, 6H), 7.19 – 7.15 (m, 1H), 0.01 (s, 9H). ^13^C NMR (100.5 MHz, CDCl_3_) δ 171.1, 141.2, 139.9, 131.1, 130.8, 129.6, 129.5, 128.9, 128.4, 128.3, 126.7, 0.2. ^1^H NMR data in agreement with literature values.^[3]^

3.3 Procedure for the preparation of 2-bromoterephthalaldehyde

In a 100 mL round-bottomed flask terephthalaldehyde (1.5 g, 11.2 mmol, 1.0 equiv.) was dissolved in 15 mL of concentrated sulphuric acid (96%). Then, previously recrystallized *N*-bromosuccinimide (498 mg, 2.80 mmol, 0.5 equiv.) was added portion wise over 30 minutes and the mixture was left 5 hours at 70 °C. The reaction mixture was cooled to room temperature and iced water was added. The resulting precipitate was collected by filtration and washed with iced water. Then, the crude product was dissolved in dichloromethane and washed with saturated sodium bicarbonate and brine. Finally, the combined organic phase was dried over anhydrous magnesium sulphate, filtered, and evaporated under reduced pressure. Recrystallization from ethyl acetate allowed the isolation of the dibrominated side-product, which was removed from the mother liquor by filtration. Then, the mother liquor was evaporated, and the residue was purified by automated flash chromatography (12 g cartridge, cyclohexane) affording 447 mg (2.24 mmol, 20% yield) as a white solid.

 ^1^H NMR (400 MHz, CDCl_3_) δ 10.43 (d, *J* = 0.8 Hz, 1H), 10.06 (s, 1H), 8.16 (d, *J* = 1.4 Hz, 1H), 8.06 (d, *J* = 7.9, 1H), 7.92 (ddd, *J* = 7.9, 1.5, 0.8 Hz, 1H). ^13^C NMR (101 MHz, CDCl_3_) δ 191.2, 190.1, 140.8, 137.2, 134.9, 130.7, 128.7, 127.5. Data in agreement with literature values.^[4a,4b]^

4. Study of the reaction parameters for the [4,2]-BN-Arex reaction

General screening conditions:

To a 25 mL Schlenk flask containing a magnetic stir bar, palladium acetate (4.5 mg, 10 mol%), phosphine (22 mol%), norbornene (NBE, 3-8 equiv.) and cesium carbonate (195 mg, 0.6 mmol, 3.0 equiv.) were added. Then, dimethoxyethane or acetonitrile (3 mL) was added, and the mixture was left stirring for 10 minutes. A solution of bromo phenyl silyl aldimine **3**-Br (0.3 mmol, 1.5 equiv.) in 1.0 mL of dimethoxyethane or acetonitrile and 1-iodo-[1,2]azaborinino[1,2-*a*][1,2]azaborinine **2** (0.2 mmol, 1.0 equiv.) were subsequently added. The resulting mixture was stirred first at room temperature for 5 minutes and then at 90 °C in a pre-heated oil-bath for 12 hours. The mixture was then allowed to reach room temperature and filtered through a pad of Celite^®^, and the pad was further washed with dichloromethane (3x15 mL). Organic solvent from the filtrate was removed by rotary evaporation, thus providing a residue that it was subsequently purified by automated flash chromatography (4 g cartridge, 95:5, cyclohexane:ethyl acetate – cyclohexane containing 1% of Et_3_N). In some cases, additional recrystallization from hexane was needed to obtain pure products.

From Entry 1, the side-product **S1** has been also isolated by automated flash chromatography (cyclohexane) affording 9 mg (0.04 mmol, 20% yield) as a colorless oil.

 (**S1**) ^1^H NMR (400 MHz, CDCl_3_) δ 7.78 (d, *J* = 6.9 Hz, 1H**d**), 7.70 dd, *J* = 11.9, 7.0 Hz, 1H**b**), 7.60 (d, *J* = 6.9 Hz, 1H**e**), 7.52 (d, *J* = 11.2 Hz, 1H**a**), 7.35 (d, *J* = 6.8 Hz, 1H**g**), 6.70 (td, *J* = 6.6, 1.5 Hz, 1H**c**), 6.64 (t, *J* = 6.9 Hz, 1H**f**), 3.33 – 3.25 (m, 1H**h**), 2.46 – 2.33 (m, 2H), 1.83 – 1.50 (m, 7H), 1.21 – 1.06 (m, 1H). ^13^C NMR (100.5 MHz, CDCl_3_) δ 138.3, 134.3, 131.2, 130.0, 113.8, 113.7, 45.2, 42.7, 38.7, 37.4, 35.7, 30.6, 29.5 (signals for two carbons next to boron atom are not observed). ^11^B NMR (128 MHz, CDCl_3_) δ 24.7. GC-HRMS (orbitrap) *m*/*z* calcd. for C_15_H_18_BN: [M]^+•^ 223.1527, found 223.1527. FTIR (ATR) cm^-1^: 2947, 2923, 2865, 1611, 1449, 1414, 1236, 766, 737, 715.

# 5*.* BN-phenanthridines from halo(hetero)aryl silyl aldimines. General procedure C

To a 25 mL Schlenk flask containing a magnetic stir bar, palladium acetate (4.5 mg, 10 mol%), tri-(2-furyl)phosphine (10 mg, 22 mol%), norbornene (NBE, 94 mg, 1.0 mmol, 5.0 equiv.) and cesium carbonate (195 mg, 0.6 mmol, 3.0 equiv.) were added. Then, dimethoxyethane (DME, 3 mL) was added, and the mixture was left stirring for 10 minutes. A solution of bromo (hetero)aryl silyl aldimine (0.3 mmol, 1.5 equiv.) in 0.8 mL of dimethoxyethane and BN-iodoarene (0.2 mmol, 1.0 equiv.) were subsequently added. Extra 0.2 mL of dimethoxyethane were used to rinse the Schlenk walls and the resulting mixture was stirred first at room temperature for 5 minutes and then at 90 °C in a pre-heated oil-bath for 12 hours. The mixture was then allowed to reach room temperature and filtered through a pad of Celite^®^, and the pad was further washed with dichloromethane (3 x 15 mL). Organic solvent from the filtrate was removed by rotary evaporation, thus providing a residue which was subsequently purified by automated flash chromatography (4 g cartridge). In some cases, additional recrystallization from hexane was needed to obtain pure products.

(**4**) Following the *General procedure C*. Reaction between 1-iodo-[1,2]azaborinino[1,2-*a*][1,2]azaborinine (**2**) (51 mg, 0.2 mmol) and (*E*)-1-(2-bromophenyl)-*N*-(trimethylsilyl)methanimine (**3-**Br) (77 mg, 0.3 mmol). The product was purified by automated flash chromatography (95:5, cyclohexane:ethyl acetate – cyclohexane containing 1% of Et_3_N) affording 23 mg (0.100 mmol, 50% yield) of **4** as a brown solid.

^1^H NMR (400 MHz, CDCl_3_) δ 9.53 (s, 1H**k**), 8.52 (d, *J* = 8.4 Hz, 1H**g**), 8.26 (d, *J* = 10.5 Hz, 1H**a**), 8.12 (d, *J* = 1.0 Hz, 1H**j**), 8.00 – 7.91 (m, 2H**b,e**), 7.91 (d, *J* = 6.9 Hz, 1H**d**), 7.87 (ddd, *J* = 8.3, 6.9, 1.4 Hz, 1H**i**), 7.78 – 7.70 (m, 2H**f,h**), 6.86 (t, *J* = 1.5 Hz, 1H**c**). ^13^C NMR (100.5 MHz, CDCl_3_) δ 151.8, 140.6, 133.8, 133.2, 131.5, 130.6, 129.5, 128.8, 128.1, 128.0, 122.3, 114.5, 108.2 (signals for two carbons next to boron atom are not observed). ^11^B NMR (128 MHz, CDCl_3_) δ 25.4. GC-HRMS (orbitrap) *m*/*z* calcd. for C_15_H_11_BN_2_: [M]^+•^ 230.1010, found 230.1009. FTIR (ATR) cm^-1^: 2953, 2217, 1628, 1604, 1536, 1423, 1242, 758, 650.

(**6**) Following *General procedure C* (albeit at double scale). Reaction between 1-iodo-9-methyl-[1,2]azaborinino[1,2-*a*][1,2]azaborinine (**5**) (108 mg, 0.4 mmol) and (*E*)-1-(2-bromophenyl)-*N*-(trimethylsilyl)methanimine (**3**-Br) (154 mg, 0.6 mmol). The product was purified by automated flash chromatography (95:5, cyclohexane:ethyl acetate – cyclohexane containing 1% of Et_3_N) affording 78 mg (0.320 mmol, 80% yield) of **6** as a brown solid.

^1^H NMR (400 MHz, CDCl_3_) δ 9.55 (s, 1H**k**), 8.54 (d, *J* = 8.4 Hz, 1H**g**), 8.12 (d, *J* = 8.0 Hz, 1H**j**), 7.96 (d, *J* = 7.5 Hz, 1H**e**), 7.87 (t, *J* = 7.1 Hz, 1H**h**), 7.80 – 7.71 (m, 3H**d,f,i**), 7.62 (d, *J* = 6.8 Hz, 1H**b**), 6.73 (t, *J* = 6.8 Hz, 1H**c**), 3.11 (s, 3H).^13^C NMR (100.5 MHz, CDCl_3_) δ 150.9, 139.2, 133.5, 132.1, 131.3, 130.2, 129.6, 128.5, 127.9, 127.4, 122.3, 113.7, 107.8, 24.6 (signals for quaternary carbons next to boron atom are not observed). ^11^B NMR (128 MHz, CDCl_3_) δ 29.1. GC-HRMS (orbitrap) *m*/*z* calcd. for C_16_H_13_BN_2_: [M]^+•^ 244.1166, found 244.1166. FTIR (ATR) cm^-1^: 3062, 2952, 2893, 1606, 1422, 1379, 1246, 810, 778, 749, 723, 651, 601.

(**9**) Following *General procedure C* employing 20 mol% of palladium acetate (9 mg) and 44 mol% of tri-(2-furyl)phosphine (20 mg). Reaction between 1-iodo-9-methyl-[1,2]azaborinino[1,2-*a*][1,2]azaborinine (**5**) (54 mg, 0.2 mmol) and (*E*)-1-(2-bromo-5-(trifluoromethyl)phenyl)-*N*-(trimethylsilyl)methanimine (**7**) (97 mg, 0.3 mmol). The product was purified by automated flash chromatography (cyclohexane containing 1% of Et_3_N) affording 49 mg (0.158 mmol, 79% yield) of **9** as a cream colored solid.

^1^H NMR (400 MHz, CDCl_3_) δ 9.56 (s, 1H**j**), 8.60 (d, *J* = 8.6 Hz, 1H**g**), 8.37 (s, 1H**i**), 8.00 (dd, *J* = 8.8, 1.9 Hz, 1H**h**), 7.95 (d, *J* = 7.6 Hz, 1H**e**), 7.75 (d, *J* = 6.8 Hz, 1H**d**), 7.66 (d, *J* = 7.6 Hz, 1H**f**), 7.63 (dt, *J* = 6.8, 1.2 Hz, 1H**b**), 6.75 (t, *J* = 6.8 Hz, 1H**c**), 3.08 (s, 3H**a**). ^13^C NMR (100.5 MHz, CDCl_3_) δ 150.7, 144.4 (broad signal, CH_3_-**C** adjacent to the boron atom), 139.6, 134.1, 132.9, 132.0, 129.6 (q, ^2^*J_C-F_* = 33.0 Hz, CF_3_-**C**), 128.9, 126.2, 126.0 (q, ^3^*J_C-F_* = 4.3 Hz, C-**i**), 125.8 (d, ^3^*J_C-F_* = 3.1 Hz, C-**h**), 124.1 (q, ^1^*J_C-F_* = 272.3 Hz, **C**-F_3_), 123.6, 114.1, 107.4, 24.5 (signal for N=**C** carbon next to boron atom is not observed). ^19^F NMR (376 MHz, CDCl_3_) δ -62.4. ^11^B NMR (128 MHz, CDCl_3_) δ 29.5. GC-HRMS (orbitrap) *m*/*z* calcd. for C_17_H_12_BF_3_N_2_: [M]^+•^ 312.1040, found 312.1034. FTIR (ATR) cm^-1^: 2919, 2853, 1633, 1606, 1453, 1375, 1324, 1290, 1193, 1158, 1122 (C-F *st*), 1067, 804, 782, 755, 670.

(**10**) Following *General procedure C*. Reaction between 1-iodo-9-methyl-[1,2]azaborinino[1,2-*a*][1,2]azaborinine (**5**) (54 mg, 0.2 mmol) and (*E*)-1-(6-bromobenzo[*d*][1,3]dioxol-5-yl)-*N*-(trimethylsilyl)methanimine (**3**-helio) (90 mg, 0.3 mmol). The product was purified by automated flash chromatography (95:5, cyclohexane:ethyl acetate – cyclohexane containing 1% of Et_3_N) affording 27 mg (0.094 mmol, 47% yield) of **10** as a cream colored solid.

^1^H NMR (400 MHz, CD_2_Cl_2_) δ 9.29 (s, 1H**j**), 7.93 (d, *J* = 7.6 Hz, 1H**e**), 7.84 (s, 1H**g**), 7.78 (d, *J* = 6.7 Hz, 1H**d**), 7.59 (dt, *J* = 6.7, 1.3 Hz, 1H**b**), 7.54 (d, *J* = 7.6 Hz, 1H**f**), 7.38 (s, 1H**i**), 6.71 (t, *J* = 6.8 Hz, 1H**c**), 6.18 (s, 2H**h**), 3.02 (s, 3H**a**). ^13^C NMR (100.5 MHz, CD_2_Cl_2_) δ 151.4, 149.4, 149.1, 139.4, 133.5, 132.5, 129.8, 129.3, 125.1, 113.6, 108.2, 104.7, 102.4, 99.8, 24.5 (signal for quaternary carbons next to boron atom are not observed). ^11^B NMR (128 MHz, CDCl_3_) δ 29.4. GC-HRMS (orbitrap) *m*/*z* calcd. for C_17_H_13_BN_2_O_2_: [M]^+•^ 288.1065, found 288.1061. FTIR (ATR) cm^-1^: 2919, 2853, 1613, 1496, 1459, 1431, 1376, 1256, 1225, 1032 (C-O-C as *st*), 941, 832, 802, 751, 716, 686.

(**12**) Following *General procedure C*. Reaction between 1-iodo-[1,2]azaborinino[1,2-*a*][1,2]azaborinine (**2**) (51 mg, 0.2 mmol) and (*E*)-1-(3-bromothiophen-2-yl)-*N*-(trimethylsilyl)methanimine (**11**) (79 mg, 0.3 mmol). The product was purified by automated flash chromatography (95:5, cyclohexane:ethyl acetate – cyclohexane containing 1% of Et_3_N) affording 34 mg (0.144 mmol, 72% yield) of **12** as a creamy colored solid.

^1^H NMR (400 MHz, CDCl_3_) δ 9.55 (d, *J* = 0.8 Hz, 1H**i**), 8.24 (d, *J* = 11.2 Hz, 1H**a**), 7.95 (dd, *J* = 11.8, 5.9 Hz, 1H**b**), 7.92 – 7.82 (m, 4H**d**,**e**,**g**,**h**), 7.44 (d, *J* = 7.4 Hz, 1H**f**), 6.84 (td, *J* = 6.7, 1.5 Hz, 1H**c**). ^13^C NMR (100.5 MHz, CDCl_3_) δ 143.6, 141.0, 140.7, 136.4, 134.0, 133.3, 131.7, 129.3, 121.4, 114.4, 109.7 (signals for two carbons next to boron atom are not observed). ^11^B NMR (128 MHz, CDCl_3_) δ 29.9. HRMS (ESI) *m*/*z* calcd. for C_13_H_10_BN_2_S: [M+H]^+^ 237.0653, found 237.0649. FTIR (ATR) cm^-1^: 3062, 2923, 2852, 1630, 1607, 1555, 1522, 1487, 1448, 1401, 1234, 1214, 929, 852, 818, 773, 738.

(**13**) Following *General procedure C*. Reaction between 1-iodo-9-methyl-[1,2]azaborinino[1,2-*a*][1,2]azaborinine (**5**) (54 mg, 0.2 mmol) and (*E*)-1-(3-bromothiophen-2-yl)-*N*-(trimethylsilyl)methanimine (**11**) (79 mg, 0.3 mmol). The product was purified by recrystallization in cyclohexane affording 44 mg (0.176 mmol, 88% yield) of **13** as a light brown solid.

^1^H NMR (400 MHz, CDCl_3_) δ 9.53 (s, 1H**i**), 7.85 (dd, *J* = 5.4, 0.9 Hz, 1H**h**), 7.82 (d, *J* = 7.4 Hz, 1H**e**), 7.78 (d, *J* = 5.3 Hz, 1H**g**), 7.69 (d, *J* = 6.8 Hz, 1H**d**), 7.60 (d, *J* = 6.8 Hz, 1H**b**), 7.37 (d, *J* = 7.4 Hz, 1H**f**), 6.68 (t, *J* = 6.8 Hz, 1H**c**), 3.08 (s, 3H**a**). ^13^C NMR (100.5 MHz, CDCl_3_) δ 143.0, 140.2, 139.4, 135.8, 133.5, 132.3, 130.5, 129.6, 121.5, 113.4, 109.3, 24.5 (signals for quaternary carbons next to boron atom are not observed). ^11^B NMR (128 MHz, CDCl_3_) δ 29.4. GC-HRMS (orbitrap) *m*/*z* calcd. for C_14_H_11_BN_2_S: [M]^+•^ 250.0731, found 250.0729. FTIR (ATR) cm^-1^: 2932, 1634, 1448, 1425, 1377, 1354, 1211, 924, 886, 785, 159, 728.

(**15**) Following *General procedure C* albeit employing 30 mol% of palladium acetate (13.5 mg) and 66 mol% of tri-(2-furyl)phosphine (30 mg). Reaction between 1-iodo-[1,2]azaborinino[1,2-*a*][1,2]azaborinine (**2**) (51 mg, 0.2 mmol) and (*E*)-1-(2-bromopyridin-3-yl)-*N*-(trimethylsilyl)methanimine (**14**) (77 mg, 0.3 mmol). The product was purified by automated flash chromatography (80:20, cyclohexane:ethyl acetate – cyclohexane containing 1% of Et_3_N) affording 21.5 mg (0.093 mmol, 47% yield) of **15** as a brown solid.

^1^H NMR (400 MHz, CDCl_3_) δ 9.54 (s, 1H**j**), 9.23 (dd, *J* = 4.3, 1.8 Hz, 1H**g**), 8.43 (dd, *J* = 8.2, 1.8 Hz, 1H**i**), 8.40 (d, *J* = 7.5 Hz, 1H**e**), 8.26 (d, *J* = 11.2 Hz, 1H**a**), 8.06 (d, *J* = 7.4 Hz, 1H**f**), 8.03 – 7.94 (m, 2H**b,d**), 7.69 (dd, *J* = 8.1, 4.3 Hz, 1H**h**), 6.91 (td, *J* = 6.7, 1.5 Hz, 1H**c**). ^13^C NMR (100.5 MHz, CDCl_3_) δ 153.6, 151.4, 146.9, 140.9, 136.2, 134.2, 133.6, 130.9, 123.3, 122.8, 114.8, 108.5 (signals for two carbons next to boron atom are not observed). ^11^B NMR (128 MHz, CDCl_3_) δ 29.7. HRMS (ESI) *m*/*z* calcd. for C_14_H_11_BN_3_: [M+H]^+^ 232.1041, found 232.1038. FTIR (ATR) cm^-1^: 2952, 1626, 1590, 1487, 1390, 802, 776, 741, 678, 654.

(**16**) Following *General procedure C* employing 30 mol% of palladium acetate (13.5 mg) and 66 mol% of tri-(2-furyl)phosphine (30 mg). Reaction between 1-iodo-9-methyl-[1,2]azaborinino[1,2-*a*][1,2]azaborinine (**5**) (54 mg, 0.2 mmol) and (*E*)-1-(2-bromopyridin-3-yl)-*N*-(trimethylsilyl)methanimine (**14**) (77 mg, 0.3 mmol). The product was purified by recrystallization with ethyl acetate affording 40 mg (0.163 mmol, 82% yield) of **16** as a brown solid.

^1^H NMR (400 MHz, DMSO-*d*_6_) δ 9.62 (s, 1H**j**), 9.28 (dd, *J* = 4.3, 1.8 Hz, 1H**g**), 8.69 (dd, *J* = 8.1, 1.8 Hz, 1H**i**), 8.40 (d, *J* = 7.5 Hz, 1H**e**), 8.33 (d, *J* = 7.4 Hz, 1H**f**), 8.19 (d, *J* = 6.8 Hz, 1H**d**), 7.88 (dd, *J* = 8.1, 4.3 Hz, 1H**h**), 7.61 (dt, *J* = 6.7, 1.2 Hz, 1H**b**), 6.84 (t, *J* = 6.8 Hz, 1H**c**), 3.00 (s, 3H**a**). ^13^C NMR (100.5 MHz, DMSO-*d*_6_) δ 153.9, 150.7, 145.5, 139.5, 136.2, 134.4, 133.1, 130.4, 123.9, 121.7, 113.6, 107.3, 24.2 (signals for quaternary carbons next to boron atom are not observed). ^11^B NMR (128 MHz, DMSO-*d*_6_) δ 24.3. GC-HRMS (orbitrap) *m*/*z* calcd. for C_15_H_12_BN_3_: [M]^+•^ 245.1119, found 245.1117. FTIR (ATR) cm^-1^: 3056, 2943, 1632, 1591, 1504, 1482, 1426, 1379, 1232, 1154, 947, 892, 851, 783, 734, 654.

(**4-inv**) Following *General procedure C*. Reaction between 4-iodo-[1,2]azaborinino[1,2-*a*][1,2]azaborinine (**2-inv**) and (*E*)-1-(2-bromophenyl)-*N*-(trimethylsilyl)methanimine (**3**-Br). The product was purified by automated flash chromatography (85:15, cyclohexane:ethyl acetate – cyclohexane containing 1% of Et_3_N) affording 37 mg (0.162 mmol, 81% yield) of **4-inv** as a light-yellow solid.

^1^H NMR (400 MHz, CDCl_3_) δ 9.87 (d, *J* = 7.4 Hz, 1H**d**), 9.28 (s, 1H**e**), 8.88 (d, *J* = 11.7 Hz, 1H**j**), 8.62 (d, *J* = 8.5 Hz, 1H**i**), 8.09 (d, *J* = 7.3 Hz, 1H**f**), 7.93 – 7.80 (m, 2H**b,h**), 7.66 – 7.61 (m, 2H**g,k**), 7.48 (ddd, *J* = 11.0, 1.6, 0.7 Hz, 1H**a**), 6.98 – 6.89 (m, 1H**c**). ^13^C NMR (100.5 MHz, CDCl_3_) δ 150.6, 143.5, 140.9, 134.9, 133.5, 131.2, 128.9, 128.7, 126.2, 126.2, 122.4, 114.6, 114.2 (signals for two carbons next to boron atom are not observed). ^11^B NMR (128 MHz, CDCl_3_) δ 28.9. HRMS (ESI) *m*/*z* calcd. for C_15_H_12_BN_2_: [M+H]^+^ 231.1088, found 231.1069. FTIR (ATR) cm^-1^: 3018, 2954, 2870, 1612, 1592, 1548, 1457, 1393, 1292, 1245, 1201, 882, 817, 770, 752, 723.

(**carbo-6**) Following *General procedure C* but applied to the reaction between 1-iodo-8-methylnaphthalene (107 mg, 0.4 mmol) and (*E*)-1-(2-bromophenyl)-*N*-(trimethylsilyl)methanimine (**3**-Br) (154 mg, 0.6 mmol). The product was purified by automated flash chromatography (cyclohexane containing 1% of Et_3_N) affording 20 mg of **carbo-6** (0.082 mmol, 21% yield) as a white solid.

^1^H NMR (400 MHz, CDCl_3_) δ 9.51 (s, 1H**k**), 8.73 (d, *J* = 8.4 Hz, 1H**d**), 8.59 (d, *J* = 8.9 Hz, 1H**e**), 8.14 (ddd, *J* = 8.0, 1.5, 0.7 Hz, 1H**j**), 8.04 (d, *J* = 8.9 Hz, 1H**f**), 7.93 – 7.85 (m, 2H**c,h**), 7.74 (ddd, *J* = 8.0, 6.9, 1.0 Hz, 1H**i**), 7.62 – 7.53 (m, 2H**b,g**), 3.43 (s, 3H**a**). ^13^C NMR (100.5 MHz, CDCl_3_) δ 149.7, 144.0, 138.6, 134.9, 132.7, 131.3, 130.9, 130.4, 129.1, 128.2, 127.1, 126.8, 126.8, 125.9, 122.5, 122.1, 119.9, 27.8. GC-HRMS (orbitrap) *m*/*z* calcd. for C_18_H_13_N: [M]^+•^ 243.1043, found 243.1043. FTIR (ATR) cm^-1^: 3022, 2957, 2918, 1618, 1580, 1517, 1428, 1376, 1259, 891, 816, 765, 753, 682.

1-iodo-8-methylnaphthalene was synthesized following the same procedure as for 1-iodo-9-methyl-[1,2]azaborinino[1,2-*a*][1,2]azaborinine (**5**) described in Section 2.4. ^1^H NMR (400 MHz, CDCl_3_) δ 8.29 (dd, *J* = 7.4, 1.3 Hz, 1H), 7.80 (dd, *J* = 8.1, 1.3 Hz, 1H), 7.71 (ddd, *J* = 7.8, 1.1, 0.6 Hz, 1H), 7.40 – 7.31 (m, 2H), 7.02 (dd, *J* = 8.1, 7.3 Hz, 1H), 3.20 (s, 3H). Data in agreement with literature values.^[6]^ 1-Bromo-8-methylnaphthalene was synthesized as described in ref. 5. ^1^H NMR (400 MHz, CDCl_3_) δ 7.82 (dd, *J* = 7.4, 1.3 Hz, 1H), 7.77 (ddd, *J* = 8.1, 1.3, 0.5 Hz, 1H), 7.73 – 7.67 (m, 1H), 7.37 – 7.31 (m, 2H), 7.21 (dd, *J* = 8.1, 7.4 Hz, 1H), 3.13 (s, 3H). Data in agreement with literature values.^[5]^

Preparation of benzo[*c*]phenanthridine species

To a 25 mL Schlenk flask containing a magnetic stir bar, palladium acetate (4.5 mg, 10 mol%), triphenylphosphine (13 mg, 25 mol%), norbornene (NBE, 151 mg, 1.0 mmol, 8.0 equiv.) and cesium carbonate (195 mg, 0.6 mmol, 3.0 equiv.) were added. Then, acetonitrile (3 mL) was added and the mixture was left stirring for 10 minutes. A solution of (*E*)-1-(2-bromophenyl)-*N*-(trimethylsilyl)methanimine (**3**-Br) (77 mg, 0.3 mmol, 1.5 equiv.) in 0.8 mL of acetonitrile and 1-iodonaphthalene (51 mg, 29 µL, 0.2 mmol, 1.0 equiv.) were subsequently added. Extra 0.2 mL of dimethoxyethane were used to rinse the Schlenk walls and the resulting mixture was stirred first at room temperature for 5 minutes and then at 90 °C in a pre-heated oil-bath for 16 hours. The mixture was then allowed to reach room temperature and filtered through a pad of Celite^®^, and the pad was further washed with dichloromethane (3 x 15 mL). Organic solvent from the filtrate was removed by rotary evaporation, thus providing a residue which was subsequently purified by automated flash chromatography (4 g cartridge, 95:5, cyclohexane:ethyl acetate – cyclohexane containing 1% of Et_3_N) affording 36 mg of **carbo-4** (0.157 mmol, 78% yield) as a yellow solid.

(**carbo-4**) ^1^H NMR (400 MHz, CDCl_3_) δ 9.49 (s, 1H), 9.42 (d, *J* = 8.3 Hz, 1H), 8.69 (d, *J* = 8.2 Hz, 1H), 8.56 (d, *J* = 9.0 Hz, 1H), 8.15 (d, *J* = 7.9 Hz, 1H), 8.04 (d, *J* = 9.0 Hz, 1H), 7.99 (d, *J* = 8.6 Hz, 1H), 7.90 (ddd, *J* = 8.4, 7.0, 1.3 Hz, 1H), 7.81 – 7.75 (m, 1H), 7.74 – 7.67 (m, 2H). ^13^C NMR (100.5 MHz, CDCl_3_) δ 152.2, 141.7, 133.4, 133.0, 132.2, 131.0, 128.8, 128.00, 127.8, 127.5, 127.3, 127.2, 127.1, 124.9, 122.4, 121.2, 120.1. Data in agreement with literature values.^[3]^

# 6. Synthesis of BN*-*phenanthridines using aryl silyl ketimines. General procedure D

To a 25 mL Schlenk flask containing a magnetic stir bar, palladium acetate (4.5 mg, 10 mol%), tri-(2-furyl)phosphine (10 mg, 22 mol%), norbornene (NBE, 94 mg, 1.0 mmol, 5.0 equiv.) -or norbornadiene (NBDE, 102 µL, 1.0 mmol, 5.0 equiv.) and cesium carbonate (195 mg, 0.6 mmol, 3.0 equiv.) were added. Then, dimethoxyethane (3 mL) was added and the mixture was left stirring for 10 minutes. Then, a solution of the corresponding chloro aryl silyl ketimine (0.3 mmol, 1.5 equiv.) in 0.8 mL of dimethoxyethane and BN-iodoarene (0.2 mmol, 1.0 equiv.) were subsequently added. Extra 0.2 mL of dimethoxyethane were used to rinse the Schlenk walls and the resulting mixture was stirred first at room temperature for 5 minutes and then at 90 °C in a pre-heated oil-bath for 16 hours. The mixture was then allowed to reach room temperature and filtered through a pad of Celite^®^, and the pad was further washed with dichloromethane (3x15 mL). Concentration of the filtrate by rotary evaporation provided a residue which was subsequently purified by automated flash chromatography (4 g cartridge) and, if necessary, recrystallized.

(**18**) Following *General procedure D*. Reaction between 1-iodo-9-methyl-[1,2]azaborinino[1,2-*a*][1,2]azaborinine (**5**) (54 mg, 0.2 mmol) and the mixture of (*E*)-1-(2-chlorophenyl)-*N*-(trimethylsilyl)ethan-1-imine with *N*-(1-(2-chlorophenyl)vinyl)-1,1,1-trimethylsilanamine (**17-Me**) (68 mg, 0.3 mmol) with norbornene. The product was purified by automated flash chromatography (cyclohexane containing 1% of Et_3_N) affording 45 mg (0.174 mmol, 87% yield) of **18** as a cream colored solid.

^1^H NMR (400 MHz, CDCl_3_) δ 8.54 (d, *J* = 8.3 Hz, 1H**g**), 8.27 (d, *J* = 8.2 Hz, 1H**j**), 7.89 (d, *J* = 7.5 Hz, 1H**e**), 7.88 – 7.79 (m, 1H**h**), 7.78 – 7.71 (m, 2H**d,i**), 7.70 (d, *J* = 7.5 Hz, 1H**f**), 7.60 (d, *J* = 6.7 Hz, 1H**b**), 6.70 (t, *J* = 6.7 Hz, 1H**c**), 3.15 (s, 3H**k**), 3.13 (s, 3H**a**). ^13^C NMR (100.5 MHz, CDCl_3_) δ 155.9, 139.0, 132.7, 132.0, 131.4, 129.6, 128.8, 127.6, 126.5, 126.3, 122.8, 113.5, 107.8, 24.4, 23.8 (signals for quaternary carbons next to boron atom are not observed). ^11^B NMR (128 MHz, DMSO-*d*_6_) δ 26.5. GC-HRMS (orbitrap) m/z calcd. for C_17_H_15_BN_2_: [M]^+•^ 258.1323, found 258.1322. FTIR (ATR) cm^-1^: 3063, 2929, 2892, 2826, 1634, 1609, 1576, 1534, 1511, 1379, 1360, 1325, 1248, 830, 801, 770, 751, 726.

(**19**) Following *General procedure D*. Reaction between 1-iodo-9-methyl-[1,2]azaborinino[1,2-*a*][1,2]azaborinine (**5**) (54 mg, 0.2 mmol) and (*E*)-1-(2-chlorophenyl)-*N*-(trimethylsilyl)pentan-1-imine (**17-Bu**) (80 mg, 0.3 mmol) with norbornene. The product was purified by automated flash chromatography (cyclohexane containing 1% of Et_3_N) affording 43 mg (0.143 mmol, 71% yield) of **19** as a yellow solid.

^1^H NMR (400 MHz, CDCl_3_) δ 8.56 (d, *J* = 8.3 Hz, 1H**g**), 8.33 (d, *J* = 9.1 Hz, 1H**j**), 7.90 (d, *J* = 7.5 Hz, 1H**e**), 7.82 (ddd, *J* = 8.3, 6.9, 1.3 Hz, 1H**g**), 7.77 – 7.69 (m, 3H**d,f,i**), 7.59 (dt, *J* = 6.7, 1.3 Hz, 1H**b**), 6.70 (t, *J* = 6.8 Hz, 1H**c**), 3.54 – 3.45 (m, 2H), 3.13 (s, 3H**a**), 2.11 (p, *J* = 7.5 Hz, 2H), 1.64 – 1.50 (m, 2H), 1.04 (t, *J* = 7.4 Hz, 3H). ^13^C NMR (100.5 MHz, CDCl_3_) δ 158.9, 138.9, 132.6, 132.0, 131.6, 129.4, 128.6, 127.5, 126.1, 125.8, 122.9, 113.5, 107.8, 35.5, 30.8, 24.8, 23.0, 14.3 (signals for quaternary carbons next to boron atom are not observed). ^11^B NMR (128 MHz, CDCl_3_) δ 29.3. GC-HRMS (orbitrap) *m*/*z* calcd. for C_20_H_21_BN_2_: [M]^+•^ 300.1792, found 300.1794. FTIR (ATR) cm^-1^: 2952, 2934, 2868, 2846, 1635, 1606, 1535, 1507, 1307, 1252, 1204, 1186, 1031, 952, 898, 840, 814, 780, 757, 731, 686, 652.

(**20**) Following *General procedure D*. Reaction between 1-iodo-9-methyl-[1,2]azaborinino[1,2-*a*][1,2]azaborinine (**5**) (54 mg, 0.2 mmol) and (*E*)-1-(2-chlorophenyl)-1-phenyl-*N*-(trimethylsilyl)methanimine (**17-Ph**) (86 mg, 0.3 mmol) with norbornene. The product was purified by automated flash chromatography (cyclohexane containing 1% of Et_3_N) affording 42 mg (0.131 mmol, 66% yield) of **20** as a cream colored solid.

^1^H NMR (400 MHz, CDCl_3_) δ 8.63 (d, *J* = 8.4 Hz, 1H**g**), 8.35 (d, *J* = 8.3 Hz, 1H**j**), 8.00 – 7.93 (m, 3H**e,2k**), 7.87 (t, *J* = 7.7 Hz, 1H**h**), 7.81 – 7.72 (m, 2H**d,f**), 7.75 – 7.65 (m, 1H**i**), 7.63 – 7.55 (m, 3H**b,2l**), 7.56 – 7.50 (m, 1H**m**), 6.72 (t, *J* = 7.0 Hz, 1H**c**), 3.08 (s, 3H**a**). ^13^C NMR (100.5 MHz, CDCl_3_) δ 157.8, 139.2, 133.4, 132.4, 132.0, 132.0, 130.8, 129.8, 128.8, 128.4, 128.3, 128.3, 127.6, 125.5, 122.7, 113.7, 107.7, 24.6 (signals for quaternary carbons next to boron atom are not observed).^11^B NMR (128 MHz, CDCl_3_) δ 29.3. HRMS (ESI) *m*/*z* calcd. for C_22_H_18_BN_2_: [M+H]^+^ 321.1558, found 321.1551. FTIR (ATR) cm^-1^: 3059, 2934, 1649, 1606, 1526, 1444, 1375, 1324, 1245, 1031, 954, 844, 821, 771, 733, 694, 666.

6.1 Reaction between 2 and 17-Me in the presence of norbornene (NBE)

Following *General procedure D*. Reaction between 1-iodo-[1,2]azaborinino[1,2-*a*][1,2]azaborinine (**2**) (51 mg, 0.2 mmol) and the mixture of (*E*)-1-(2-chlorophenyl)-*N*-(trimethylsilyl)ethan-1-imine with *N*-(1-(2-chlorophenyl)vinyl)-1,1,1-trimethylsilanamine (**17-Me**) (68 mg, 0.3 mmol) with norbornene produced a mixture, predominantly containing species **21** and **22-b** (NMR ratio: 41% *vs* 59%, respectively). The main product **22-b** was purified by automated flash chromatography (95:5, pentane:ethyl acetate) affording 22 mg (0.064 mmol, 32% yield) as light-yellow solid.

(**22-b**)^1^H NMR (600 MHz, CDCl_3_) δ 7.61 (d, *J* = 11.7 Hz, 1H**a**), 7.53 (d, *J* = 6.9 Hz, 1H**d**), 7.49 (ddd, *J* = 11.8, 6.4, 1.3 Hz, 1H**b**), 7.36 (d, *J* = 7.2 Hz, 1H**e**), 7.07 (d, *J* = 7.8 Hz, 1H**h**), 7.00 (td, *J* = 7.6, 1.5 Hz, 1H**i**), 6.94 (t, *J* = 7.0, 1H**j**), 6.87 (dd, *J* = 7.4, 1.5 Hz, 1H**k**), 6.47 (td, *J* = 6.6, 1.3 Hz, 1H**c**), 6.04 (d, *J* = 7.1 Hz, 1H**f**), 3.37 (s, 1H**n**), 3.09 (d, *J* = 10.0 Hz, 1H**m**), 2.96 (d, *J* = 9.9 Hz, 1H**l**), 2.76 (s, 1H**q**), 2.50 (s, 3H**g**), 2.24 (d, *J* = 9.6 Hz, 1H**r**), 1.81 (dd, *J* = 9.6, 2.2 Hz, 2H**o**,**p**), 1.64 (dt, *J* = 9.5, 1.8 Hz, 1H**r**), 1.46 (dd, *J* = 7.5, 2.2 Hz, 2H**o**,**p**). ^13^C NMR (100.5 MHz, CDCl_3_) δ 172.7, 158.2, 141.8, 139.8, 137.1, 135.0, 133.3, 128.4, 125.1, 124.5, 123.0, 112.4, 109.7, 55.9, 55.4, 42.3, 40.0, 38.0, 31.1, 30.5, 27.5 (signals for two carbons next to boron atom are not observed). ^11^B NMR (193 MHz, CDCl_3_) δ 27.0. HRMS (ESI) *m*/*z* calcd. for C_23_H_24_BN_2_: [M+H]^+^ 339.2027, found 339.2026. FTIR (ATR) cm^-1^: 2955 (C-H *st*), 2921 (C-H *st*), 2868, 1630, 1606 (C=C *st*), 1517, 1420 (H-C-H δ), 1401, 1279, 1201, 923, 810, 743, 670.

# 7. [2+2+4]-BN*-Arex* reaction between 2 and 17-Me or 17-Bu and norbornadiene (NBDE)

Following *General procedure D*, reaction between 1-iodo-[1,2]azaborinino[1,2-*a*][1,2]azaborinine (**2**) (51 mg, 0.2 mmol) and (*E*)-1-(2-chlorophenyl)-*N*-(trimethylsilyl)ethan-1-imine (**17-Me**) (68 mg, 0.3 mmol) with norbornadiene. The product was purified by automated flash chromatography (90:10, pentane:ethyl acetate) affording 37 mg (0.110 mmol, 55% yield) of **23** as light-yellow solid.

(**23**) ^1^H NMR (400 MHz, CDCl_3_) δ 7.54 (d, *J* = 7.1 Hz, 1H**d**), 7.50 (dd, *J* = 4.0, 1.0 Hz, 2H**a,b**), 7.38 (d, *J* = 7.1 Hz, 1H**e**), 7.05 – 7.02 (m, 2H**j,k**), 7.02 – 6.93 (m, 1H**i**), 6.90 (dt, *J* = 7.3, 1.1 Hz, 1H**h**), 6.48 (dt, *J* = 6.9, 3.9 Hz, 1H**c**), 6.44 – 6.34 (m, 2H**o,p**), 6.06 (d, *J* = 7.1 Hz, 1H**f**), 3.92 (s, 1H**n**), 3.28 (s, 1H**q**), 3.00 (dd, *J* = 9.6, 2.1 Hz, 1H**m**), 2.88 (dd, *J* = 9.6, 1.8 Hz, 1H**l**), 2.47 (s, 3H**g**), 2.30 (d, *J* = 8.3 Hz, 1H**r**), 1.91 (dt, *J* = 8.2, 1.9 Hz, 1H**r**). ^13^C NMR (100.5 MHz, CDCl_3_) δ 172.5, 158.6, 142.1, 140.2, 139.8, 138.6, 137.4, 135.1, 133.5, 128.6, 125.2, 124.5, 123.0, 112.5, 109.7, 109.6, 50.1, 47.6, 47.3, 45.1, 27.4 (signals for two carbons next to boron atom are not observed). ^11^B NMR (128 MHz, CDCl_3_) δ 27.9. HRMS (ESI) *m*/*z* calcd. for C_23_H_22_BN_2_: [M+H]^+^ 337.1871, found 337.1871. FTIR (ATR) cm^-1^: 3047, 2923, 1561, 1502, 1381, 1084, 837, 819, 801, 768, 738.

(**24**) Following *General procedure D*. Reaction between 1-iodo-[1,2]azaborinino[1,2-*a*][1,2]azaborinine (**2**) (51 mg, 0.2 mmol) and (*E*)-1-(2-chlorophenyl)-*N*-(trimethylsilyl)pentan-1-imine (**17-Bu**) (80 mg, 0.3 mmol) with norbornadiene. The product was purified by automated flash chromatography (90:10, pentane:ethyl acetate) affording 35 mg (0.092 mmol, 46% yield) of **24** as light-yellow solid.

^1^H NMR (400 MHz, CDCl_3_) δ 7.53 (d, *J* = 6.9 Hz, 1H**d**), 7.49 (d, *J* = 3.9 Hz, 2H**a,b**), 7.37 (d, *J* = 7.2 Hz, 1H**e**), 7.05 – 7.01 (m, 2H**m,n**), 6.97 (dt, *J* = 8.6, 4.2 Hz, 1H**l**), 6.88 (d, *J* = 7.3 Hz, 1H**k**), 6.47 (dt, *J* = 7.3, 3.9 Hz, 1H**c**), 6.43 – 6.34 (m, 2H**r,s**), 6.04 (d, *J* = 7.2 Hz, 1H**f**), 3.92 (s, 1H**q**), 3.27 (s, 1H**t**), 2.98 (dd, *J* = 9.6, 2.1 Hz, 1H**p**), 2.87 (dd, *J* = 9.6, 1.8 Hz, 1H**o**), 2.81 – 2.57 (m, 2H**g**), 2.30 (d, *J* = 8.2 Hz, 1H**u**), 1.90 (dt, *J* = 8.3, 1.9 Hz, 1H**u**), 1.86 – 1.70 (m, 2H**h**), 1.50 (h, *J* = 7.4 Hz, 2H**i**), 0.99 (t, *J* = 7.4 Hz, 3H**j**). ^13^C NMR (100.5 MHz, CDCl_3_) δ 175.3, 158.9, 142.5, 140.1, 139.8, 138.6, 137.4, 135.1, 133.4 (broad signal, **C**-H adjacent to boron atom), 132.3, 128.5, 125.1, 124.5, 123.3 112.4, 109.7, 50.1, 50.1, 47.6, 47.3, 45.1 40.1, 27.8, 22.8, 14.1 (signal for the quaternary carbon next to boron atom is not observed). ^11^B NMR (193 MHz, CDCl_3_) δ 27.2. HRMS (ESI) *m*/*z* calcd. for C_26_H_28_BN_2_: [M+H]^+^ 379.2340, found 379.2339.

# 8. Synthesis of BN-dibenzoazepine derivative (25)

An oven dried Schlenk tube equipped with a magnetic stir bar was purged with argon, and then charged with palladium acetate (4.5 mg, 10 mol%), tri-(2-furyl)phosphine (10 mg, 22 mol%), cesium carbonate (195 mg, 0.6 mmol, 3 equiv.), norbornadiene (55 mg, 61 μL 0.6 mmol, 3 equiv.). Then, anhydrous dimethylformamide (3.5 mL) was added and the mixture was left stirring for 10 minutes at room temperature. Then, *o*-bromoaniline (52 mg, 34 μL, 0.3 mmol, 1.5 equiv.) and 1-iodo-[1,2]azaborinino[1,2-*a*][1,2]azaborinine (**2**) (51 mg, 0.2 mmol, 1.0 equiv.) were subsequently added. Additional 0.5 mL of dimethylformamide was added to rinse the walls of the Schlenk flask. The resulting mixture was stirred first at room temperature for 5 minutes and then heated at 130 °C in a pre-heated oil-bath for 24 hours. The mixture was then allowed to reach room temperature and filtered through a pad of Celite^®^, and the pad was further washed with dichloromethane (3x15 mL). Concentration of the filtrate by rotary evaporation provided a residue which was purified by automated flash chromatography (4 g cartridge, 95:5, cyclohexane:ethyl acetate) affording 27 mg (0.111 mmol, 56% yield) of **25** as a red-brown solid.

 ^1^H NMR (400 MHz, CDCl_3_) δ 7.58 – 7.48 (m, 3H**b,d,e**), 7.30 (d, *J* = 10.8 Hz, 1H**a**), 6.96 (ddd, *J* = 7.8, 6.5, 2.5 Hz, 1H**i**), 6.84 – 6.75 (m, 3H**m,k,j**), 6.53 (td, *J* = 6.6, 1.4 Hz, 1H**c**), 6.34 (d, *J* = 8.8 Hz, 1H**h**), 6.24 (d, *J* = 11.5 Hz, 1H**l**), 5.91 (d, *J* = 7.2 Hz, 1H**f**), 4.83 (broad singlet, 1H**g**). ^13^C NMR (100.5 MHz, CDCl_3_) δ 154.9, 148.3, 138.3, 135.8, 134.2, 133.1, 131.9, 130.8, 130.2, 129.2, 123.7, 119.4, 113.5, 111.0 (signals for two carbons next to boron atom are not observed). ^11^B NMR (128 MHz, CDCl_3_) δ 29.1. GC-HRMS (orbitrap) *m*/*z* calcd. for C_16_H_13_BN_2_: [M]^+•^ 244.1166, found 244.1166. FTIR (ATR) cm^-1^: 3363 (N-H *st*), 2914, 1614, 1600, 1550, 1485, 1466, 1415, 1298, 1262, 1234, 803, 784, 732, 695, 659.

# 9. Synthesis of pleiadiene 1,2-azaborine (26)

An oven dried Schlenk tube equipped with a magnetic stir bar was purged with argon, and then charged with palladium acetate (2.3 mg, 5 mol%), tricyclohexylphosphine (8.4 mg, 15 mol%), 8-bromo-1-naphthoic acid (100 mg, 0.4 mmol, 2 equiv.), 1-iodo-[1,2]azaborinino[1,2-*a*][1,2]azaborinine (**2**) (51 mg, 0.2 mmol, 1 equiv.) and cesium carbonate (195 mg, 0.6 mmol, 3 equiv.). Then, anhydrous 1,4-dioxane (2.0 mL, 0.1 M,) and norbornadiene (51 μL, 0.5 mmol, 2.5 equiv.) were subsequently added. The resulting mixture was placed into a preheated oil bath at 130 °C and stirred at this temperature for 18 hours. After that time, the mixture was allowed to reach room temperature and water was added. The organic layer was extracted with ethylacetate (3x10 mL), the combined organic phase was dried over anhydrous magnesium sulphate, filtered and evaporated under reduced pressure. The resulting residue was purified by automated flash chromatography (4 g cartridge, 95:5, cyclohexane:ethyl acetate) affording 34 mg (0.122 mmol, 61% yield) of **26** as a red-brown solid.

^1^H NMR (400 MHz, CDCl_3_) δ 7.79 – 7.71 (m, 3H**b,d,e**), 7.61 (d, *J* = 10.9 Hz, 1H**a**), 7.57 (dd, *J* = 7.4, 1.9 Hz, 1H**i**), 7.49 (dd, *J* = 8.1, 1.3 Hz, 1H**j**), 7.45 – 7.37 (m, 2H**g,h**), 7.30 – 7.18 (m, 2H **k,n**), 7.09 (dt, *J* = 7.2, 1.2 Hz, 1H**l**), 6.74 (td, *J* = 6.6, 1.4 Hz, 1H**c**), 6.64 (d, *J* = 7.4 Hz, 1H**f**), 6.49 (d, *J* = 11.8 Hz, 1H**m**). ^13^C NMR (100.5 MHz, CDCl_3_) δ 146.9, 140.8, 140.3, 139.3, 137.3, 135.9, 135.8, 134.9, 133.3, 131.3, 128.8, 128.2, 128.1, 126.6, 126.4, 125.9, 119.5, 114.6 (signals for two carbons next to boron atom are not observed). ^11^B NMR (128 MHz, CDCl_3_) δ 29.0. GC-HRMS (orbitrap) *m*/*z* calcd. for C_20_H_14_BN: [M]^+•^ 279.1214, found 279.1216. FTIR (ATR) cm^-1^: 3049, 2921, 2851, 1675, 1623, 1597, 1511, 1442, 1414, 1398, 1260, 834, 806, 785, 767, 730, 670.

# 10. Study of the [3+2]-BN*-Arex* reaction with bromo (hetero)aryl aldehydes

General screening conditions:

To a 25 mL Schlenk flask containing a magnetic stir bar, palladium acetate (4.5 mg, 10 mol%), the corresponding phosphine (22 mol%), norbornene (1-8 equiv. depending on the case) and cesium carbonate (195 mg, 0.6 mmol, 3.0 equiv.) were added. Next, the corresponding solvent (4 mL, 0.05 M) was added, and the suspension was degassed by bubbling argon for 10 minutes. Then, bromobenzaldehyde (55 mg, 35 μL, 0.3 mmol, 1.5 equiv.) and 1-iodo-[1,2]azaborinino[1,2-*a*][1,2]azaborinine (**2**) (51 mg, 0.2 mmol, 1.0 equiv.) were added, and the resulting mixture was stirred at room temperature for 5 minutes. The reaction mixture was placed in a pre-heated oil-bath at 90 °C for 12 hours. The mixture was then allowed to reach room temperature and filtered through a pad of Celite^®^, and the pad was further washed with dichloromethane (3 x 15 mL). Concentration of the filtrate by rotary evaporation provided a residue from which compound **27** was purified by automated flash chromatography (4 g cartridge, 90:10, cyclohexane:ethyl acetate).

# 11. Synthesis of BN-containing fluorenones. General procedure E

To a 25 mL Schlenk flask containing a magnetic stir bar, palladium acetate (4.5 mg, 10 mol%), tri-(2-furyl)phosphine (10 mg, 22 mol%), norbornene (94 mg, 1.0 mmol, 5.0 equiv.) and cesium carbonate (195 mg, 0.6 mmol, 3.0 equiv.) were added. Then, dimethoxyethane (4 mL, 0.05 M) was added and the suspension was degassed by bubbling argon for 10 minutes. Then, bromo (hetero) aryl aldehyde (0.3 mmol, 1.5 equiv.) and the BN-iodoarene (0.2 mmol, 1.0 equiv.) were added. The resulting mixture was stirred at room temperature for 5 minutes. The reaction mixture was placed in a pre-heated oil-bath at 90 °C for 12 hours. Next, the mixture was allowed to reach room temperature and filtered through a pad of Celite^®^, and the pad was further washed with dichloromethane (3 x 15 mL). Concentration of the filtrate by rotary evaporation provided a residue which was purified by automated flash chromatography (4 g cartridge) and/or, if necessary, recrystallized.

(**27**) Following the *General procedure E* (albeit at double scale). Reaction between 1-iodo-[1,2]azaborinino[1,2-*a*][1,2]azaborinine (**2**) (102 mg, 0.4 mmol) and 2-bromo benzaldehyde (110 mg, 70 μL, 0.6 mmol). The product was purified by automated flash chromatography (90:10, cyclohexane:ethyl acetate) affording 40 mg (0.172 mmol, 43% yield) of **27** as a yellow solid.

^1^H NMR (400 MHz, CDCl_3_) δ 8.05 (d, *J* = 6.9 Hz, 1H**e**), 7.91 (d, *J* = 11.3 Hz, 1H**a**), 7.73 (d, *J* = 7.2 Hz, 1H**d**), 7.71 – 7.66 (m, 1H**b**), 7.63 (dt, *J* = 7.2, 1.0 Hz, 1H**j**), 7.48 – 7.40 (m, 2H**h,g**), 7.37 – 7.29 (m, 1H**i**), 7.06 (dd, *J* = 6.8, 1.0 Hz, 1H**f**), 6.73 (td, *J* = 7.1, 1.5 Hz, 1H**c**) ^13^C NMR (100.5 MHz, CDCl_3_) δ 199.9, 159.7, 144.0, 142.0, 139.6, 136.1, 133.6 133.3, 130.1, 123.5, 120.0, 115.9, 106.9 (signals for two carbons next to boron atom are not observed).^11^B NMR (128 MHz, CDCl_3_) δ 24.2. GC-HRMS (orbitrap) *m*/*z* calcd. for C_15_H_10_BNO: [M]^+•^ 231.0850, found 231.0850. FTIR (ATR) cm^-1^: 2928, 1688 (C=O *st*), 1603, 1588, 1525, 1341, 1165, 1094, 812, 767, 719.

(**28**) Following the *General procedure E* (albeit at double scale). Reaction between 1-iodo-9-methyl-[1,2]azaborinino[1,2-*a*][1,2]azaborinine (**5**) (108 mg, 0.4 mmol) and 2-bromobenzaldehyde (110 mg, 70 μL, 0.6 mmol). The product was purified by automated flash chromatography (90:10, cyclohexane:ethyl acetate) affording 70 mg (0.284 mmol, 71% yield) of **28** as a light brown solid.

^1^H NMR (400 MHz, CDCl_3_) δ 8.05 (d, *J* = 6.8 Hz, 1H**e**), 7.62 (d, *J* = 7.5 Hz, 1H**j**), 7.56 (d, *J* = 7.1 Hz, 1H**d**), 7.47 – 7.41 (m, 2H**g,h**), 7.39 (dt, *J* = 6.7, 1.3 Hz, 1H**b**), 7.38 – 7.29 (m, 1H**i**), 7.04 (d, *J* = 6.8 Hz, 1H**f**), 6.59 (t, *J* = 6.8 Hz, 1H**c**), 2.82 (s, 3H**a**). ^13^C NMR (100.5 MHz, CDCl_3_) δ 199.2, 160.2, 143.6, 142.8, 137.8, 135.6, 133.3, 131.7, 130.1, 123.4, 119.6, 115.2, 106.5, 25.1 (signals for quaternary carbons next to boron atom are not observed). ^11^B NMR (128 MHz, CDCl_3_) δ 28.8. GC-HRMS (orbitrap) *m*/*z* calcd. for C_16_H_12_BNO: [M]^+•^ 245.1006, found 245.1007. FTIR (ATR) cm^-1^: 2948, 1692 (C=O *st*), 1604, 1587, 1534, 1510, 810, 768, 771.

(**29**) Following the *General procedure E*. Reaction between 1-iodo-9-methyl-[1,2]azaborinino[1,2-*a*][1,2]azaborinine (**5**) (54 mg, 0.2 mmol) and 2-bromo-5-(trifluoromethyl)benzaldehyde (76 mg, 50 μL, 0.3 mmol). The product was purified by recrystallization in cyclohexane and ethyl acetate drops affording 31 mg (0.100 mmol, 50% yield) of **29** as a yellow solid.

^1^H NMR (400 MHz, CDCl_3_) δ 8.14 (d, *J* = 6.9 Hz, 1H**e**), 7.85 (s, 1H**i**), 7.74 (ddd, *J* = 7.6, 1.7, 0.9 Hz, 1H**h**), 7.61 (d, *J* = 7.0 Hz, 1H**d**), 7.58 (d, *J* = 7.7 Hz, 1H**g**), 7.44 (d, *J* = 6.8 Hz, 1H**b**), 7.10 (d, *J* = 6.8 Hz, 1H**f**), 6.66 (t, *J* = 6.9 Hz, 1H**c**), 2.83 (d, *J* = 1.2 Hz, 3H**a**). ^13^C NMR (151 MHz, CDCl_3_) δ 197.2, 158.6, 146.7, 143.3, 138.3, 136.1, 132.2 (q, ^2^*J_C-F_* = 32.7 Hz, CF_3_-**C**), 131.7, 130.5 (q, ^3^*J_C-F_* = 3.9 Hz, C-**h**), 124.0 (q, ^1^*J_C-F_* = 272.1 Hz, **C**-F_3_), 120.3 (q, ^3^*J_C-F_* = 3.8 Hz, C-**i**), 119.6, 115.7, 106.5, 25.0 (signals for quaternary carbons next to boron atom are not observed).^11^B NMR (193 MHz, CDCl_3_) δ 28.5. ^19^F NMR (376 MHz, CDCl_3_) δ -62.7. GC-HRMS (orbitrap) *m*/*z* calcd. for C_17_H_11_BF_3_NO: [M]^+•^ 313.0880, found 313.0877. FTIR (ATR) cm^-1^: 2954, 1693 (C=O *st*), 1632, 1614, 1586, 1534, 1511, 1382, 1315, 1265, 1173, 1156, 1116 (C-F *st*), 1052, 894, 837, 815, 782, 759.

(**30**) Following the *General procedure E*. Reaction between 1-iodo-9-methyl-[1,2]azaborinino[1,2-*a*][1,2]azaborinine (**5**) (54 mg, 0.2 mmol) and 2-bromoterephthaldehyde (64 mg, 0.3 mmol). The product was purified by automated flash chromatography (80:20, cyclohexane:ethyl acetate) affording 36 mg (0.132 mmol, 66% yield) of **30** as a light brown solid.

^1^H NMR (400 MHz, CDCl_3_) δ 10.09 (s, 1H**h**), 8.16 (d, *J* = 6.8 Hz, 1H**e**), 7.99 (s, 1H**g**), 7.87 (dd, *J* = 7.4, 1.3 Hz, 1H**i**), 7.78 (d, *J* = 7.3 Hz, 1H**j**), 7.61 (d, *J* = 7.0 Hz, 1H**d**), 7.44 (d, *J* = 6.6 Hz, 1H**b**), 7.15 (d, *J* = 6.8 Hz, 1H**f**), 6.65 (t, *J* = 6.8 Hz, 1H**c**), 2.83 (s, 3H**a**). ^13^C NMR (100.5 MHz, CDCl_3_) δ 191.8, 182.5, 159.1, 144.3, 143.6, 140.3, 140.3, 138.3, 134.3, 131.7, 123.6, 118.5, 115.6, 106.5, 25.0 (signals for quaternary carbons next to boron atom are not observed).^11^B NMR (128 MHz, CDCl_3_) δ 29.6. GC-HRMS (orbitrap) *m*/*z* calcd. for C_17_H_12_BNO_2_: [M]^+•^ 273.0956, found 273.0956. FTIR (ATR) cm^-1^: 2921, 1690 (C=O *st*), 1630, 1588, 1536, 1460, 1430, 1403, 1376, 1158, 811, 785, 761, 710.

(**31**) Following the *General procedure E*. Reaction between 1-iodo-9-methyl-[1,2]azaborinino[1,2-*a*][1,2]azaborinine (**5**) (54 mg, 0.2 mmol) and 6-bromo-1,3-benzodioxole-5-carboxaldehyde (69 mg, 0.3 mmol). The product was purified by automated flash chromatography (80:20, cyclohexane:ethyl acetate) affording 39 mg (0.135 mmol, 68% yield) of **31** as an orange-red solid.

^1^H NMR (400 MHz, DMSO-*d*_6_) δ 8.55 (d, *J* = 7.0 Hz, 1H**e**), 7.95 (d, *J* = 7.2 Hz, 1H**d**), 7.46 (s, 1H**g**), 7.38 – 7.30 (m, 2H**b,f**), 7.06 (s, 1H**i**), 6.66 (t, *J* = 6.8 Hz, 1H**c**), 6.15 (s, 2H**h**), 2.67 (s, 3H**a**). ^13^C NMR (100.5 MHz, DMSO-*d*_6_) δ 196.9, 159.6, 151.9, 148.9, 144.2, 139.4, 137.7, 132.5, 129.3, 114.4, 106.7, 104.1, 102.4, 102.4, 24.6 (signals for quaternary carbons next to boron atom are not observed). ^11^B NMR (128 MHz, CDCl_3_) δ 28.2. GC-HRMS (orbitrap) *m*/*z* calcd. for C_17_H_12_BNO_3_: [M]^+•^ 289.0905, found 289.0904. FTIR (ATR) cm^-1^: 2919, 1677 (C=O *st*), 1587, 1471, 1457, 1327, 1241, 1028 (C-O-C *st* as), 822, 786, 672.

(**32**) Following the *General procedure E*. Reaction between 1-iodo-9-methyl-[1,2]azaborinino[1,2-*a*][1,2]azaborinine (**5**) (54 mg, 0.2 mmol) and 3-bromothiophene-2-carbaldehyde (57 mg, 33 μL, 0.3 mmol). The product was purified by automated flash chromatography (95:5, cyclohexane:ethyl acetate) affording 12 mg (0.046 mmol, 23% yield) of **32** as an orange-brown solid.

^1^H NMR (400 MHz, CDCl_3_) δ 8.00 (d, *J* = 6.7 Hz, 1He), 7.63 (d, *J* = 4.6 Hz, 1H**h**), 7.52 (d, *J* = 7.0 Hz, 1H**d**), 7.33 (dt, *J* = 6.6, 1.3 Hz, 1H**b**), 7.06 (d, *J* = 4.6 Hz, 1H**g**), 6.80 (d, *J* = 6.7 Hz, 1H**f**), 6.57 (t, *J* = 6.8 Hz, 1H**c**), 2.75 (s, 3H**a**). ^13^C NMR (100.5 MHz, CDCl_3_) δ 191.1, 156.7, 155.3, 142.7, 139.7, 137.2, 136.8, 131.5, 119.4, 115.2, 107.0, 25.2 (signals for quaternary carbons next to boron atom are not observed). ^11^B NMR (128 MHz, CDCl_3_) δ 28.1. GC-HRMS (orbitrap) *m*/*z* calcd. for C_14_H_10_BNOS: [M]^+•^ 251.0571, found 251.0571. FTIR (ATR) cm^-1^: 2922, 2853, 1687 (C=O *st*), 1627, 1587, 1540, 1515, 1456, 1373, 1317, 1202, 1080, 935, 808, 772, 760, 708, 693, 672.

(**33**) Following the *General procedure E* employing 30 mol% of palladium acetate and 66 mol% of tri-(2-furyl)phosphine, the reaction between 1-iodo-[1,2]azaborinino[1,2-*a*][1,2]azaborinine (**2**) (51 mg, 0.2 mmol) and 3-bromopicolinaldehyde (56 mg, 0.3 mmol). The product was purified by automated flash chromatography (65:35, cyclohexane:ethyl acetate – cyclohexane containing 1% of Et_3_N) affording 33 mg (0.140 mmol, 70% yield) of **33** as a dark yellow solid.

^1^H NMR (400 MHz, CD_2_Cl_2_) δ 8.56 (dd, *J* = 5.0, 1.4 Hz, 1H**i**), 8.18 (d, *J* = 6.9 Hz, 1H**e**), 7.87 – 7.68 (m, 4H**a,b,d,g**), 7.29 (dd, *J* = 7.5, 5.0 Hz, 1H**h**), 7.09 (d, *J* = 6.9 Hz, 1H**f**), 6.79 (ddd, *J* = 7.0, 6.3, 1.6 Hz, 1H**c**). ^13^C NMR (100.5 MHz, CD_2_Cl_2_) δ 198.6, 157.5, 155.8, 150.9, 143.5, 140.4, 139.3, 134.2, 127.5, 126.2, 116.5, 107.3 (signals for two carbons next to boron atom are not observed). ^11^B NMR (128 MHz, CD_2_Cl_2_) δ 27.8. HRMS (ESI) *m*/*z* calcd. for C_14_H_10_BN_2_O: [M+H]^+^ 233.0881, found 233.0878. FTIR (ATR) cm^-1^: 3043, 1696 (C=O *st*), 1623, 1583, 1529, 1389, 1340, 1240, 1153, 1006, 818, 790, 743, 648.

(**34**) Following the *General procedure E* employing 30 mol% of palladium acetate and 66 mol% of tri-(2-furyl)phosphine, the reaction between 1-iodo-9-methyl-[1,2]azaborinino[1,2-*a*][1,2]azaborinine (**5**) (54 mg, 0.2 mmol) and 3-bromopicolinaldehyde (56 mg, 0.3 mmol). The product was purified by automated flash chromatography (70:30, cyclohexane:ethyl acetate – cyclohexane containing 1% of Et_3_N) affording 39 mg (0.160 mmol, 80% yield) of **34** as a brown solid.

^1^H NMR (400 MHz, CDCl_3_) δ 8.57 (dd, *J* = 5.0, 1.4 Hz, 1H**i**), 8.09 (d, *J* = 6.7 Hz, 1H**e**), 7.72 (dd, *J* = 7.5, 1.4 Hz, 1H**g**), 7.54 (d, *J* = 7.7 Hz, 1H**d**), 7.39 (dt, *J* = 6.7, 1.3 Hz, 1H**b**), 7.25 (dd, *J* = 7.5, 5.0 Hz, 1H**h**), 6.99 (d, *J* = 6.8 Hz, 1H**f**), 6.61 (t, *J* = 6.9 Hz, 1H**c**), 2.82 (s, 3H**a**). ^13^C NMR (100.5 MHz, CDCl_3_) δ 197.1, 157.3, 154.7, 150.8, 143.6, 138.2, 138.1, 131.6, 126.7, 125.9, 115.7, 106.5, 29.8 (signals for quaternary carbons next to boron atom are not observed).^11^B NMR (128 MHz, CDCl_3_) δ 28.0. GC-HRMS (orbitrap) *m*/*z* calcd. for C_15_H_11_BN_2_O: [M]^+•^ 246.0959, found 246.0961. FTIR (ATR) cm^-1^: 2920, 2852, 1707 (C=O *st*), 1581, 1371, 1156, 993, 821, 790, 743, 649.

(**35**) Following the *General procedure E* employing 30 mol% of palladium acetate and 66 mol% of tri-(2-furyl)phosphine. Reaction between 1-iodo-[1,2]azaborinino[1,2-*a*][1,2]azaborinine (**2**) (51 mg, 0.2 mmol) and 2-bromonicotinaldehyde (56 mg, 0.3 mmol). The product was purified by automated flash chromatography (65:35, cyclohexane:ethyl acetate – cyclohexane containing 1% of Et_3_N) affording 20 mg (0.086 mmol, 43% yield) of **35** as a brown solid.

^1^H NMR (400 MHz, CDCl_3_) δ 8.58 (dd, *J* = 5.2, 1.5 Hz, 1H**g**), 8.18 (d, *J* = 6.9 Hz, 1H**e**), 7.93 (d, *J* = 10.9 Hz, 1H**a**), 7.86 (dd, *J* = 7.3, 1.6 Hz, 1H**h**), 7.81 (d, *J* = 7.1 Hz, 1H**d**), 7.79 – 7.70 (m, 1H**b**), 7.40 (d, *J* = 6.9 Hz, 1H**f**), 7.23 (dd, *J* = 7.3, 5.1 Hz, 1H**i**), 6.80 (td, *J* = 6.7, 1.5 Hz, 1H**c**). ^13^C NMR (151 MHz, CDCl_3_) δ 197.6, 165.4, 158.5, 152.7, 148.3, 142.9, 140.1, 133.8, 130.4, 123.9, 116.5, 106.4 (signals for two carbons next to boron atom are not observed). ^11^B NMR (193 MHz, CDCl_3_) δ 27.1. HRMS (ESI) *m*/*z* calcd. for C_14_H_10_BN_2_O: [M+H]^+^ 233.0881, found 233.0879. FTIR (ATR) cm^-1^: 2923, 2855, 1699 (C=O *st*), 1601, 1576, 1526, 1459, 1397, 1067, 1008, 781, 761, 646.

(**36**) Following the *General procedure E* employing 30 mol% of palladium acetate and 66 mol% of tri-(2-furyl)phosphine, the reaction between 1-iodo-9-methyl-[1,2]azaborinino[1,2-*a*][1,2]azaborinine (**5**) (54 mg, 0.2 mmol) and 2-bromonicotinaldehyde (56 mg, 0.3 mmol). The product was purified by automated flash chromatography (80:20, cyclohexane:ethyl acetate – cyclohexane containing 1% of Et_3_N) affording 36 mg (0.146 mmol, 73% yield) of **36** as a brown solid.

^1^H NMR (400 MHz, CDCl_3_) δ 8.59 (dd, *J* = 5.1, 1.6 Hz, 1H**g**), 8.20 (d, *J* = 6.8 Hz, 1H**e**), 7.86 (dd, *J* = 7.3, 1.6 Hz, 1H**i**), 7.65 (d, *J* = 6.9 Hz, 1H**d**), 7.45 (dt, *J* = 6.8, 1.3 Hz, 1H**b**), 7.43 (d, *J* = 6.8 Hz, 1H**f**), 7.24 (dd, *J* = 7.3, 5.2 Hz, 1H**h**), 6.67 (t, *J* = 6.9 Hz, 1H**c**), 2.83 (s, 3H**a**). ^13^C NMR (100.5 MHz, CDCl_3_) δ 196.9, 164.8, 158.9, 152.8, 143.7, 138.3, 131.9, 130.3, 129.5, 124.0, 115.7, 106.0, 29.8 (signals for quaternary carbons next to boron atom are not observed). ^11^B NMR (128 MHz, CDCl_3_) δ 27.7. GC-HRMS (orbitrap) *m*/*z* calcd. for C_15_H_11_BN_2_O: [M]^+•^ 246.0959, found 246.0960. FTIR (ATR) cm^-1^: 3274, 1696 (C=O *st*), 1579, 1531, 1359, 785, 761.

Preparation of 11*H*-benzo[*a*]fluoren-11-one (**carbo-27**)

To a 50 mL Schlenk flask containing a magnetic stir bar, palladium acetate (9.0 mg, 10 mol%), triphenylphosphine (23 mg, 22 mol%), norbornene (113 mg, 1.2 mmol, 3.0 equiv.) and cesium carbonate (390 mg, 1.2 mmol, 3.0 equiv.) were added. Then, dimethoxyethane (8 mL, 0.05 M) was added and the suspension was degassed by bubbling argon for 10 minutes. Then, 2-bromobenzaldehyde (70 µL, 0.6 mmol, 1.5 equiv.) and 1-iodonaphthalene (102 mg, 58 µL, 0.2 mmol, 1.0 equiv.) were subsequently added. The resulting mixture was stirred first at room temperature for 5 minutes and then at 90 °C in a pre-heated oil-bath for 12 hours. Next, the mixture was allowed to reach room temperature and filtered through a pad of Celite^®^, and the pad was further washed with dichloromethane (3x30 mL). Concentration of the filtrate by rotary evaporation provided a residue which was purified by automated flash chromatography (4 g cartridge, 70:30, cyclohexane:dichloromethane) affording 80 mg of **carbo-27** (0.347 mmol, 87% yield) as an orange solid.

(**carbo-27**) ^1^H NMR (400 MHz, CDCl_3_) δ 8.96 (dd, *J* = 8.5, 0.8 Hz, 1H), 8.00 (d, *J* = 8.3 Hz, 1H), 7.79 (ddd, *J* = 8.3, 1.3, 0.7 Hz, 1H), 7.66 (d, *J* = 8.2 Hz, 1H), 7.63 – 7.55 (m, 2H), 7.53 – 7.46 (m, 1H), 7.47 – 7.41 (m, 2H), 7.31 – 7.25 (m, 1H). ^13^C NMR (100.5 MHz, CDCl_3_) δ 195.6, 146.3, 144.0, 136.0, 134.7, 134.5, 134.3, 130.3, 129.6, 129.4, 128.7, 127.0, 126.6, 124.4, 124.0, 120.1, 118.2. Data in agreement with literature values.^[6]^

(**carbo-28**) Following the *General procedure E*. Reaction between 1-iodo-8-methylnaphthalene (54 mg, 0.2 mmol) and 2-bromobenzaldehyde (55 mg, 35 μL, 0.3 mmol). The product was purified by automated flash chromatography (95:5, cyclohexane:ethyl acetate) affording 12 mg of **carbo-28** (0.049 mmol, 25% yield) as an orange solid.

^1^H NMR (400 MHz, CDCl_3_) δ 8.01 (d, *J* = 8.1 Hz, 1H**e**), 7.65 (d, *J* = 8.1 Hz, 1H**f**), 7.62 (ddd, *J* = 8.1, 1.5, 0.8 Hz, 1H**c**), 7.59 (ddt, *J* = 7.1, 1.2, 0.6 Hz, 1H**j**), 7.52 – 7.48 (m, 1H**g**), 7.44 (td, *J* = 7.4, 1.1 Hz, 1H**h**), 7.40 (ddt, *J* = 7.0, 1.7, 0.8 Hz, 1H**b**), 7.37 – 7.29 (m, 1H**d**), 7.28 (td, *J* = 7.6, 1.4 Hz, 1H**i**), 3.01 (s, 3H**a**). ^13^C NMR (100.5 MHz, CDCl_3_) δ 193.8, 148.0, 143.1, 137.8, 136.2, 136.0, 134.1, 134.1, 132.2, 131.1, 129.8, 129.4, 127.5, 126.2, 123.9, 119.4, 117.9, 25.5. GC-HRMS (orbitrap) *m*/*z* calcd. for C_18_H_12_O: [M]^+•^ 244.0883, found 244.0882. FTIR (ATR) cm^-1^: 2912, 1925, 1703 (C=O *st*), 1602, 1583, 1525, 1461, 1177, 1102, 1021, 1011, 963, 835, 787.

# 12. Post-synthetic modification of new BN-containing structures

12.1 Iodination of BN-phenanthridine 4-inv and BN-fluorenone 27. General procedure F

An oven dried Schlenk tube equipped with a magnetic stir bar was purged with argon and then charged with grinded molecular iodine (2.2 equiv.) and silver tosylate (1.3 equiv.). Then, anhydrous dichloromethane (typically 6 mL/mmol) was added, and the mixture was stirred for 30 minutes at room temperature protected from light. After cooling down to the appropriate temperature (0 °C or -40 °C depending on the substrate), the aromatic BN-substrate (1.0 equiv.) was added to the mixture as a solid. Additional anhydrous dichloromethane was added (0,6 mL/mmol) to rinse the Schlenk walls. The mixture was stirred at 0 °C for 1 hour, then slowly warmed up to room temperature and finally left stirring at this temperature for 2 hours. Afterwards, the mixture was diluted with dichloromethane and filtered through a pad of Celite^®^, and the pad was further washed with dichloromethane (3x10 mL/mmol). The resulting filtrate was extracted with a saturated solution of sodium sulfite to quench the excess of iodine. The combined organic phase was dried over anhydrous magnesium sulphate, filtered, and evaporated under reduced pressure. Finally, the iodinated desired product was purified by automated flash chromatography (4 g cartridge).

 (***iodo*-4-inv**) Following the *General procedure F*, BN-phenanthridine **4-inv** (52 mg, 0.230 mmol) was added at 0 °C to a mixture of iodine (123 mg, 0.48 mmol, 2.2 equiv.) and silver tosylate (71 mg, 0.25 mmol, 1.1 equiv.) in 1.4 mL of anhydrous dichloromethane. The product was purified by automated flash chromatography (90:10, cyclohexane:ethyl acetate – cyclohexane containing 1% of Et_3_N) affording 59 mg (0.170 mmol, 72% yield) of ***iodo*-4-inv** as a brown solid.

^1^H NMR (400 MHz, CDCl_3_) δ 9.91 (d, *J* = 7.4 Hz, 1H**c**), 9.32 (s, 1H**d**), 8.95 (d, *J* = 11.9 Hz, 1H**i**), 8.66 (d, *J* = 8.4 Hz, 1H**h**), 8.37 (d, *J* = 6.9, 0.9 Hz, 1H**a**), 8.14 (d, *J* = 8.0 Hz, 1H**e**), 7.90 (ddd, *J* = 8.4, 6.9, 1.4 Hz, 1H**g**), 7.75 (d, *J* = 11.8 Hz, 1H**j**), 7.70 (ddd, *J* = 8.0, 6.9, 1.1 Hz, 1H**f**), 6.67 (t, *J* = 6.8 Hz, 1H**b**). ^13^C NMR (100.5 MHz, CDCl_3_) δ 151.2, 148.4, 142.9, 140.0, 135.2, 134.8, 131.5, 129.5, 128.8, 126.7, 126.4, 122.4, 114.8 (signals for two carbons next to boron atom are not observed). ^11^B NMR (193 MHz, CDCl_3_) δ 28.5. HRMS (QTOF) m/z calcd. for C_15_H_11_BIN_2_: [M+H]^+^ 357.0055, found 357.0062. FTIR (ATR) cm^-1^: 2921, 2852, 1613, 1585, 1450, 1344, 1284, 1240, 1222, 760, 746, 716.

(**37**) Following the *General procedure F*, BN-fluorenone **27** (75 mg, 0.325 mmol) was added at -40 °C to a mixture of iodine (181 mg, 0.715 mmol, 2.2 equiv.) and silver tosylate (118 mg, 0.423 mmol, 1.3 equiv.) in 2 mL of anhydrous dichloromethane. The product was purified by automated flash chromatography (90:10, cyclohexane:ethyl acetate) affording 58 mg (0.162 mmol, 50% yield) of **37** as a brown solid.

^1^H NMR (400 MHz, CDCl_3_) δ 8.44 (d, *J* = 1.0 Hz, 1H**a**), 8.00 (d, *J* = 0.6 Hz, 1H**d**), 7.74 (d, *J* = 0.8 Hz, 1H**c**), 7.64 (d, *J* = 1.0 Hz, 1H**f**), 7.47 – 7.42 (m, 2H**h,i**), 7.36 (ddd, *J* = 7.1, 6.1, 2.4 Hz, 1H**g**), 7.11 (d, *J* = 6.9 Hz, 1H**e**), 6.38 (t, *J* = 7.1 Hz, 1H**b**). ^13^C NMR (100.5 MHz, CDCl_3_) δ 197.4, 161.5, 150.9, 142.7, 142.5, 135.6, 134.4, 133.4, 130.6, 123.7, 119.7, 115.8, 107.6 (signals for quaternary carbons next to boron atom are not observed). ^11^B NMR (128 MHz, CDCl_3_) δ 29.5. HRMS (ESI) *m*/*z* calcd. for C_15_H_10_BINO: [M+H]^+^ 357.9895, found 357.9897. FTIR (ATR) cm^-1^: 2979, 2920, 2835, 1692 (C=O *st*), 1582, 1521, 1460, 1372, 1171, 984, 816, 776, 728.

# 13. Synthetic elaboration of 1-iodo-BN-fluorenone 37

Sonogashira cross-coupling reaction

An oven dried 5 mL Schlenk tube equipped with a magnetic stir bar was purged with argon and charged with 1-iodo-BN-fluorenone (**37**) (31 mg, 0.087 mmol, 1 equiv.), bis(triphenylphosphine)palladium(II) dichloride (3.0 mg, 5 mol%), copper (I) iodide (1 mg, 6 mol%) and freshly distilled Et_3_N (18 μL, 0.130 mmol, 1.5 equiv.). Then, anhydrous tetrahydrofuran (0.2 mL) was added at room temperature and, under stirring, trimethylsilyl acetylene (18 μL, 0.130 mmol, 1.5 equiv.) was slowly added. The reaction was left stirring at room temperature for 15 hours. After this time, the mixture was diluted with dichloromethane and filtered through a pad of Celite^®^, and the pad was further washed with dichloromethane (3x5 mL). The filtrate was concentrated by rotary evaporation, and the resulting product was purified by automated flash chromatography (4 g cartridge, cyclohexane) affording 22 mg (0.068 mmol, 78% yield) of **38** as a brown solid.

(**38**) ^1^H NMR (400 MHz, CDCl_3_) δ 8.01 (d, *J* = 6.9 Hz, 1H**d**), 7.90 (d, *J* = 5.9 Hz, 1H**a**), 7.71 (d, *J* = 6.9 Hz, 1H**c**), 7.66 (d, *J* = 7.1 Hz, 1H**i**), 7.48 – 7.40 (m, 2H**f,g**), 7.35 (td, *J* = 7.0, 1.7 Hz, 1H**h**), 7.09 (d, *J* = 6.9 Hz, 1H**e**), 6.67 (t, *J* = 7.0 Hz, 1H**b**), 0.39 (s, 9H**j**). ^13^C NMR (100 MHz, CDCl_3_) δ 197.0, 160.3, 145.8, 143.2, 141.8, 135.6, 134.5, 133.0, 130.3, 123.5, 119.6, 114.7, 109.0, 107.5, 99.6, 0.4. (signals for quaternary carbons next to boron atom are not observed). ^11^B NMR (128 MHz, CDCl_3_) δ 28.3. HRMS (ESI) *m*/*z* calcd. for C_20_H_19_BNOSi: [M+H]^+^ 328.1324, found 328.1319. FTIR (ATR) cm^-1^: 2956, 2137 (C≡C *st*), 1699 (C=O *st*), 1621, 1579, 1524, 1381, 1347, 1244, 1100, 880, 838, 759, 734, 645.

Deprotection of the TMSi group of BN-fluorenone derivative **39**:

An oven dried round bottomed flask with magnetic stir bar and septum was charged with compound **38** (15 mg, 0.046 mmol, 1.0 equiv.). Then, a solution of tetra-*n*-butylammonium fluoride in tetrahydrofuran (0.5 mL, 1 M, 10.0 equiv.) was added at room temperature under argon atmosphere. The solution was left stirring at this temperature for 1 hour. After this time, the reaction crude was diluted with ethyl acetate (8 mL) and washed with water to remove the ammonium salts (3 x 5 mL). The organic phase was dried over anhydrous magnesium sulphate, filtered and evaporated under reduced pressure. Finally, the residue was purified by flash column chromatography (80:20, cyclohexane:ethyl acetate) affording 8 mg of **39** (0.031 mmol, 67% yield) as a yellow solid.

(**39**) ^1^H NMR (400 MHz, CDCl_3_) δ 8.03 (d, *J* = 6.9 Hz, 1H**d**), 7.92 (d, *J* = 8.1 Hz, 1H**a**), 7.73 (d, *J* = 8.2 Hz, 1H**c**), 7.64 (dt, *J* = 7.1, 1.0 Hz, 1H**i**), 7.47 – 7.40 (m, 2H**f,g**), 7.39 – 7.31 (m, 1H**h**), 7.10 (d, *J* = 6.9 Hz, 1H**e**), 6.68 (t, *J* = 7.0 Hz, 1H**b**), 3.62 (s, 1H**j**). ^13^C NMR (100.5 MHz, CDCl_3_) δ 198.0, 160.8, 146.5, 143.2, 142.2, 135.5, 134.8, 133.3, 130.5, 123.5, 119.8, 114.7, 107.7, 87.5, 82.1 (signals for quaternary carbons next to boron atom are not observed). ^11^B NMR (128 MHz, CDCl_3_) δ 28.2. HRMS (ESI) *m*/*z* calcd. for C_17_H_11_BNO: [M+H]^+^ 256.0929, found 256.0903. FTIR (ATR) cm^-1^: 3290 (≡C-H *st*), 2100 (C≡C *st*), 1696 (C=O *st*), 1621, 1581, 1524, 1380, 1346, 1165, 1099, 961, 815, 730, 651.

Gold-catalyzed hydration of 1-ethynyl BN-fluorenone **40**:

A 5-mL Schlenk tube equipped with a magnetic stir bar was charged with 1-ethynyl-12*H*-[1,2]azaborinino[1,2-*a*]indeno[2,1-*c*][1,2]azaborinin-12-one (**39**) (8 mg, 0.031 mmol, 1.0 equiv.) and methanol (0.5 mL). Then, a solution of HAuCl_4_·3H_2_O in H_2_O (70 µL, 0.1 M) and 1 drop of H_2_SO_4_ were added at room temperature. The resulting mixture was left stirring at 70 °C for 45 minutes. After this time, a color change was observed from dark yellow to dark brown. The reaction mixture was allowed to cool to room temperature and transferred to a vial with saturated sodium bicarbonate. The resulting mixture was extracted with ethyl acetate (3x5 mL) and the combined organic phase was dried over anhydrous magnesium sulphate, filtered and evaporated under reduced pressure. Finally, the residue was purified by flash column chromatography (50:50, cyclohexane:ethyl acetate) affording 5 mg of **40** (0.019 mmol, 60% yield) as a yellow solid.

(**40**) ^1^H NMR (400 MHz, CDCl_3_) δ 8.08 (d, *J* = 6.8 Hz, 1H**d**), 7.77 (d, *J* = 6.4 Hz, 1H**a**), 7.59 (dd, *J* = 7.2, 0.9 Hz, 1H**c**), 7.51 (d, *J* = 6.7 Hz, 1H**i**), 7.48 – 7.41 (m, 2H**f,g**), 7.38 – 7.31 (m, 1H**h**), 7.12 (d, *J* = 6.9 Hz, 1H**e**), 6.71 (t, *J* = 6.9 Hz, 1H**b**), 2.63 (s, 3H**j**). ^13^C NMR (151 MHz, CDCl_3_) δ 209.4, 198.5, 160.8, 143.5, 142.2, 135.5, 135.5, 135.1, 133.5, 130.6, 123.8, 120.1, 114.4, 107.8, 32.2 (signals for quaternary carbons next to boron atom are not observed). ^11^B NMR (193 MHz, CDCl_3_) δ 25.2. HRMS (ESI) *m*/*z* calcd. for C_17_H_13_BNO_2_: [M+H]^+^ 274.1034, found 274.1029. FTIR (ATR) cm^-1^: 2922, 2852, 1686 (C=O *st*), 1584, 1532, 1346, 1259, 1168, 817, 760, 732, 653.

# 14. Synthesis of inverted BN-doped fluorenone species (27-inv)

A 100 mL Schlenk flask equipped with a magnetic stir bar was charged with palladium acetate (12 mg, 10 mol%), tri-(2-furyl)phosphine (27 mg, 22 mol%), norbornene (251 mg, 2.66 mmol, 5.0 equiv.) and cesium carbonate (520 mg, 1.60 mmol, 3.0 equiv.). Then, dimethoxyethane (10 mL) was added, and the suspension was degassed by bubbling argon for 10 minutes. Subsequently, 2-bromobenzaldehyde (148 mg, 93 μL, 0.800 mmol) was introduced, followed by the addition of 4-iodo-[1,2]azaborinino[1,2-*a*][1,2]azaborinine (**2-inv**) (136 mg, 0.533 mmol) dissolved in 1 mL of dimethoxyethane. The resulting mixture was stirred at room temperature for 5 minutes, then placed in a pre-heated oil-bath at 90 °C for 12 hours. Next, the mixture was allowed to reach room temperature and filtered through a pad of Celite^®^, and the pad was further washed with dichloromethane (3x30 mL). Concentration of the filtrate by rotary evaporation provided a residue which was purified by automated flash chromatography (12 g cartridge, cyclohexane) affording 18 mg (0.03 mmol, 15% yield) of **27-inv** as a red solid.

(**27-inv**) ^1^H NMR (600 MHz, CDCl_3_) δ 9.63 (d, *J* = 7.1 Hz, 1H**d**), 7.92 (d, *J* = 11.0 Hz, 1H**k**), 7.80 (d, *J* = 11.0 Hz, 1H**i**), 7.73 (ddd, *J* = 11.1, 6.4, 1.1 Hz, 1H**b**), 7.49 (d, *J* = 7.1 Hz, 1H**e**), 7.44 (d, *J* = 11.3 Hz, 1H**a**), 7.38 (td, *J* = 7.4, 1.1 Hz, 1H**g**), 7.34 (d, *J* = 7.2 Hz, 1H**h**), 7.18 (td, *J* = 7.4, 1.1 Hz, 1H**f**), 6.88 (ddd, *J* = 7.1, 6.5, 1.6 Hz, 1H**c**). ^13^C NMR (151 MHz, CDCl_3_) δ 191.4, 142.8, 139.5, 138.9, 134.8, 131.5, 131.4, 131.2, 129.5, 128.7, 124.0, 119.4, 116.9 (signal for C-H carbons next to boron atom are not observed). ^11^B NMR (193 MHz, CDCl_3_) δ 27.9. HRMS (ESI) *m*/*z* calcd. for C_15_H_11_BNO: [M+H]^+^ 232.0929, found 232.0879. FTIR (ATR) cm^-1^: 3027, 2951, 2861, 1695 (C=O *st*), 1603, 1456, 1274, 1182, 999, 905, 847, 802, 751, 685, 629.

Side-product structures obtained in the [3+2]-*BN-Arex* reaction with **2-inv** and **3**-Br.

As mentioned in the Main text, the formation of **27-inv** was accompanied by two other side-products: compound **41**, obtained as an atropoisomeric brown solid mixture (purified with 95:5, cyclohexane:ethyl acetate, 59 mg, 0.181 mmol, 34% yield); and the fused cyclobutene side product **42**, isolated as a viscous yellowish oil during the same purification (cyclohexane), affording 42 mg (0.192 mmol, 36% yield).

(**41**) Two rotamers were observed at room temperature: ^1^H NMR (600 MHz, CDCl_3_) δ 9.97 (s, 0.5H**a**), 9.89 (s, 0.5 H**a**), 8.08 (d, *J* = 7.9 Hz, 0.5H**b**), 8.04 (d, *J* = 7.8 Hz, 0.5H**b**), 7.77 – 7.68 (m, 1H**i**), 7.71 – 7.68 (m, 0.5H**d**), 7.67 – 7.60 (m, 0.5H**d**, 0.5H**f**), 7.55 – 7.48 (m, 1H**c**, 0.5 H**f**), 7.45 (dd, *J* = 7.6, 4.5 Hz, 1H**e**), 7.31 – 7.20 (m, 0.5H**g**), 7.25 – 7.09 (m, 1H**h**, 0.5 H**g**), 6.69 – 6.60 (m, 1H**j**), 3.56 (d, *J* = 8.0 Hz, 0.5H**p**), 3.42 (dd, *J* = 15.5, 8.0 Hz, 1H**k**), 3.14 (d, *J* = 8.0 Hz, 0.5H**p**), 2.47 (d, *J* = 14.1 Hz, 1H**l**), 1.84 (s, 1H**o**), 1.58 – 1.51 (m, 1H**m**), 1.41 – 1.31 (m, 1H**m**, 1H**n**), 1.14 – 1.03 (m, 1H**n**), 1.01 – 0.95 (m, 1H**q**), 0.84 (d, *J* = 10.6 Hz, 1H**q**). ^13^C NMR (151 MHz, CDCl_3_) δ 192.7, 192.4, 152.2, 152.0, 150.5, 150.5, 148.3, 147.8, 144.4, 144.3, 143.0, 141.8, 140.1, 134.7, 134.1, 133.8, 132.0, 130.8, 128.2, 128.1, 127.8, 127.4, 119.1, 118.9, 108.9, 108.8, 50.5, 50.5, 50.3, 49.9, 44.8, 44.6, 41.5, 41.3, 33.6, 33.5, 28.7, 28.7, 28.3, 28.2. (signals for C-H carbons next to boron atom are not observed). ^11^B NMR (128 MHz, CDCl_3_) δ 27.5. HRMS (ESI) *m*/*z* calcd. for C_22_H_21_BNO: [M+H]^+^ 326.1711, found 326.1706. FTIR (ATR) cm^-1^: 2952, 2869, 1689 (C=O *st*), 1599, 1525, 1436, 1392, 1333, 1190, 817, 760, 690.

Variable Temperature NMR study to confirm the atropoisomeric nature of species **41**:

The presence of rotamers in **41** was further assessed by redissolving the sample in DMSO-*d_6_* and performing ^1^H VT NMR experiments. As shown in **Figure S1**, the two sets of signals from each rotamer coalesced upon increasing temperature, yielding one set of signals at 120 °C, a temperature in which fast exchange regime (kex >> |Δν|) is reached.


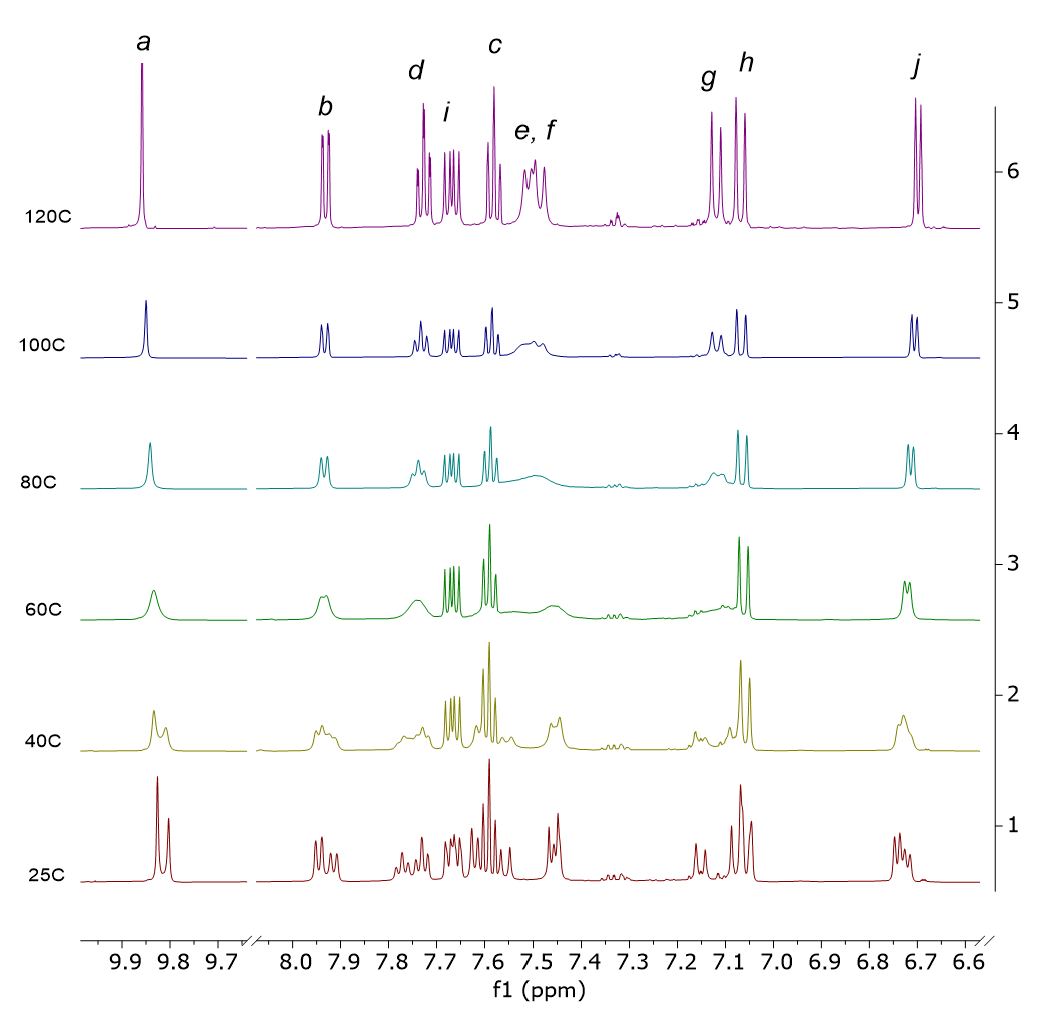


**Figure S1.** ^1^H VT NMR experiments for **41** from 25 to 120 °C in DMSO-*d_6_*.

(**42**) ^1^H NMR (400 MHz, CDCl_3_) δ 7.84 (d, *J* = 6.8 Hz, 1H**d**), 7.70 (dd, *J* = 10.9, 6.2 Hz, 1H**b**), 7.47 – 7.41 (m, 2H**a,e**), 7.32 (d, *J* = 10.7 Hz, 1H**f**), 6.77 (td, *J* = 6.6, 1.4 Hz, 1H**c**), 3.37 (d, *J* = 3.4 Hz, 1H), 3.07 (d, *J* = 3.3 Hz, 1H), 2.43 (s, 1H), 2.32 (s, 1H), 1.70 – 1.62 (m, 2H), 1.38 – 1.20 (m, 2H), 0.97 (dt, *J* = 10.4, 1.5 Hz, 1H), 0.76 (dt, *J* = 10.4, 2.0 Hz, 1H). ^13^C NMR (100.5 MHz, CDCl_3_) δ 141.0, 137.7, 135.0, 129.1, 127.5, 114.3, 50.1, 47.1, 36.2, 34.6, 31.9, 28.2, 27.9 (signals for two carbons next to boron atom are not observed). ^11^B NMR (128 MHz, CDCl_3_) δ 28.7.

# 15. Characterization data

 ^1^H NMR (400 MHz, CDCl_3_)


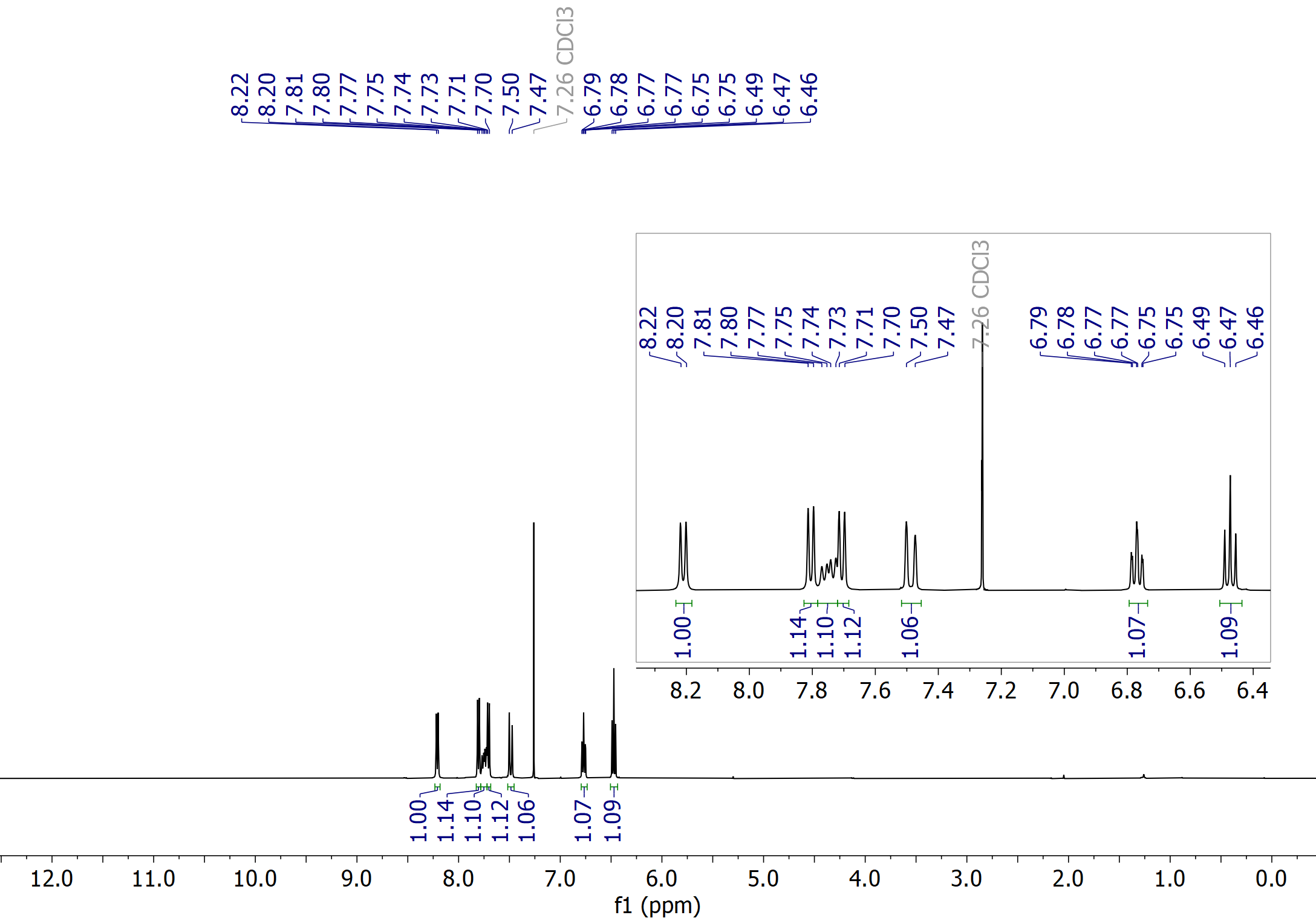


^13^C NMR (100.5 MHz, CDCl_3_)


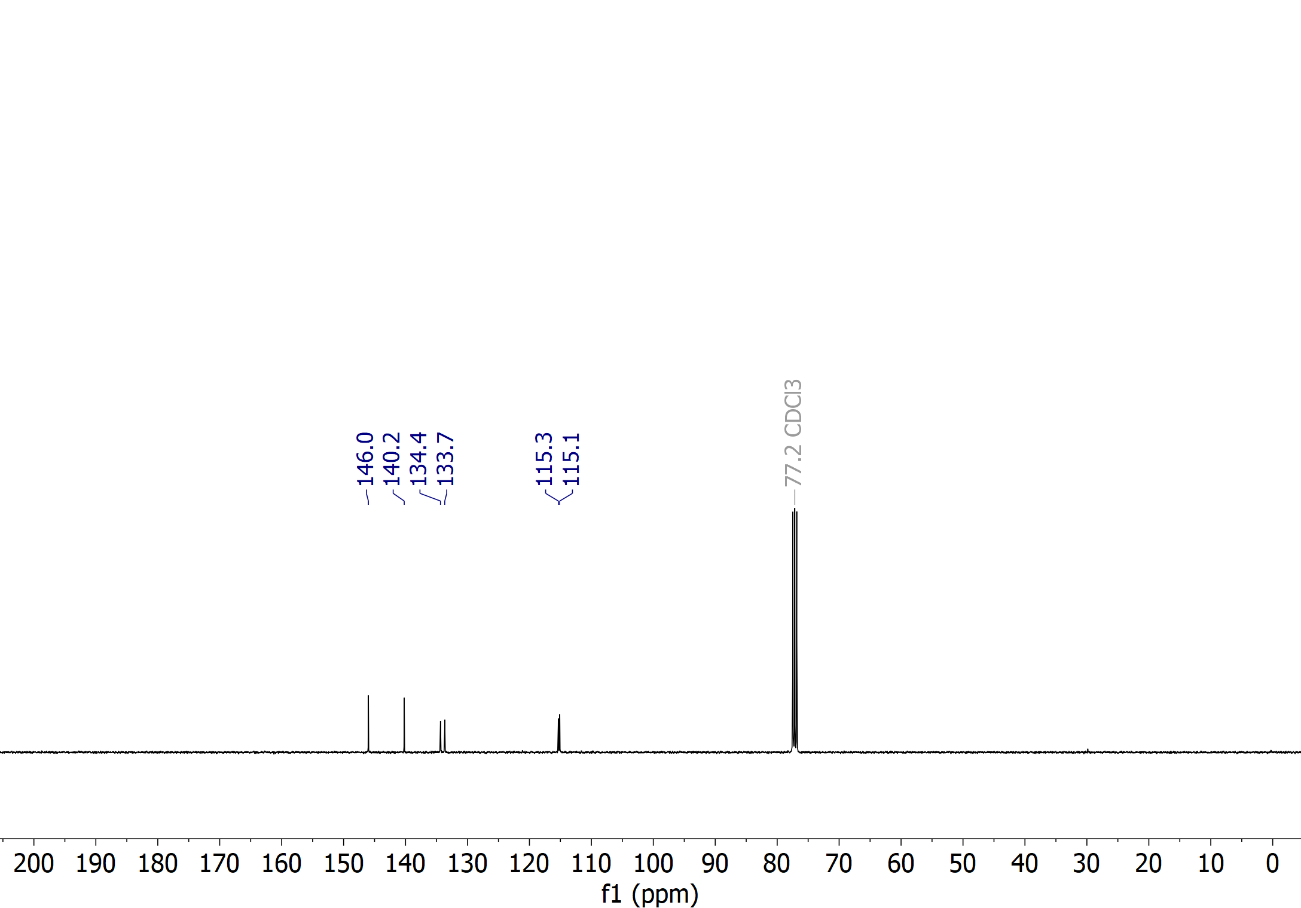


^11^B NMR (128 MHz, CDCl_3_)


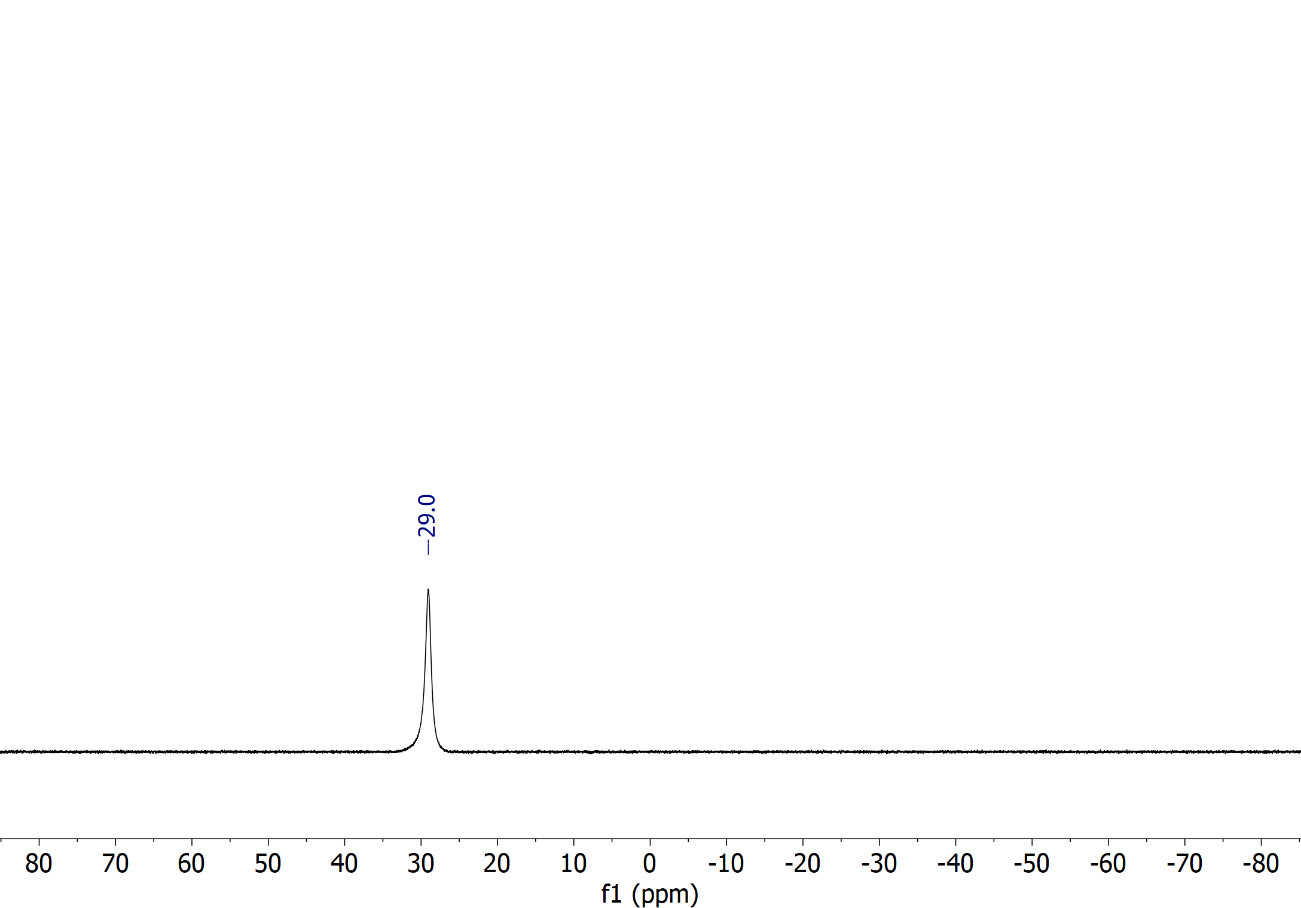

 ^1^H NMR (400 MHz, CDCl_3_)


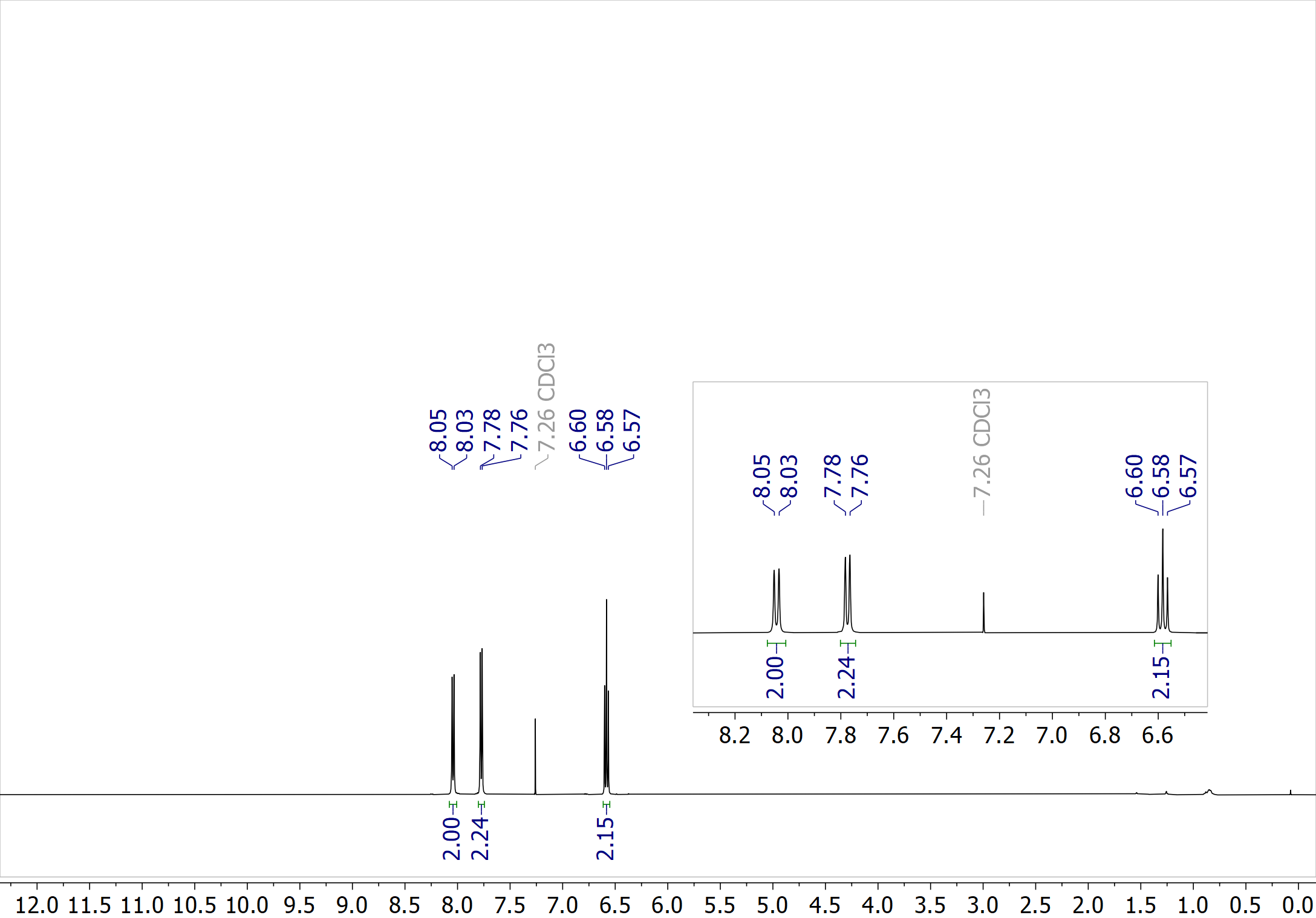

 ^1^H NMR (400 MHz, CDCl_3_)


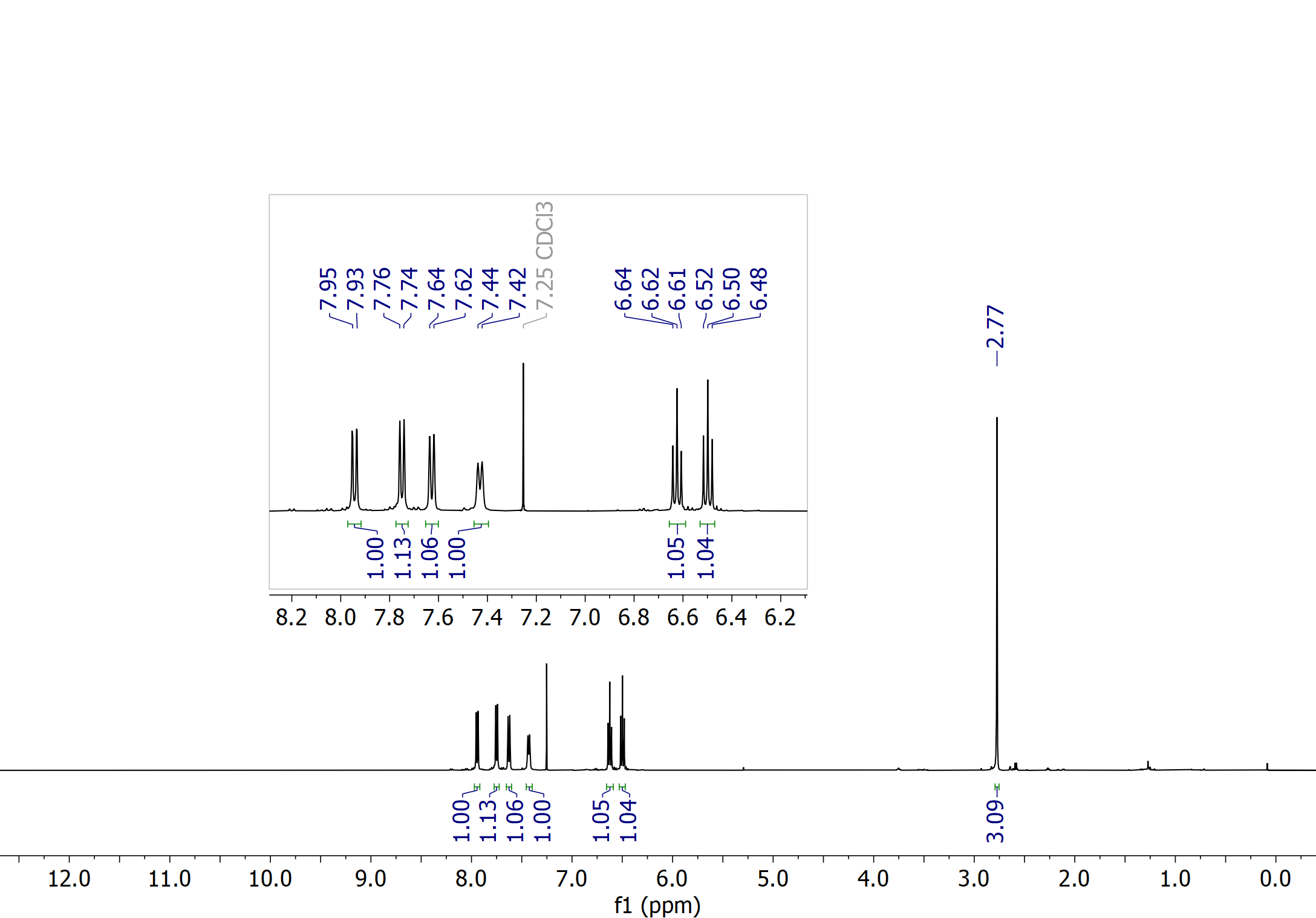

 ^13^C NMR (100.5 MHz, CDCl_3_)


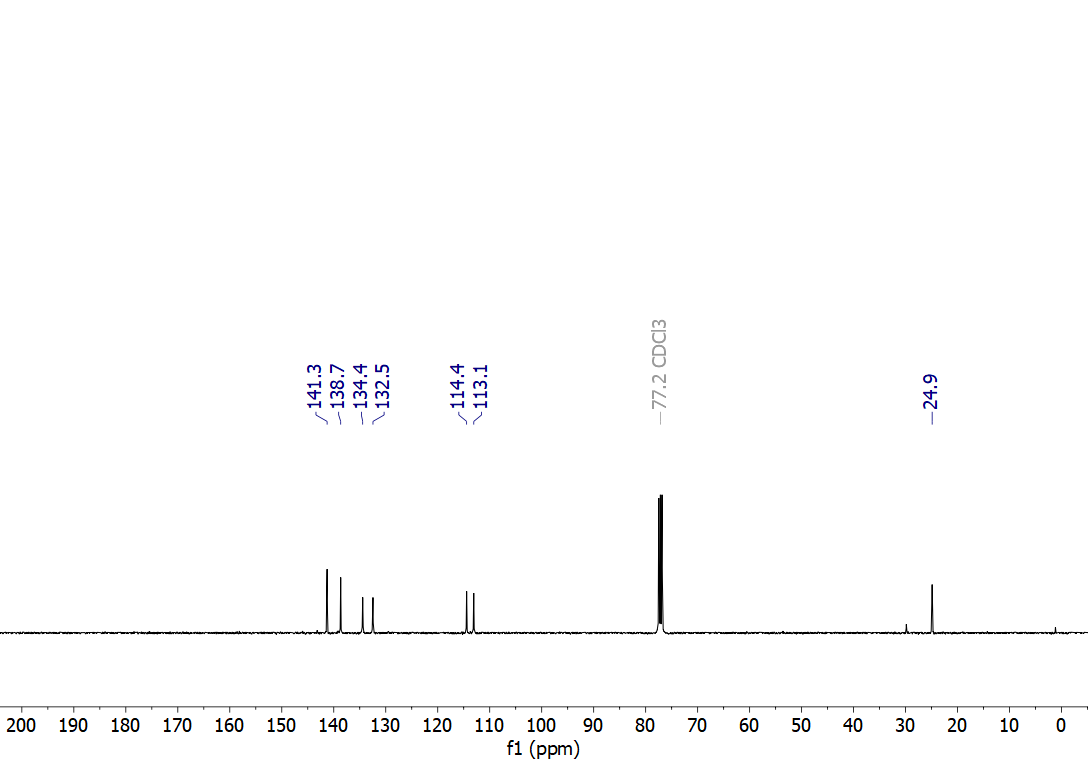


^11^B NMR (128 MHz, CDCl_3_)


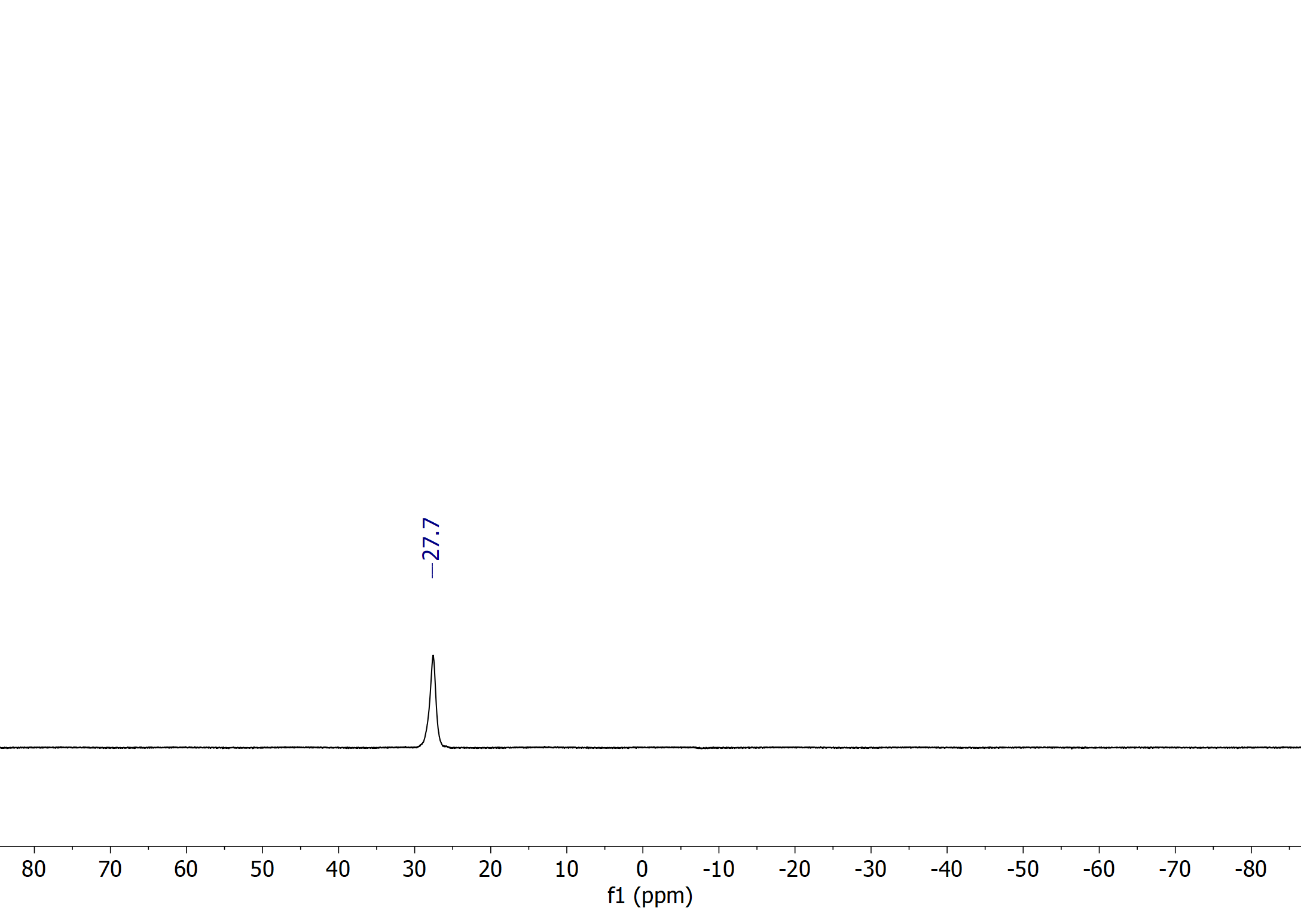

 ^1^H NMR (400 MHz, CDCl_3_)


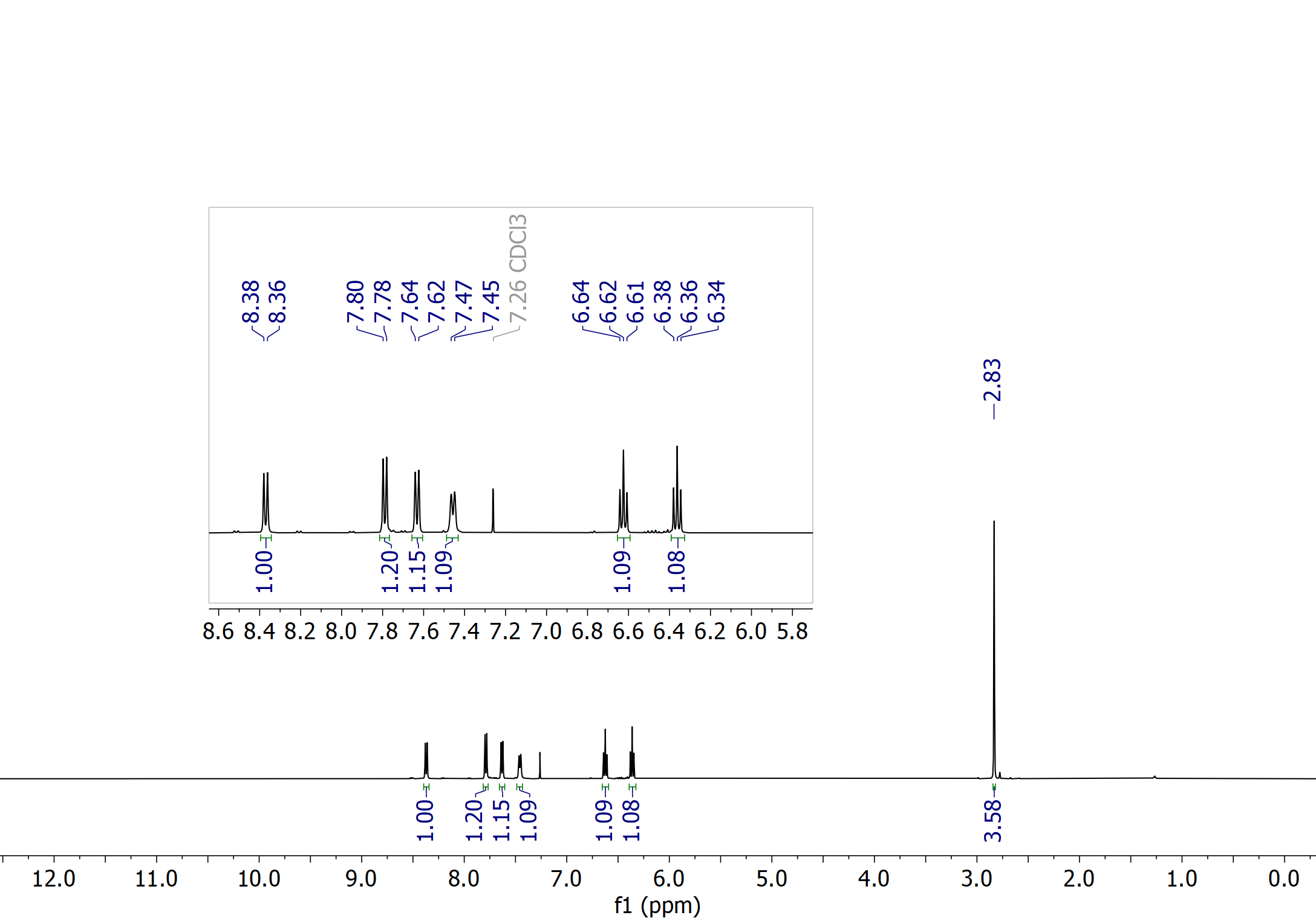


^13^C NMR (100.5 MHz, CDCl_3_)


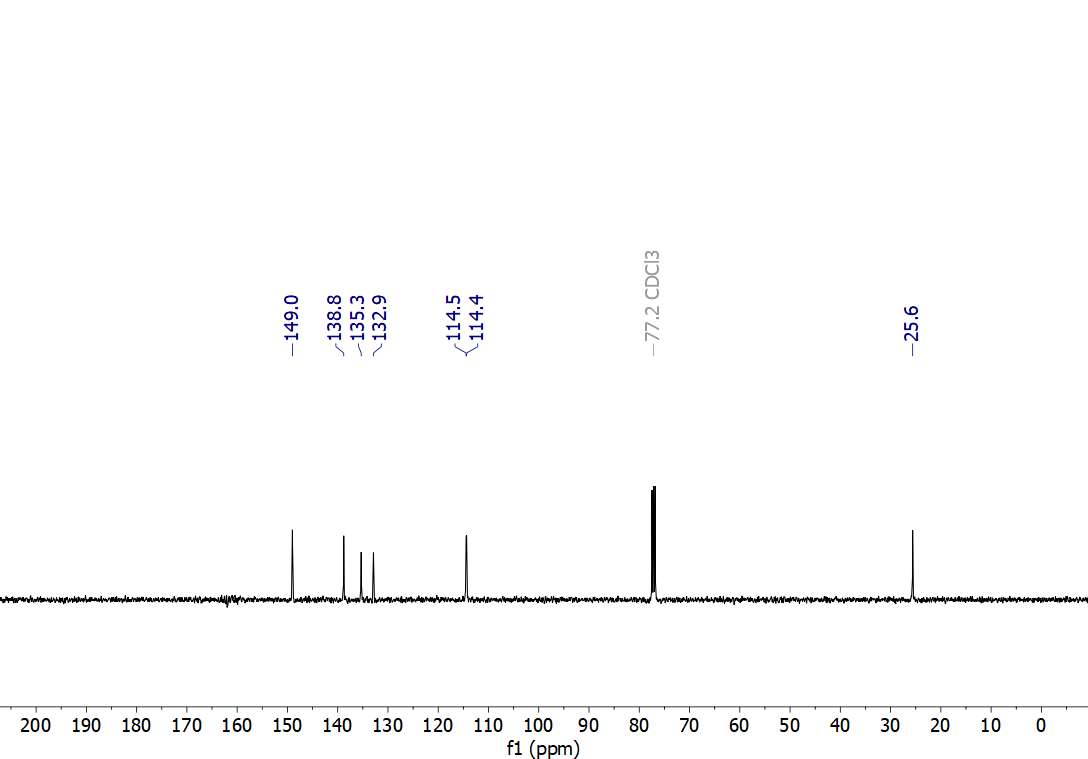


^11^B NMR (128 MHz, CDCl_3_)


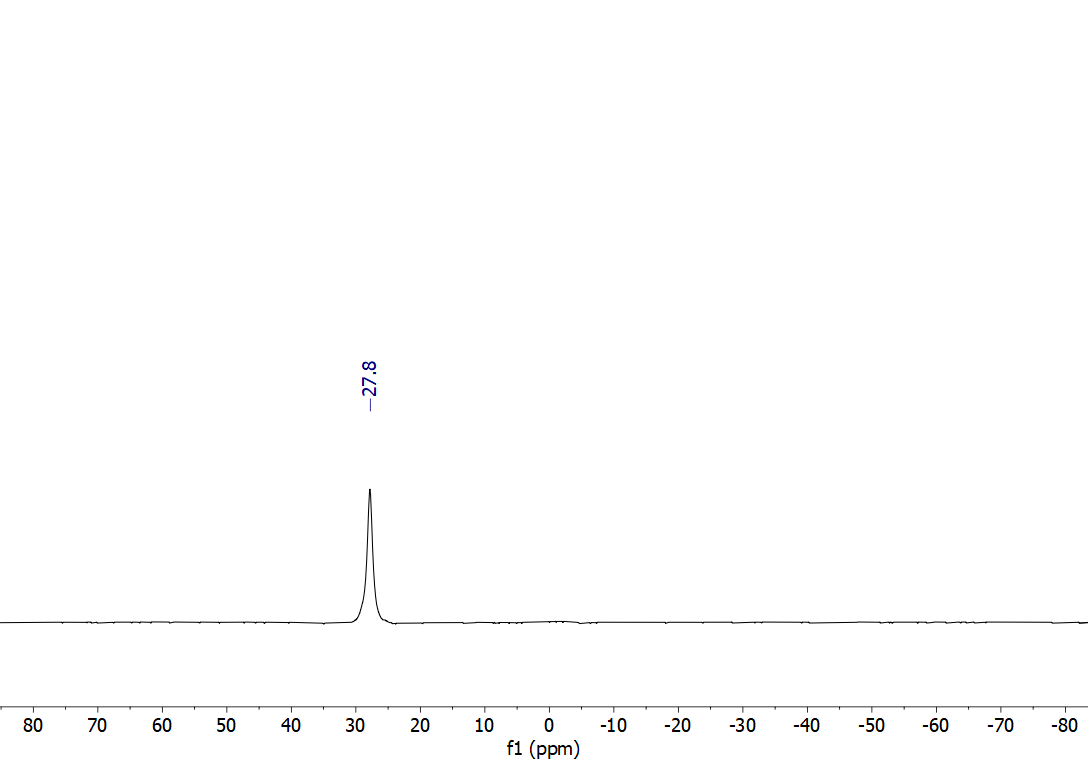

 ^1^H NMR (400 MHz, CDCl_3_)


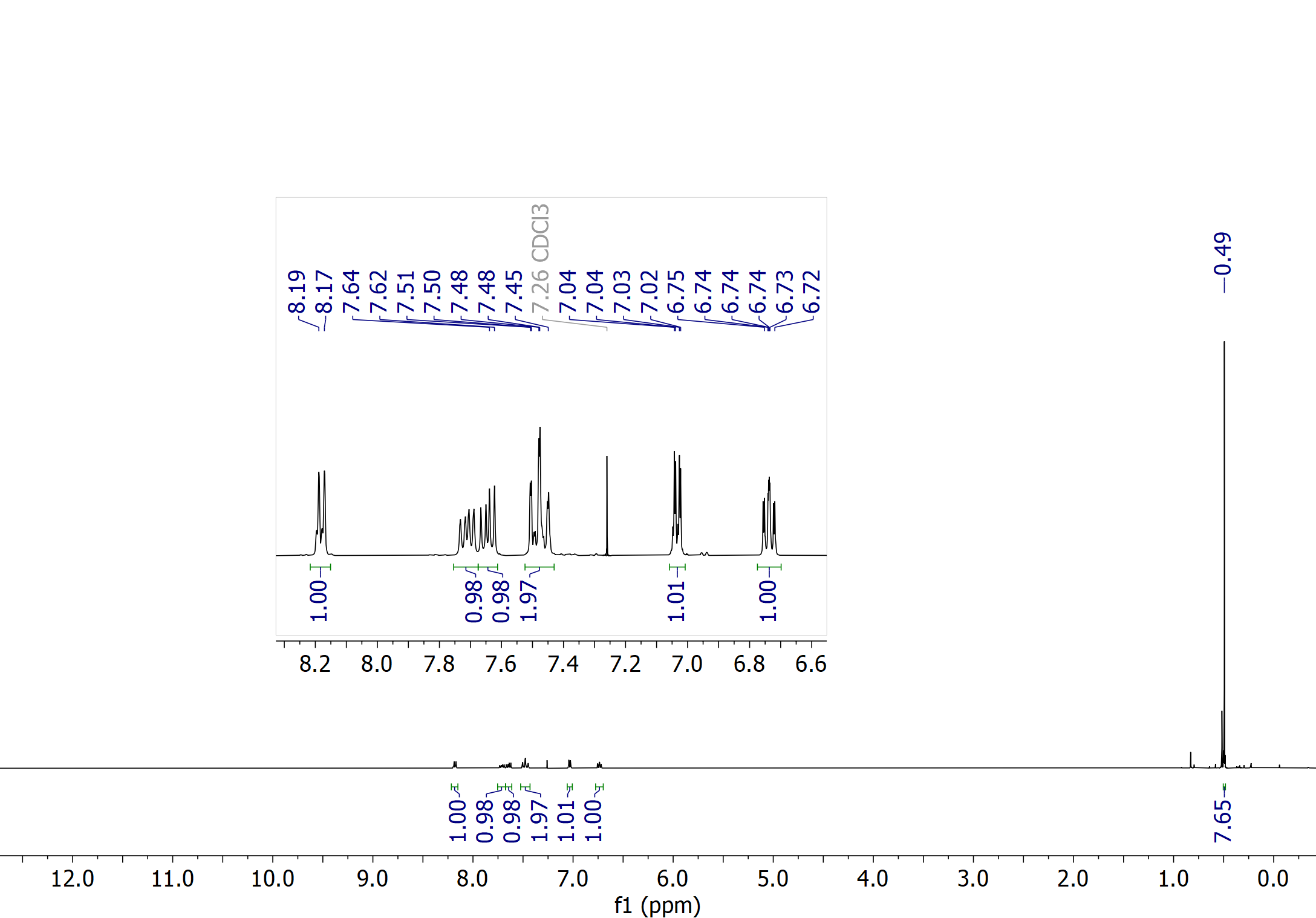


^13^C NMR (100.5 MHz, CDCl_3_)


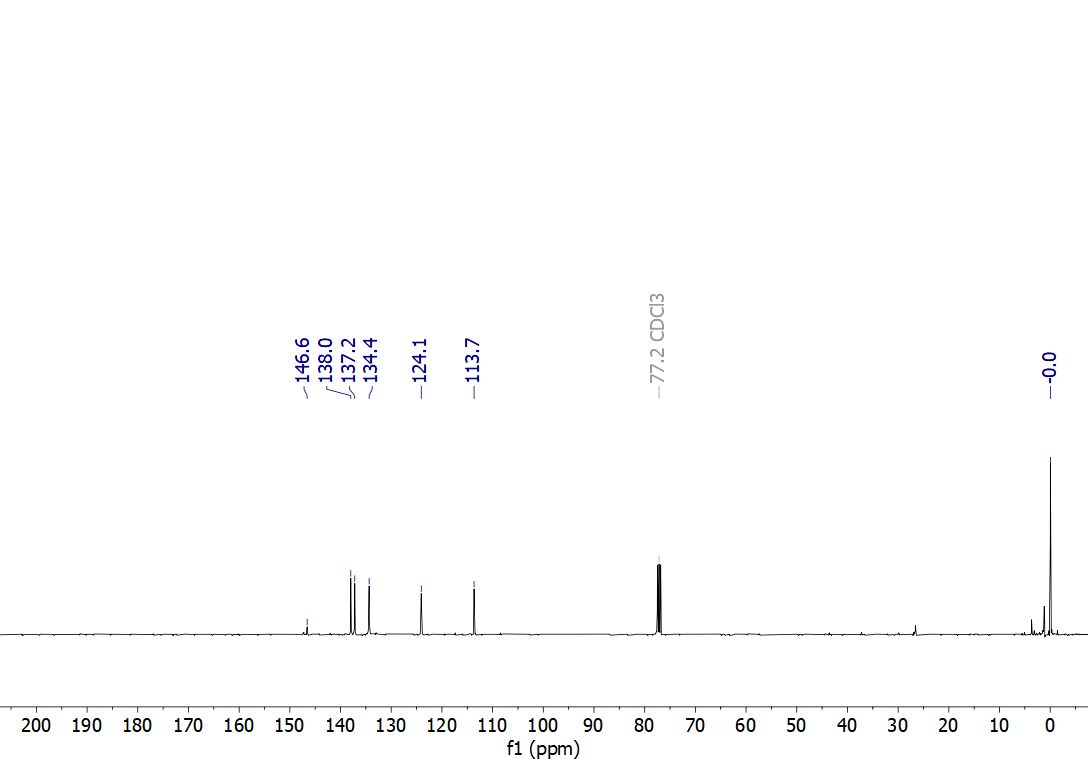


^11^B NMR (128 MHz, CDCl_3_)


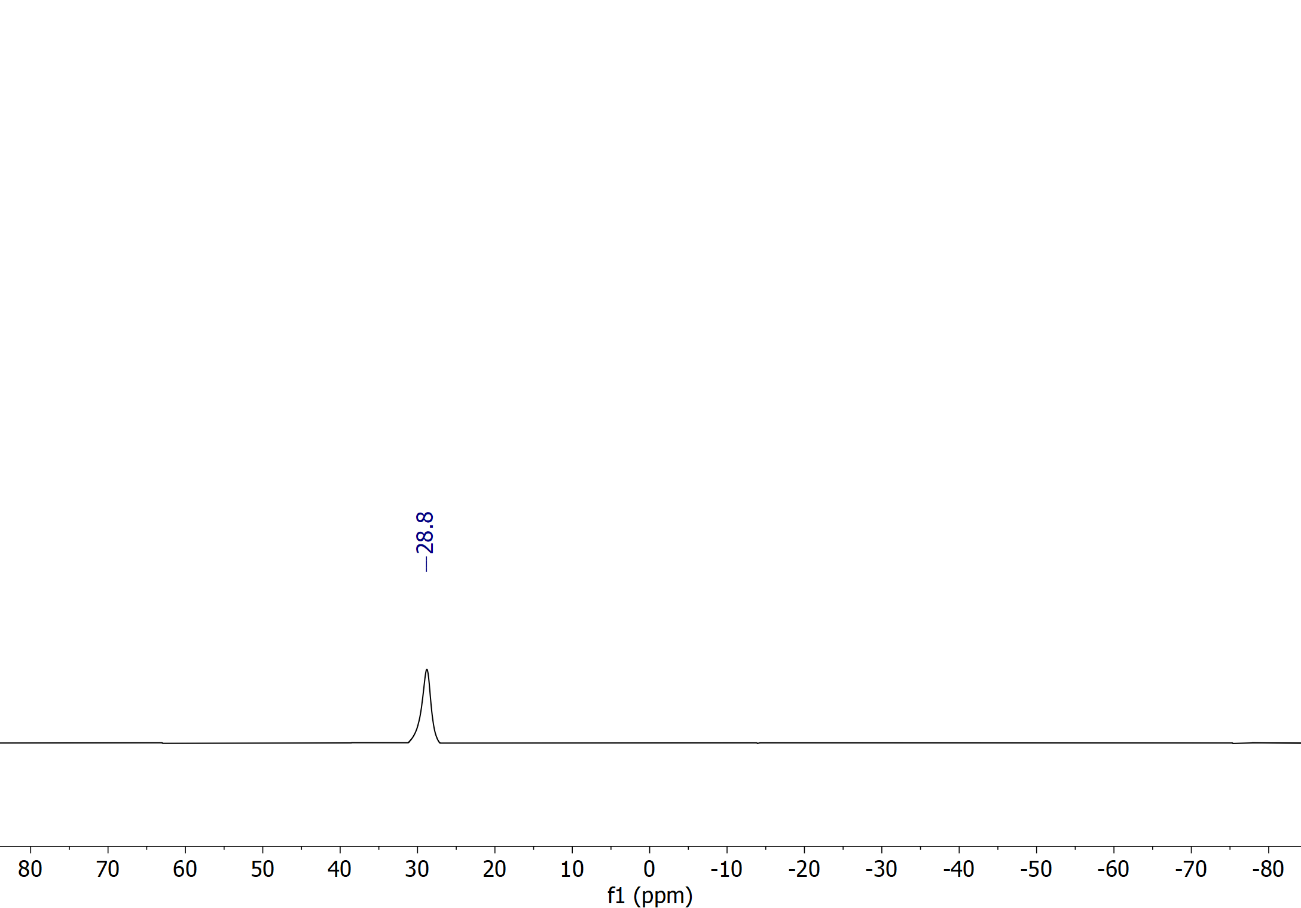

 ^1^H NMR (400 MHz, CDCl_3_)


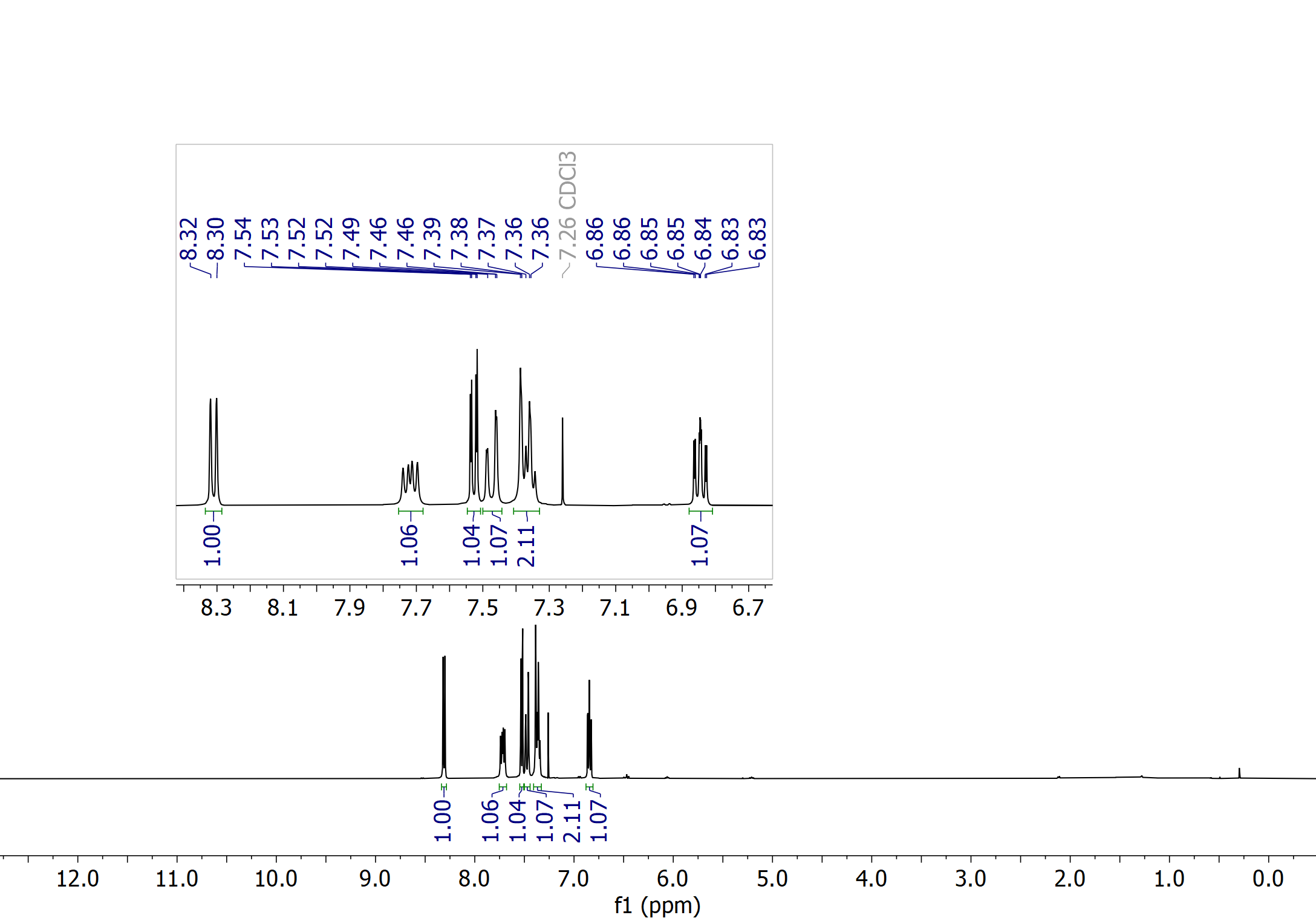


^13^C NMR (100.5 MHz, CDCl_3_)


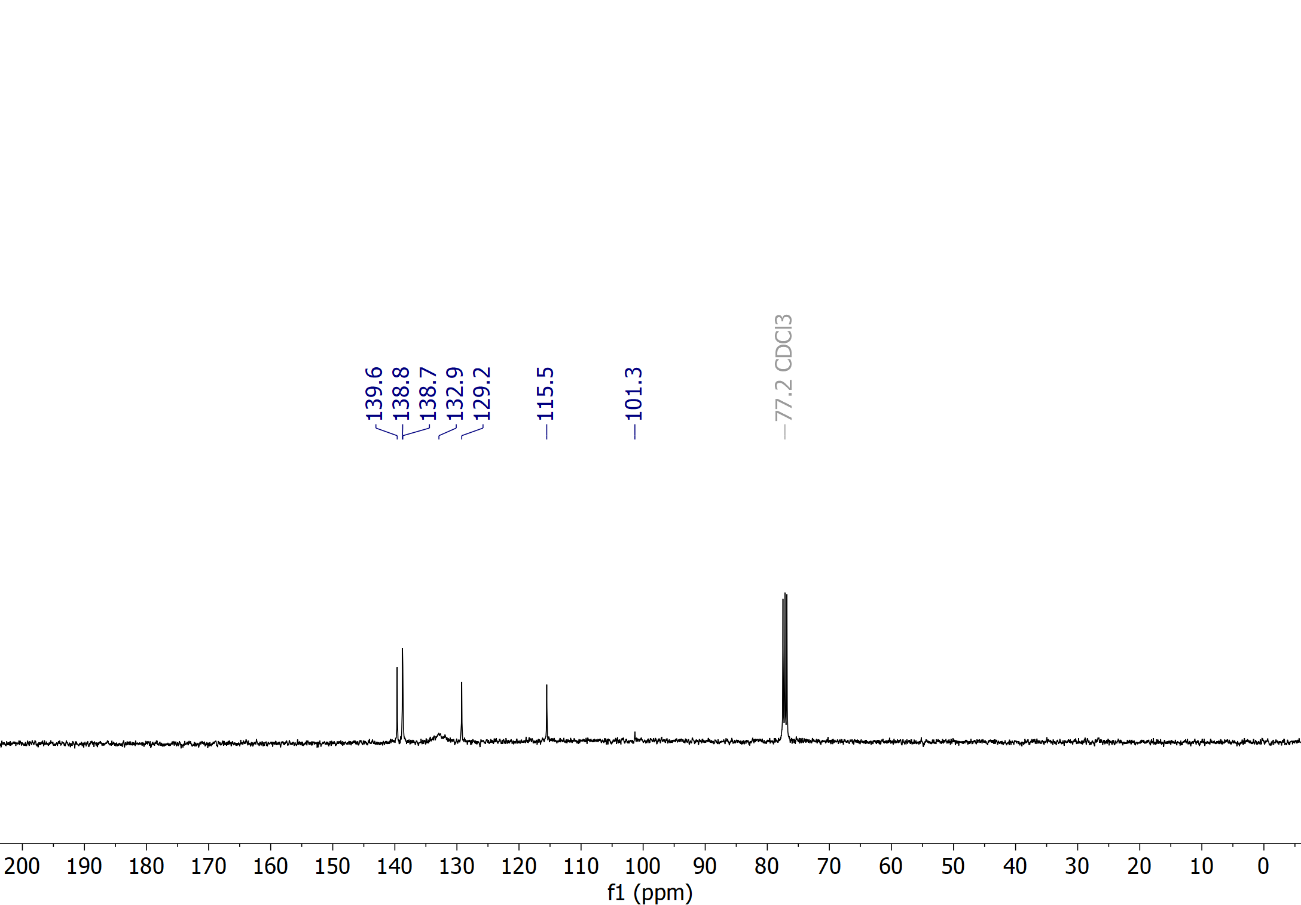


^11^B NMR (128 MHz, CDCl_3_)


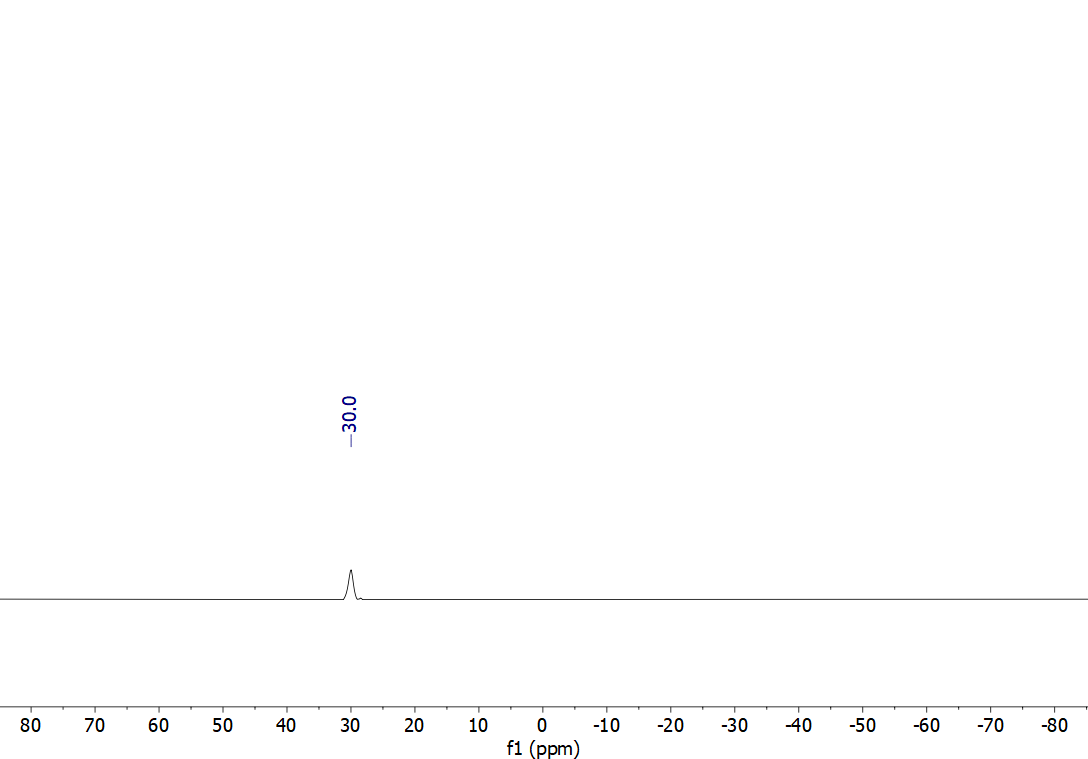

 ^1^H NMR (400 MHz, CDCl_3_)


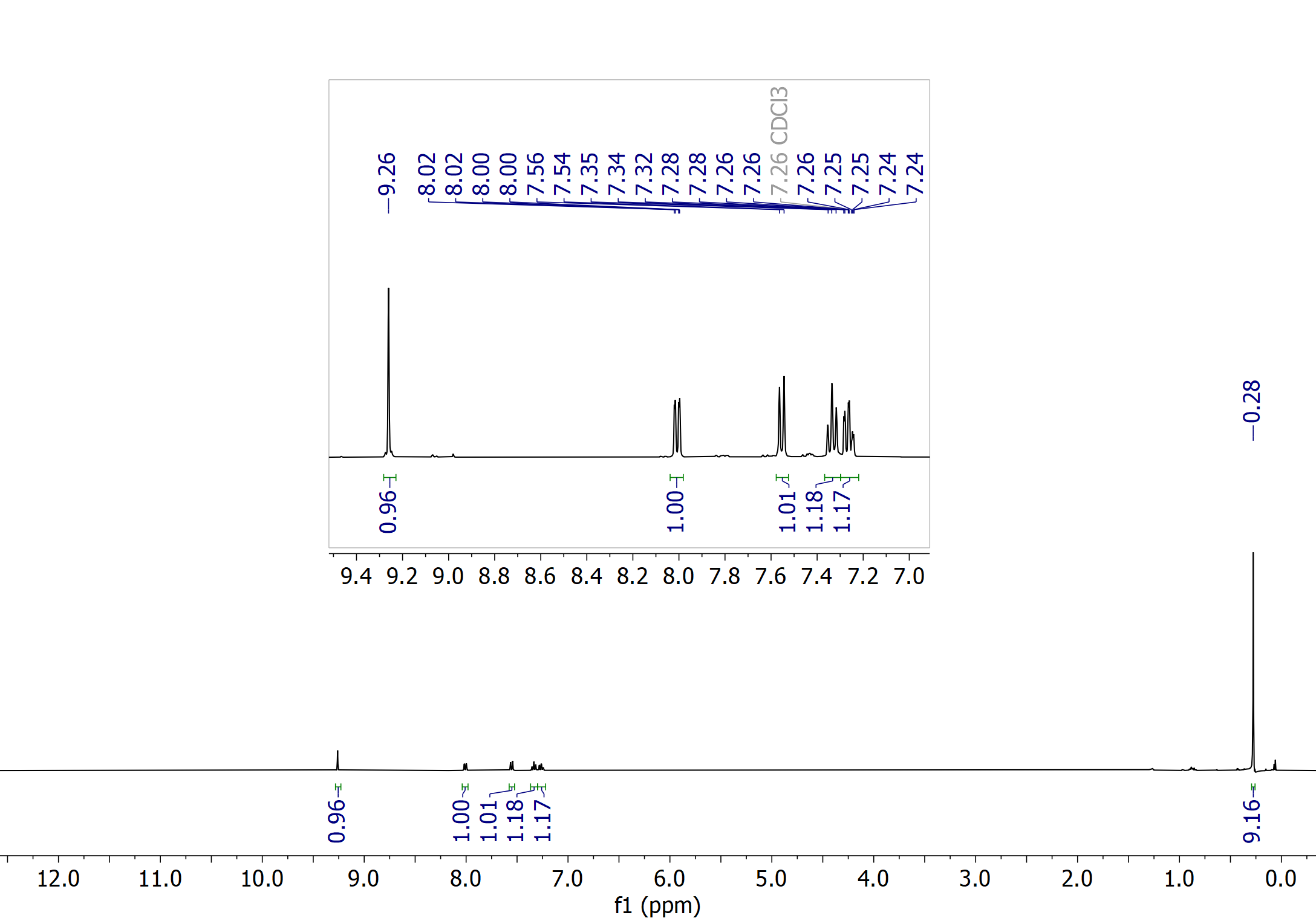

 ^1^H NMR (400 MHz, CDCl_3_)


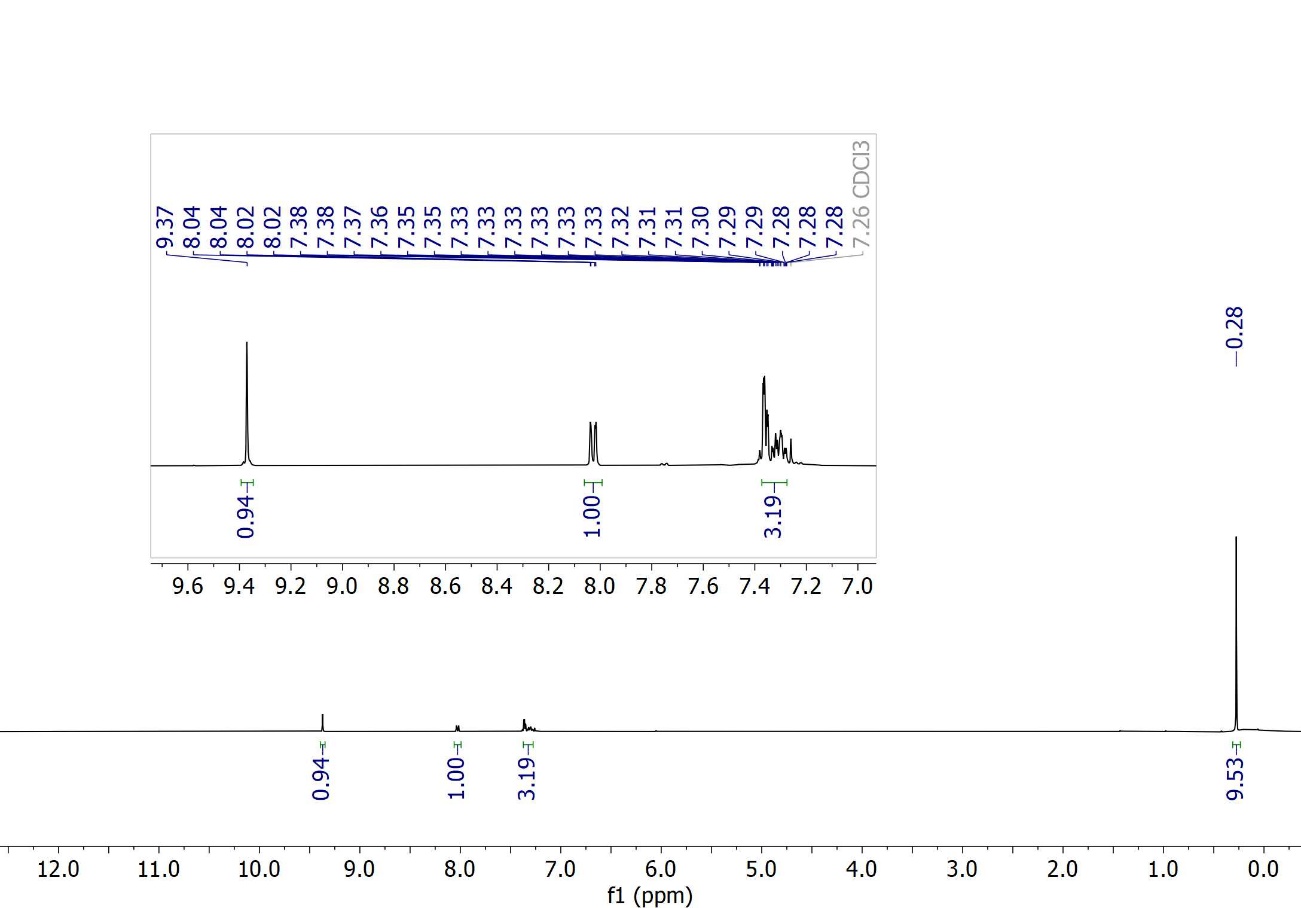

 ^1^H NMR (400 MHz, CDCl_3_)


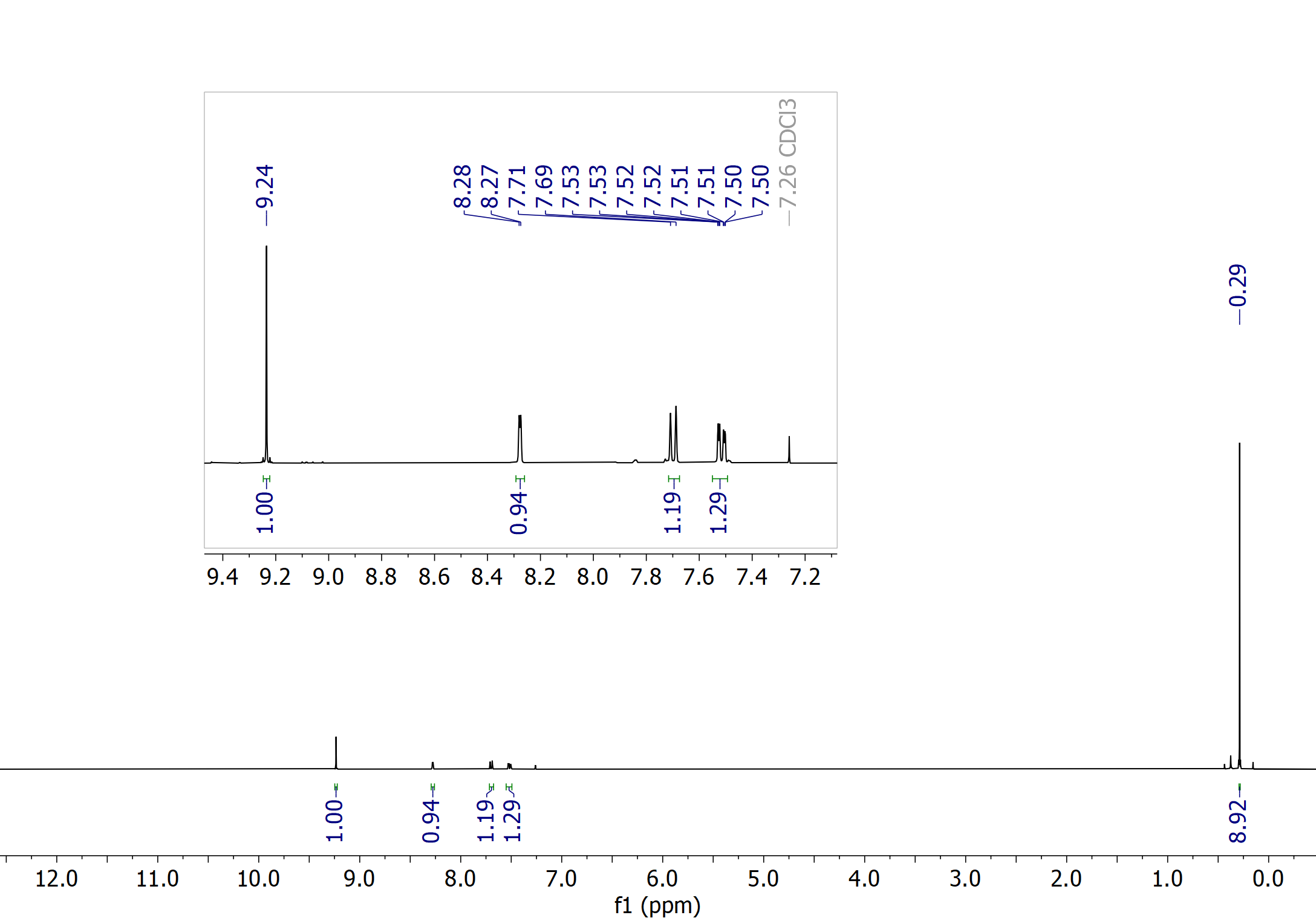

 ^1^H NMR (400 MHz, CDCl_3_)


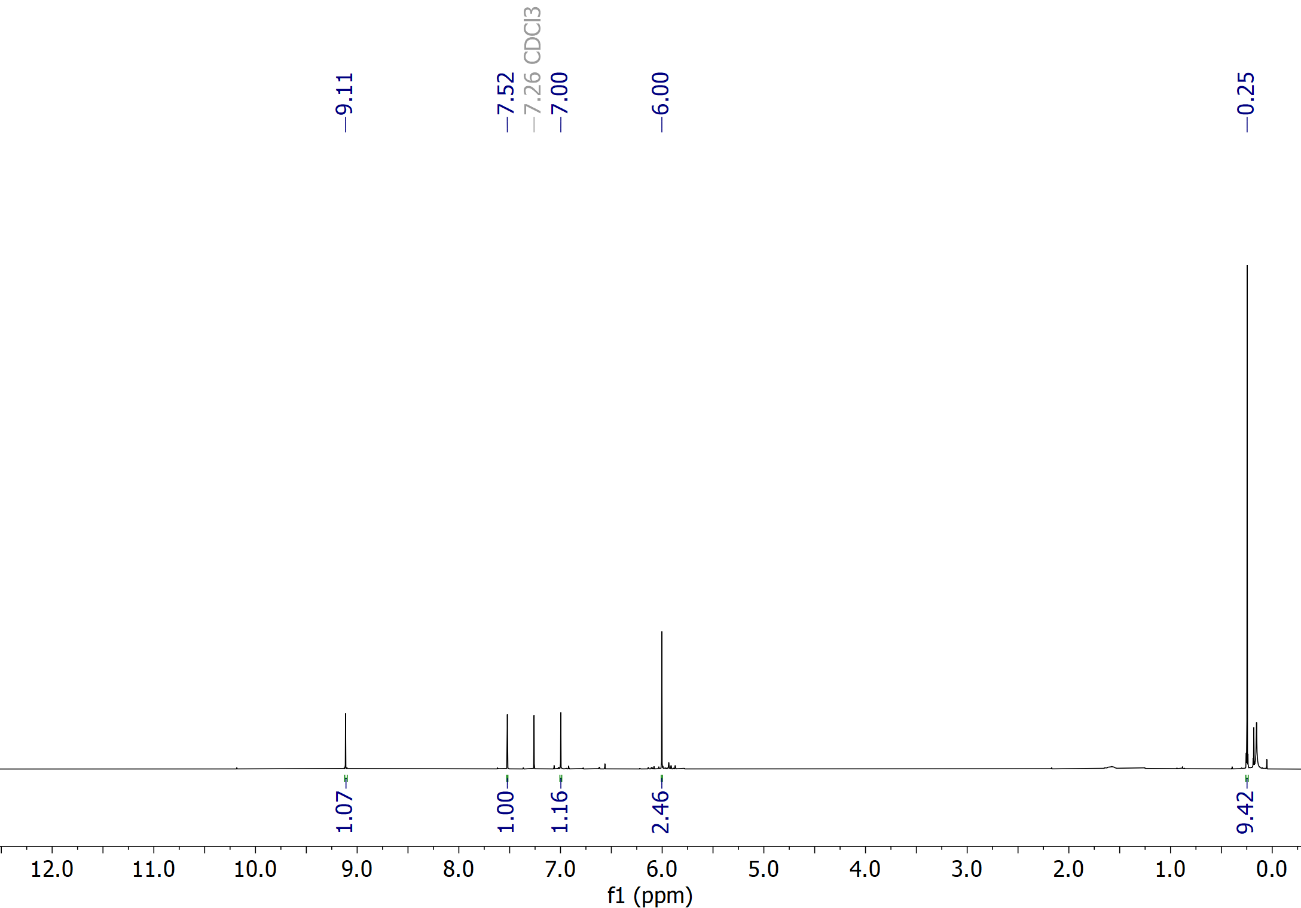

 ^13^C NMR (100.5 MHz, CDCl_3_)


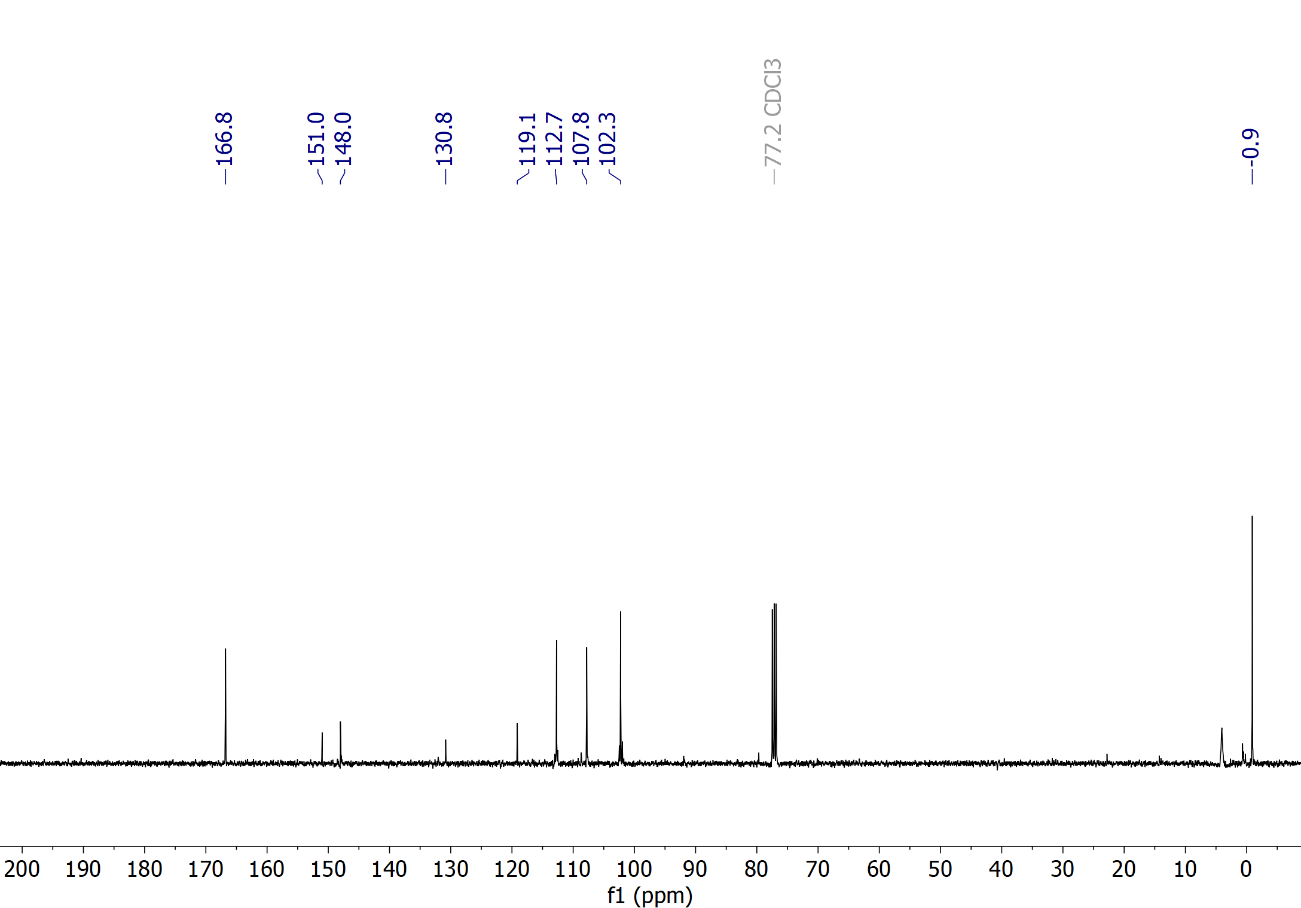

 ^1^H NMR (400 MHz, CDCl_3_)


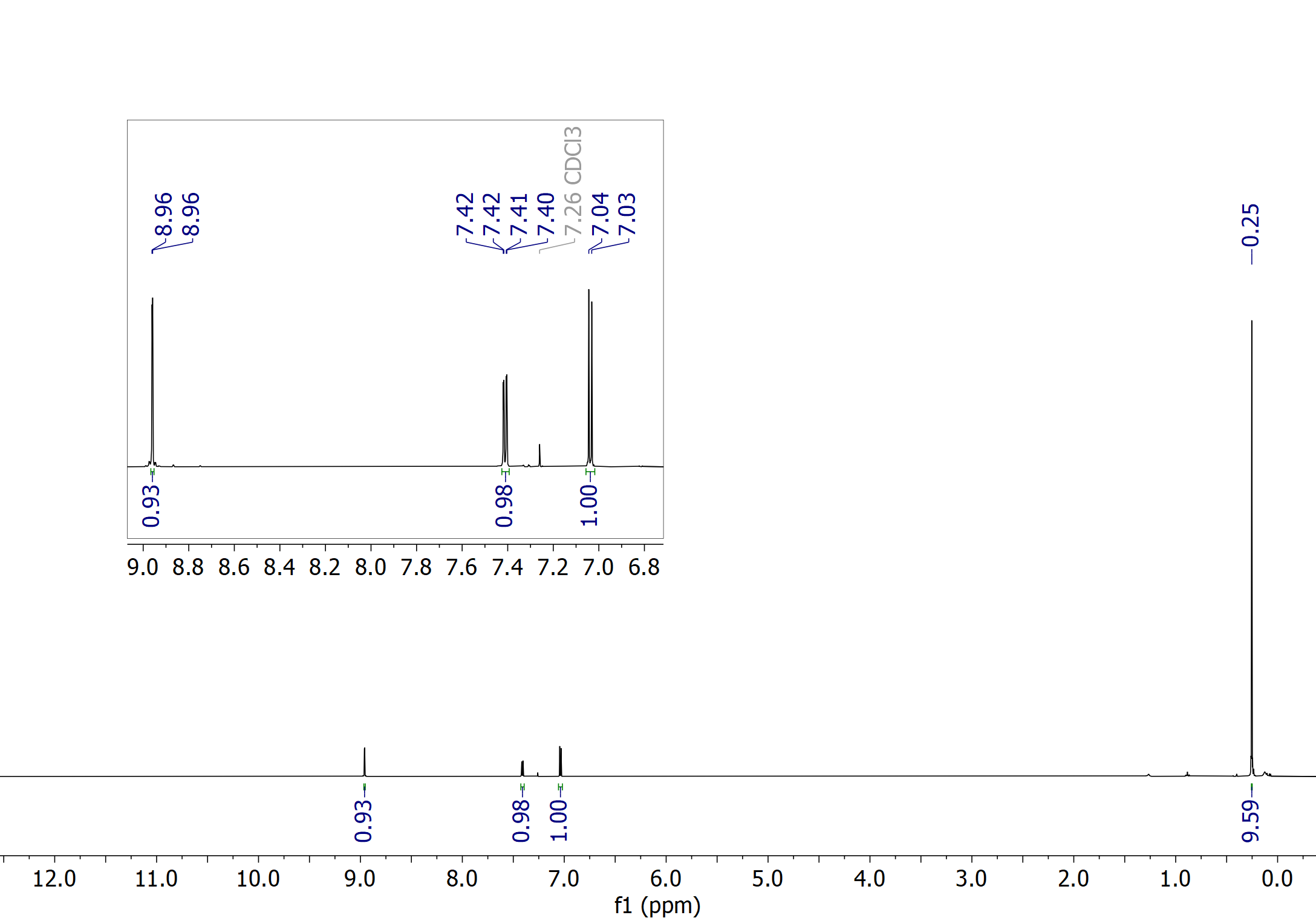

 ^13^C NMR (100.5 MHz, CDCl_3_)


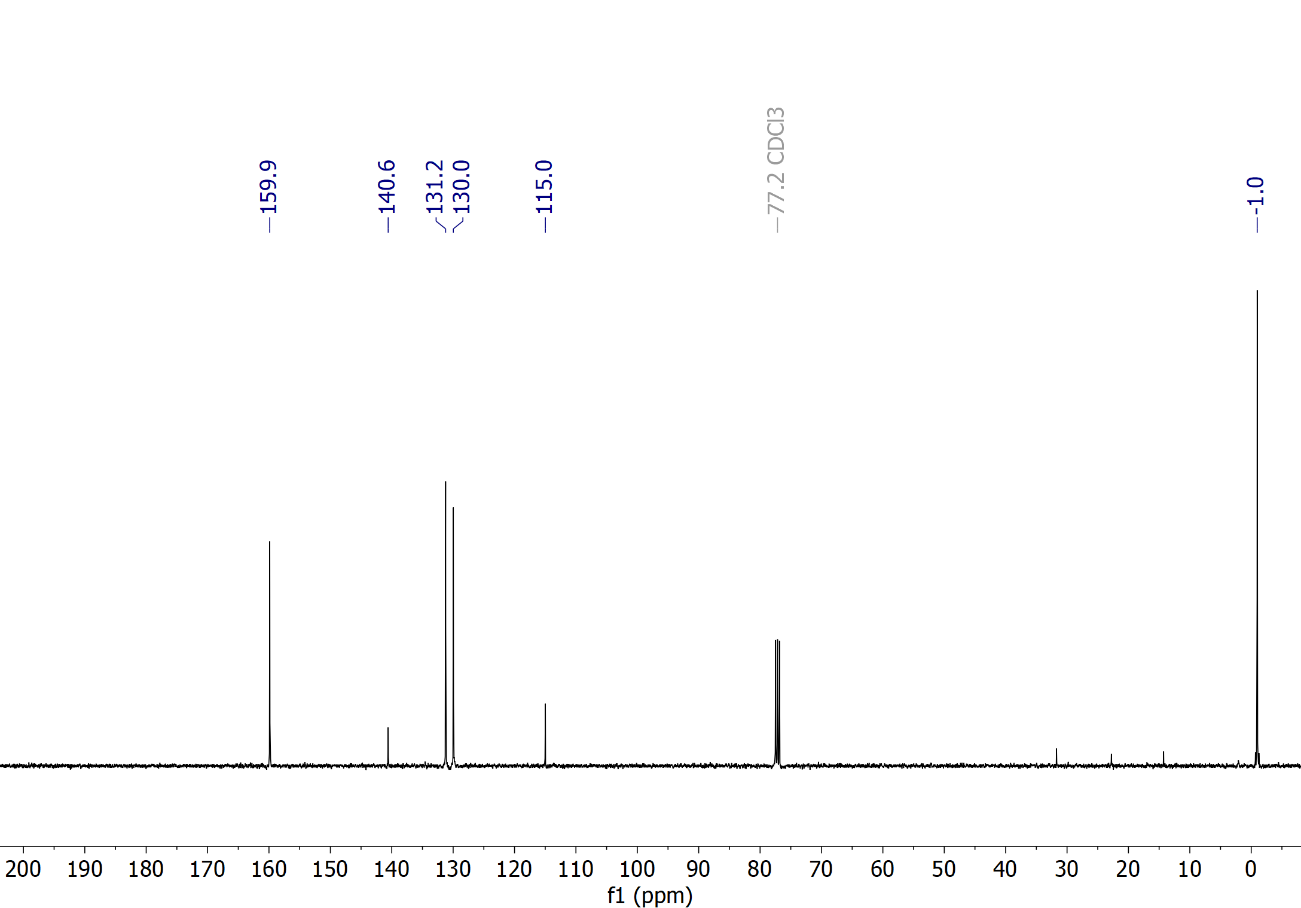

 ^1^H NMR (400 MHz, CDCl_3_)


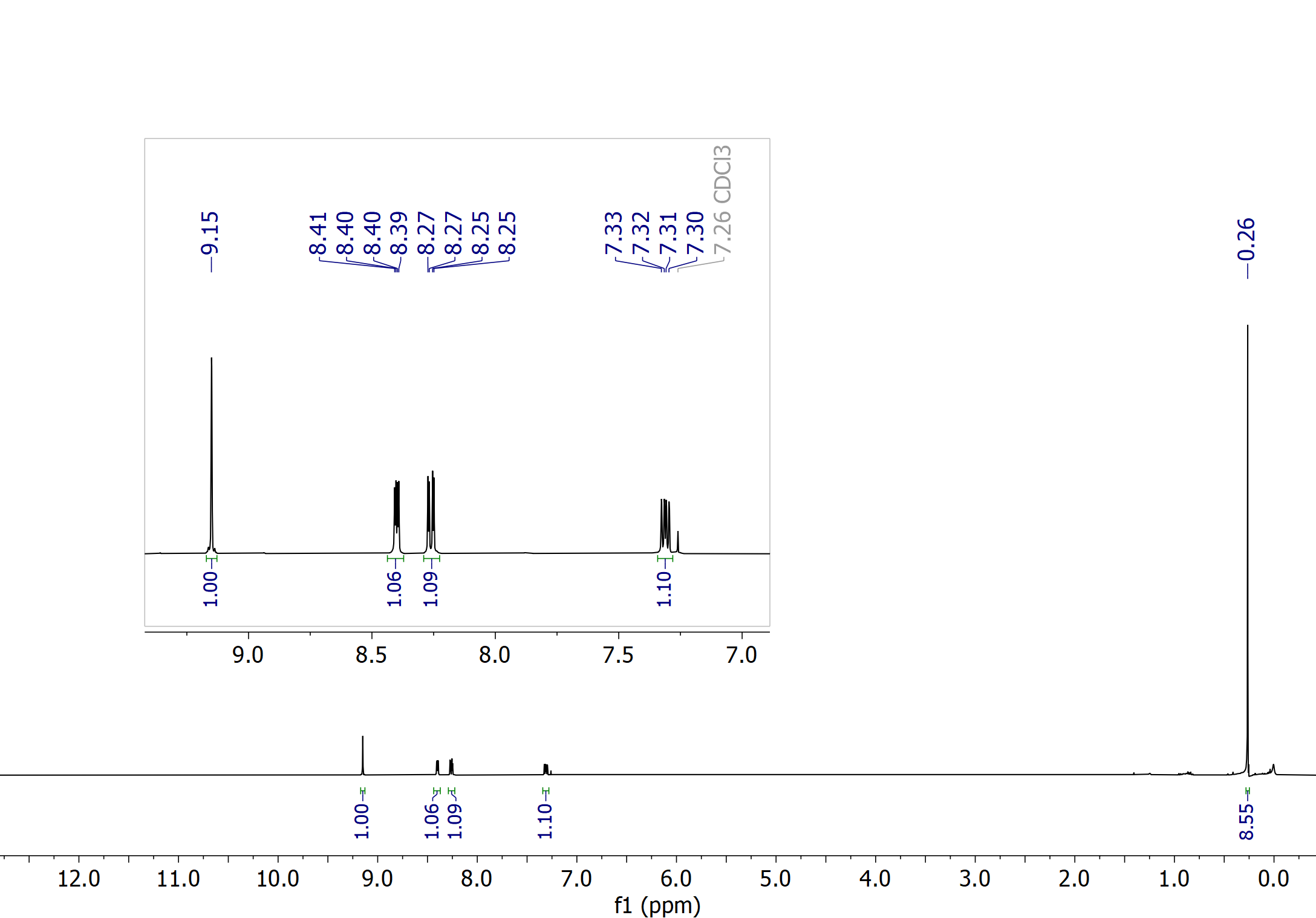

 ^13^C NMR (100.5 MHz, CDCl_3_)


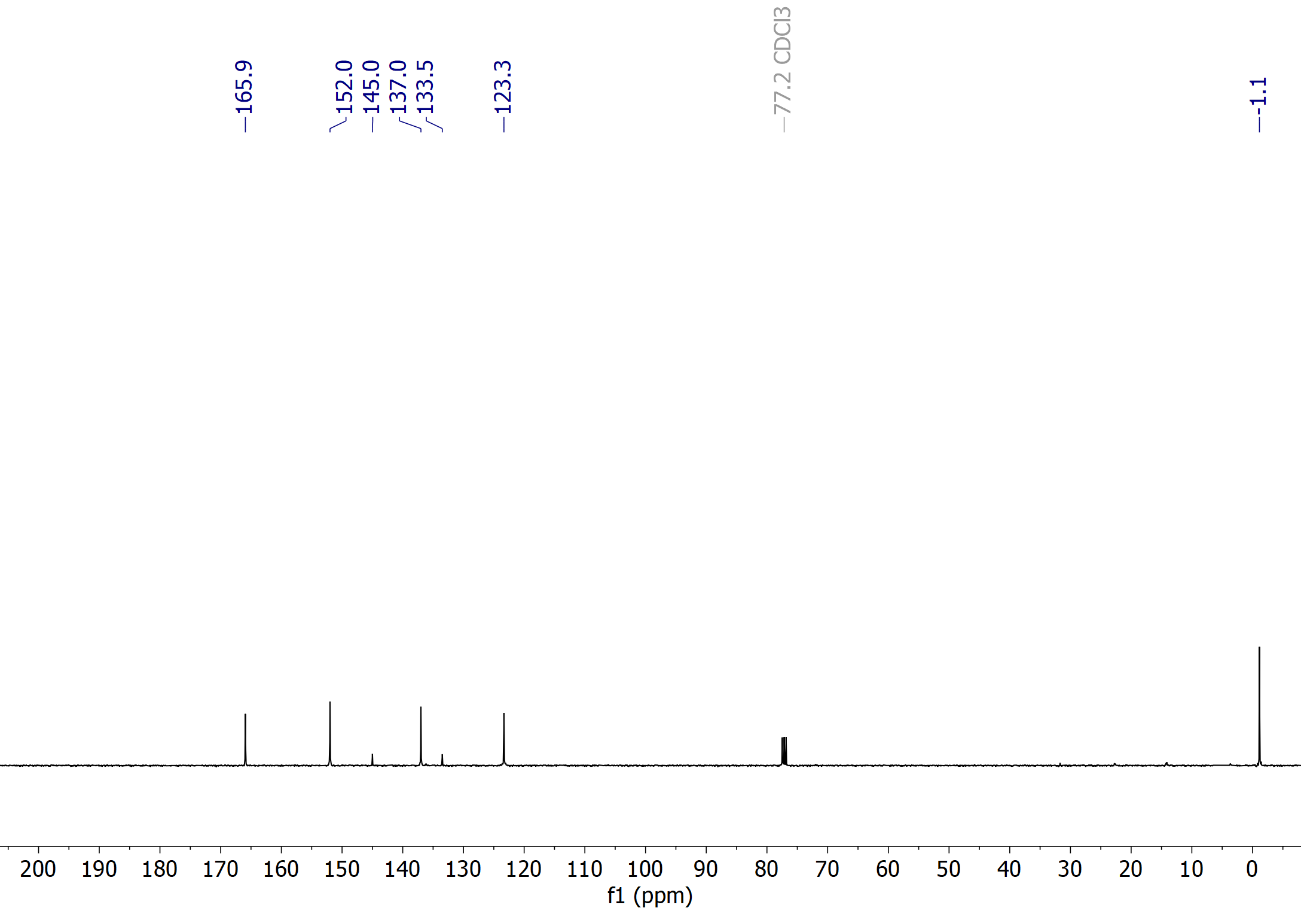

 ^1^H NMR (400 MHz, CDCl_3_)


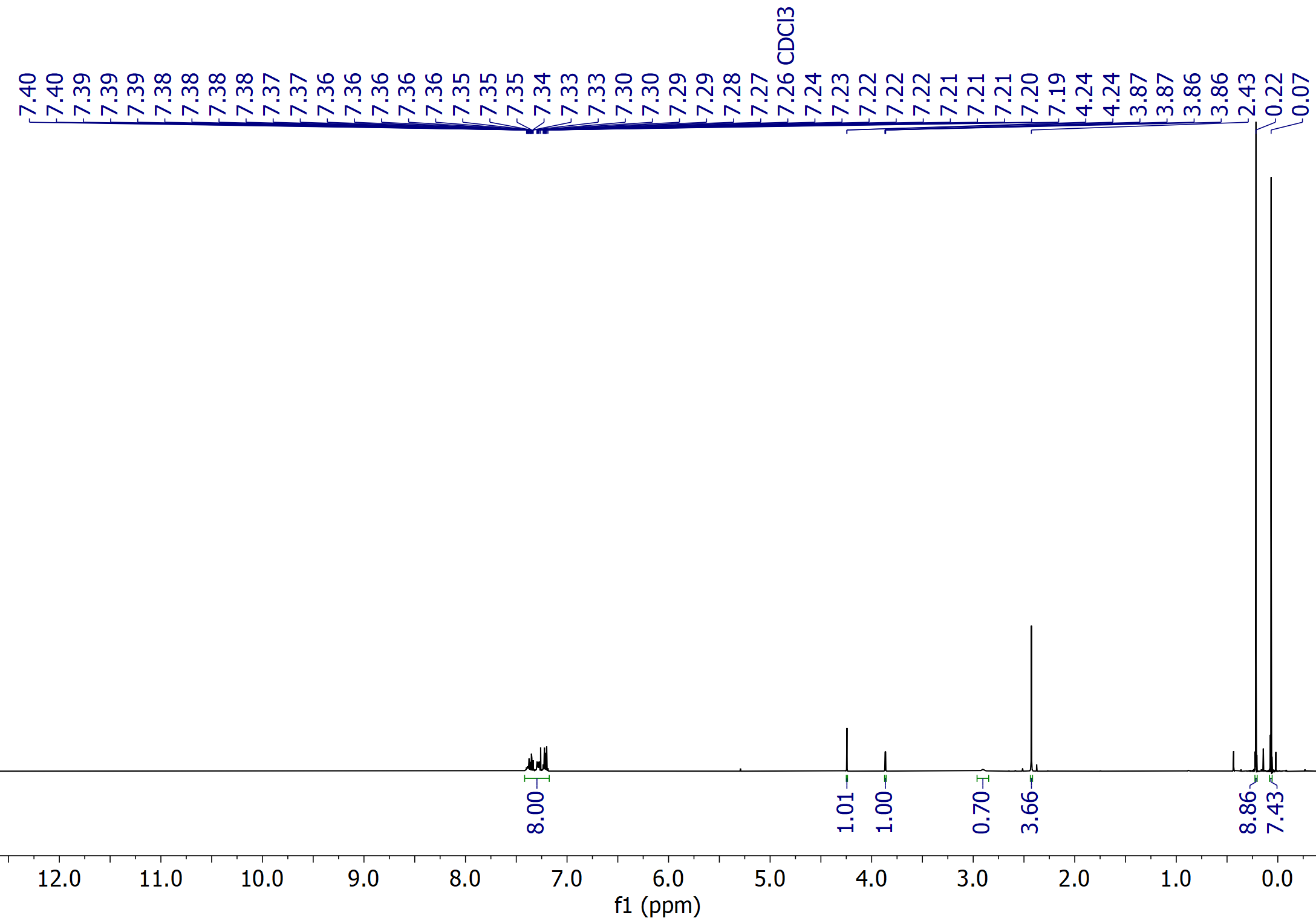

 ^13^C NMR (100.5 MHz, CDCl_3_)


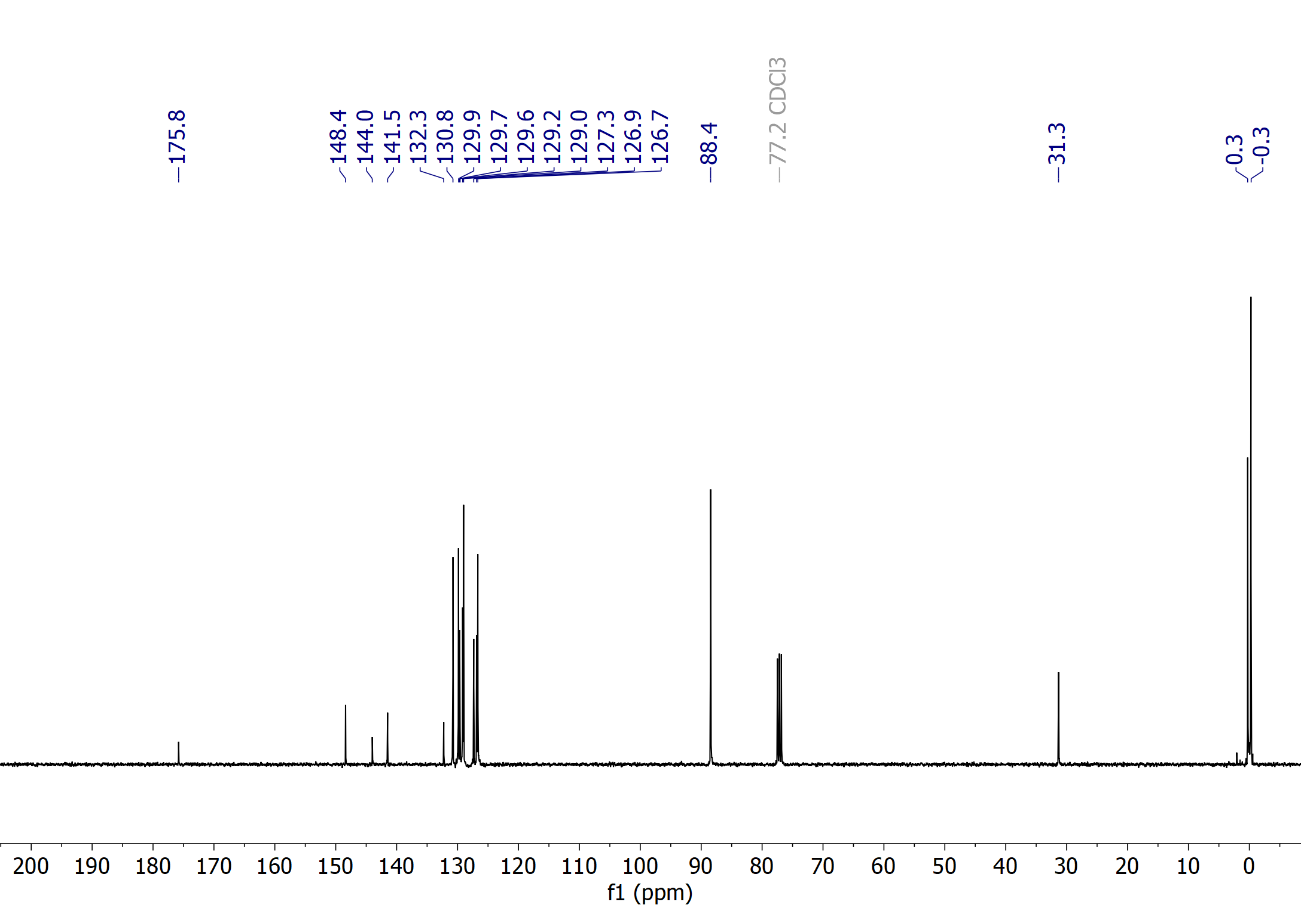

 ^1^H NMR (400 MHz, CDCl_3_)


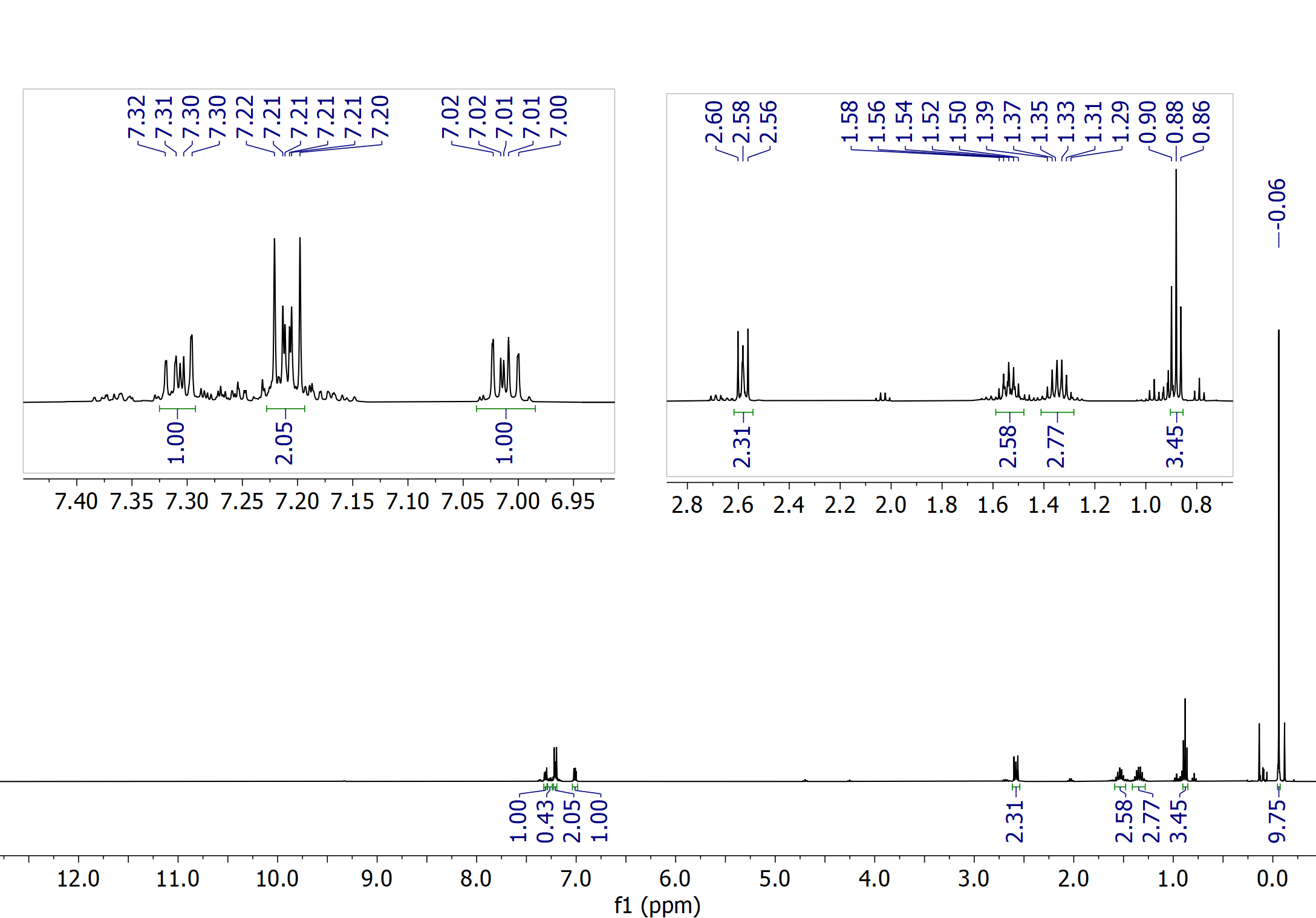

 ^13^C NMR (100.5 MHz, CDCl_3_)


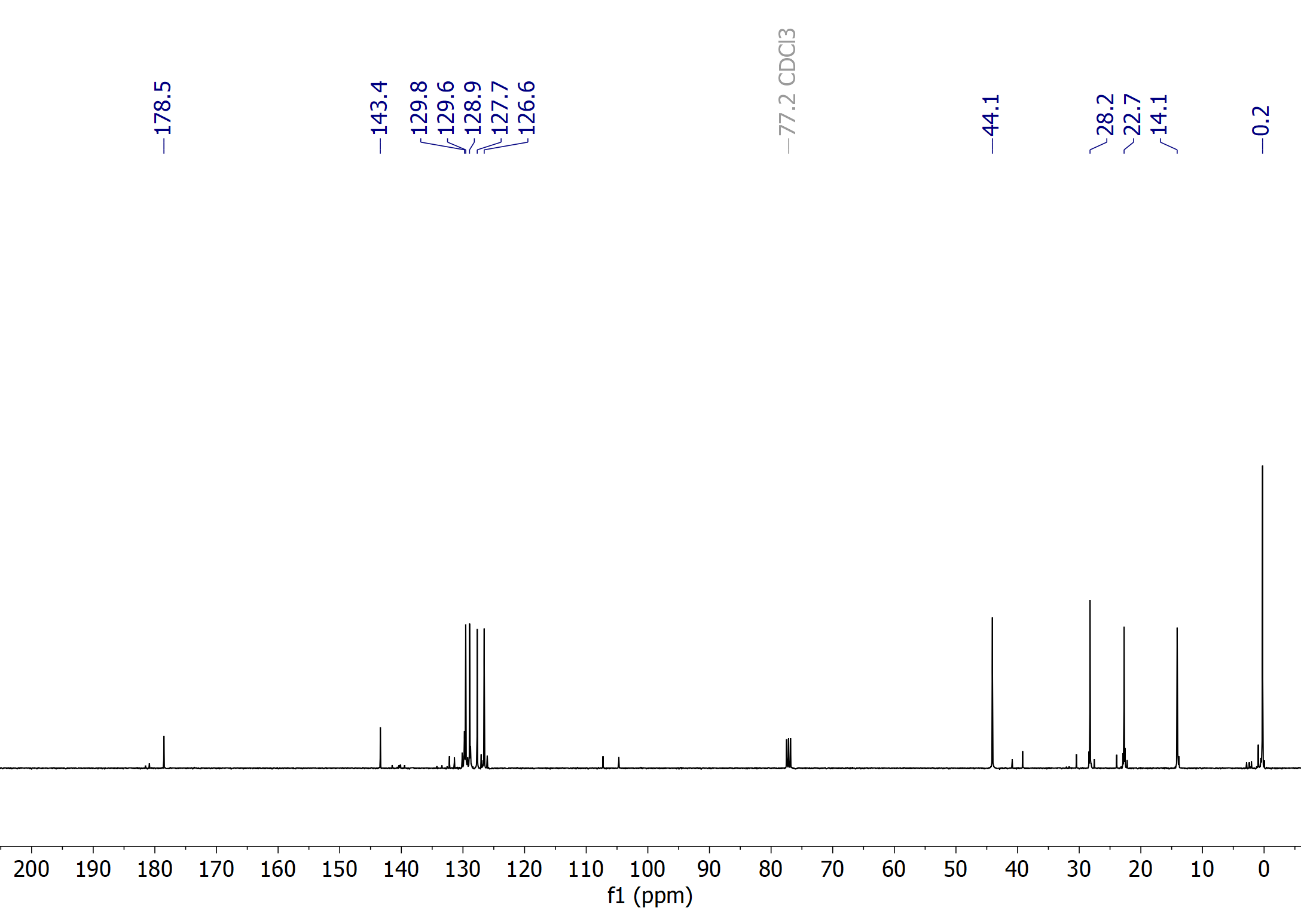

 ^1^H NMR (400 MHz, CDCl_3_)


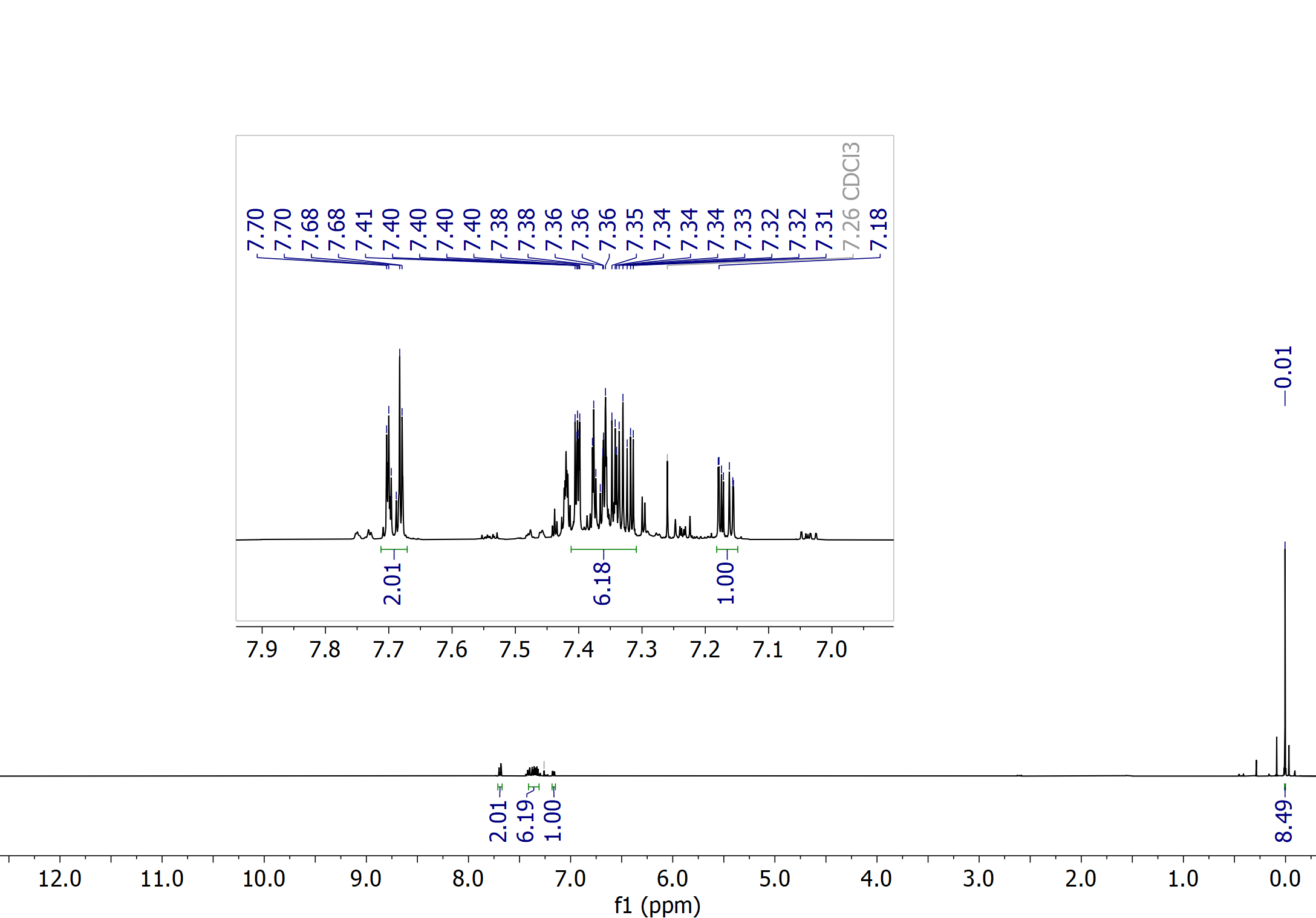


^13^C NMR (100.5 MHz, CDCl_3_)


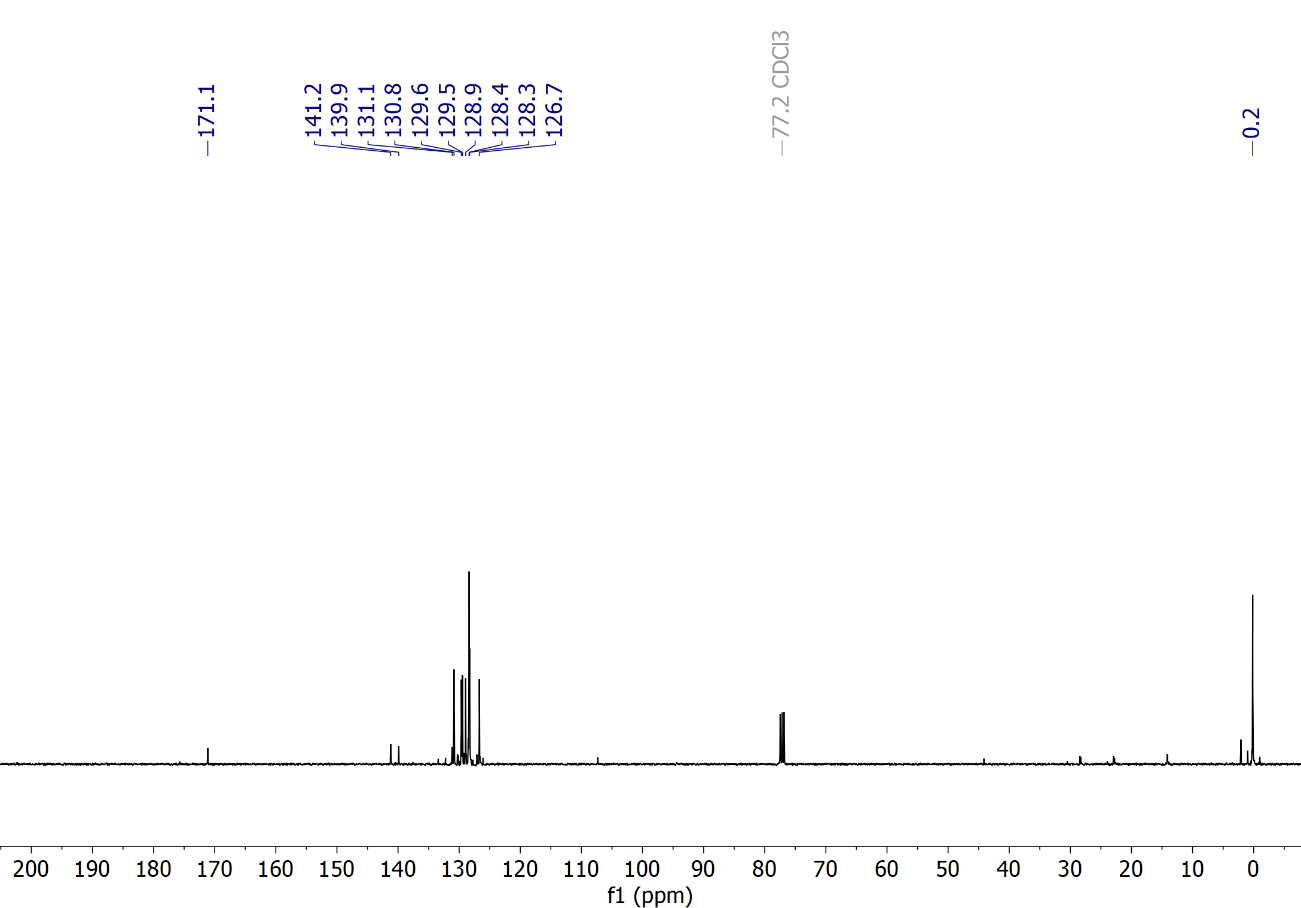


^1^H NMR (400 MHz, CDCl_3_)


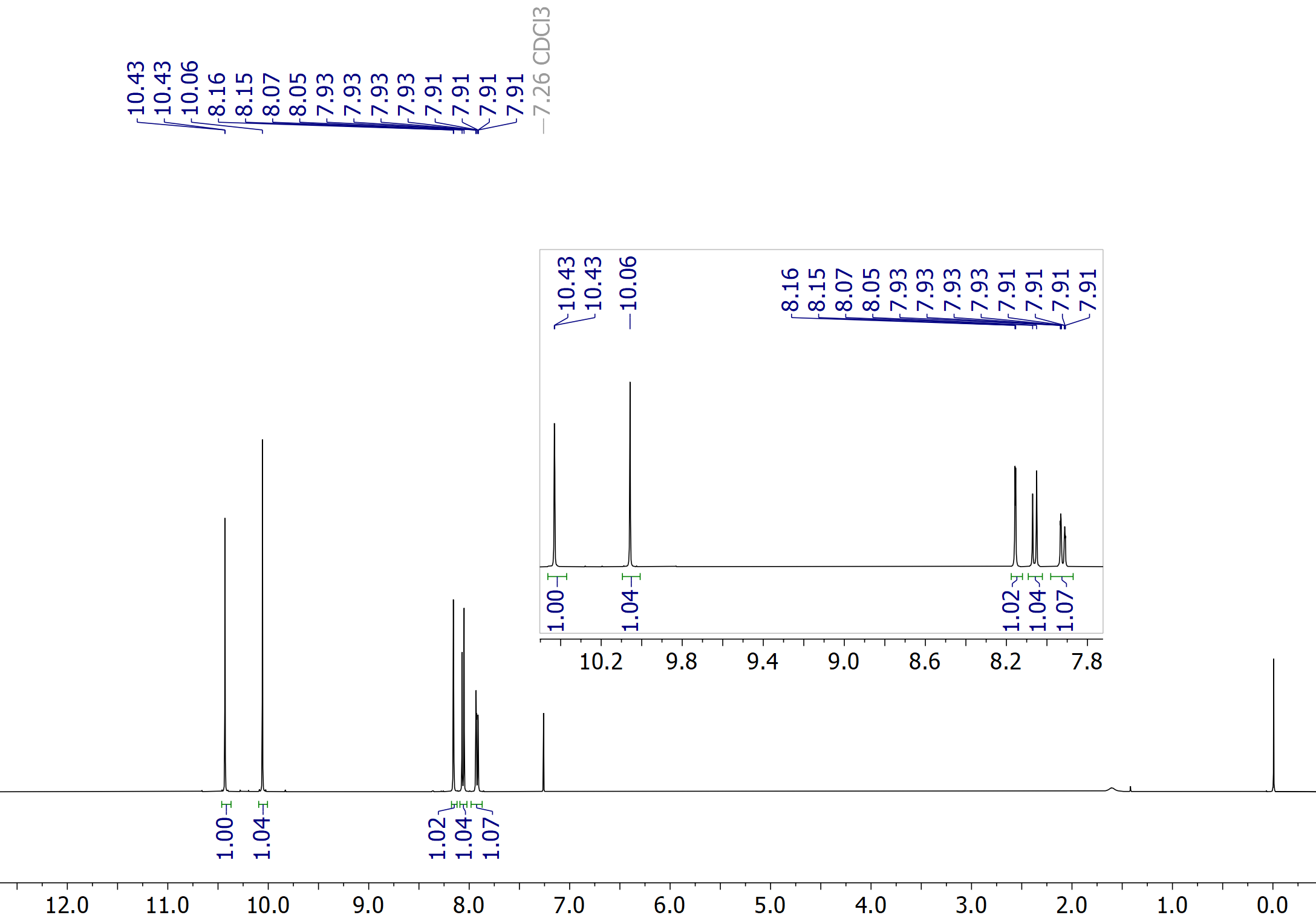


^13^C NMR (100.5 MHz, CDCl_3_)


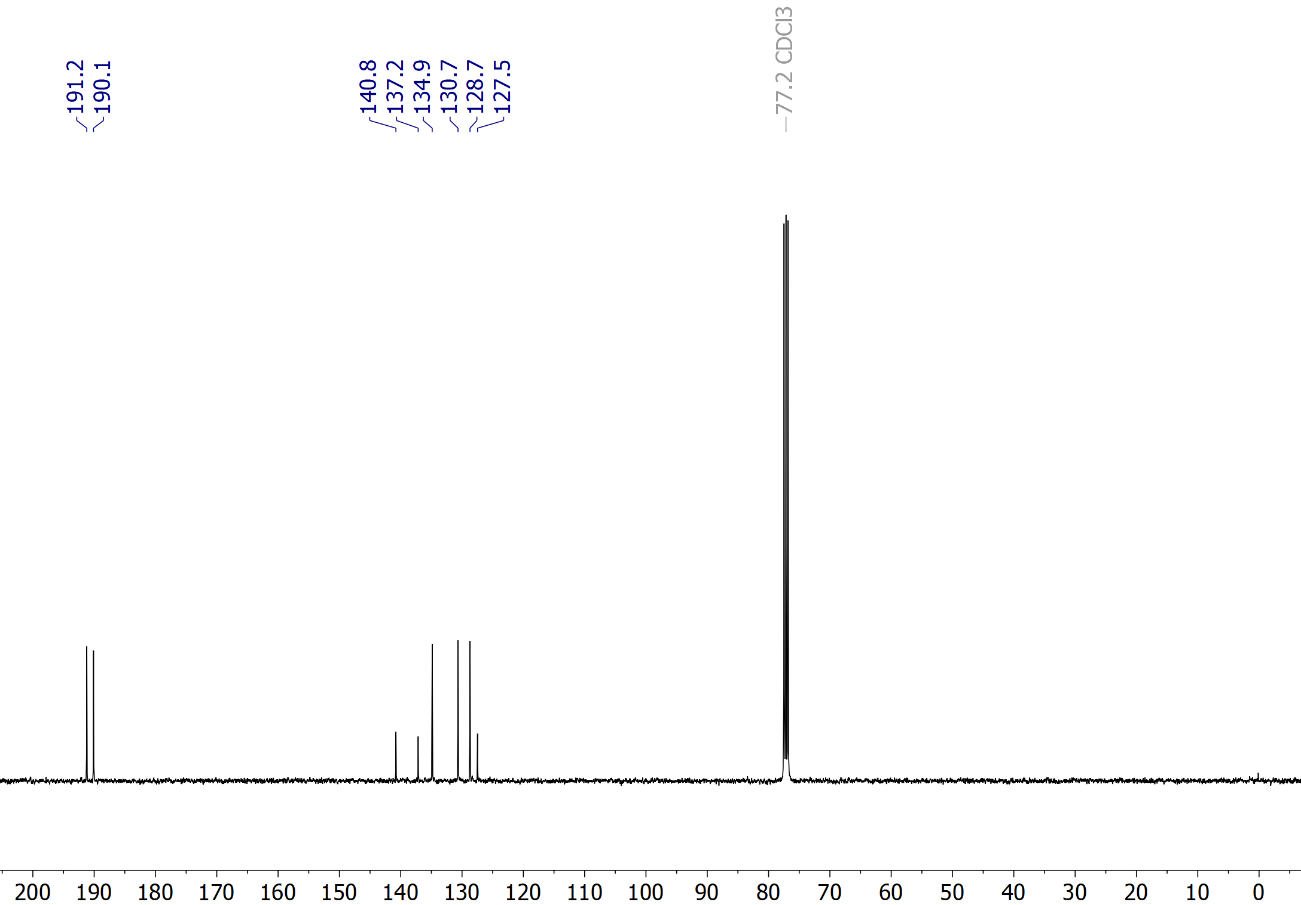


(**S1**) ^1^H NMR (400 MHz, CDCl_3_)


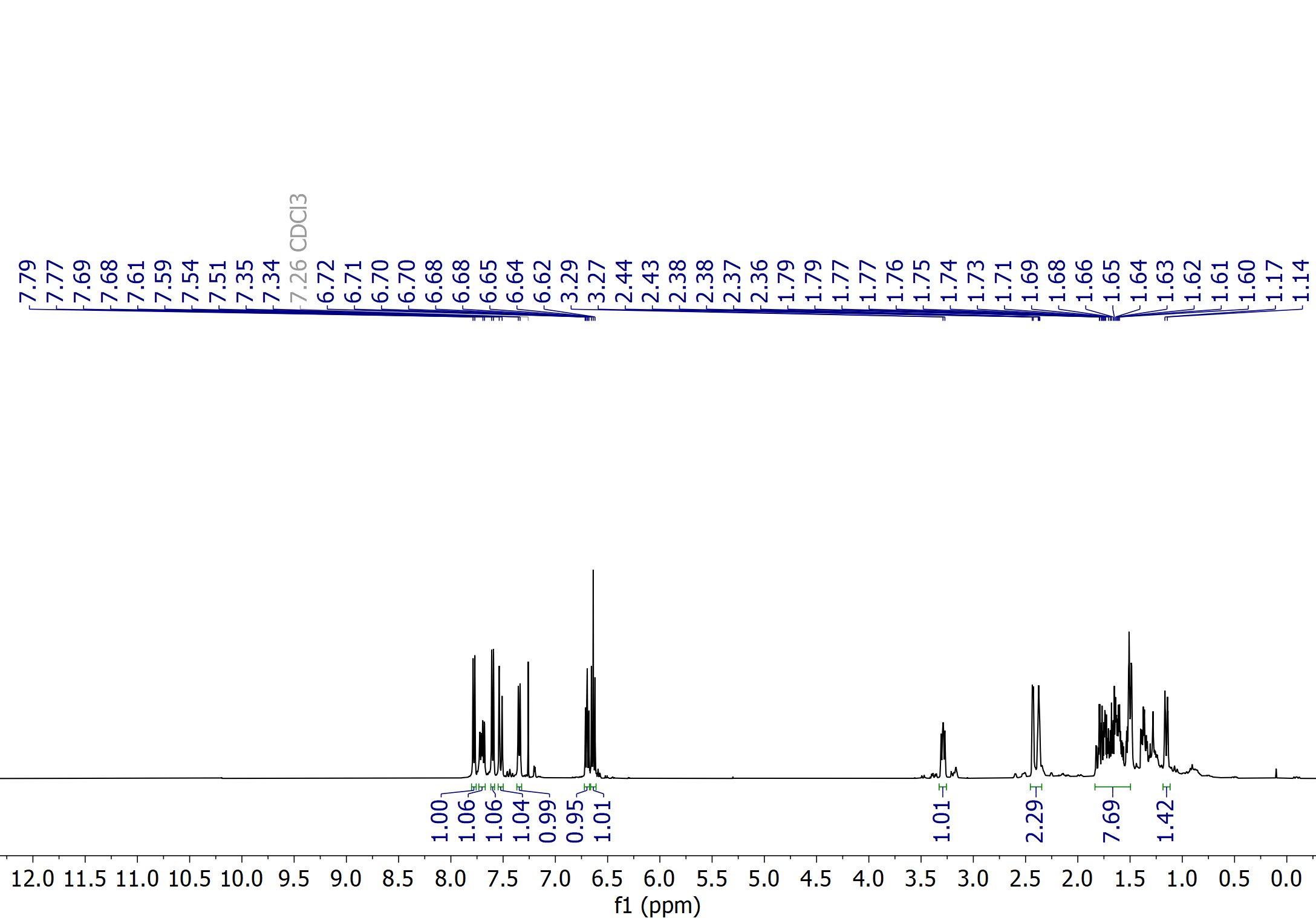


(**S1**) ^13^C NMR (100.5 MHz, CDCl_3_)
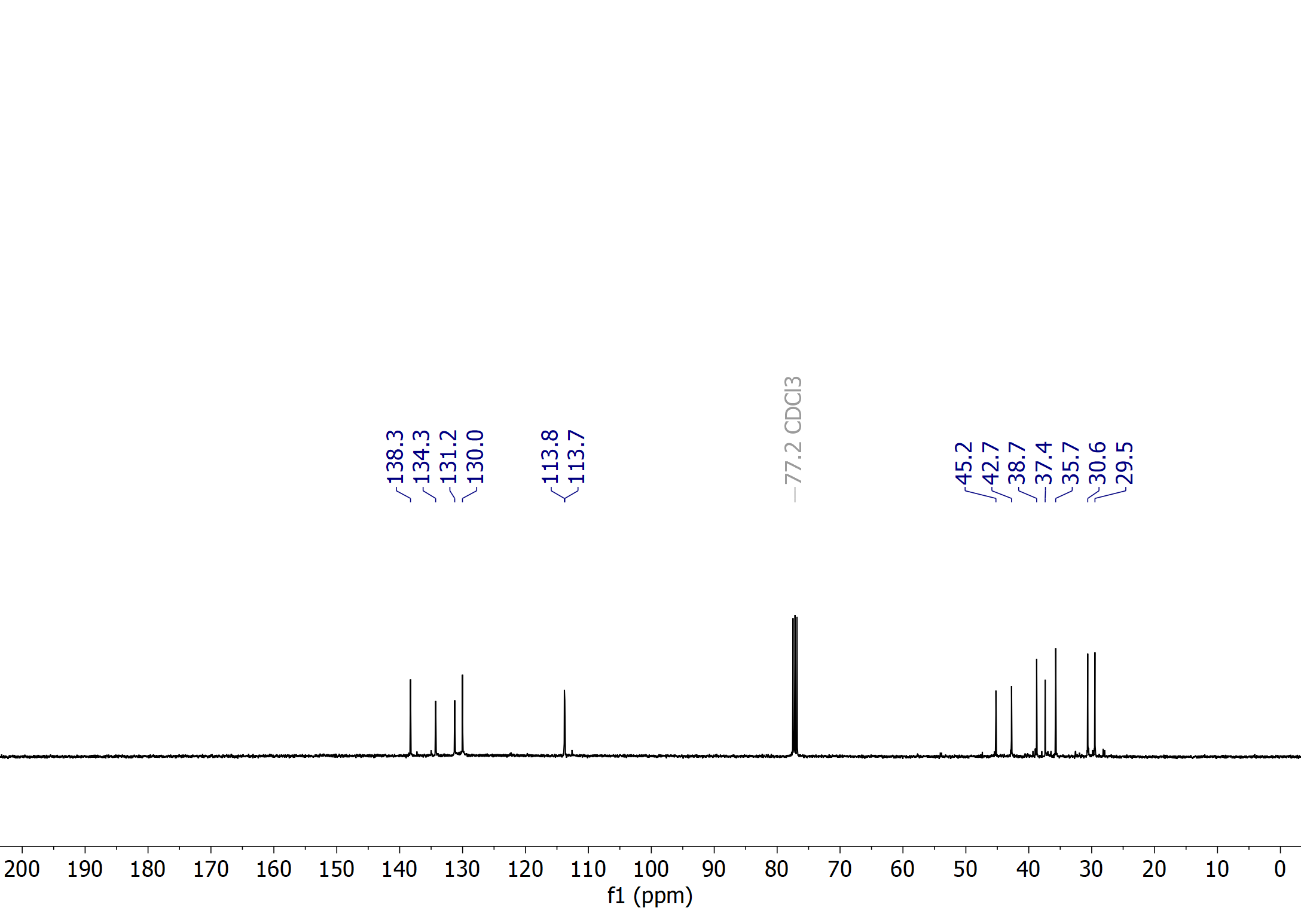


^11^B NMR (128 MHz, CDCl_3_)


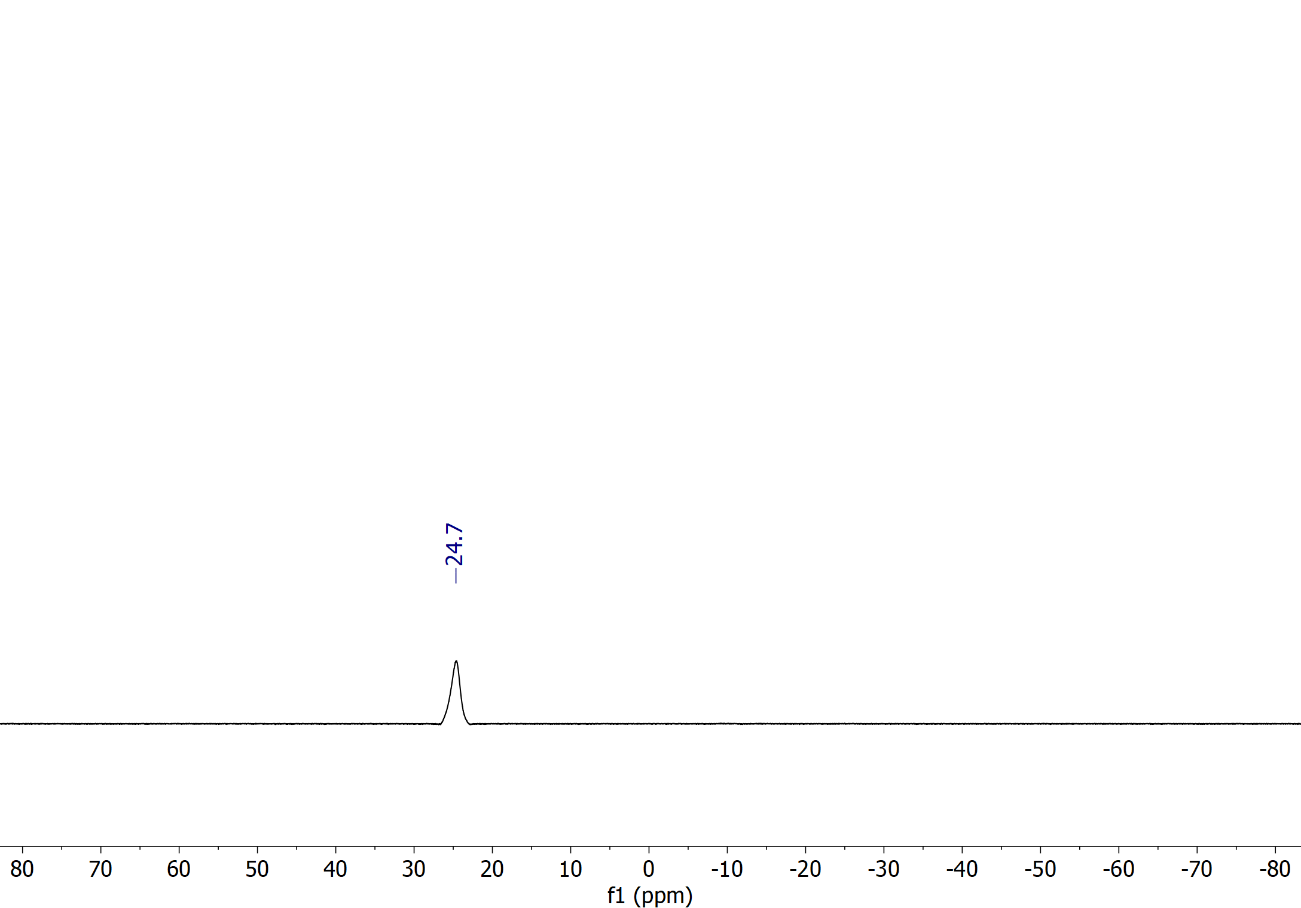


^1^H NMR (400 MHz, CDCl_3_)


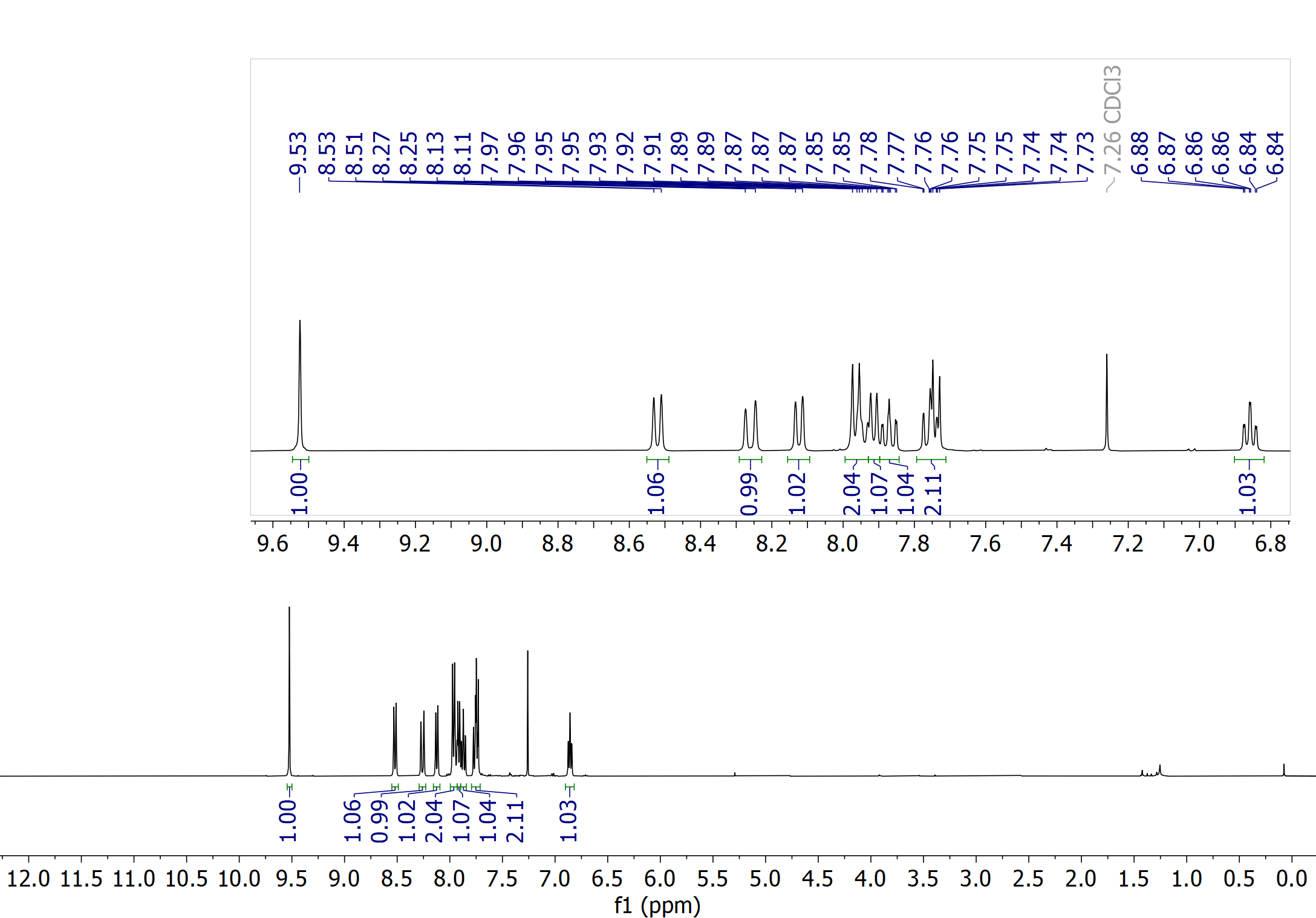


^13^C NMR (100.5 MHz, CDCl_3_)


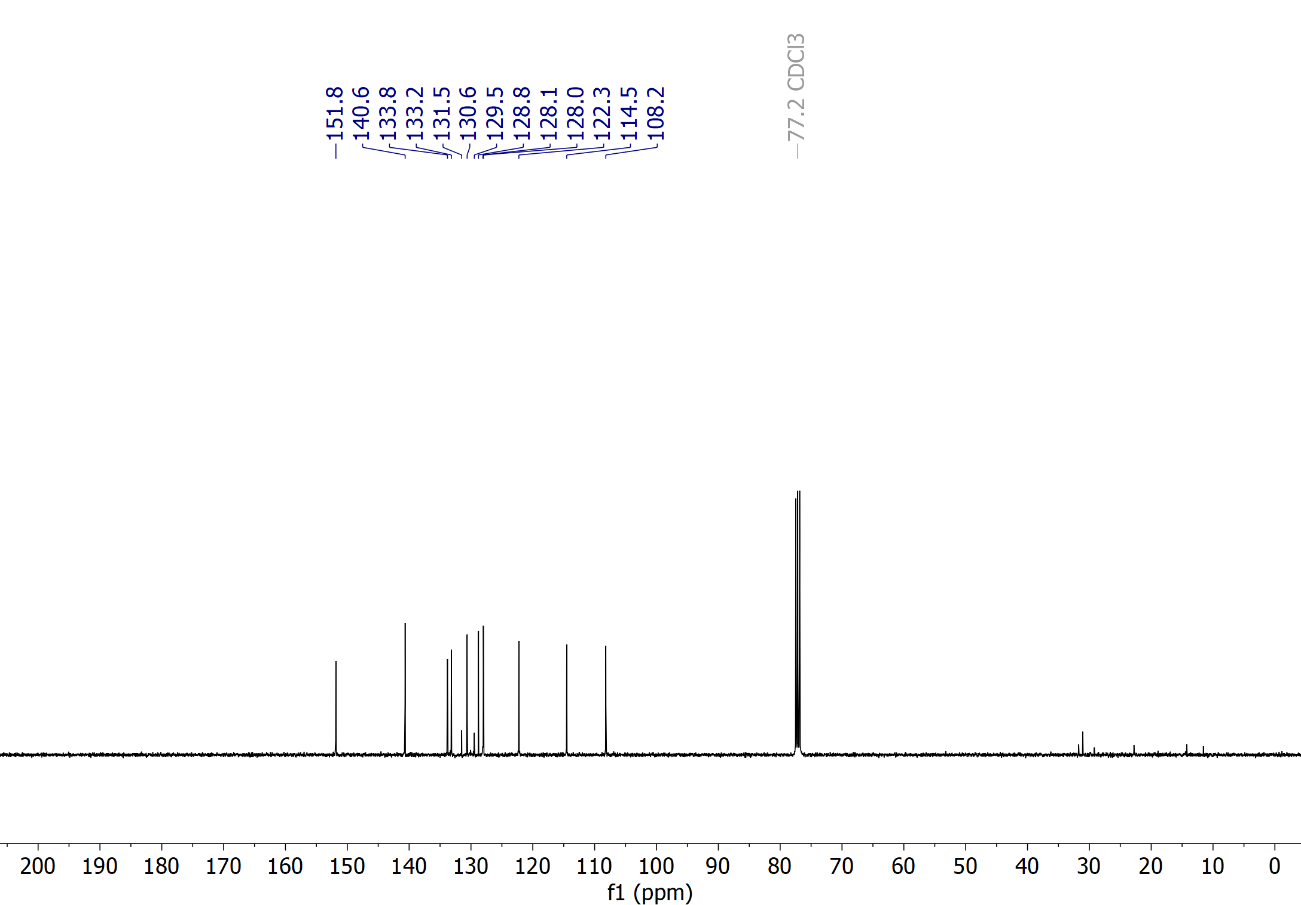


^11^B NMR (128 MHz, CDCl_3_)


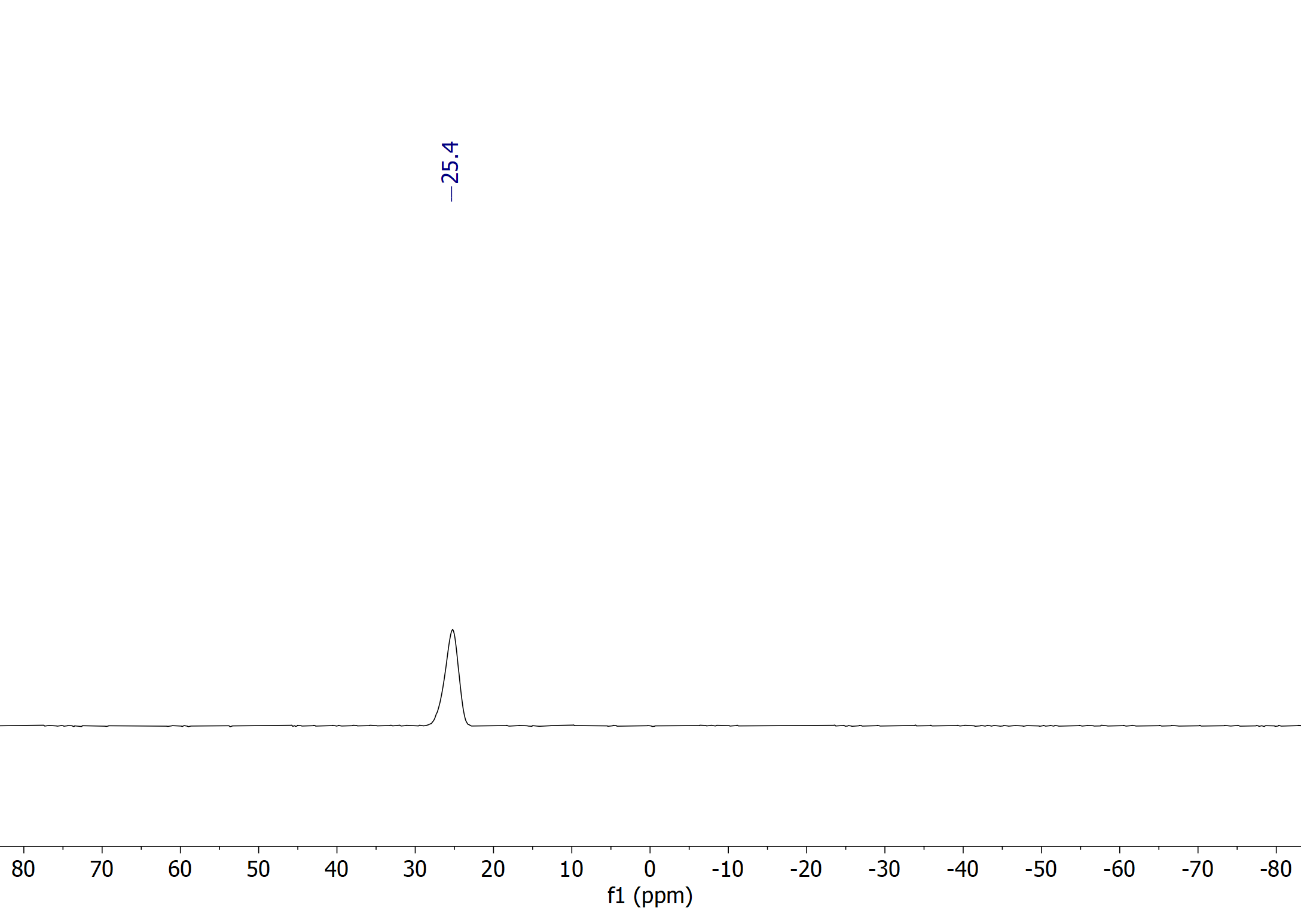


COSY (400, 400 MHz, CDCl_3_)


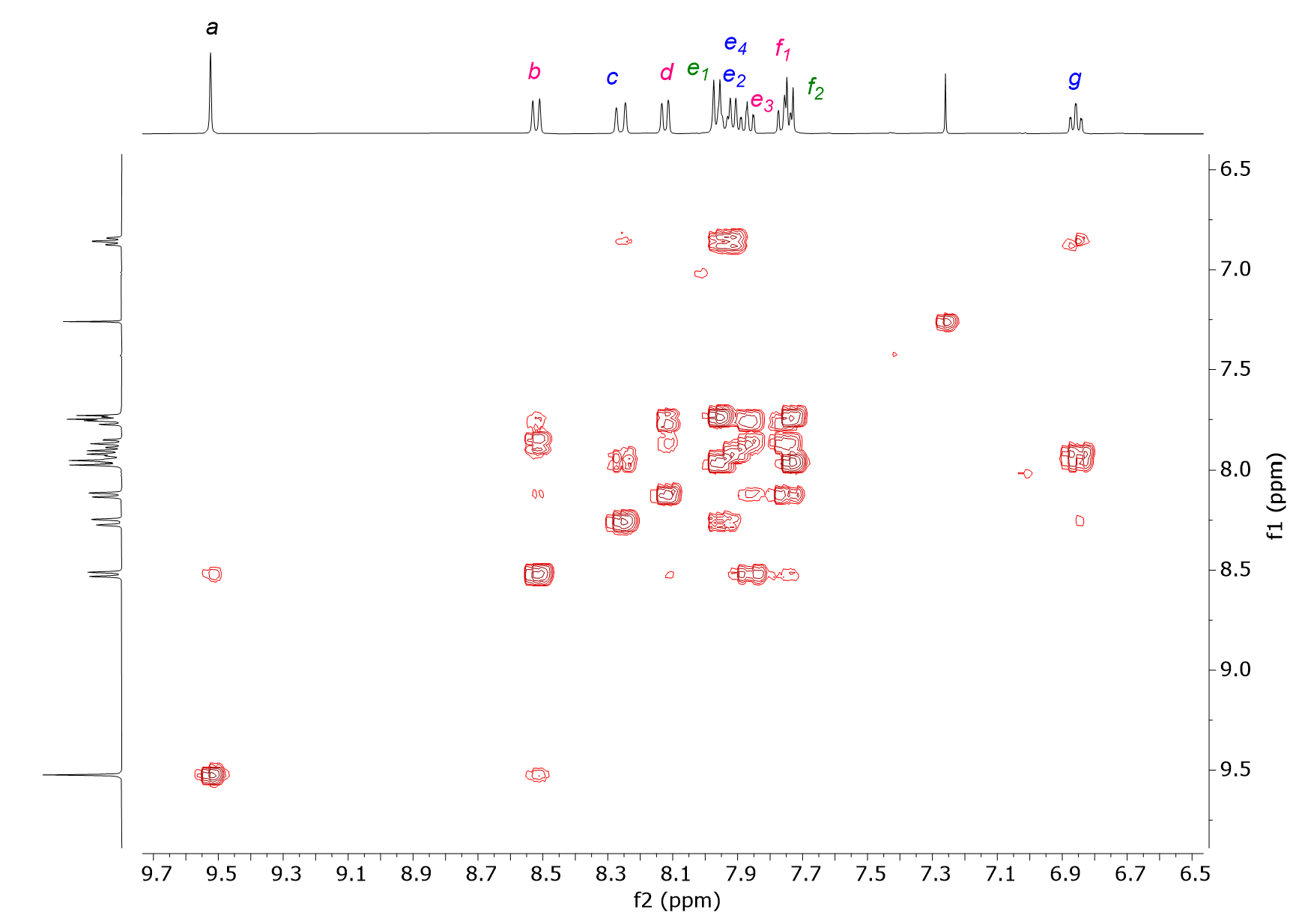


HSQC (400, 100.5 MHz, CDCl_3_)


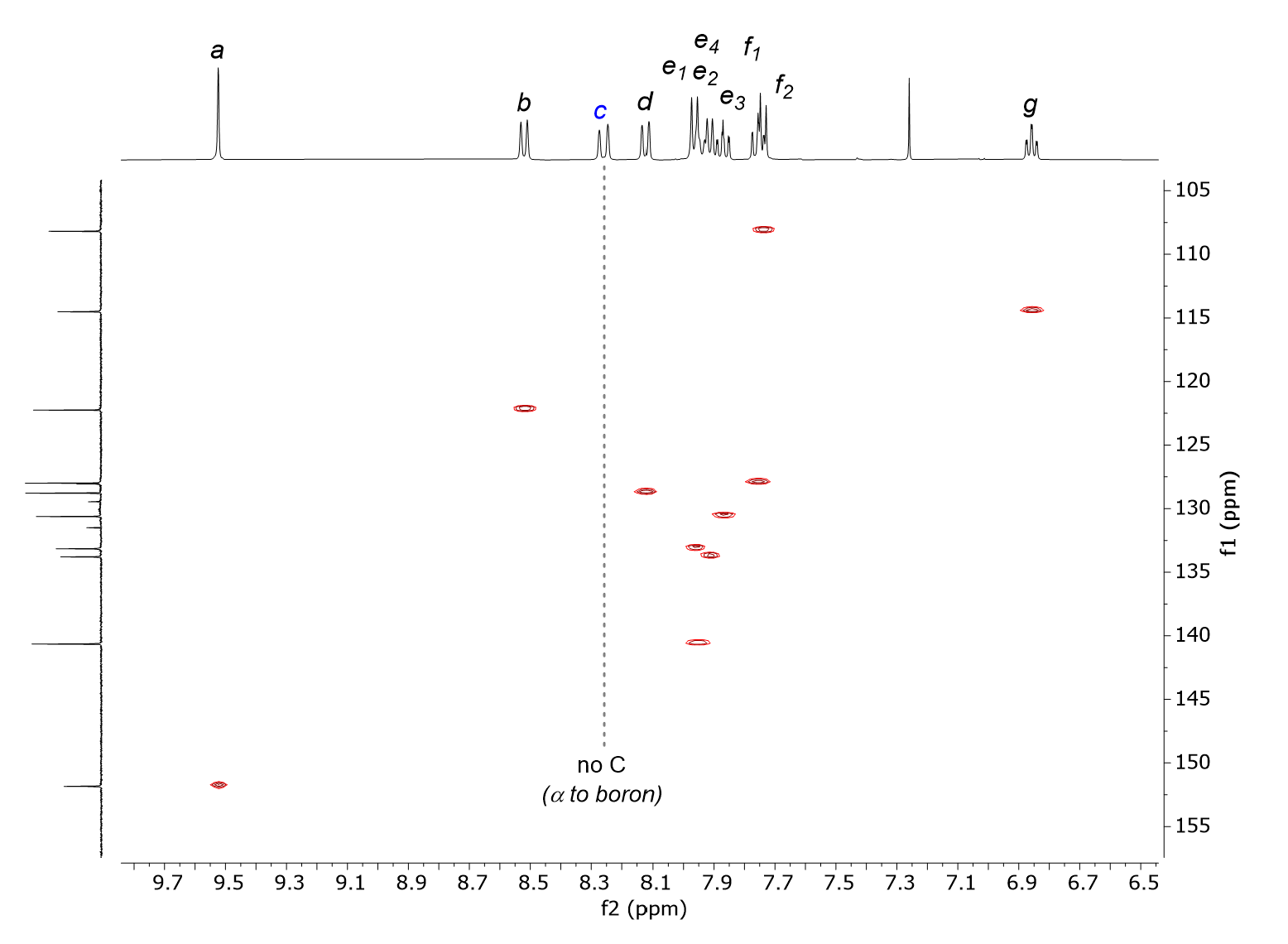


HMBC (400, 100.5 MHz, CDCl_3_)


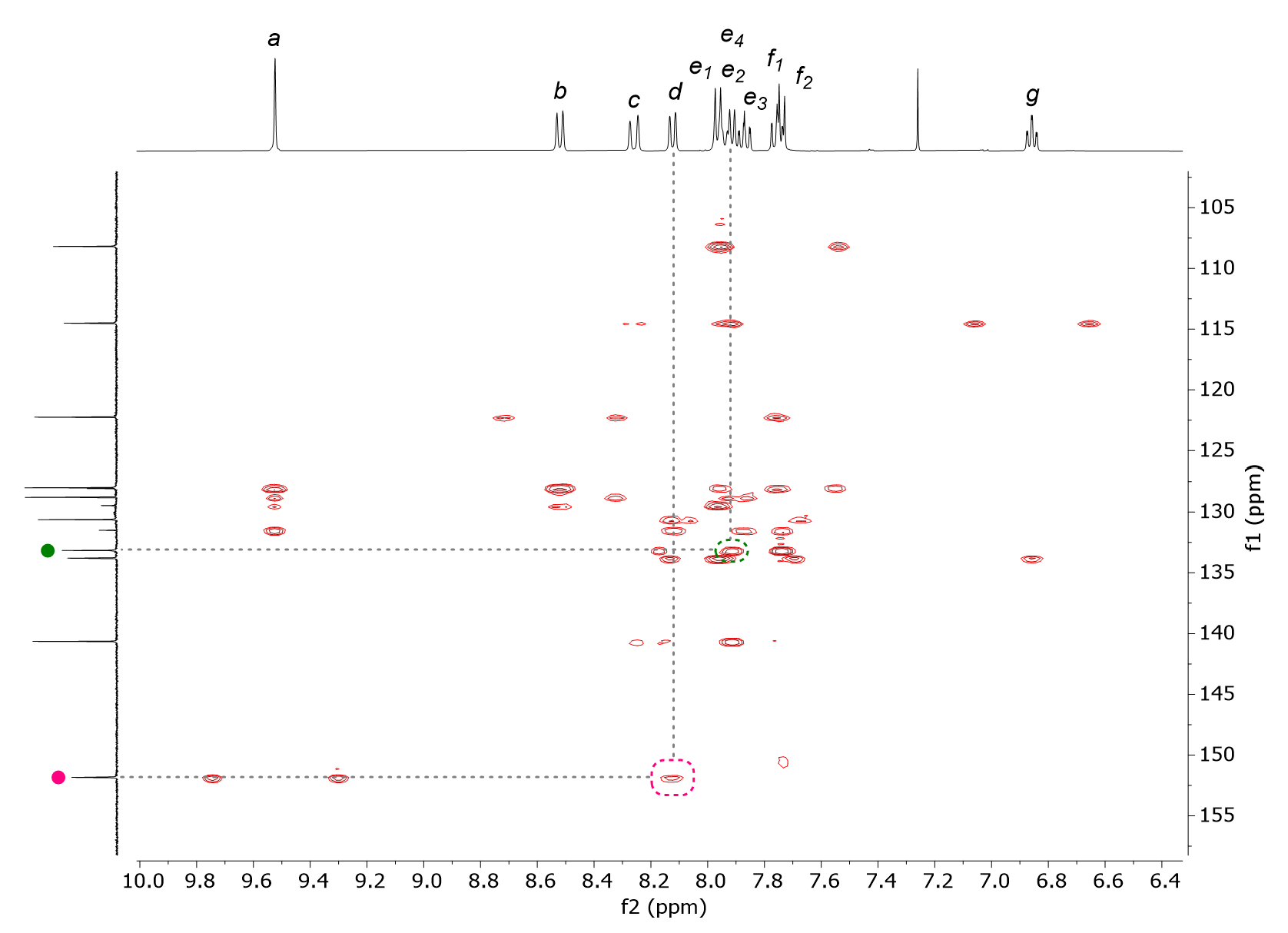


^1^H NMR (400 MHz, CDCl_3_)


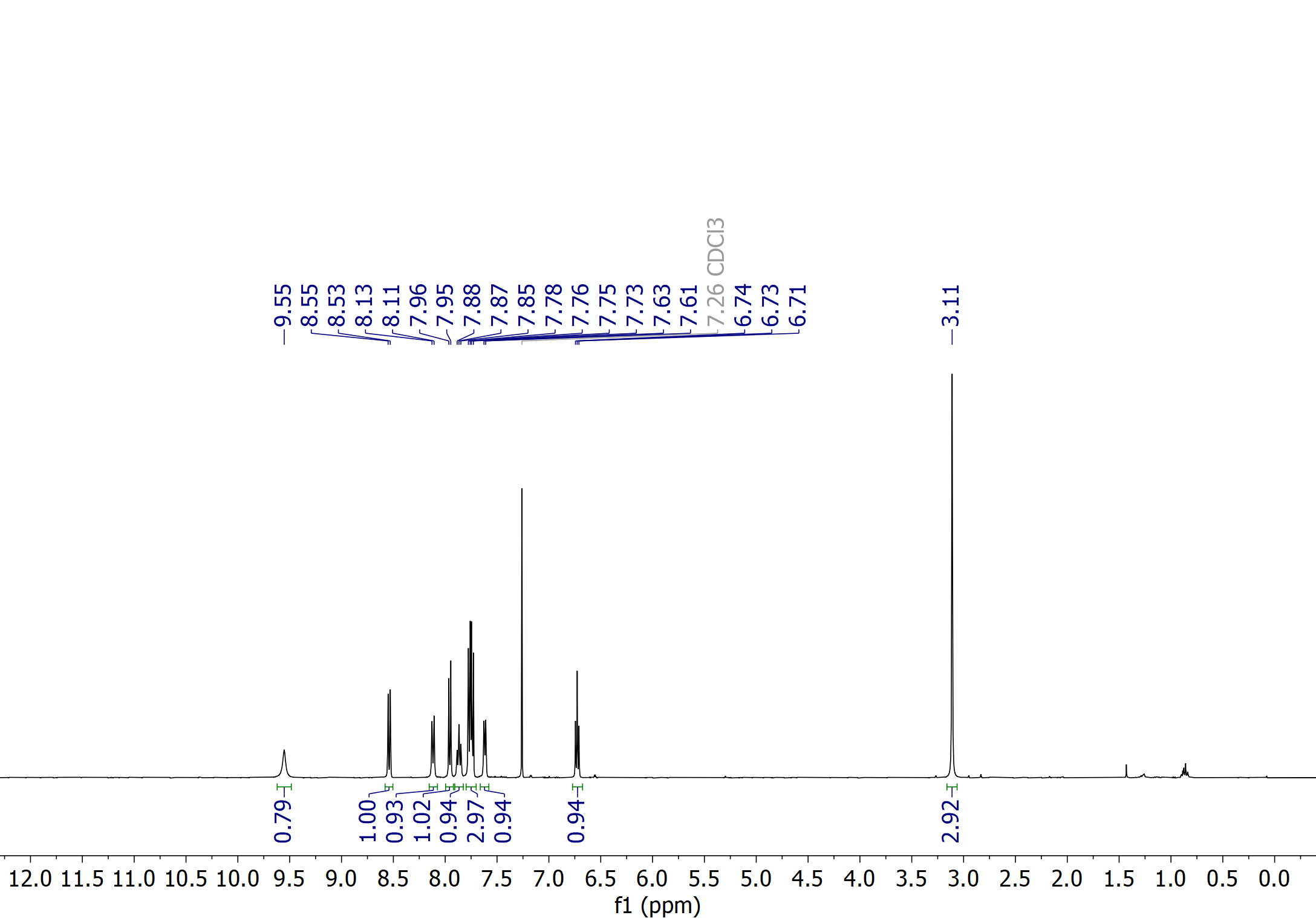


^13^C NMR (100.5 MHz, CDCl_3_)


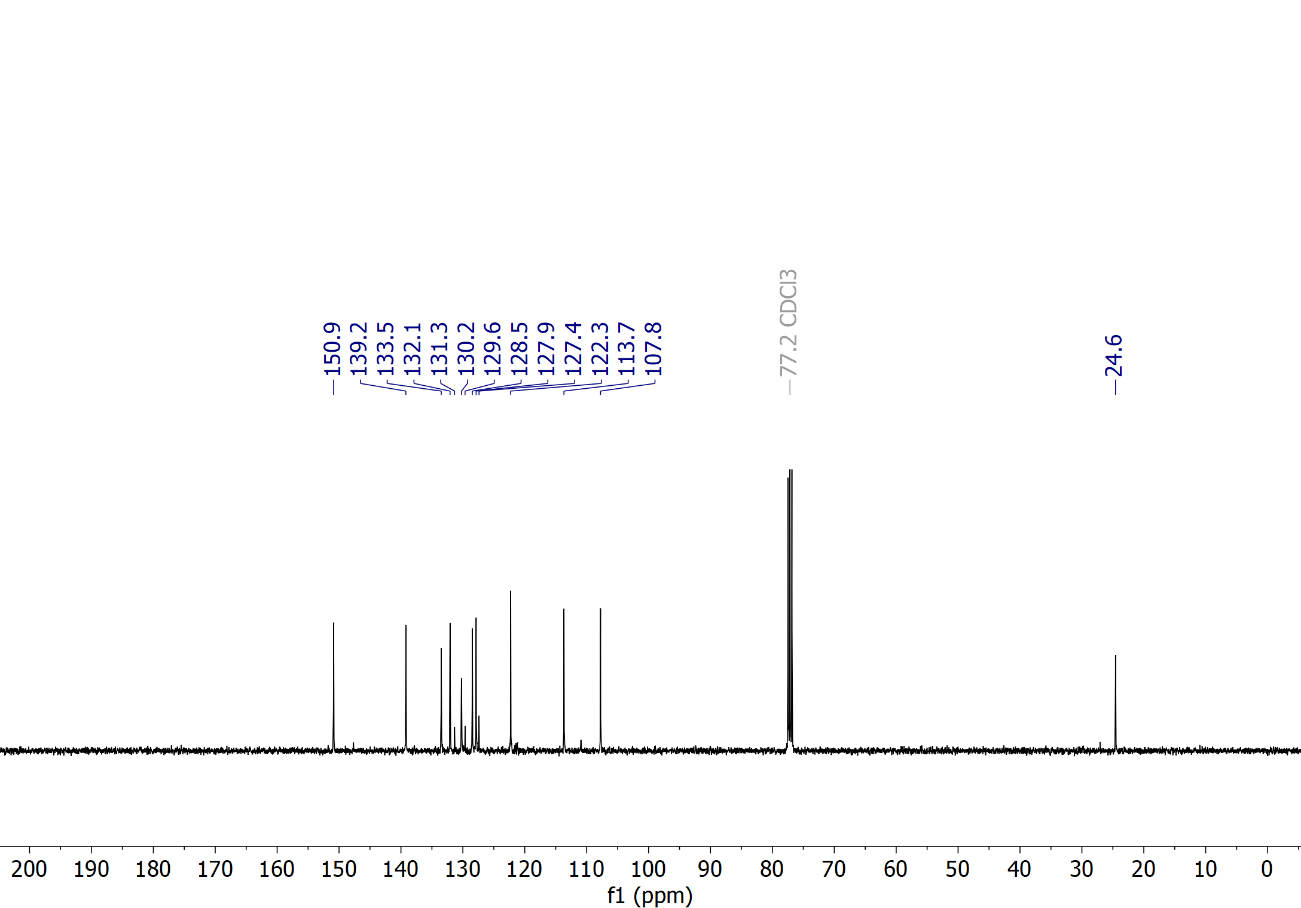


^11^B NMR (128 MHz, CDCl_3_)


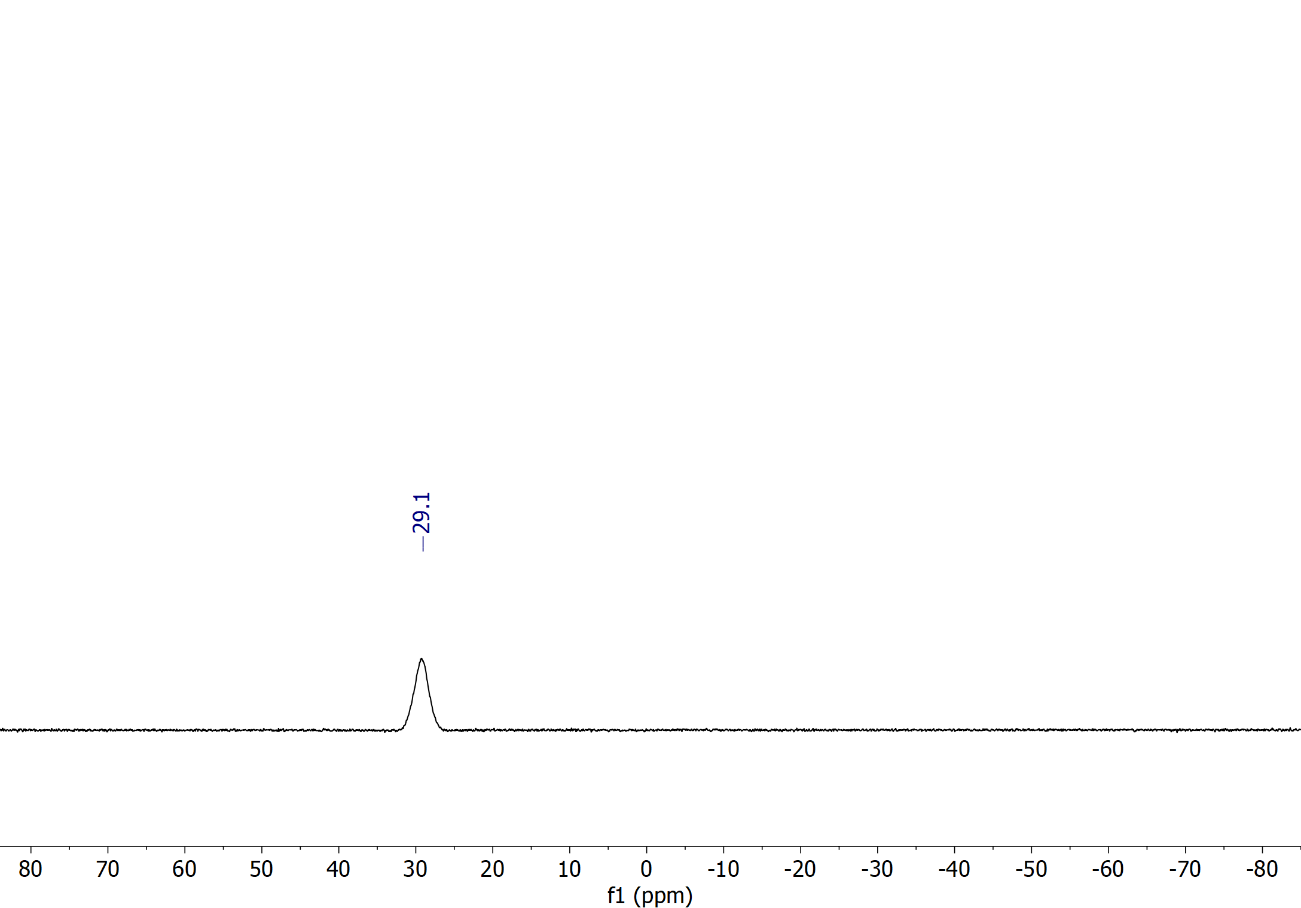


COSY (400, 400 MHz, CDCl_3_)


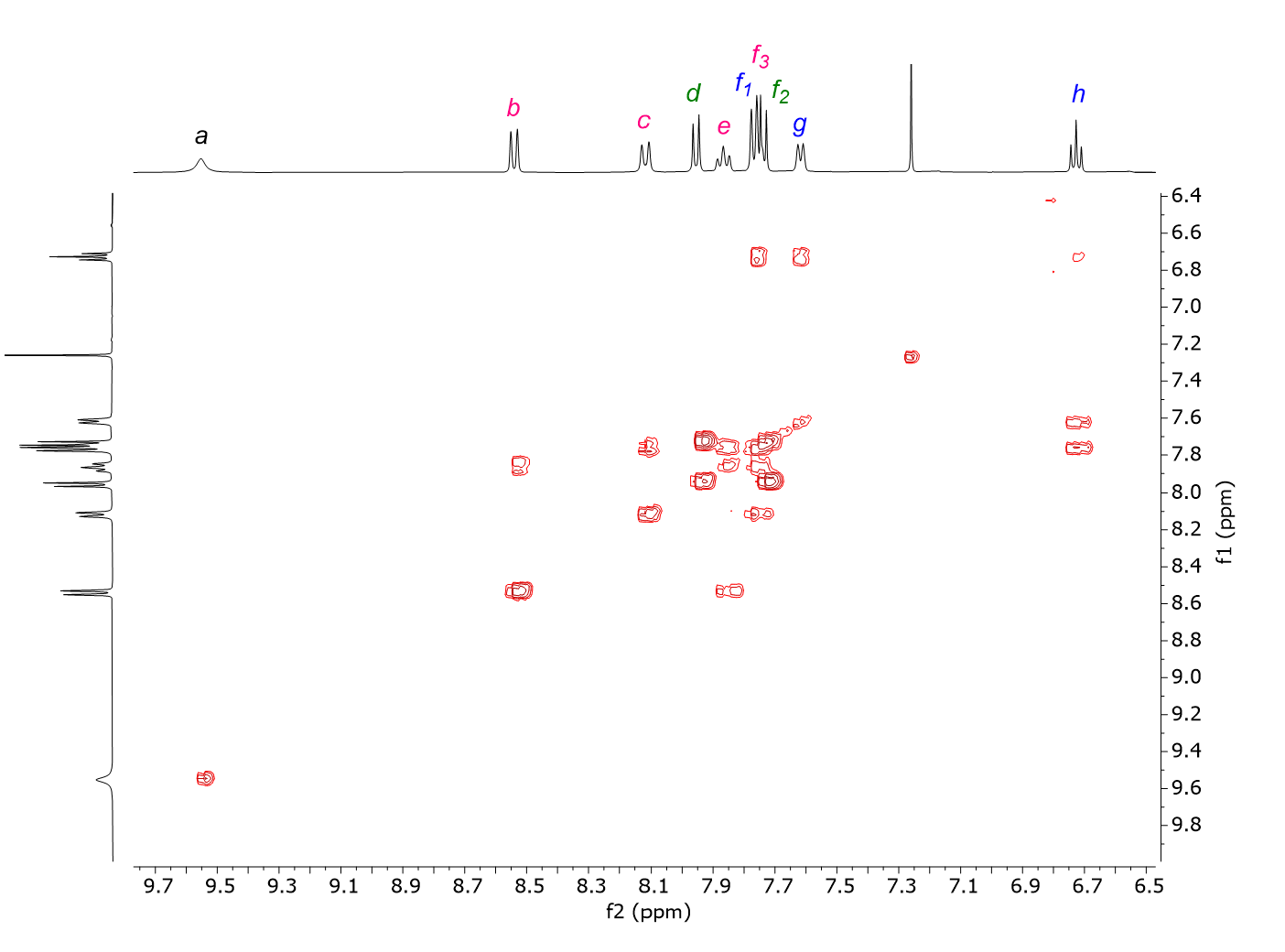


HSQC (400, 100.5 MHz, CDCl_3_)


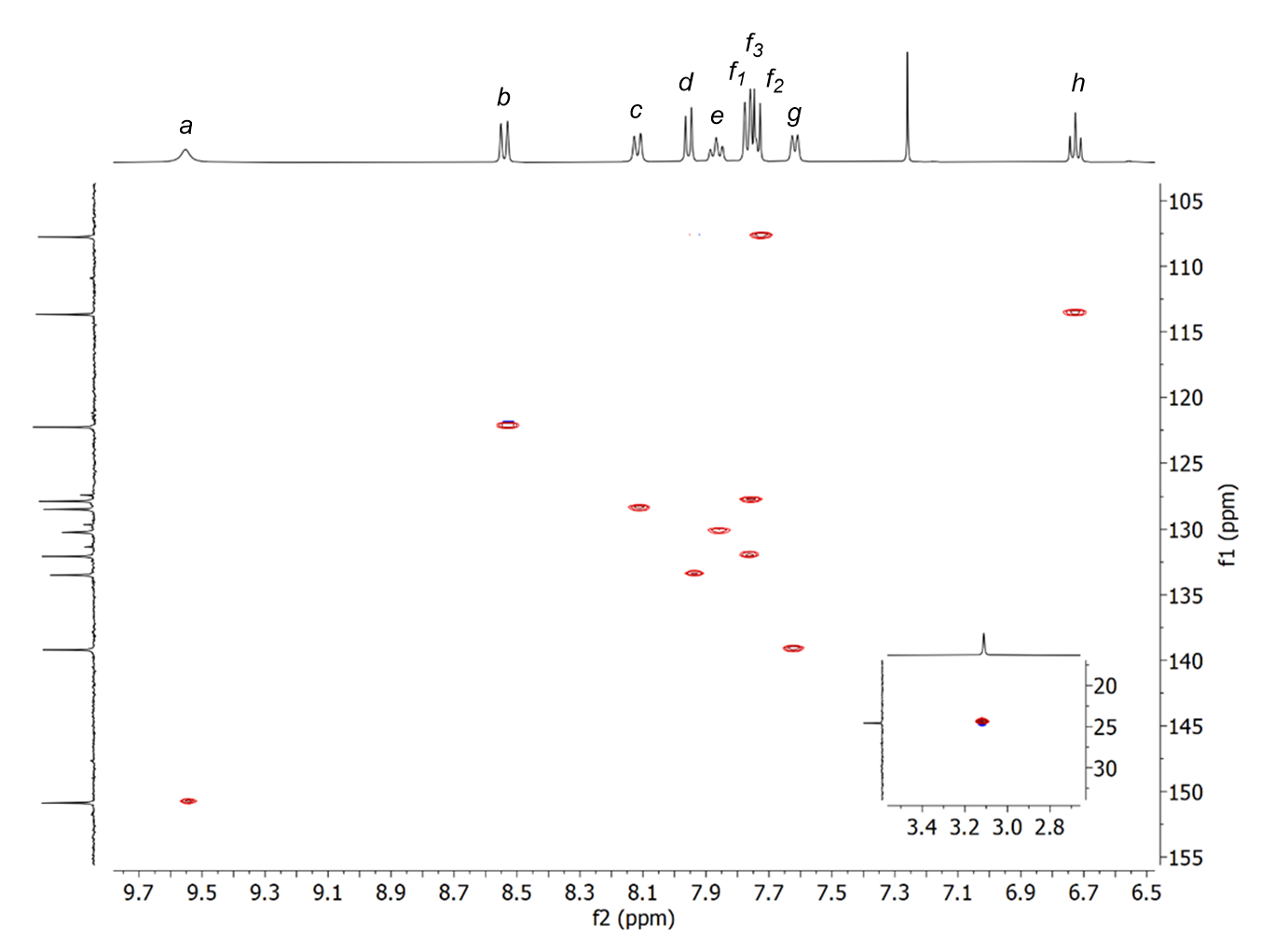


HMBC (400, 100.5 MHz, CDCl_3_)


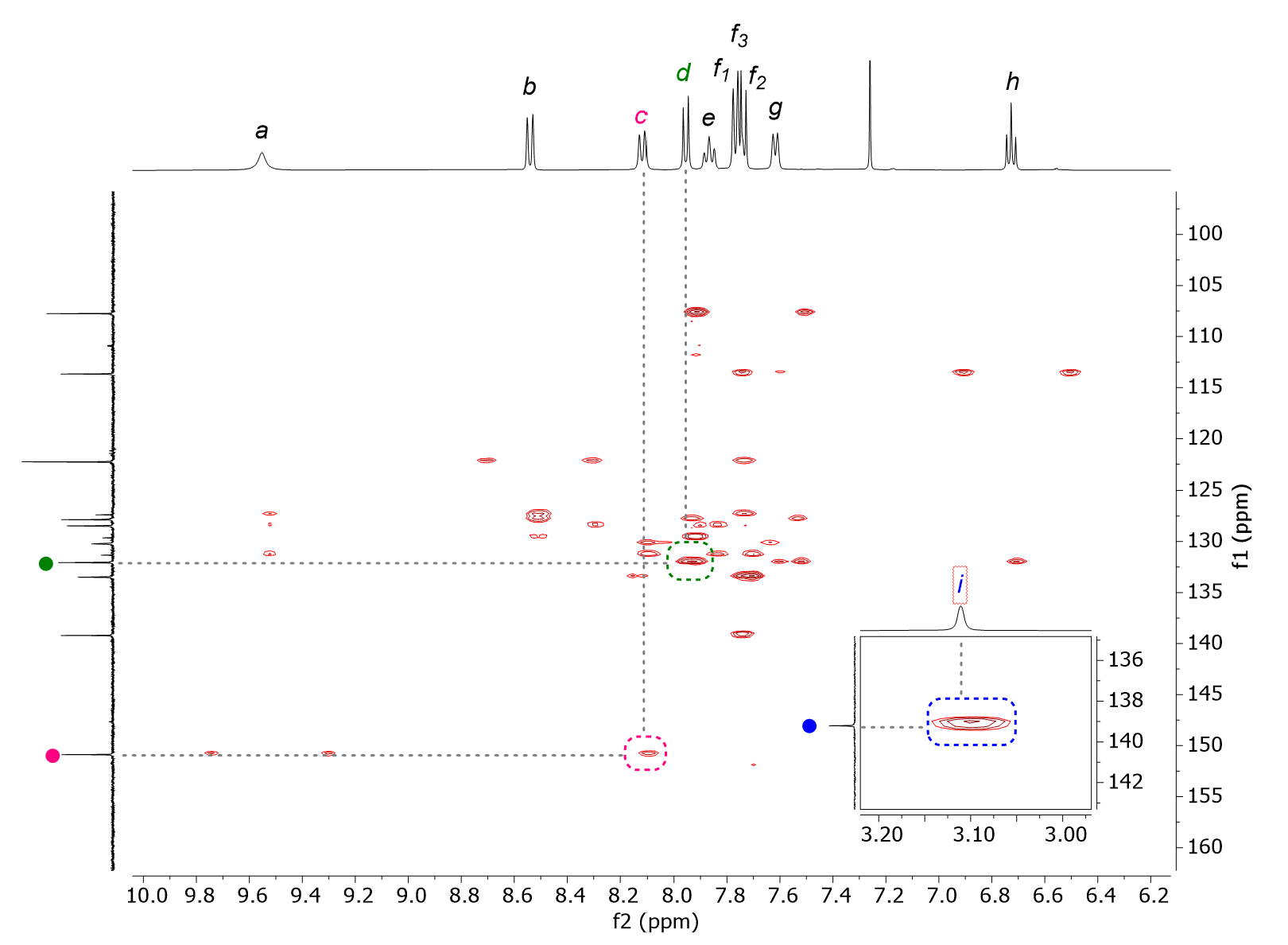


^1^H NMR (400 MHz, CDCl_3_)


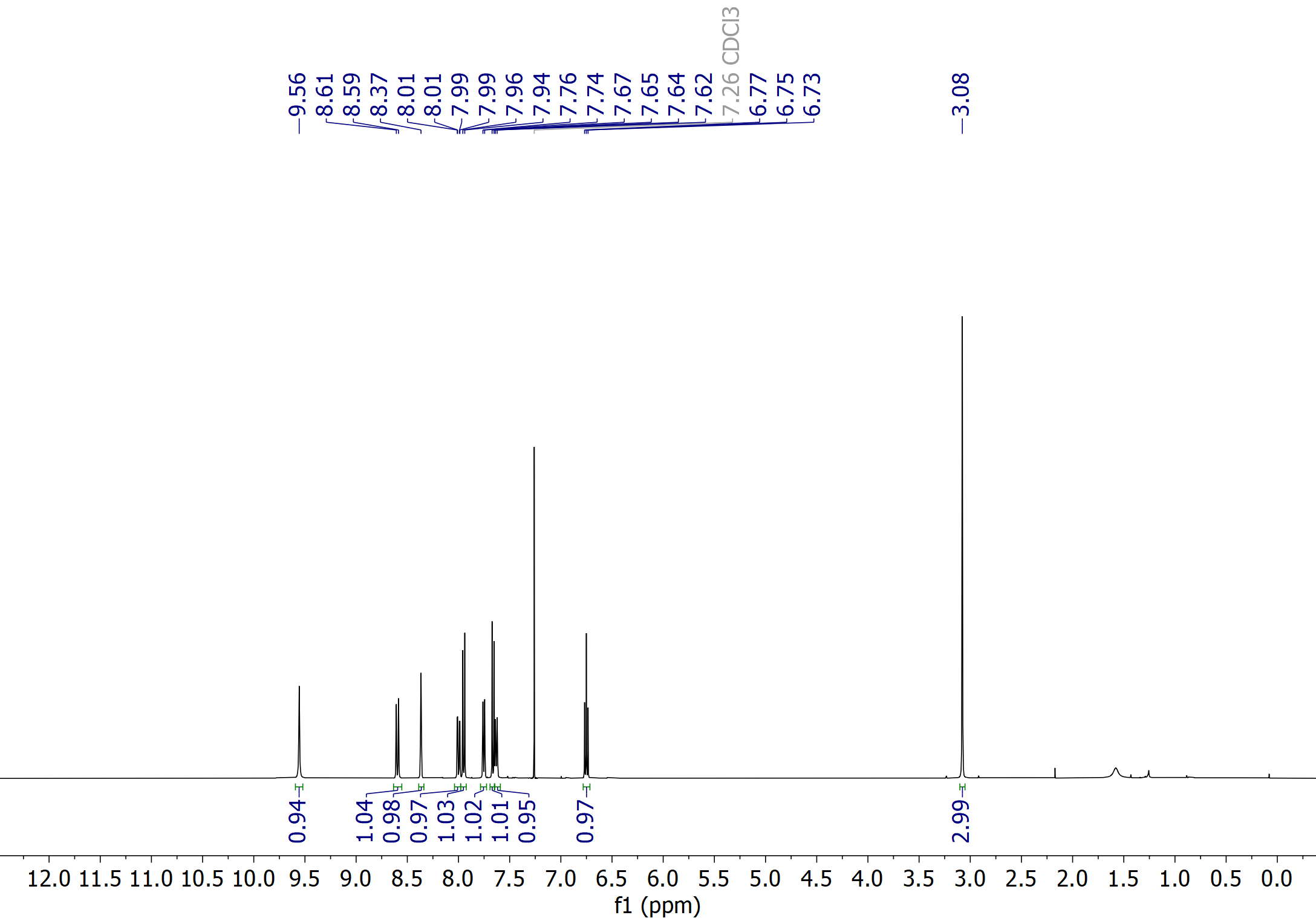


^13^C NMR (100.5 MHz, CDCl_3_)


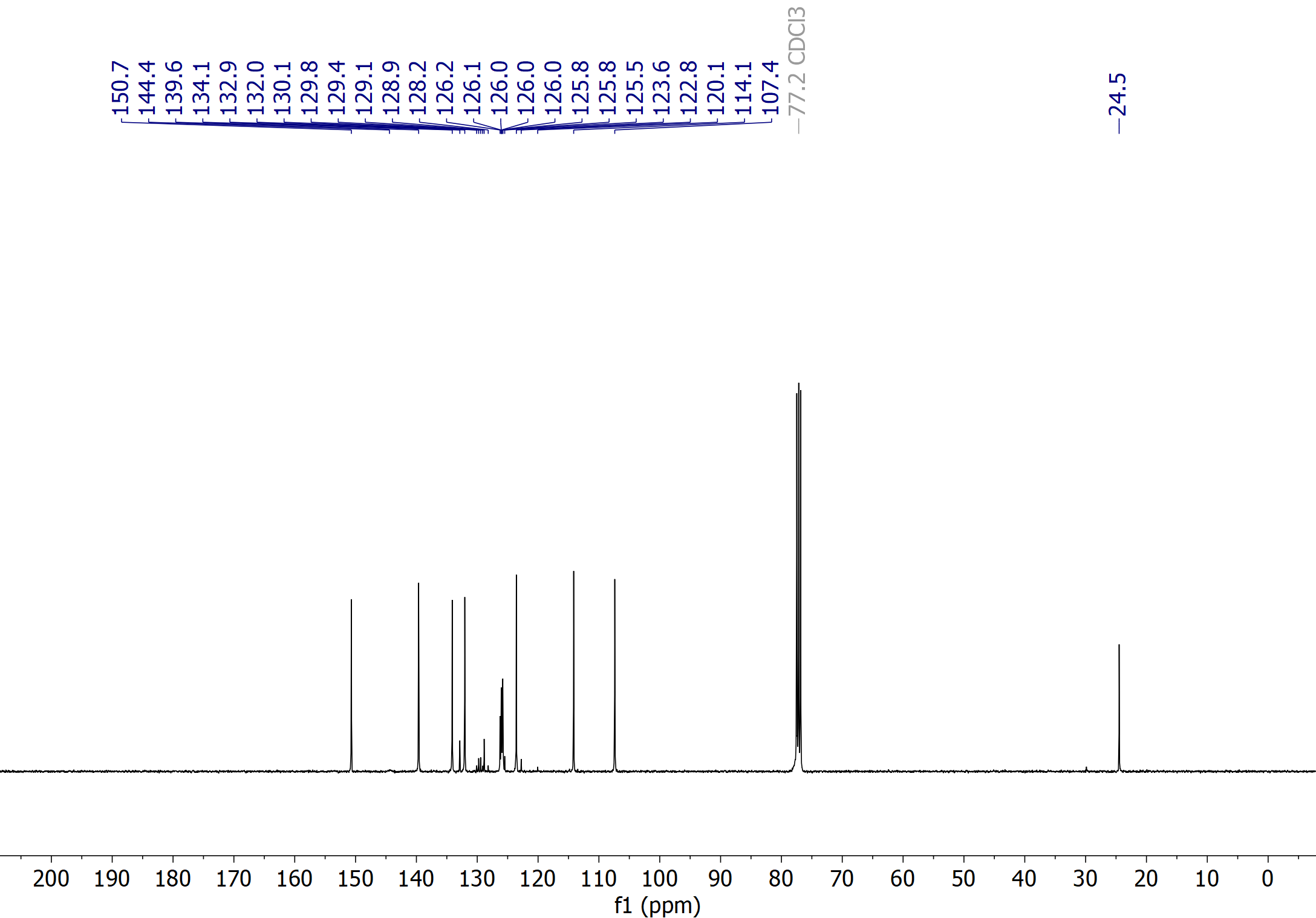


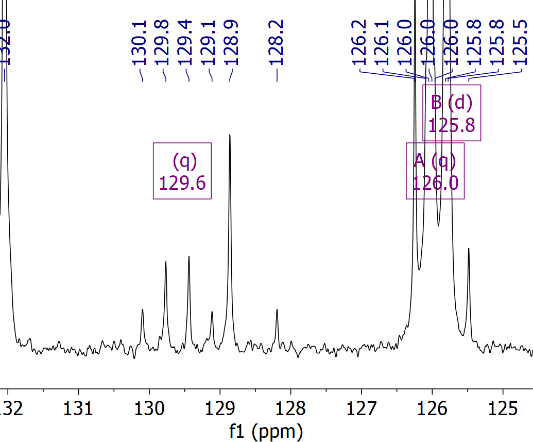

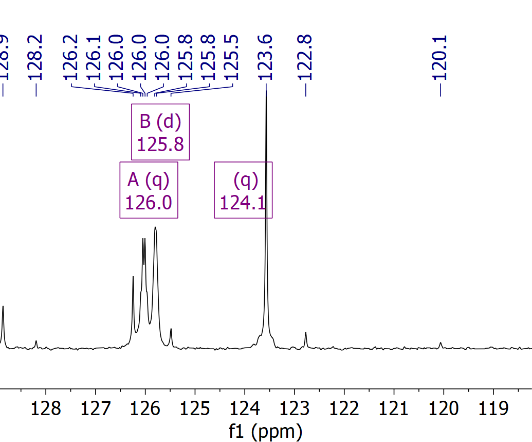


^19^F NMR (376 MHz, CDCl_3_)


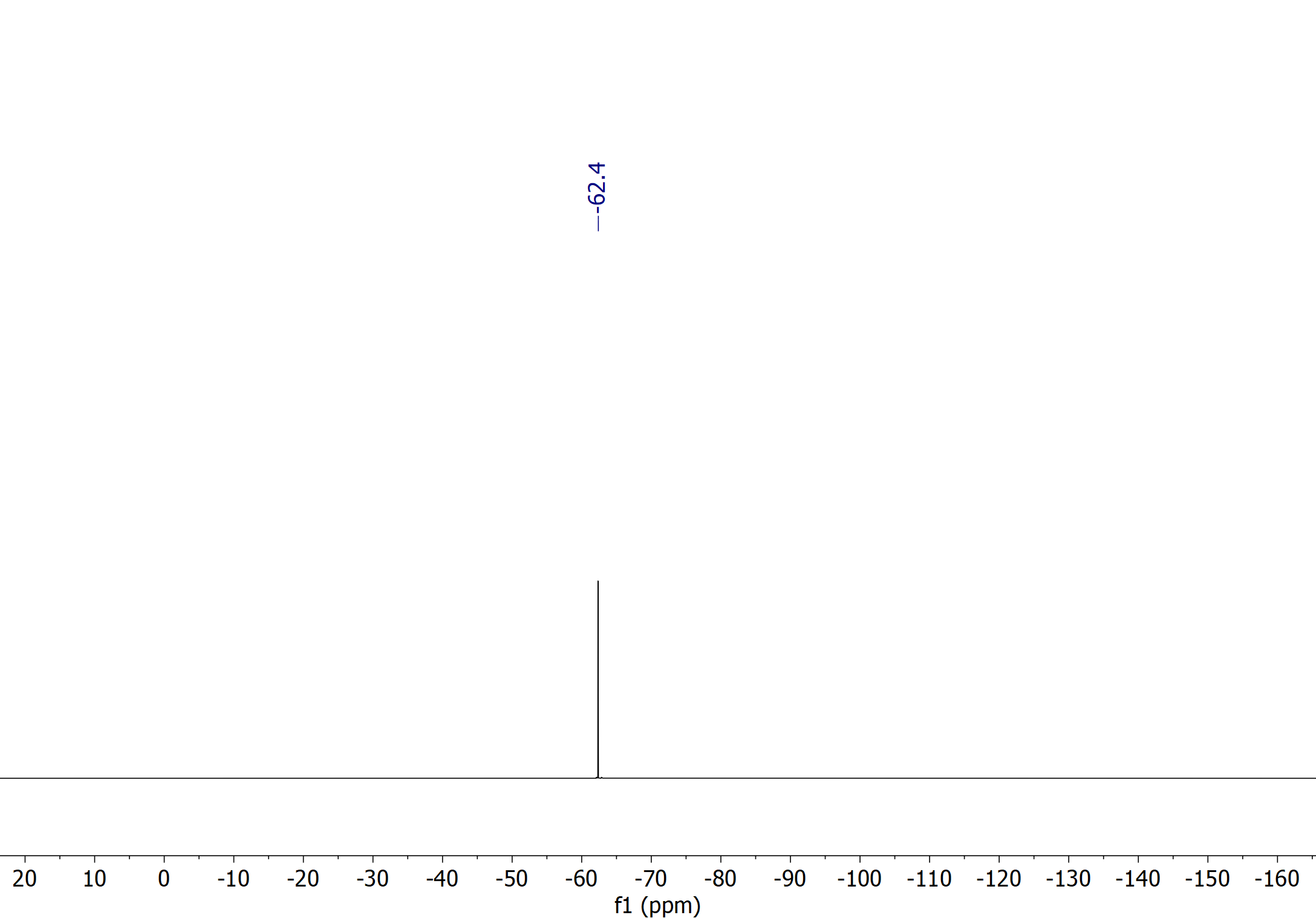


^11^B NMR (128 MHz, CDCl_3_)


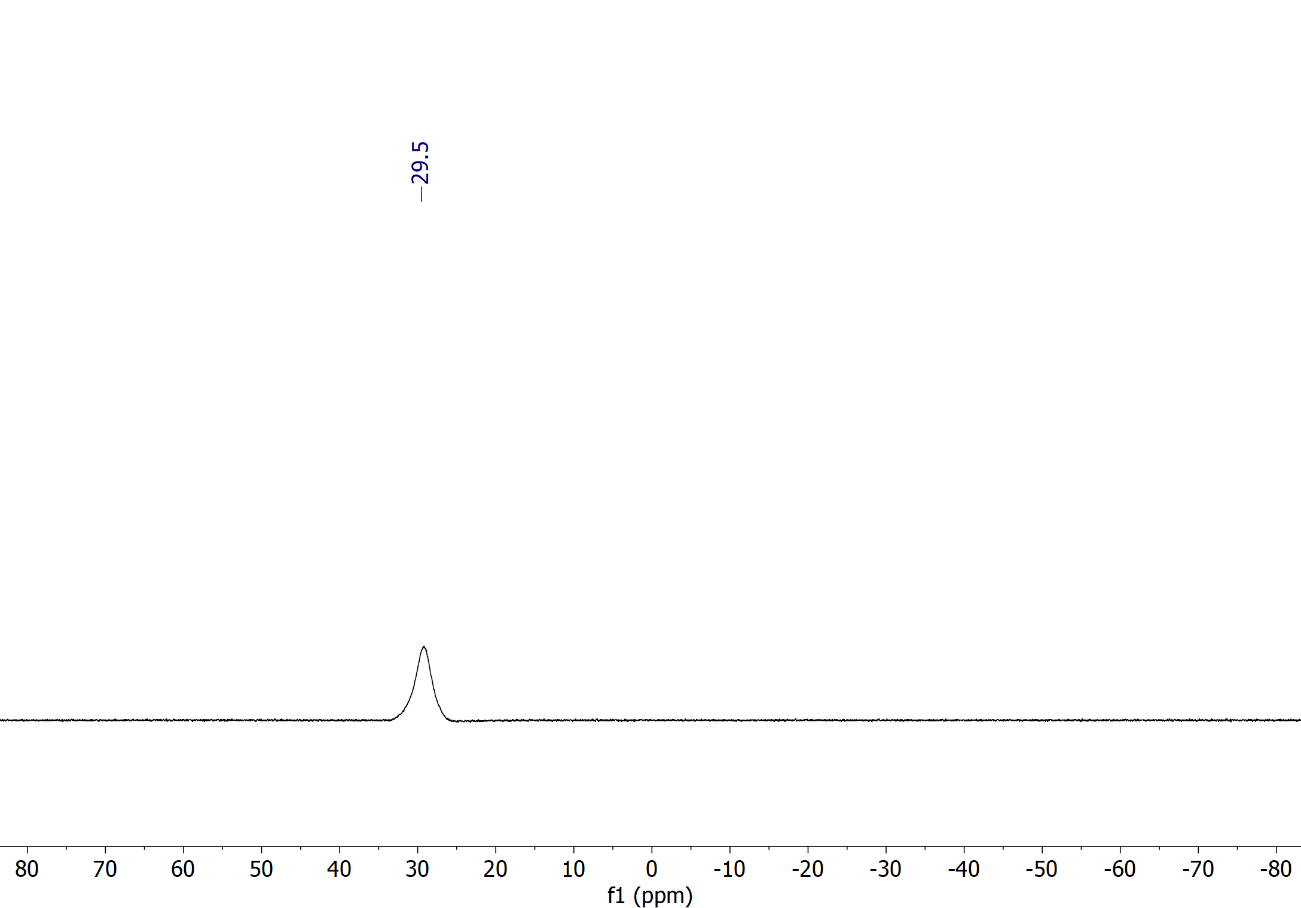


^1^H NMR (400 MHz, CD_2_Cl_2_)


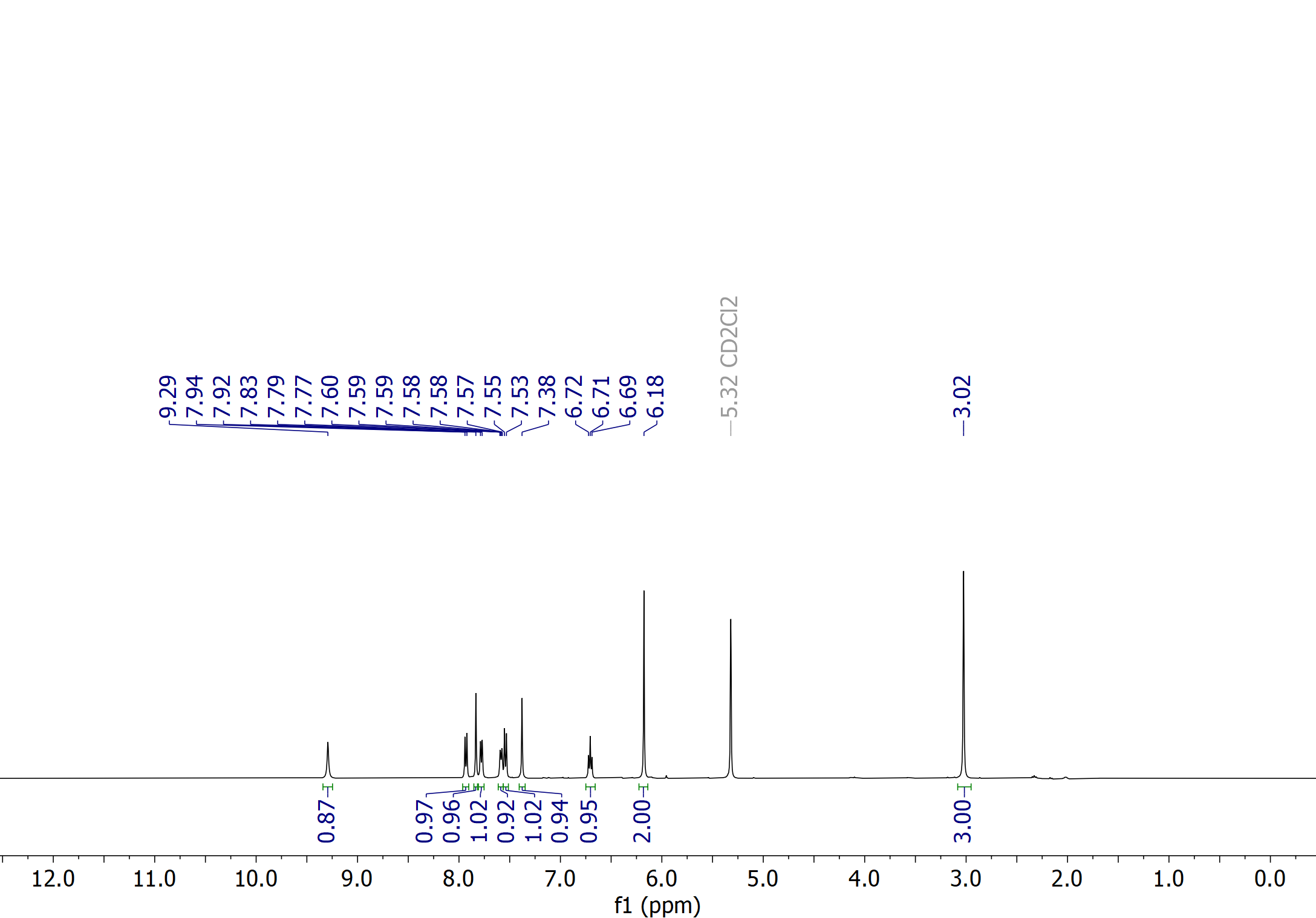


^13^C NMR (100.5 MHz, CD_2_Cl_2_)


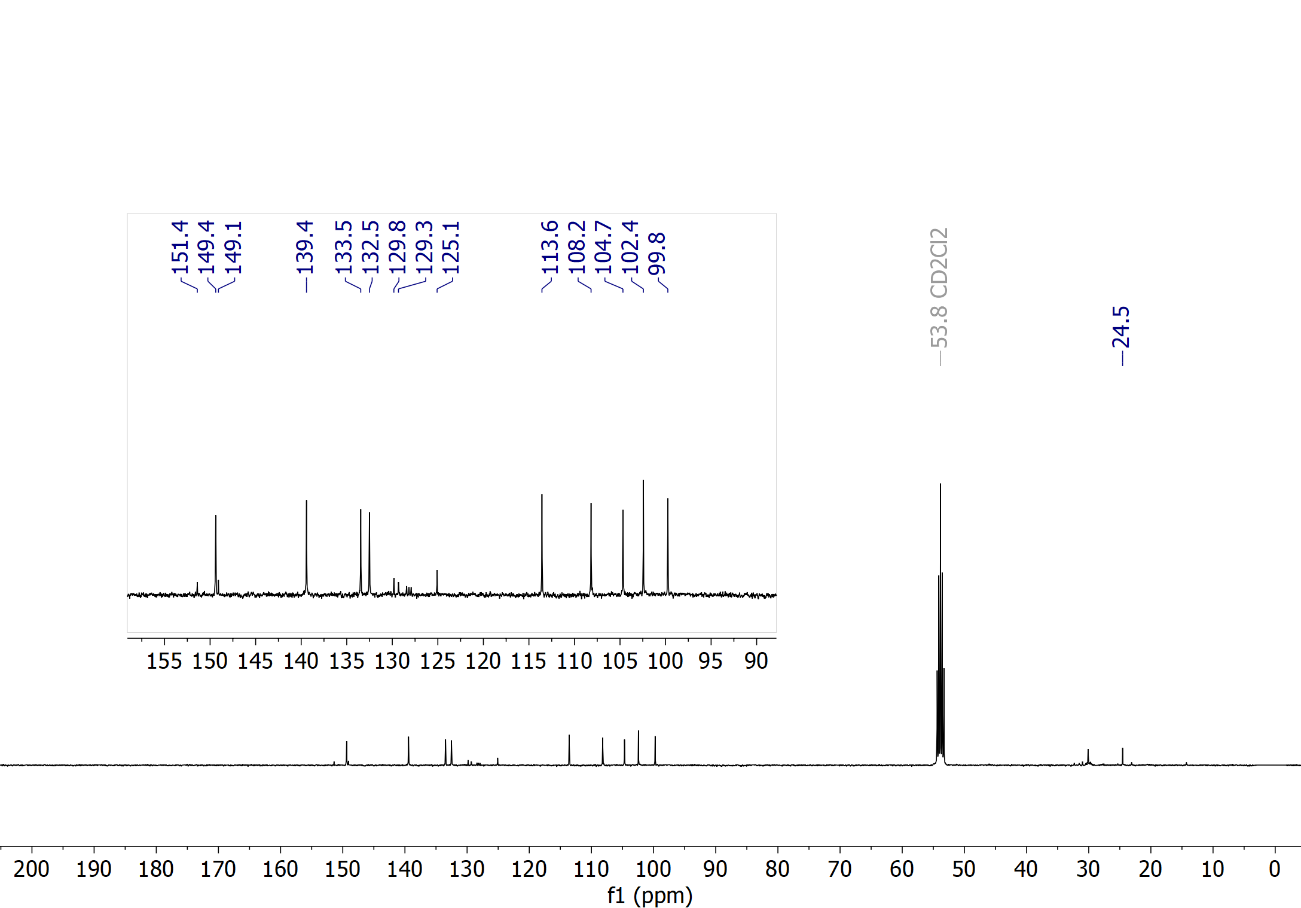


^11^B NMR (128 MHz, CDCl_3_)


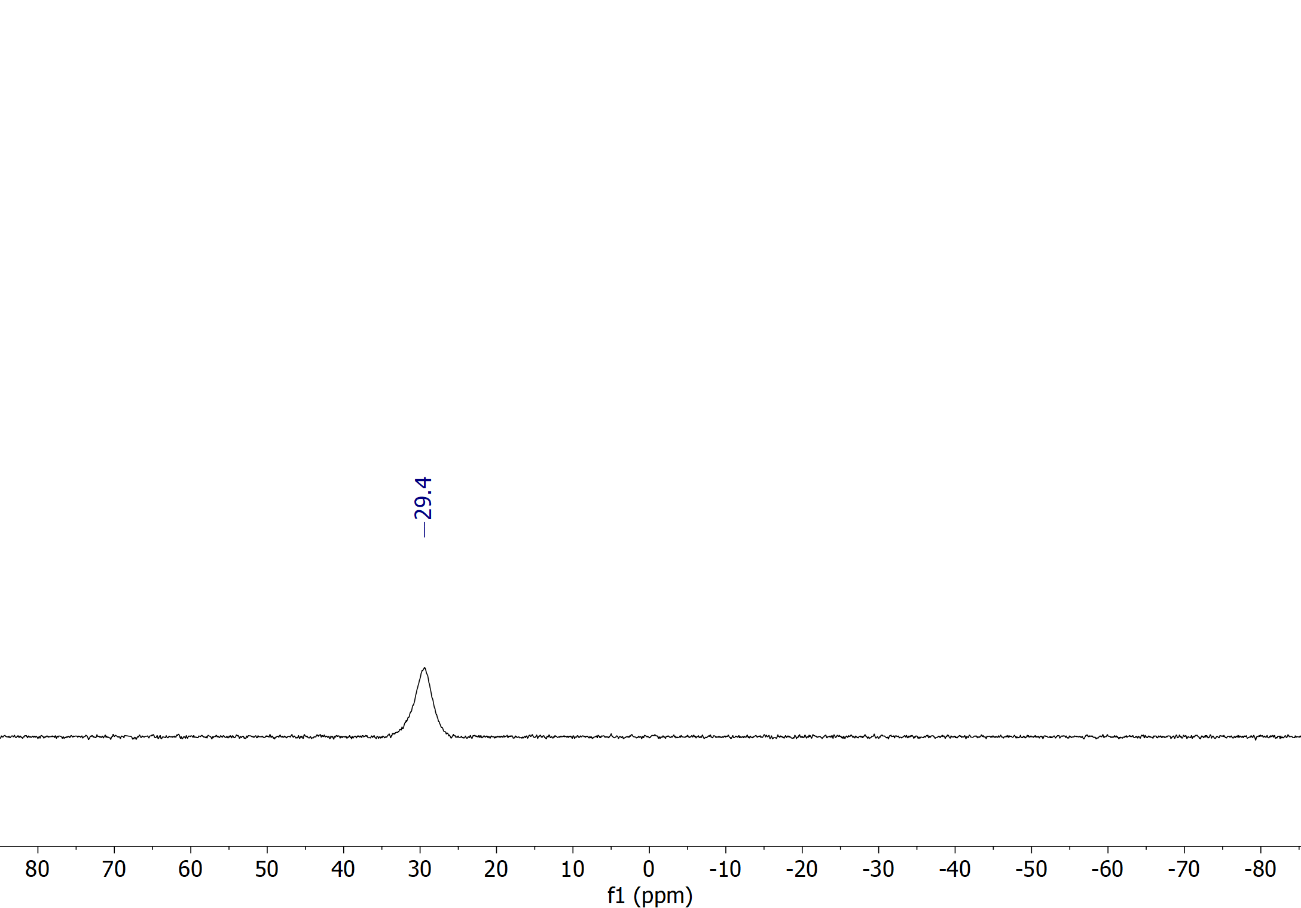


^1^H NMR (400 MHz, CDCl_3_)


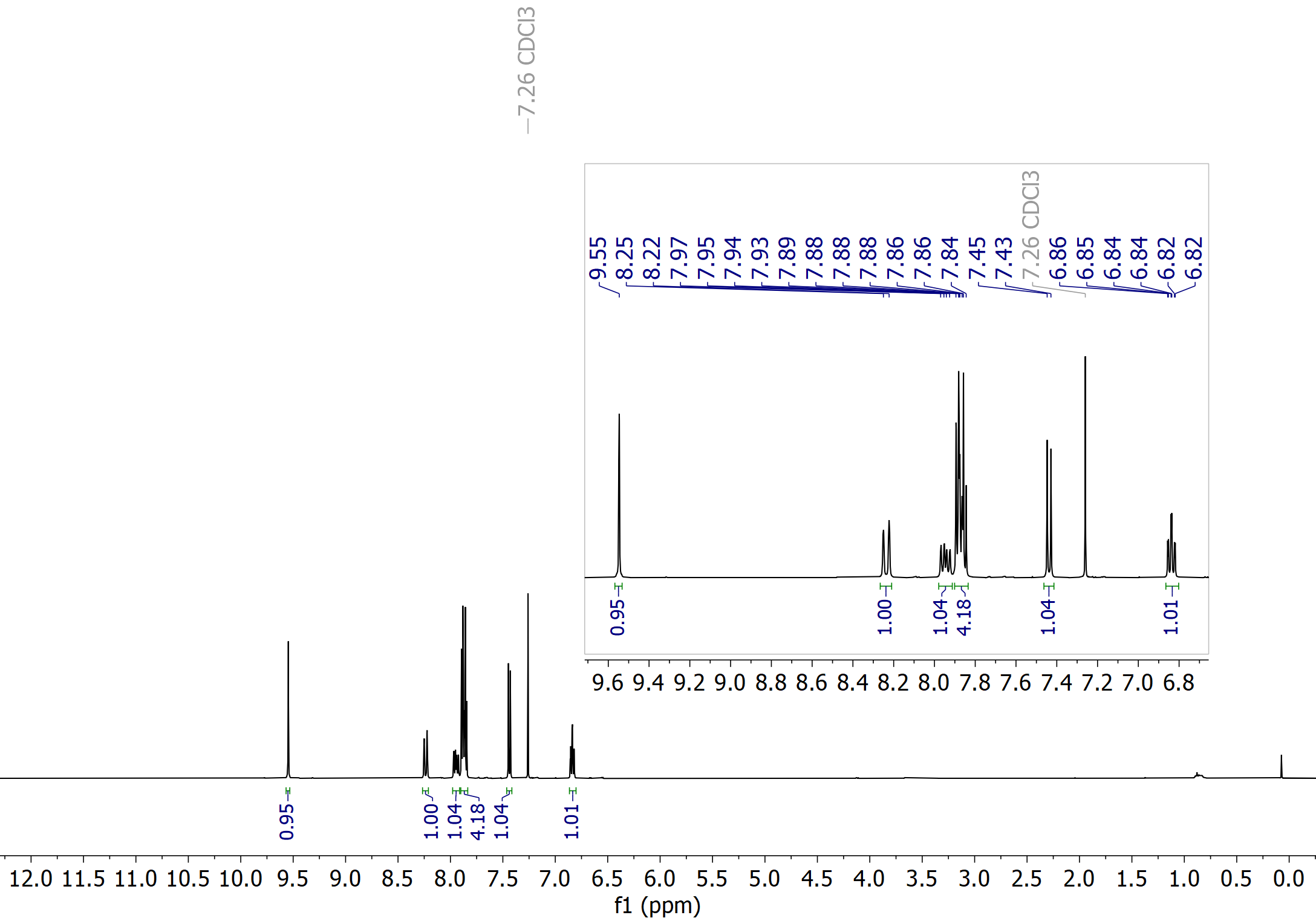


^13^C NMR (100.5 MHz, CDCl_3_)


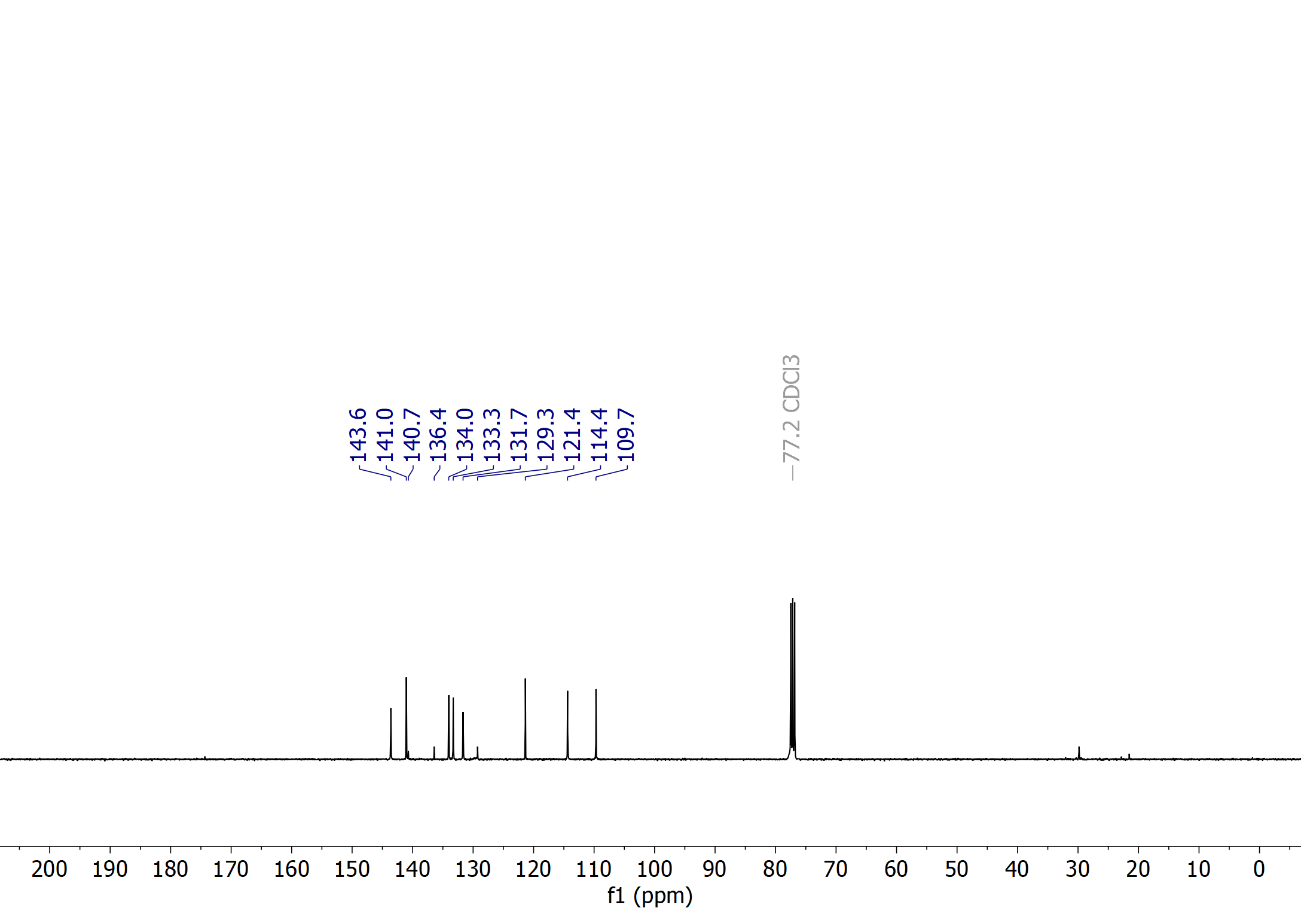


^11^B NMR (128 MHz, CDCl_3_)


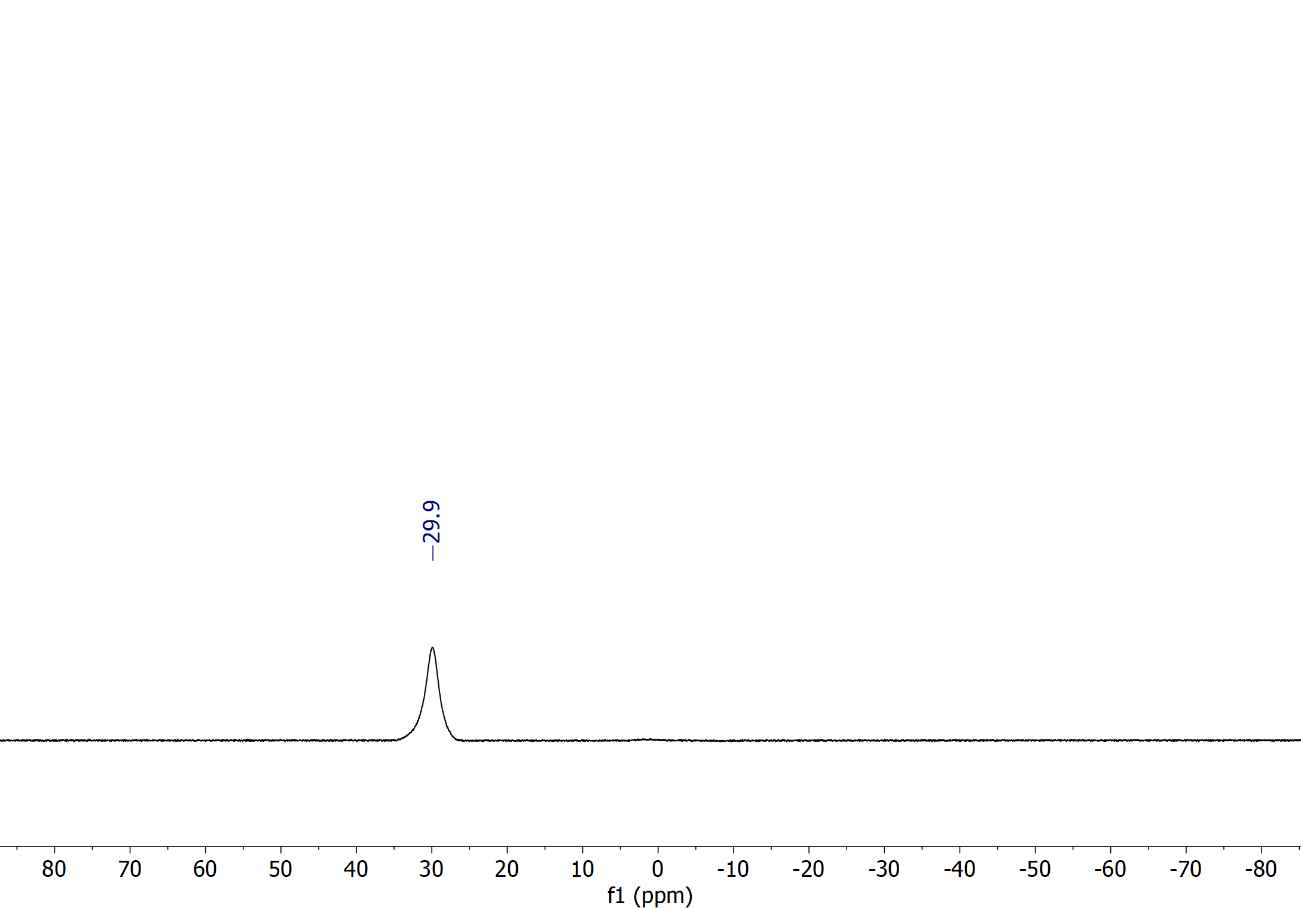


^1^H NMR (400 MHz, CDCl_3_)


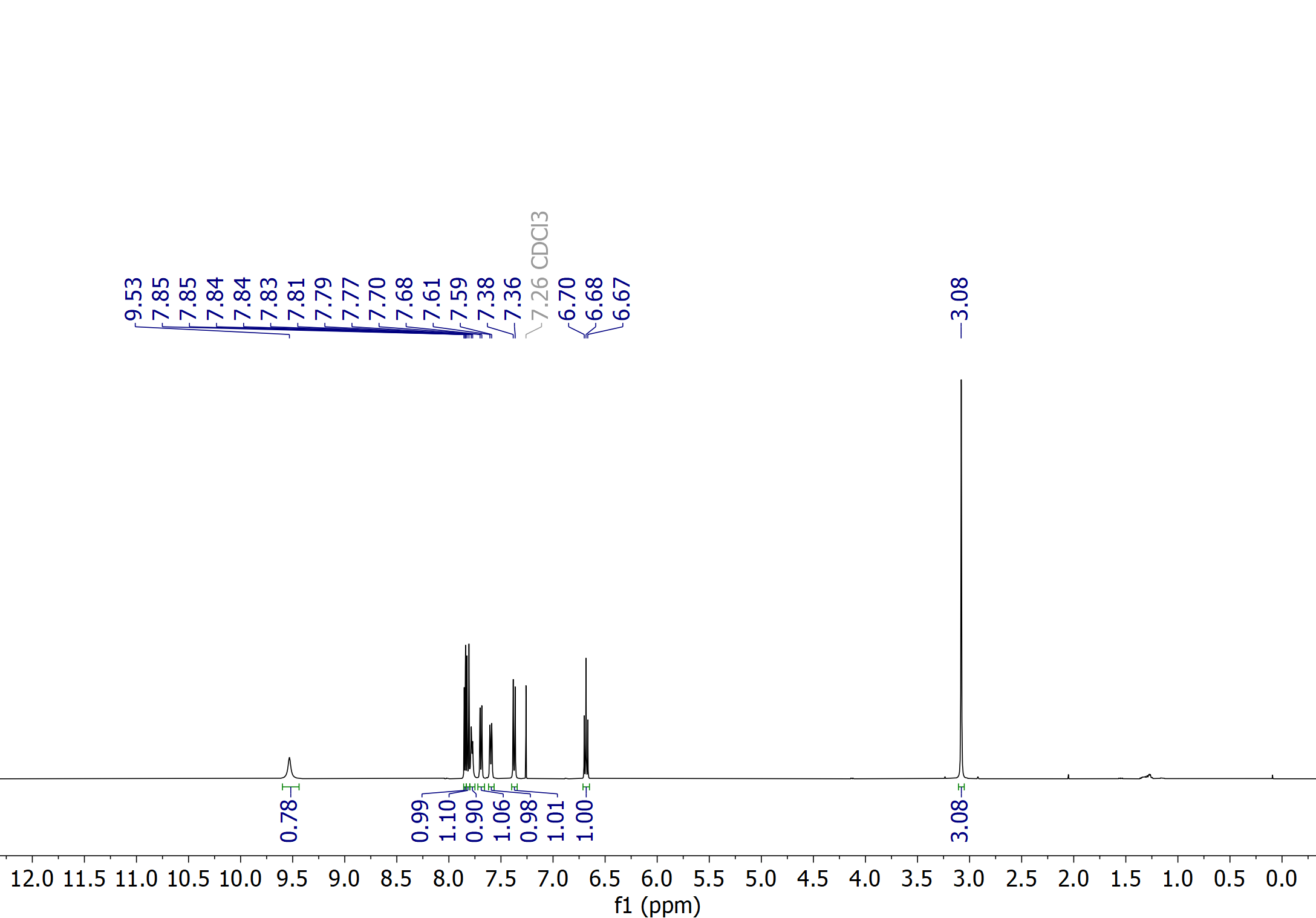


^13^C NMR (100.5 MHz, CDCl_3_)


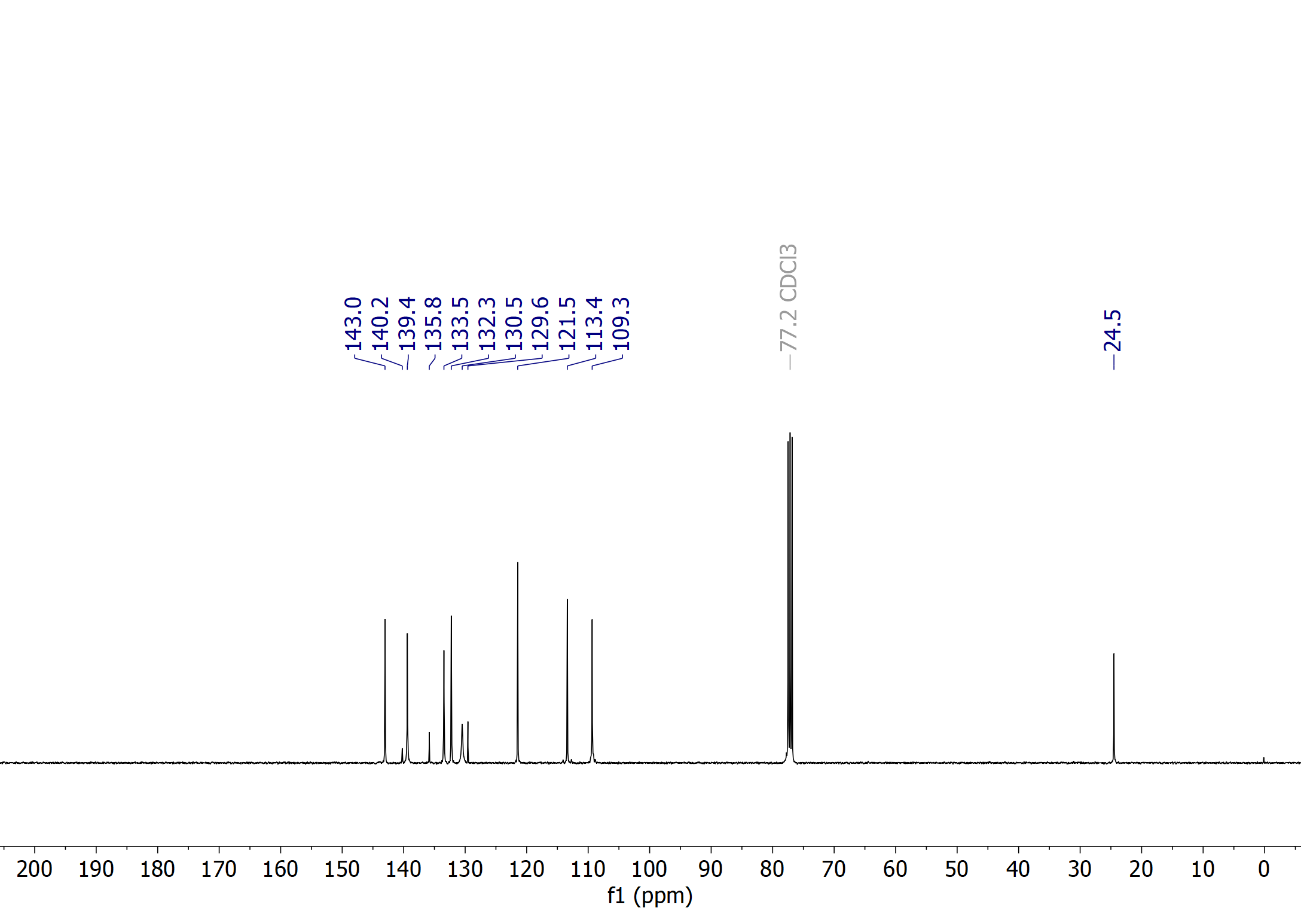


^11^B NMR (128 MHz, CDCl_3_)


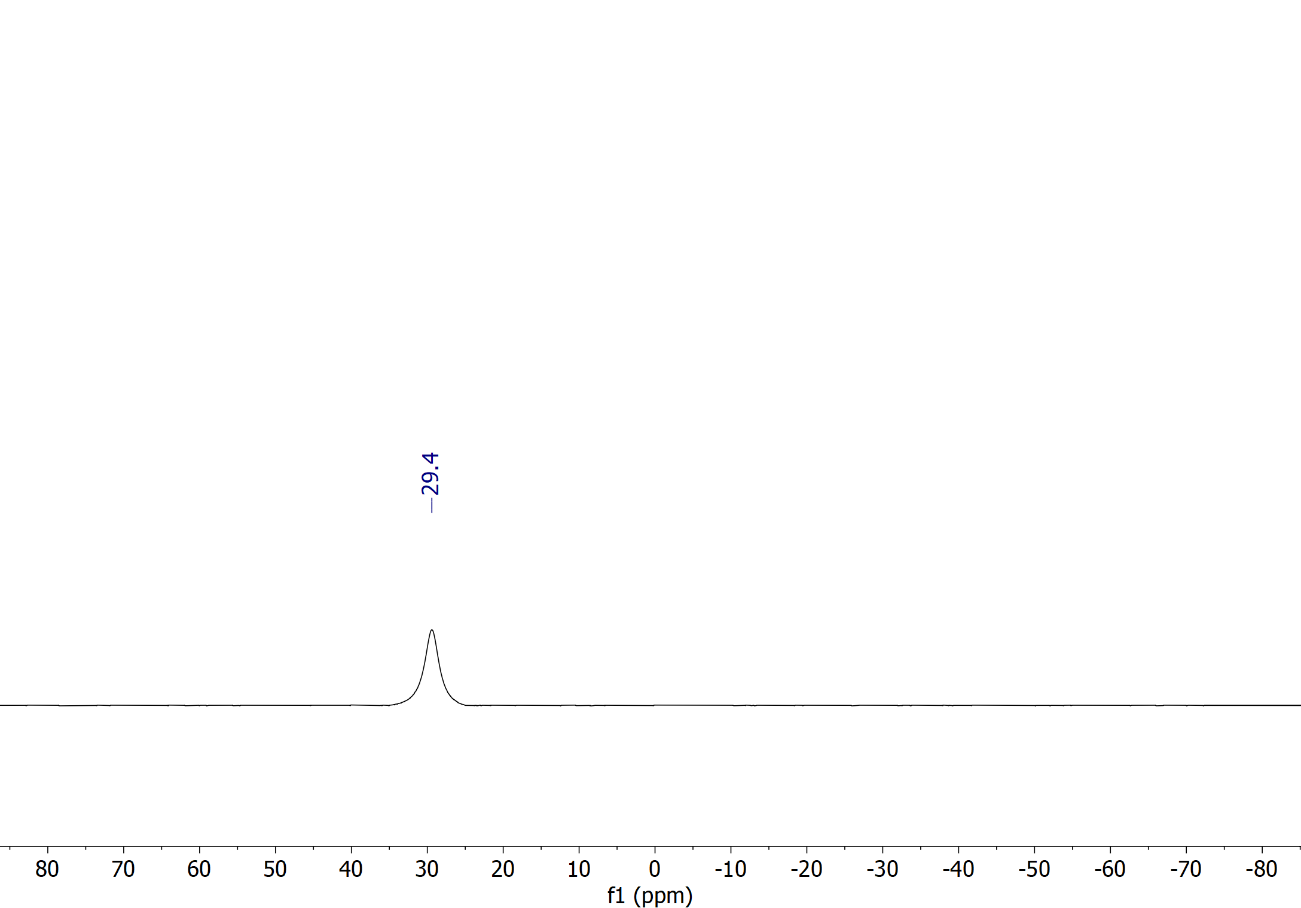


^1^H NMR (400 MHz, CDCl_3_)


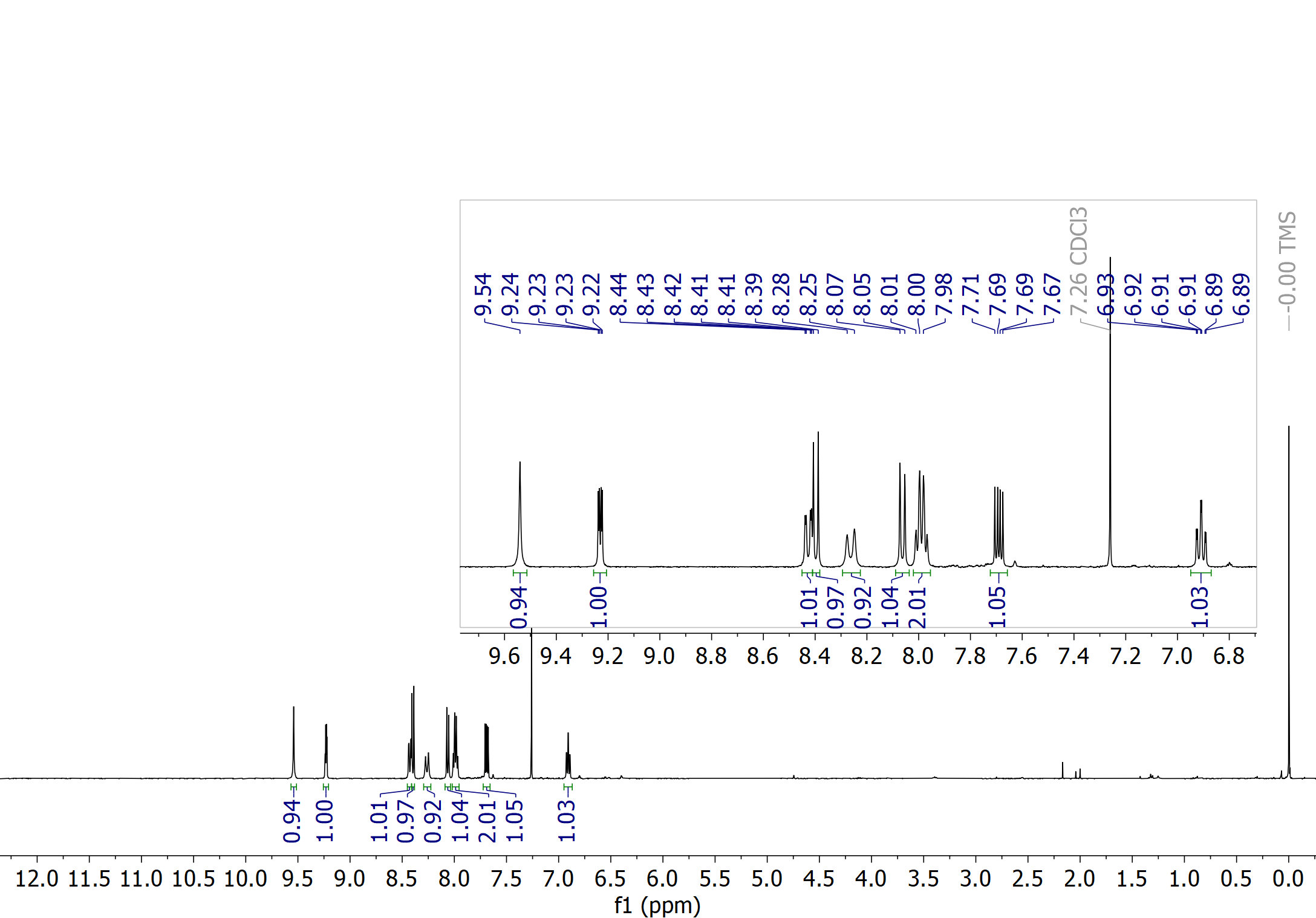


^13^C NMR (100.5 MHz, CDCl_3_)


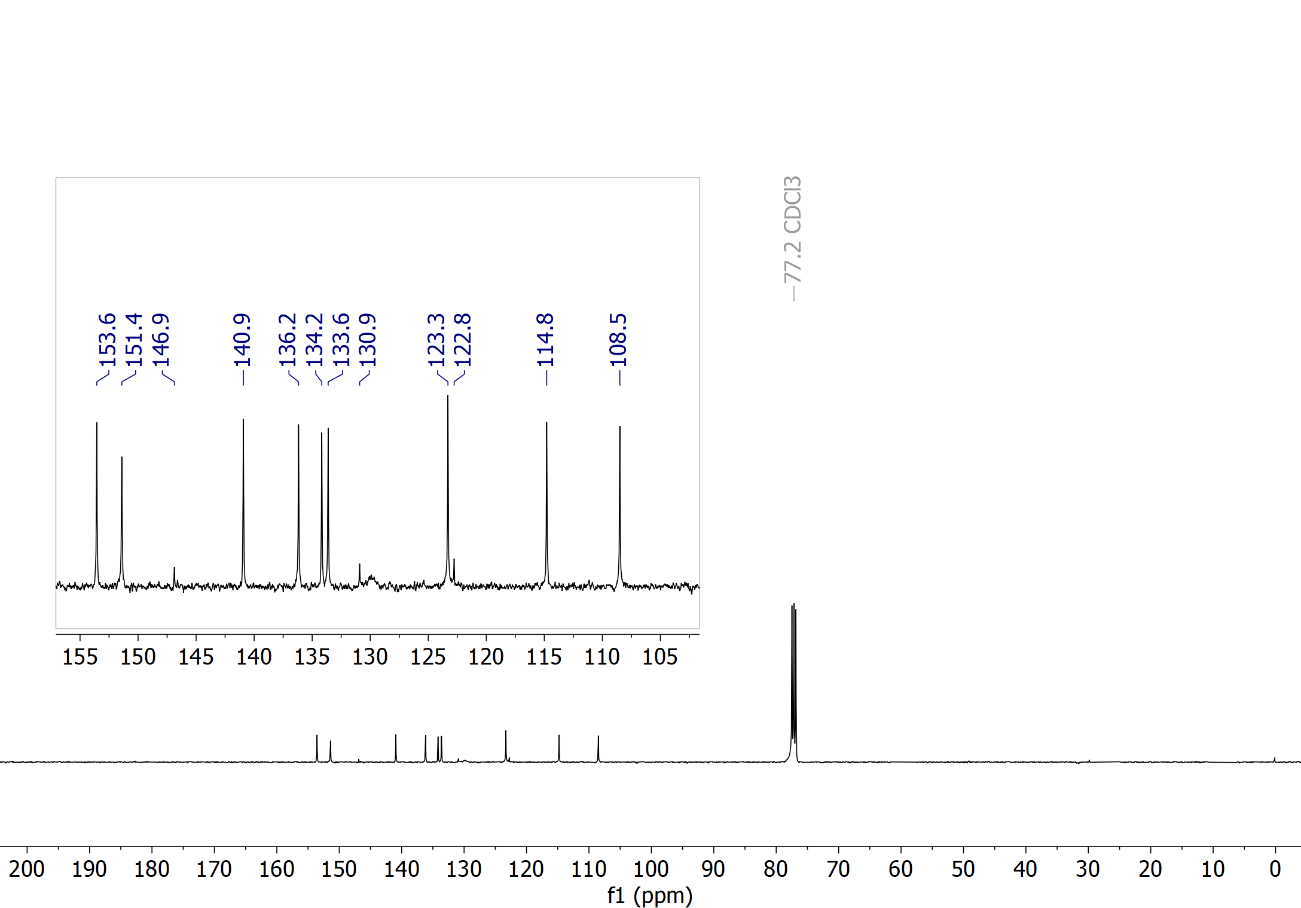


^11^B NMR (128 MHz, CDCl_3_)


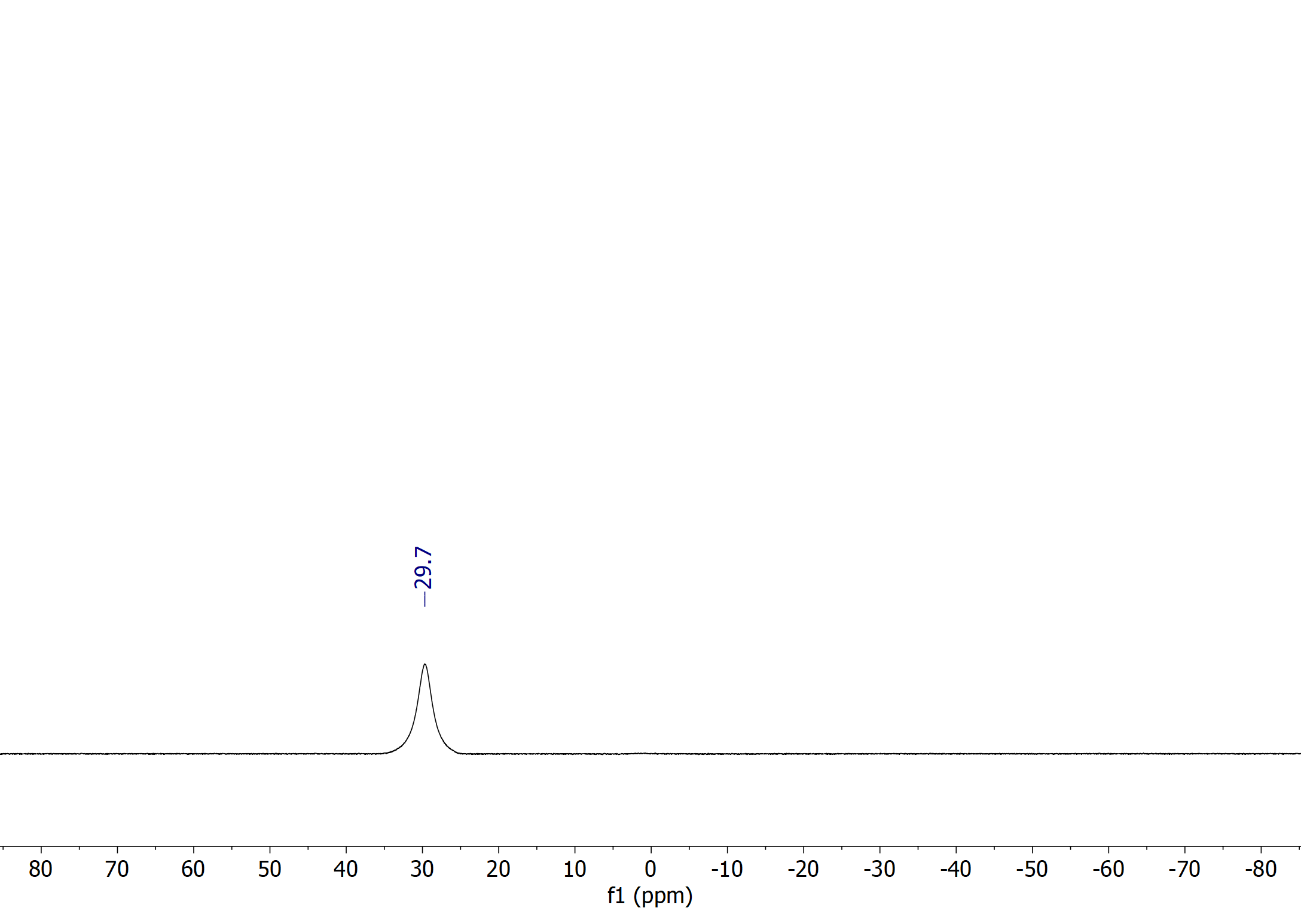


^1^H NMR (400 MHz, DMSO-*d*_6_)


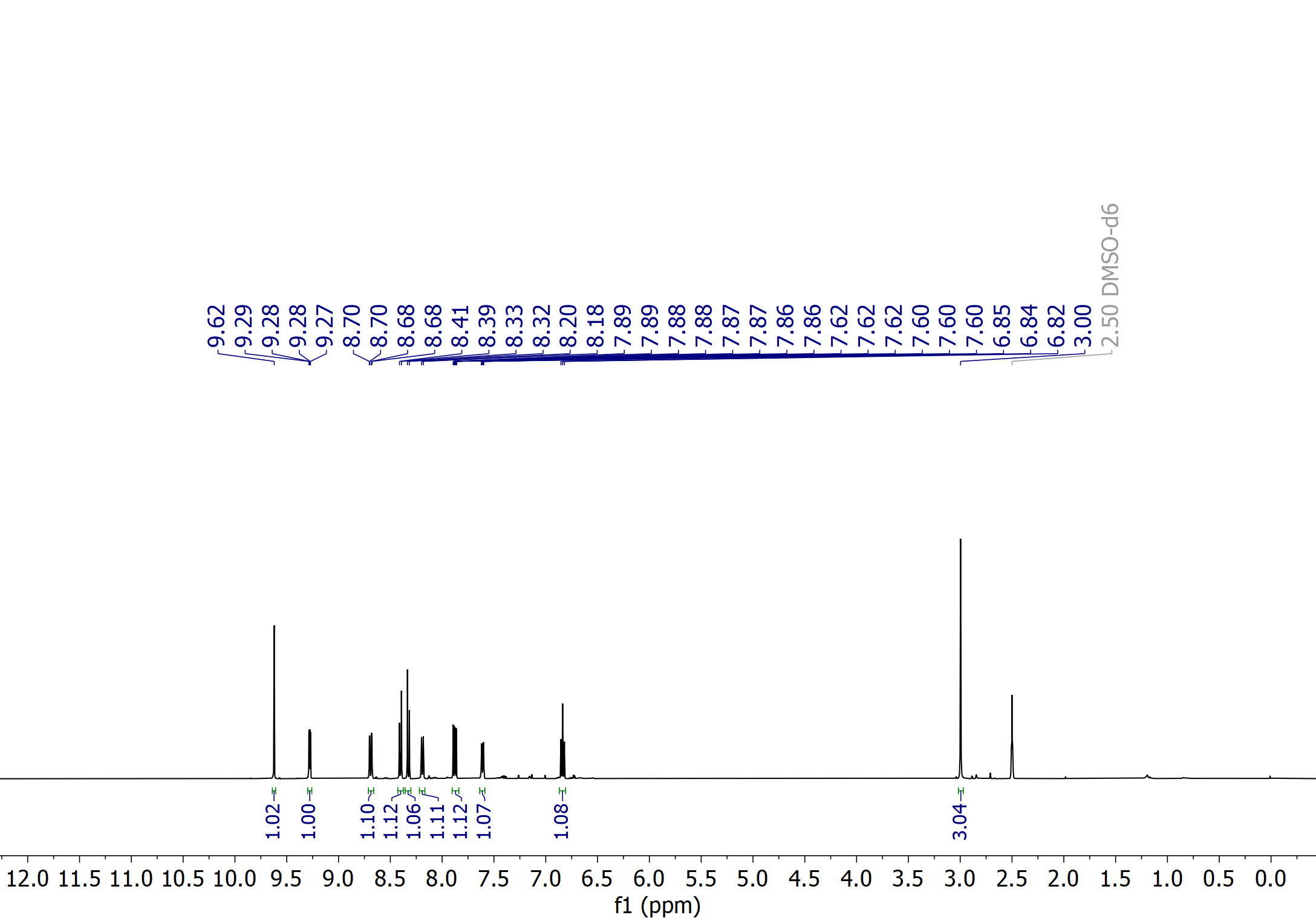


^13^C NMR (100.5 MHz, DMSO-*d*_6_)


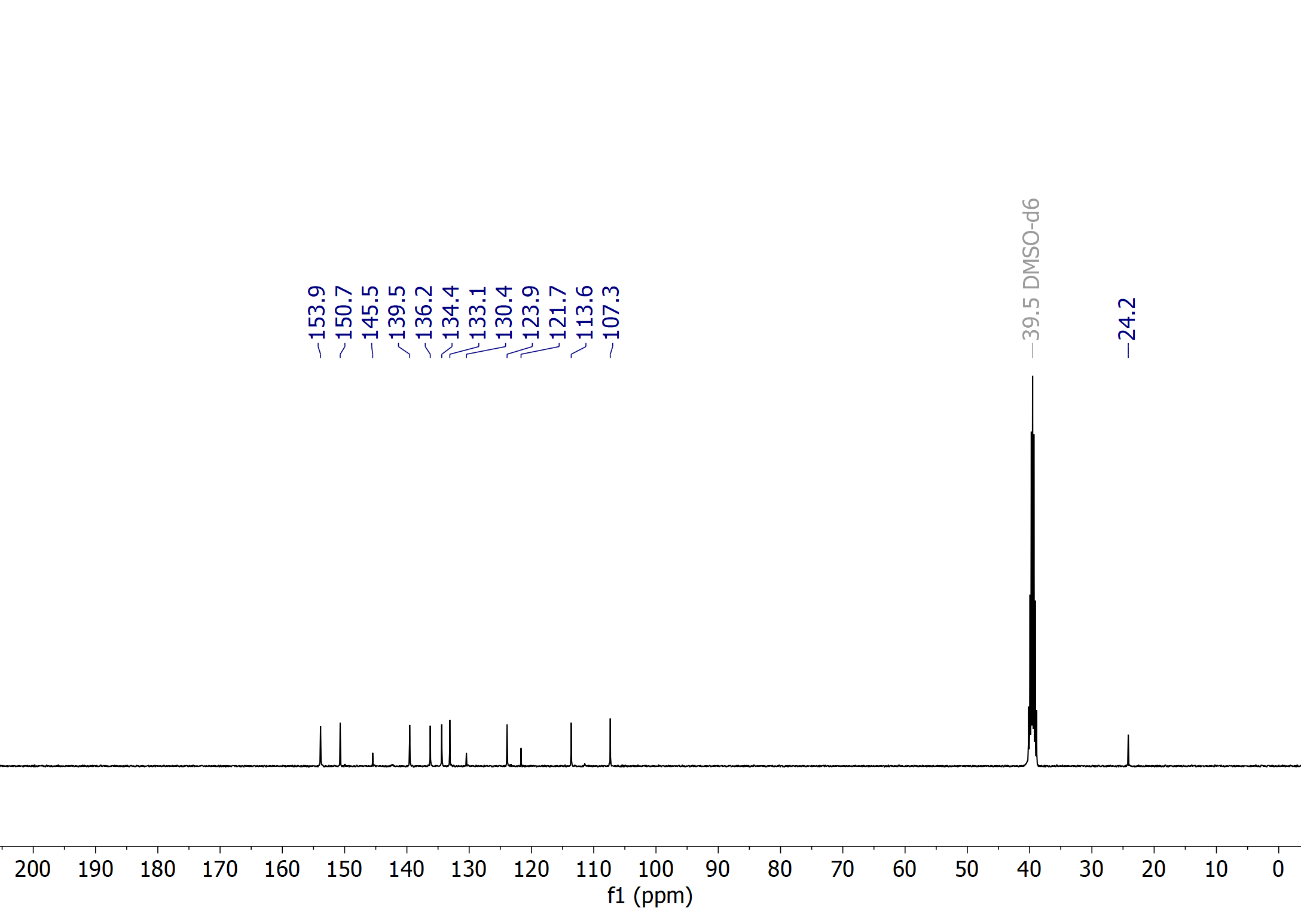


^11^B NMR (128 MHz, DMSO-*d*_6_)


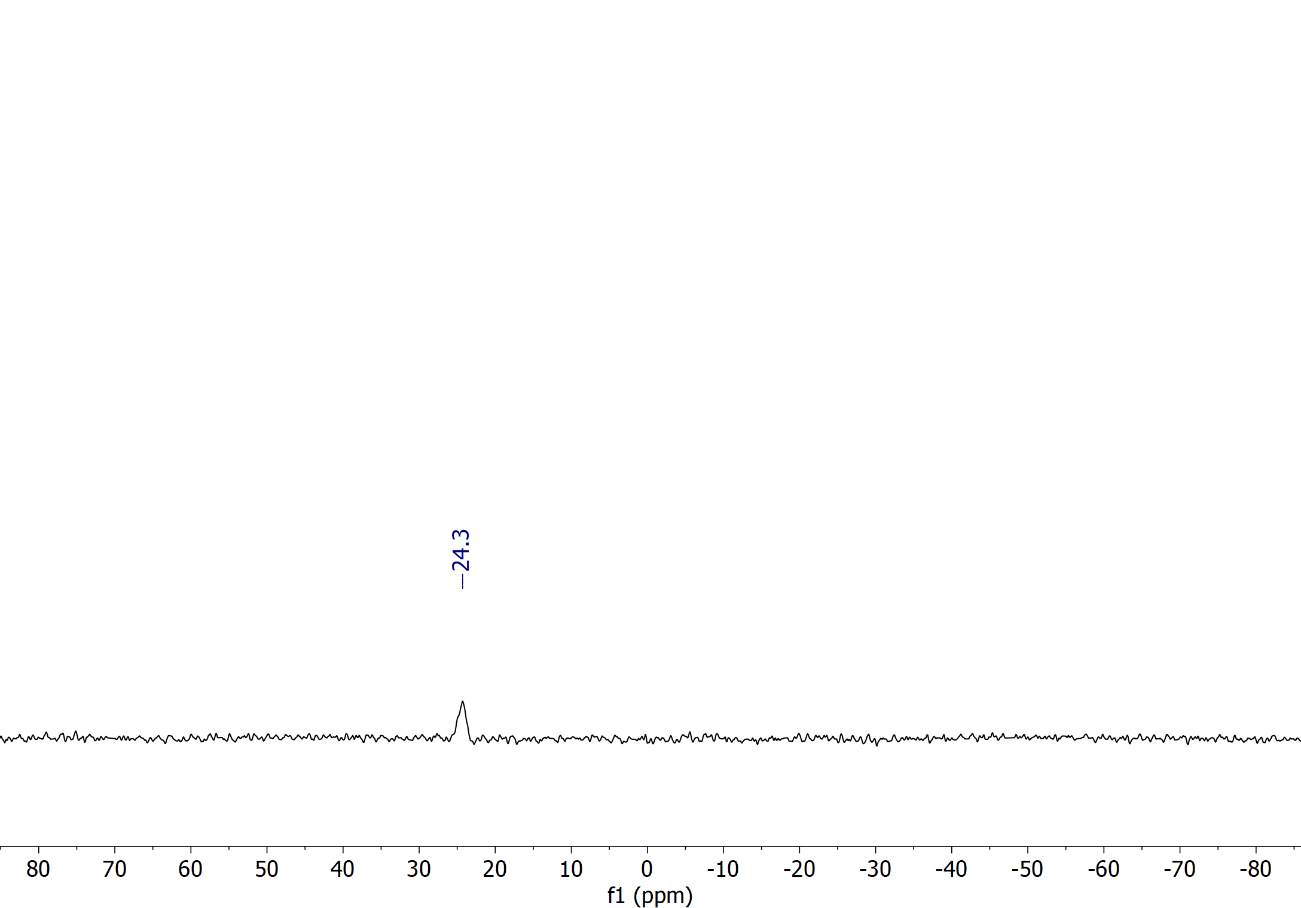


^1^H NMR (400 MHz, CDCl_3_)


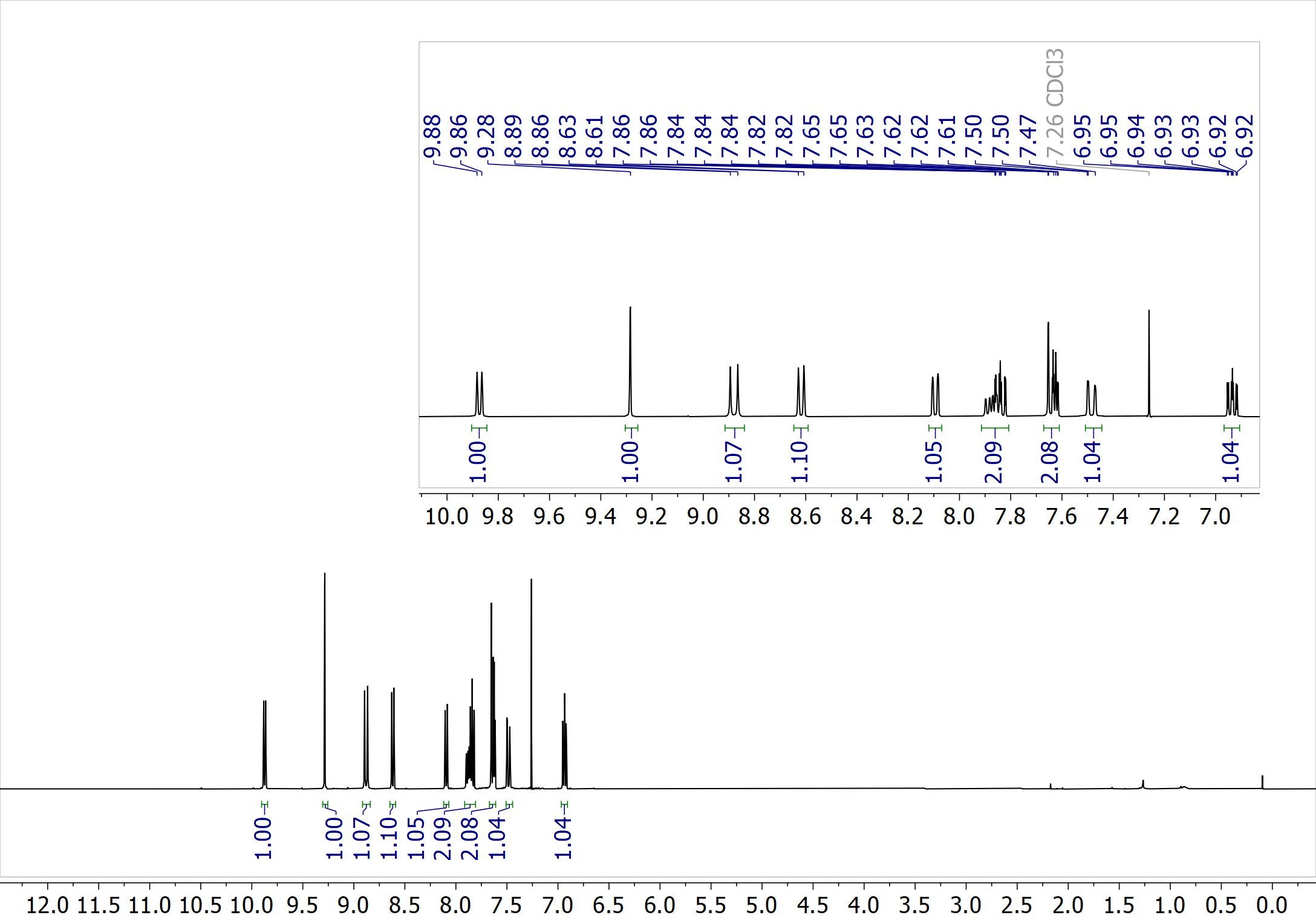


^13^C NMR (100.5 MHz, CDCl_3_)


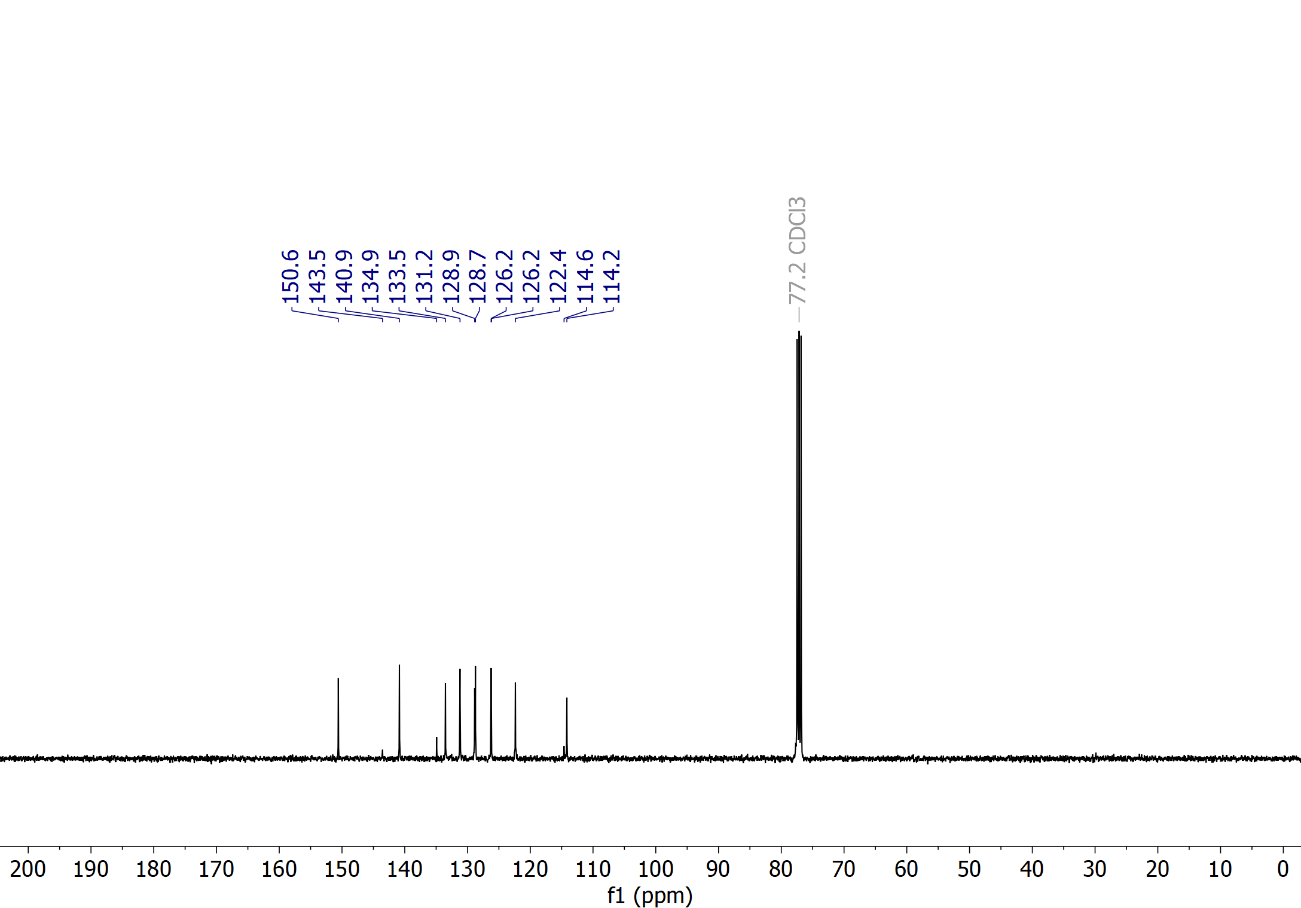


^11^B NMR (128 MHz, CDCl_3_)


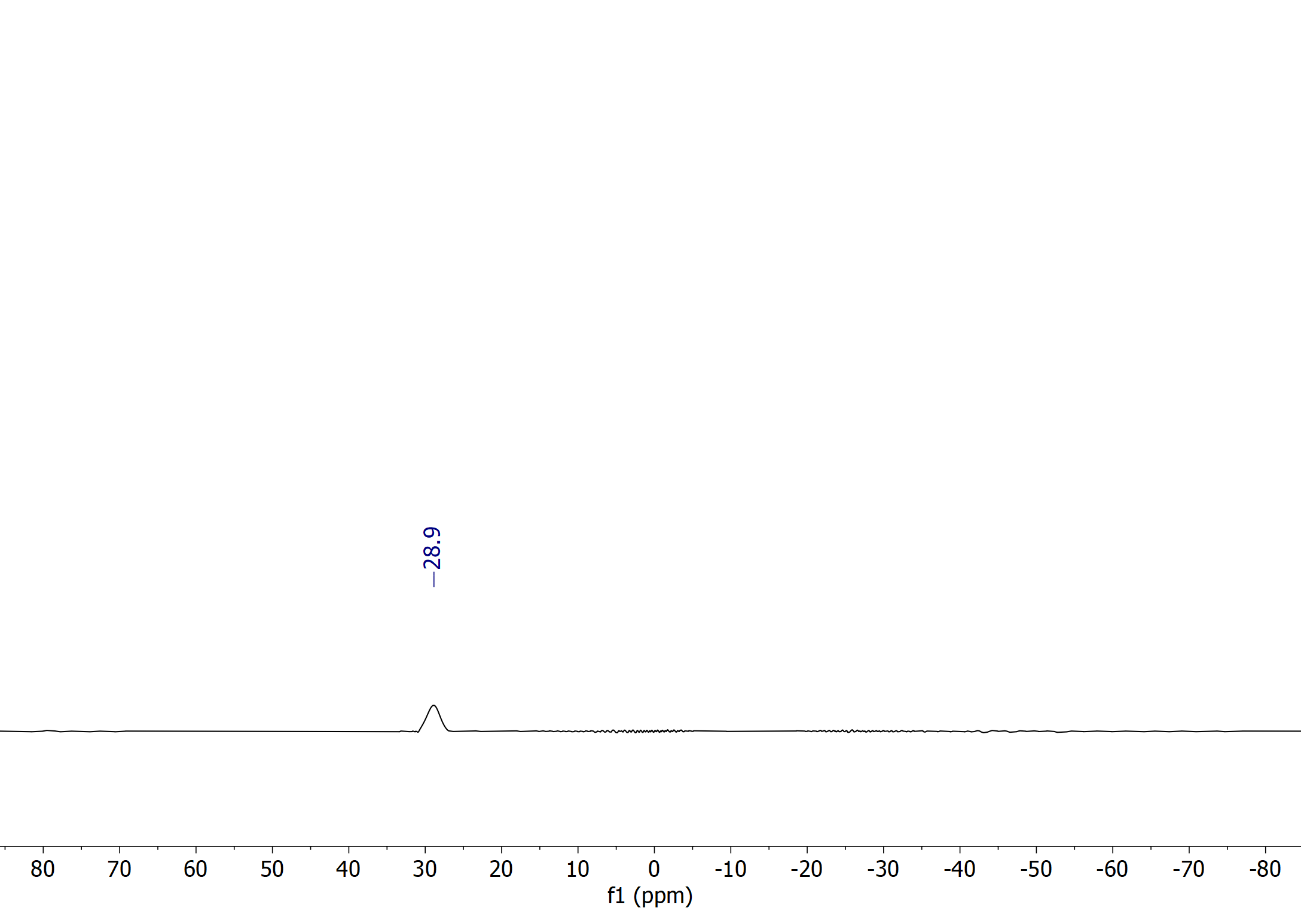


COSY (400, 400 MHz, CDCl_3_)


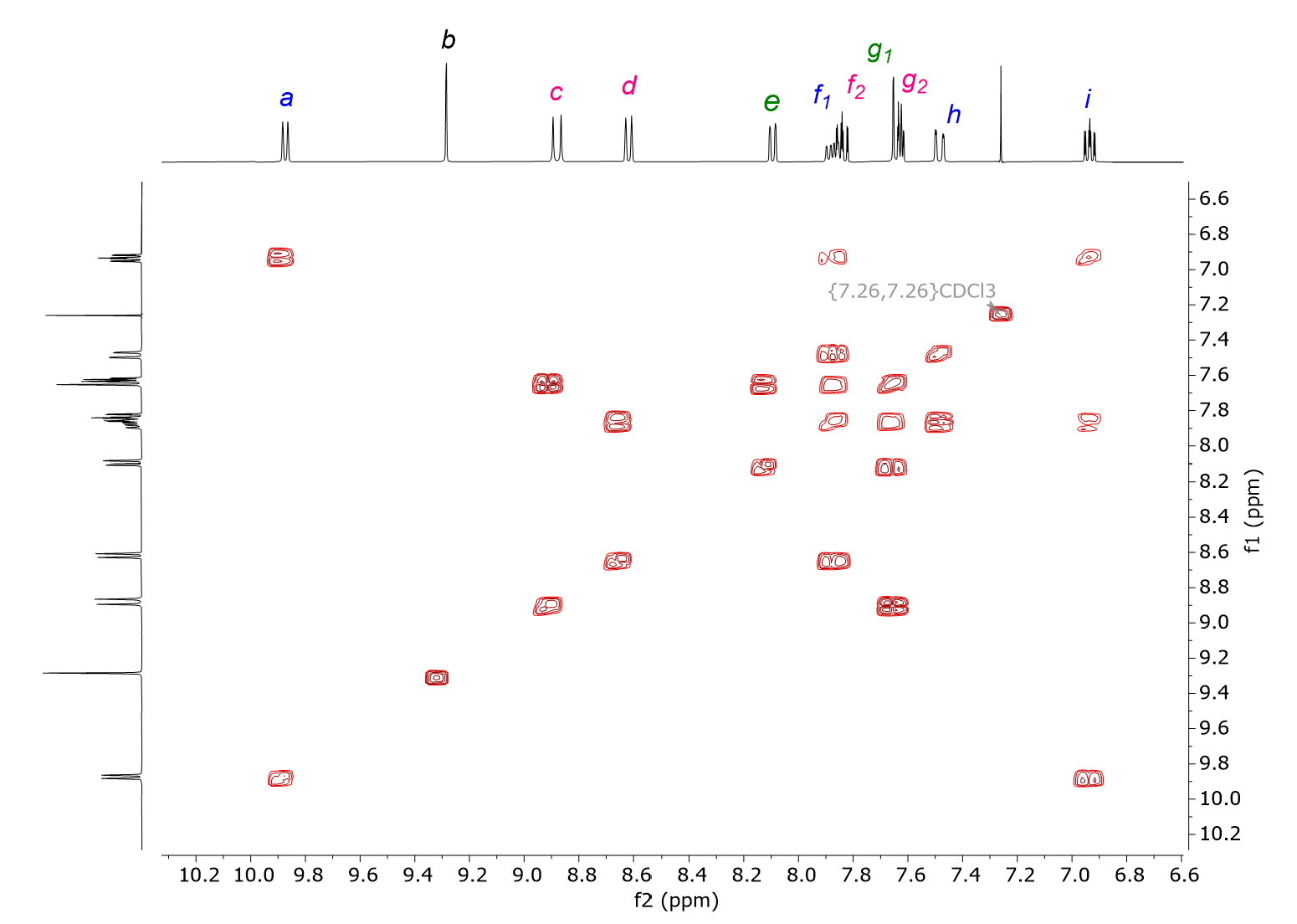


HSQC (400, 100.5 MHz, CDCl_3_)


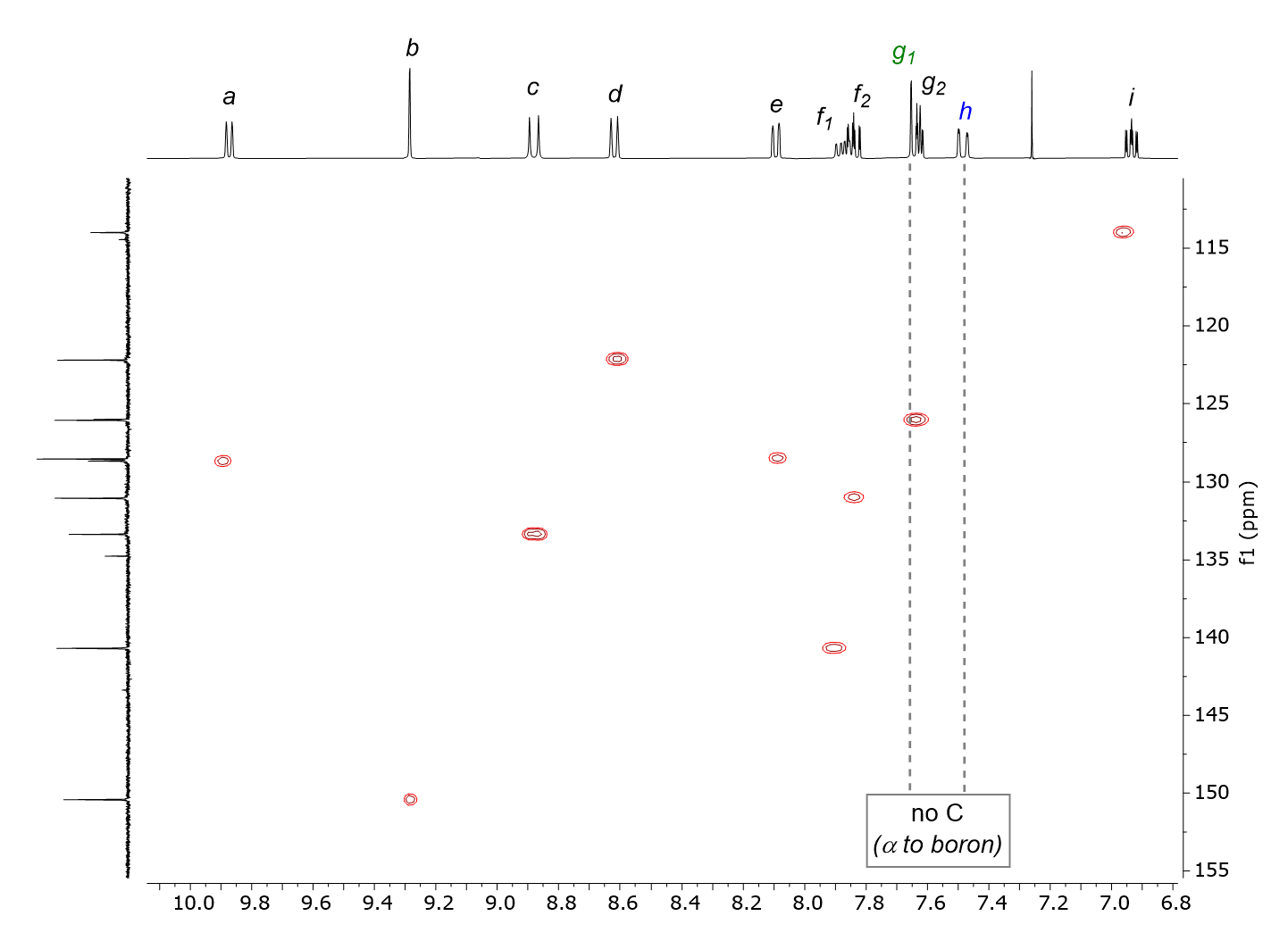


HMBC (600, 151 MHz, CDCl_3_)


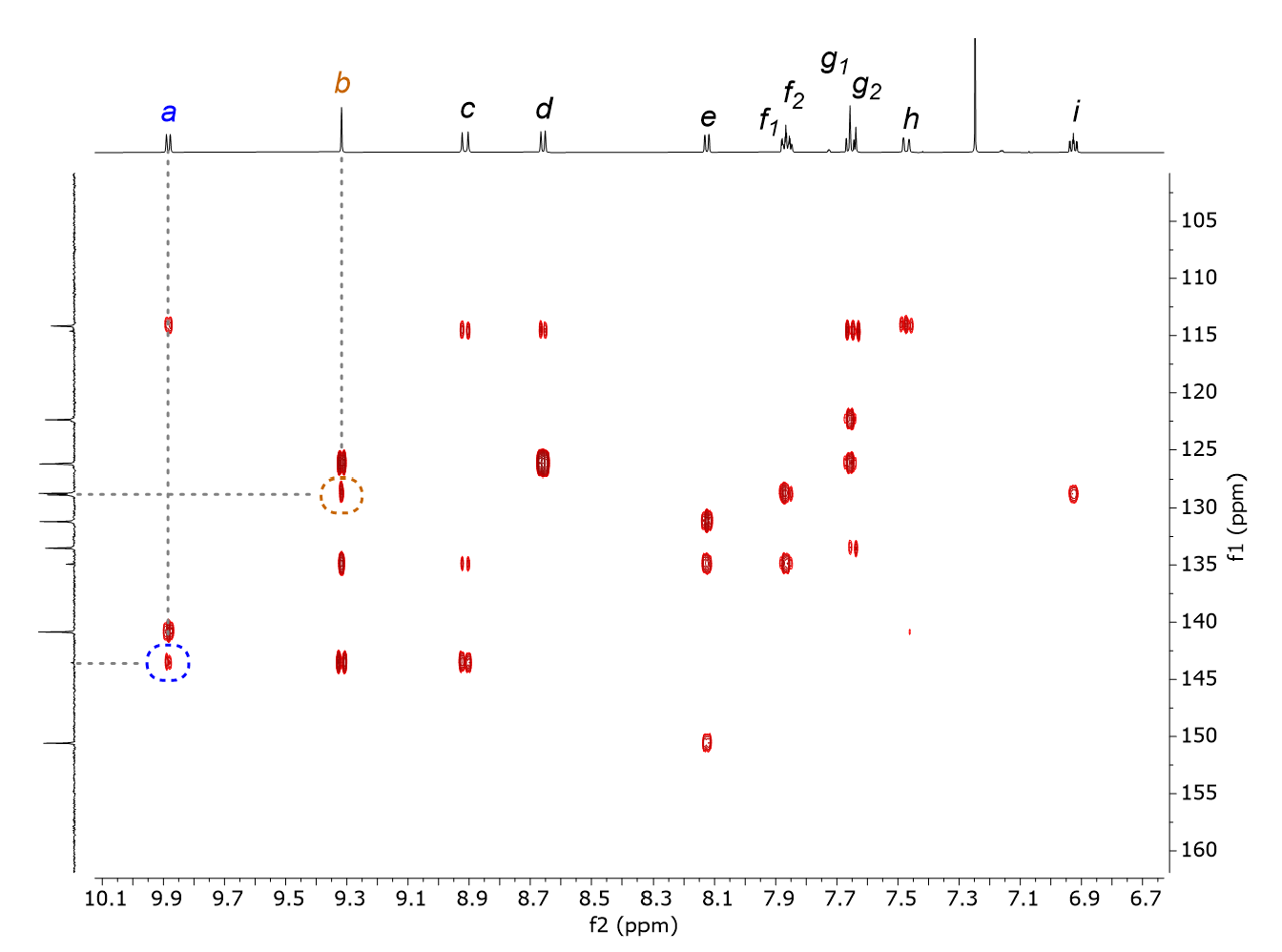


^1^H NMR (400 MHz, CDCl_3_)


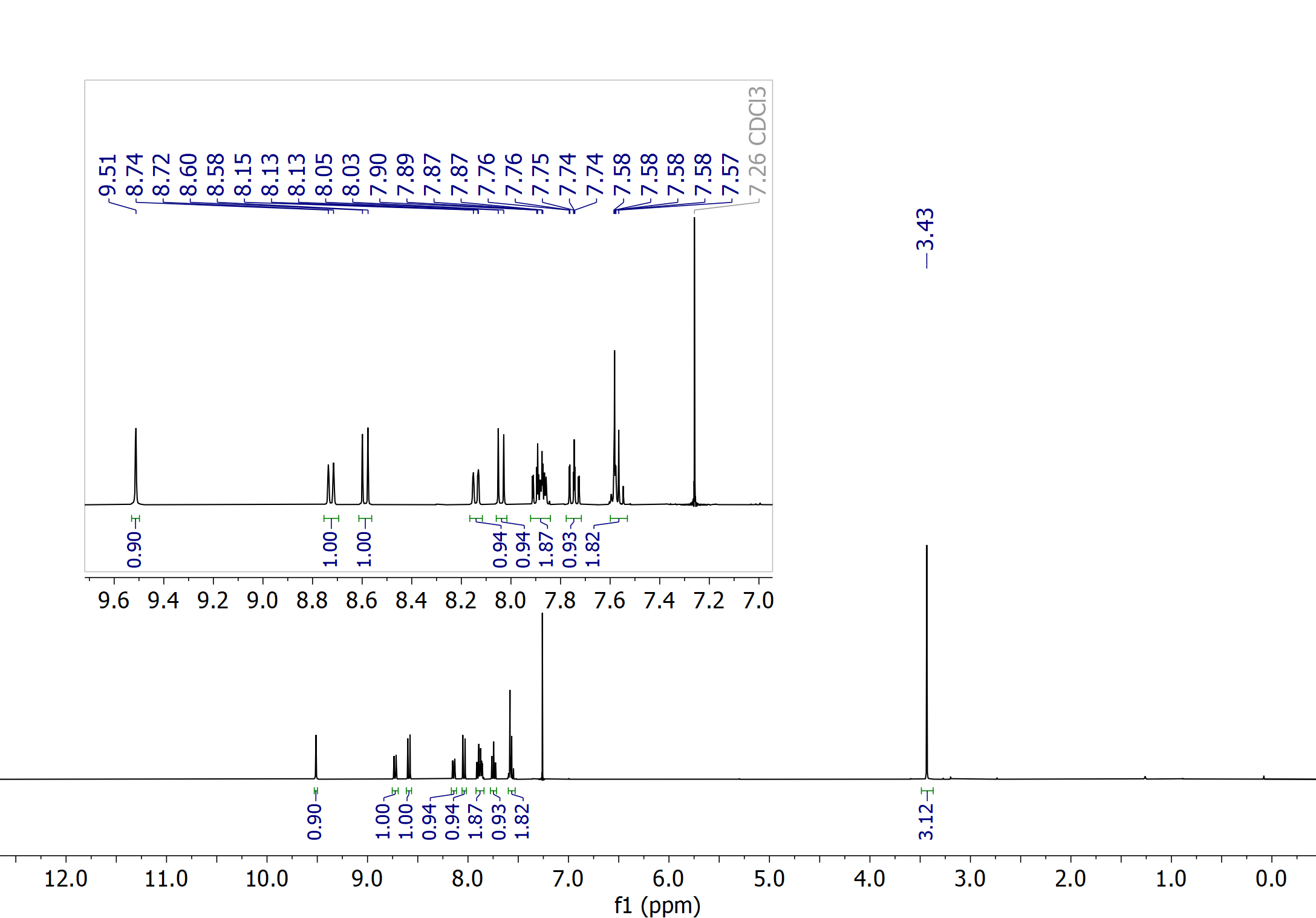


^13^C NMR (100.5 MHz, CDCl_3_)


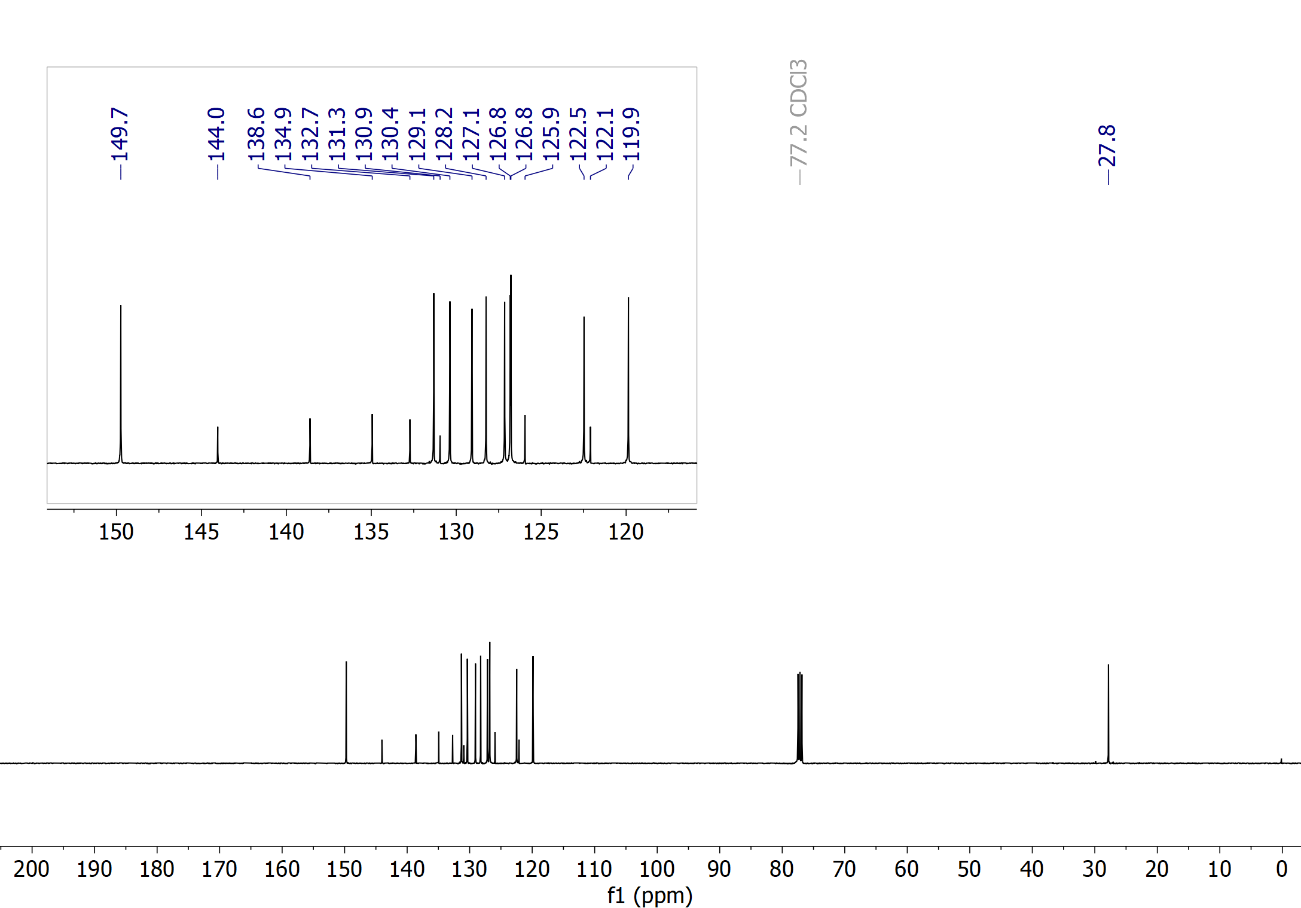


^1^H NMR (400 MHz, CDCl_3_)


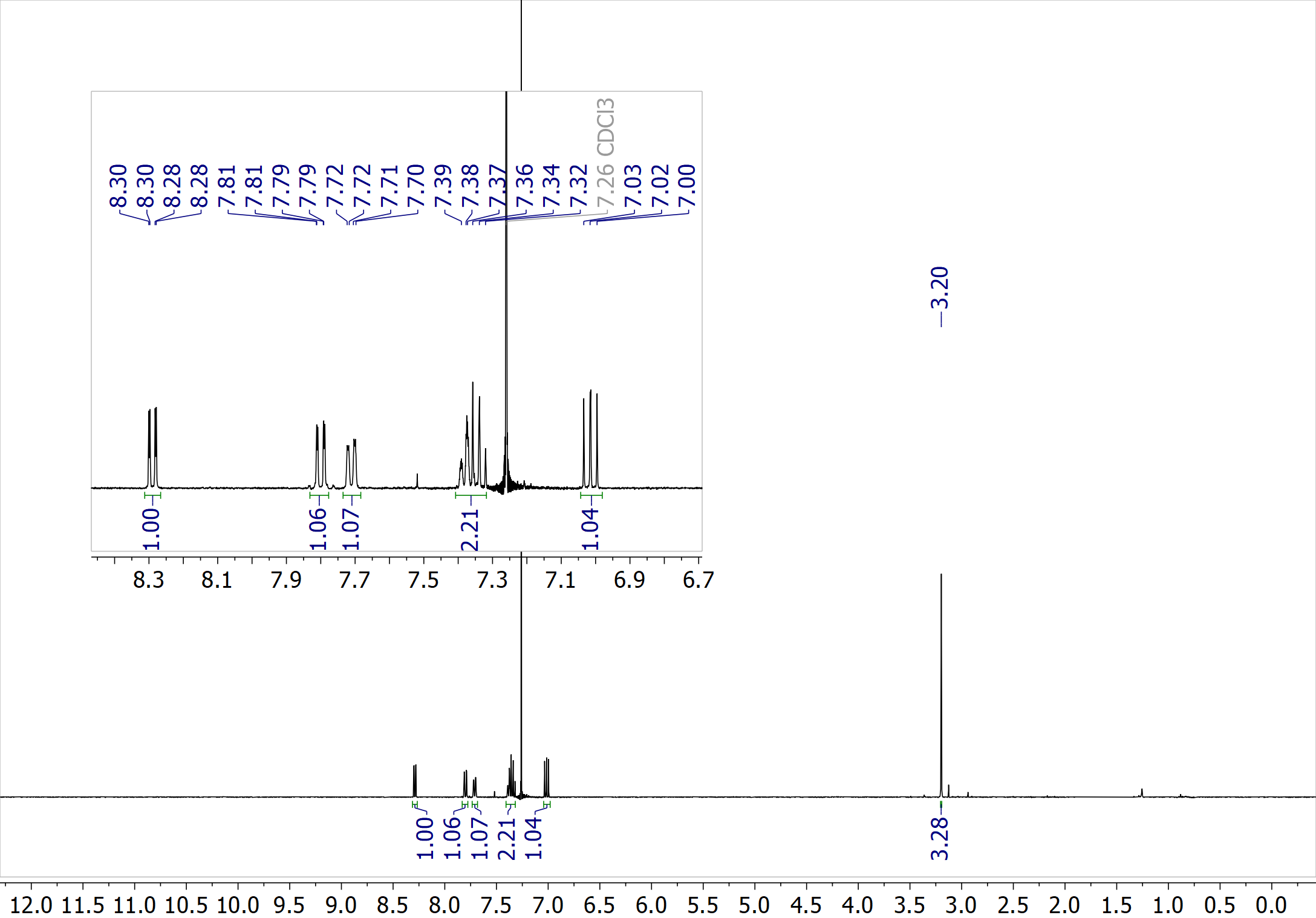


^1^H NMR (400 MHz, CDCl_3_)


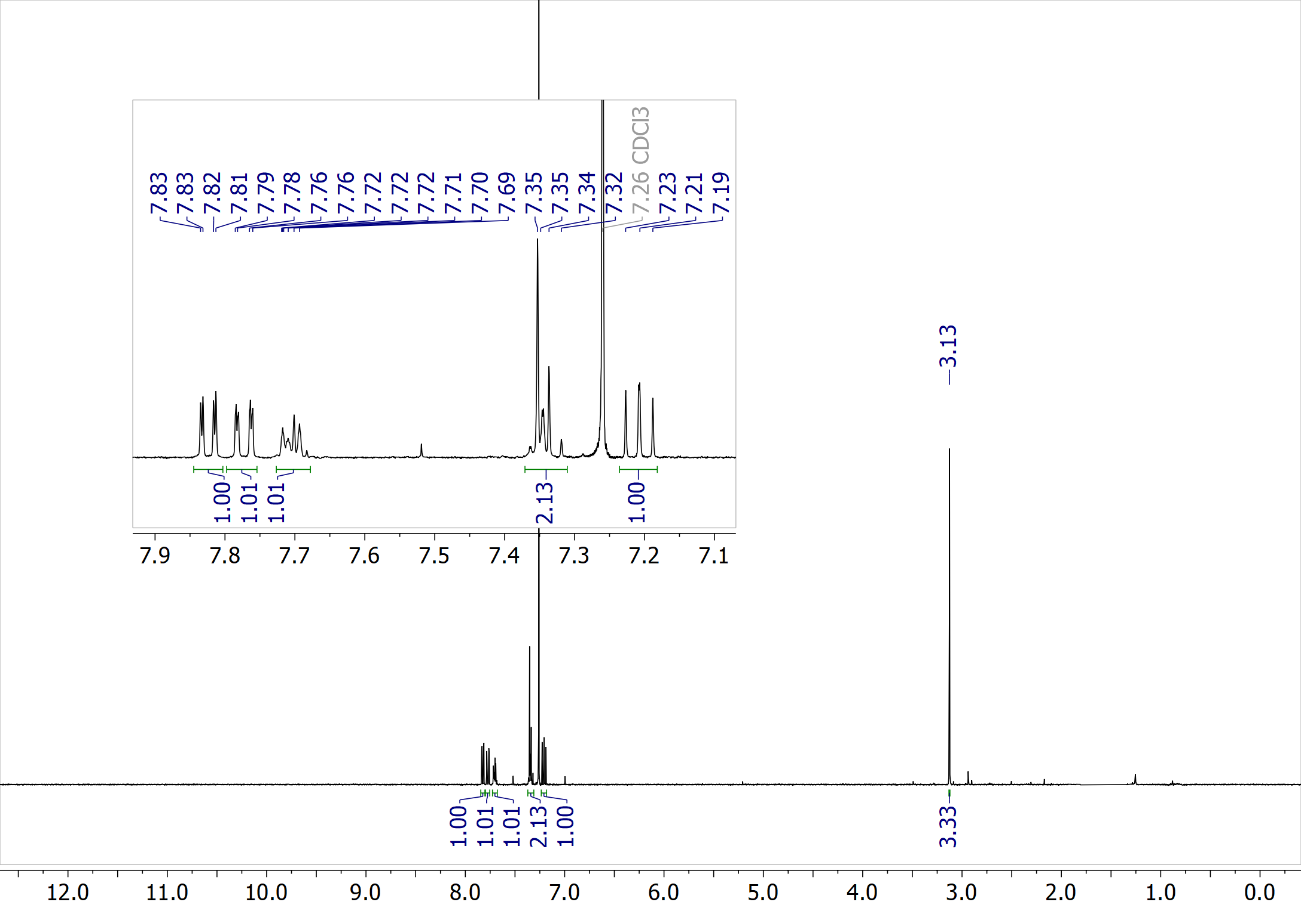


^1^H NMR (400 MHz, CDCl_3_)


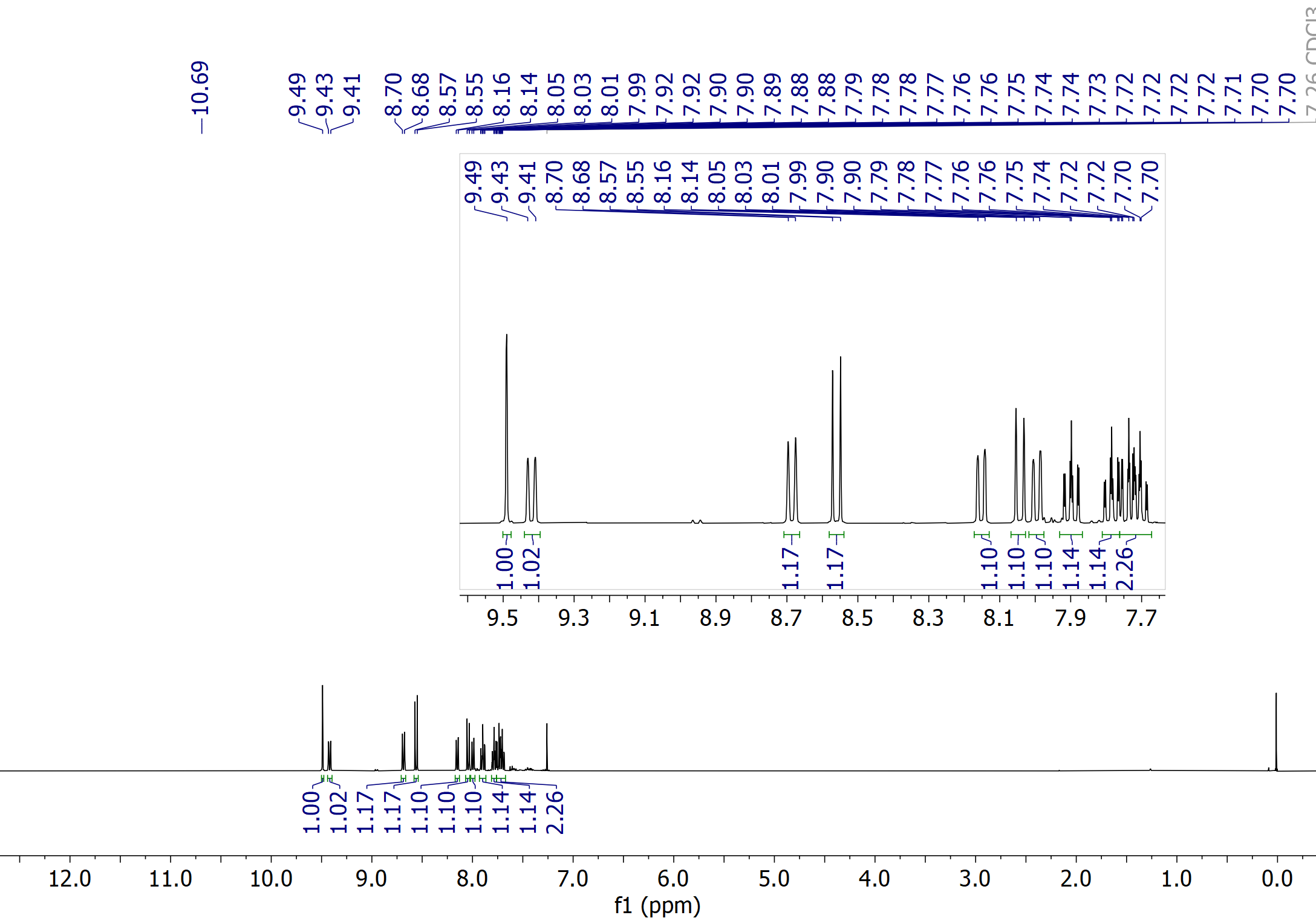


^13^C NMR (100.5 MHz, CDCl_3_)


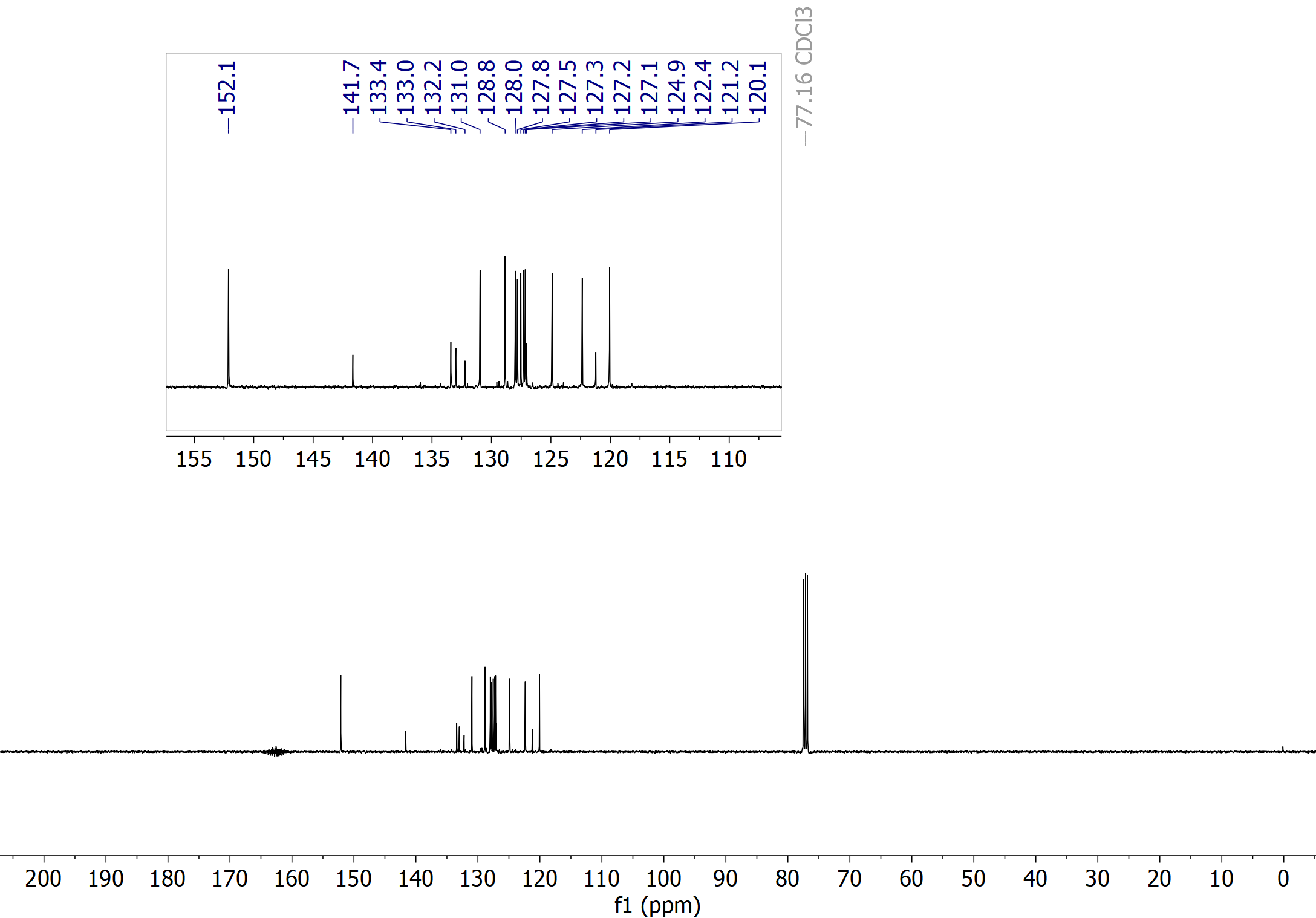


^1^H NMR (400 MHz, CDCl_3_)


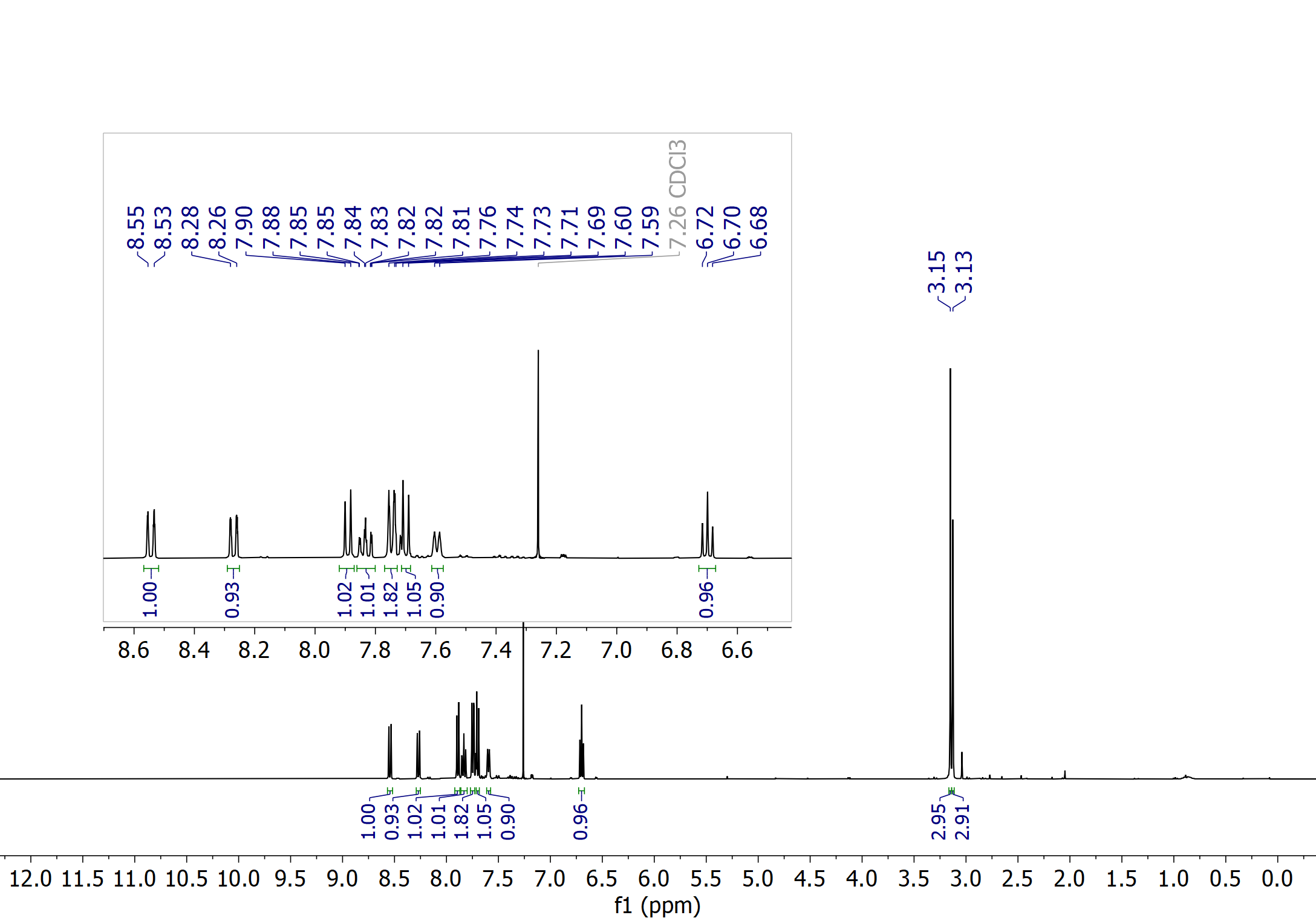


^13^C NMR (100.5 MHz, CDCl_3_)


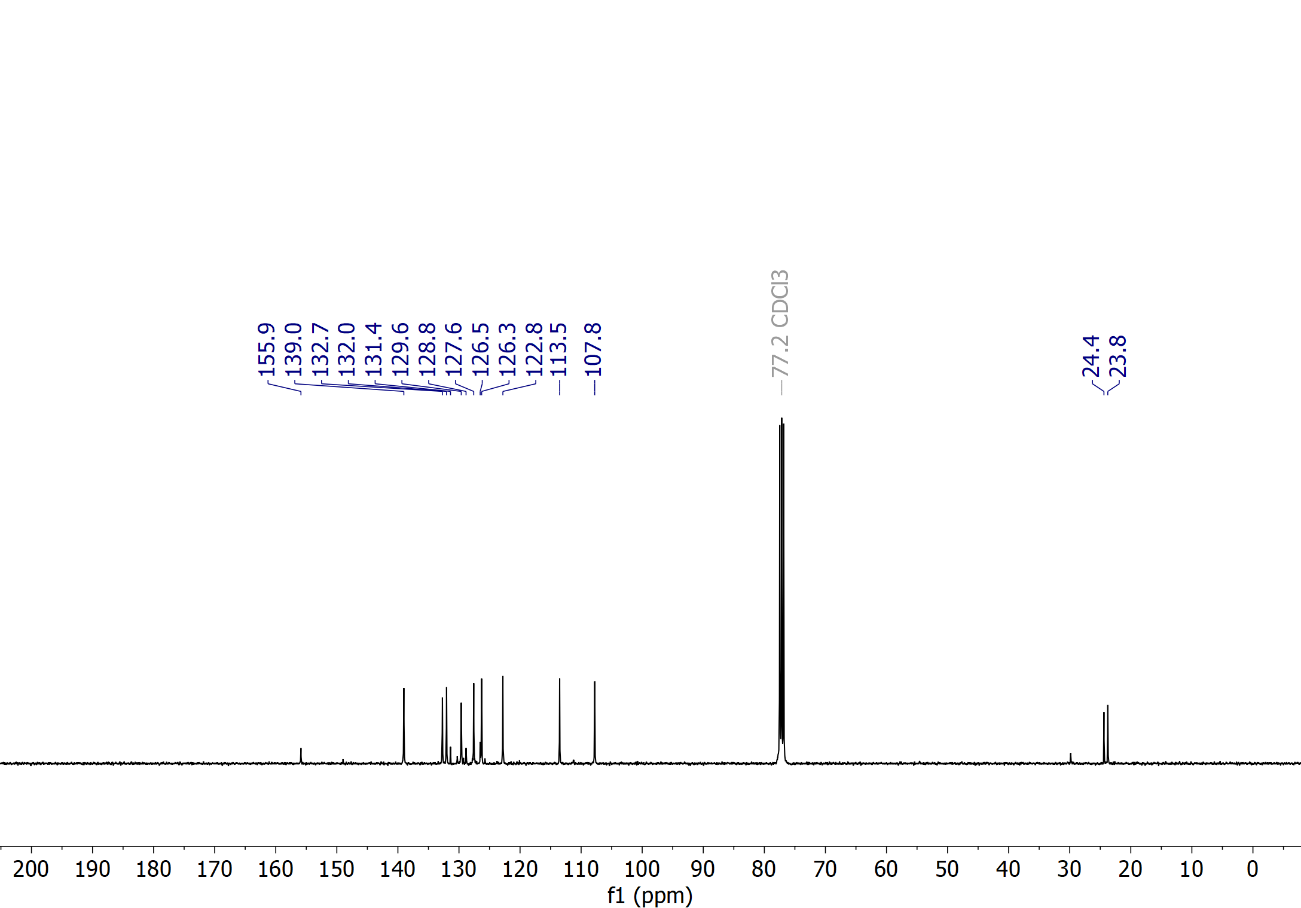


^11^B NMR (128 MHz, DMSO-*d*_6_)


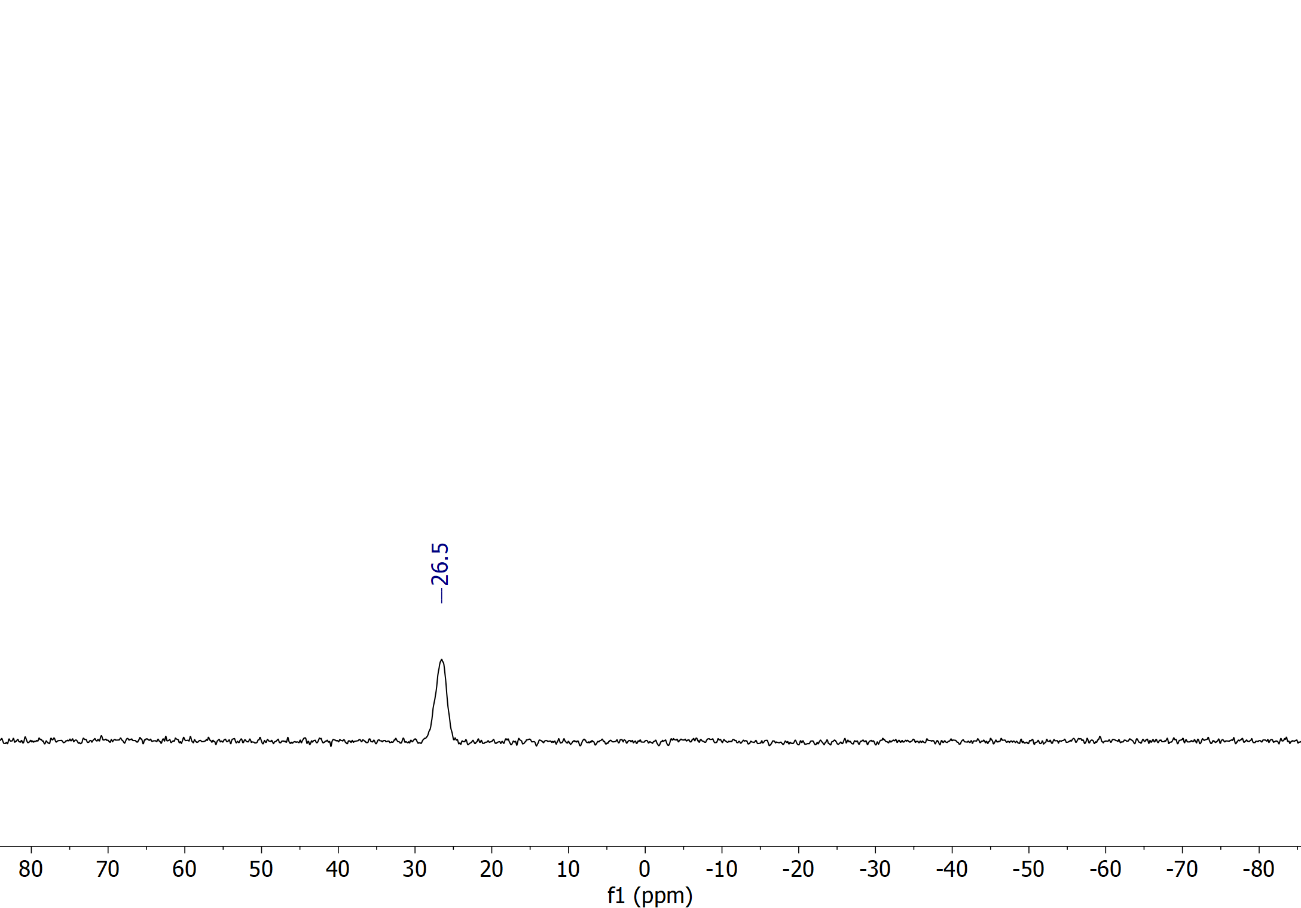


^1^H NMR (400 MHz, CDCl_3_)


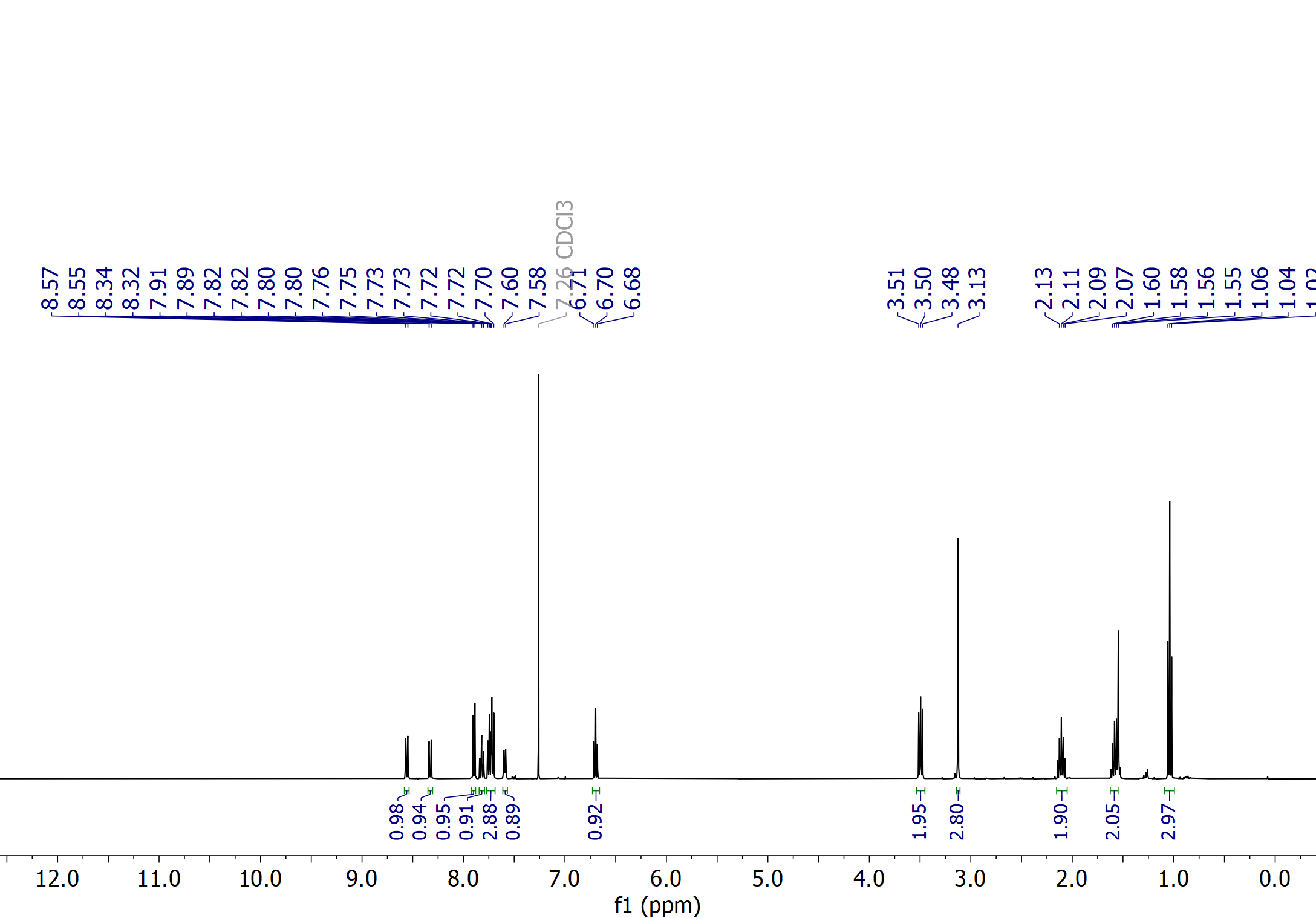


^13^C NMR (100.5 MHz, CDCl_3_)


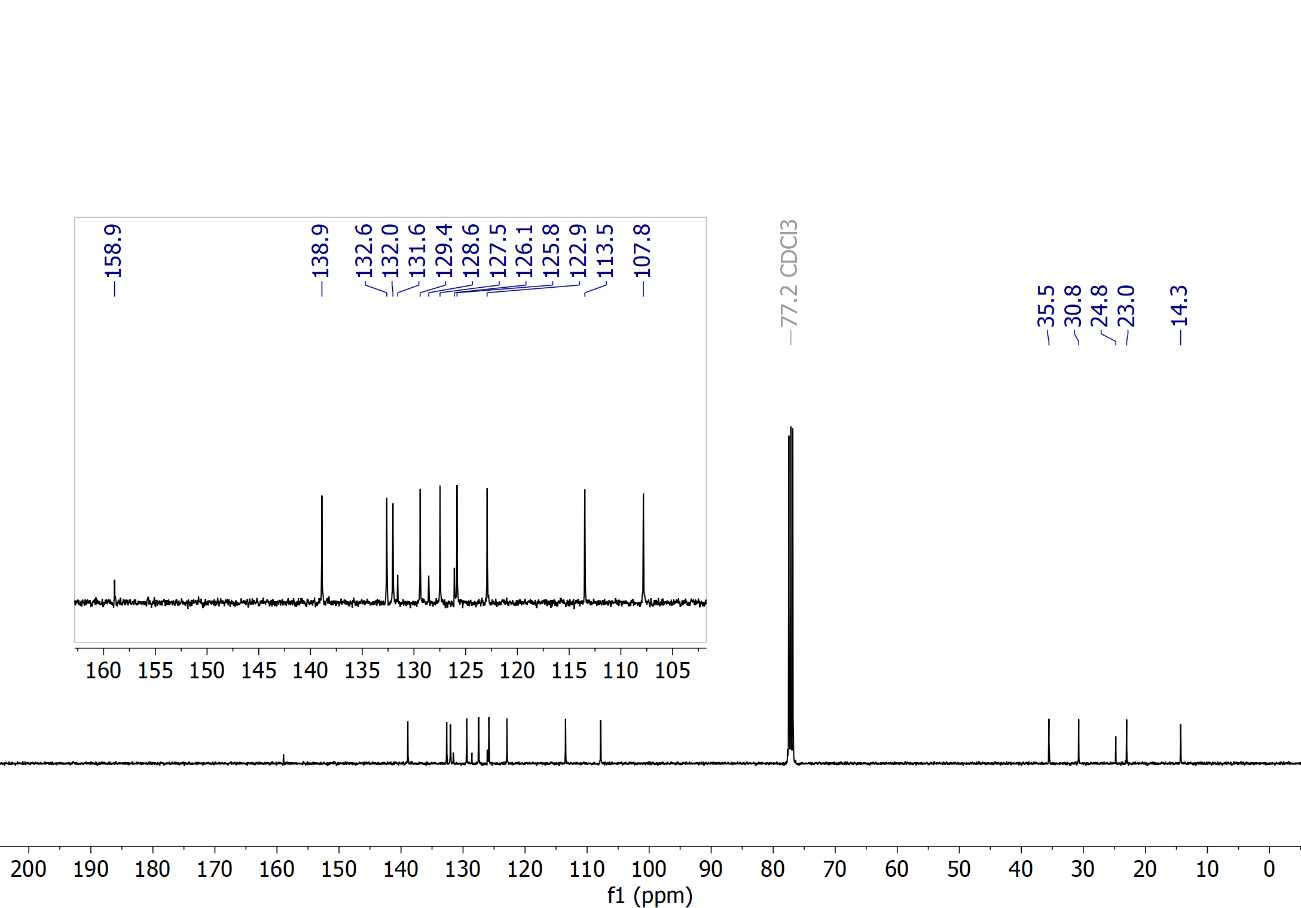


^11^B NMR (128 MHz, CDCl_3_)


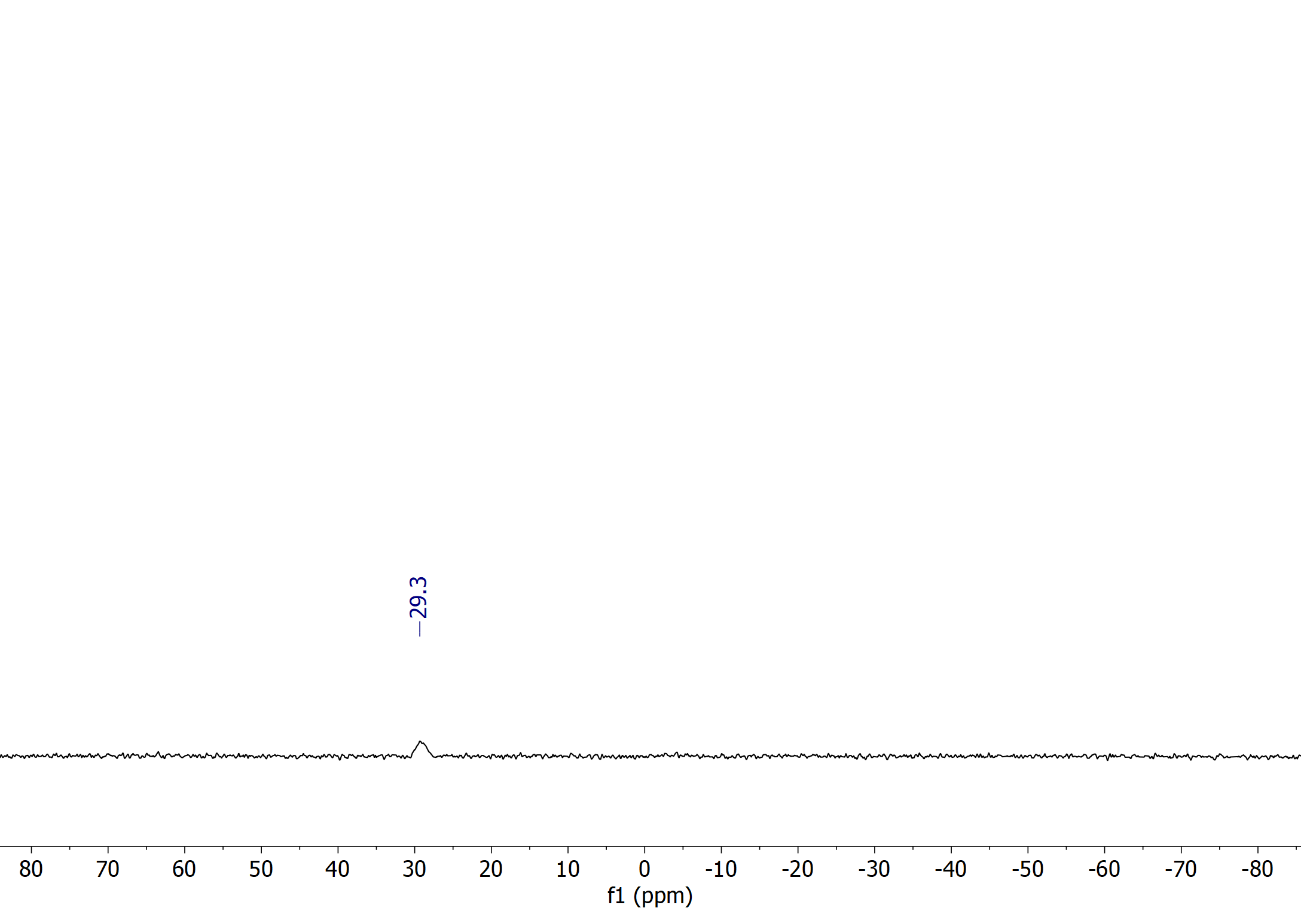


^1^H NMR (400 MHz, CDCl_3_)


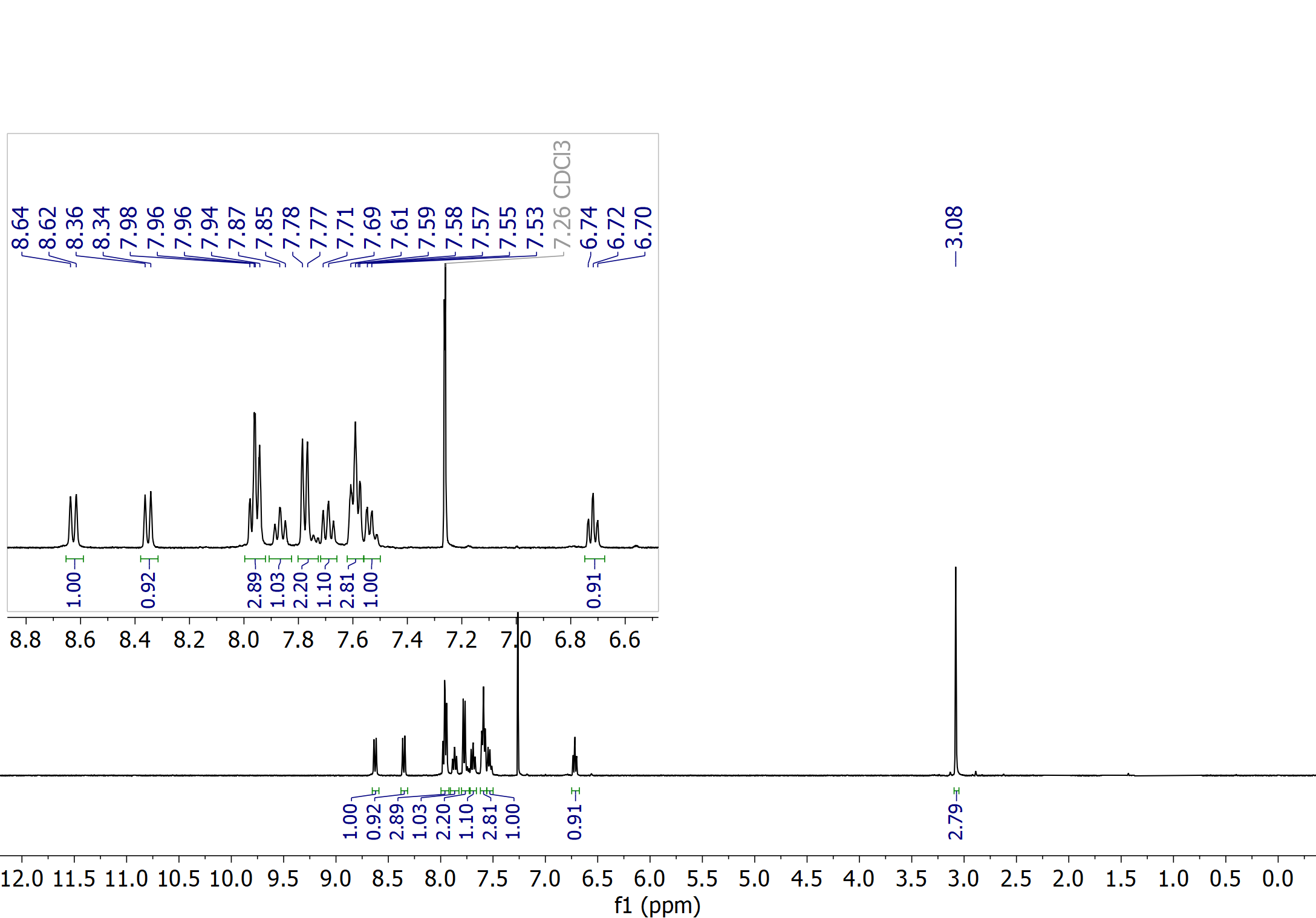


^13^C NMR (100.5 MHz, CDCl_3_)


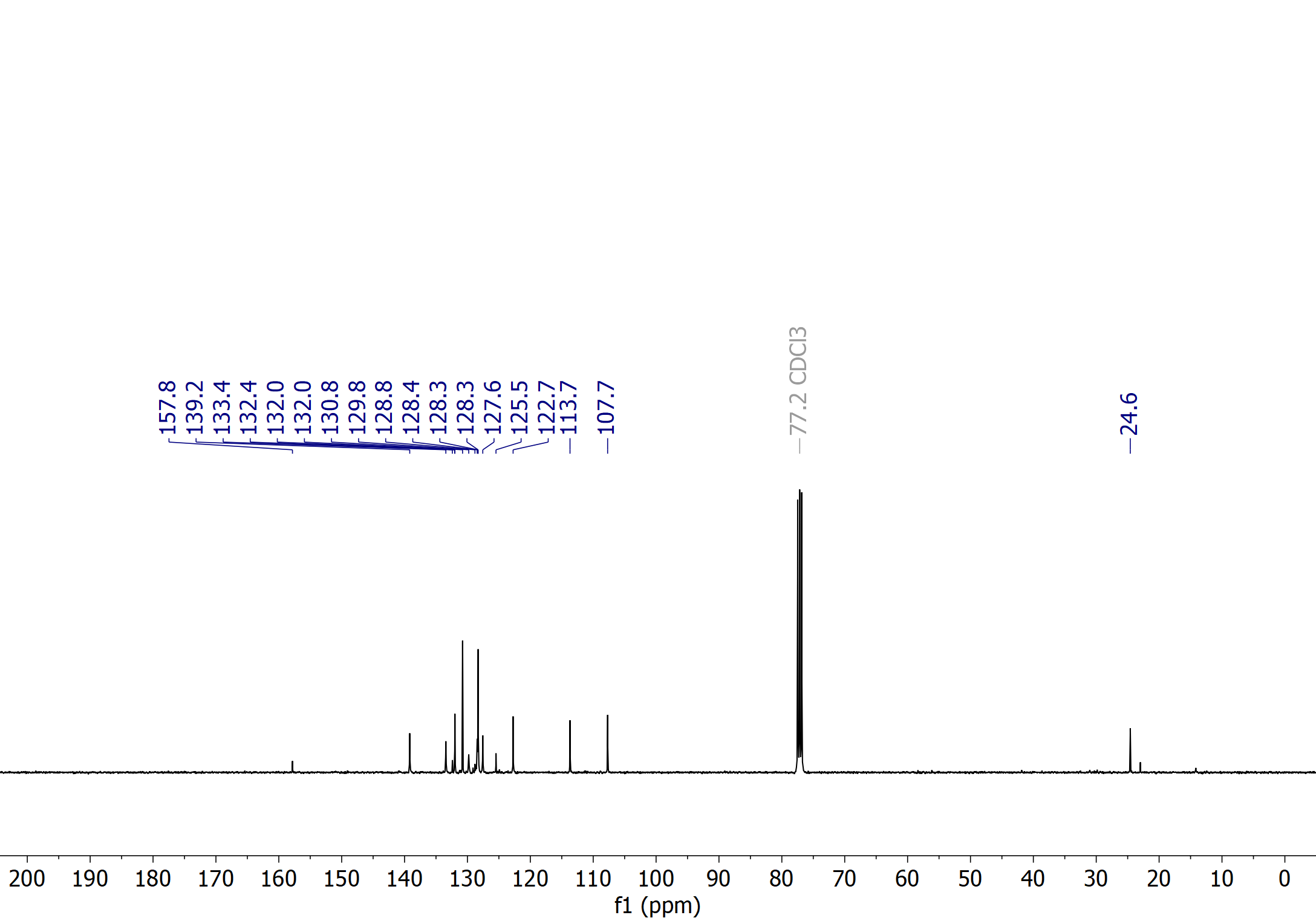


^11^B NMR (128 MHz, CDCl_3_)


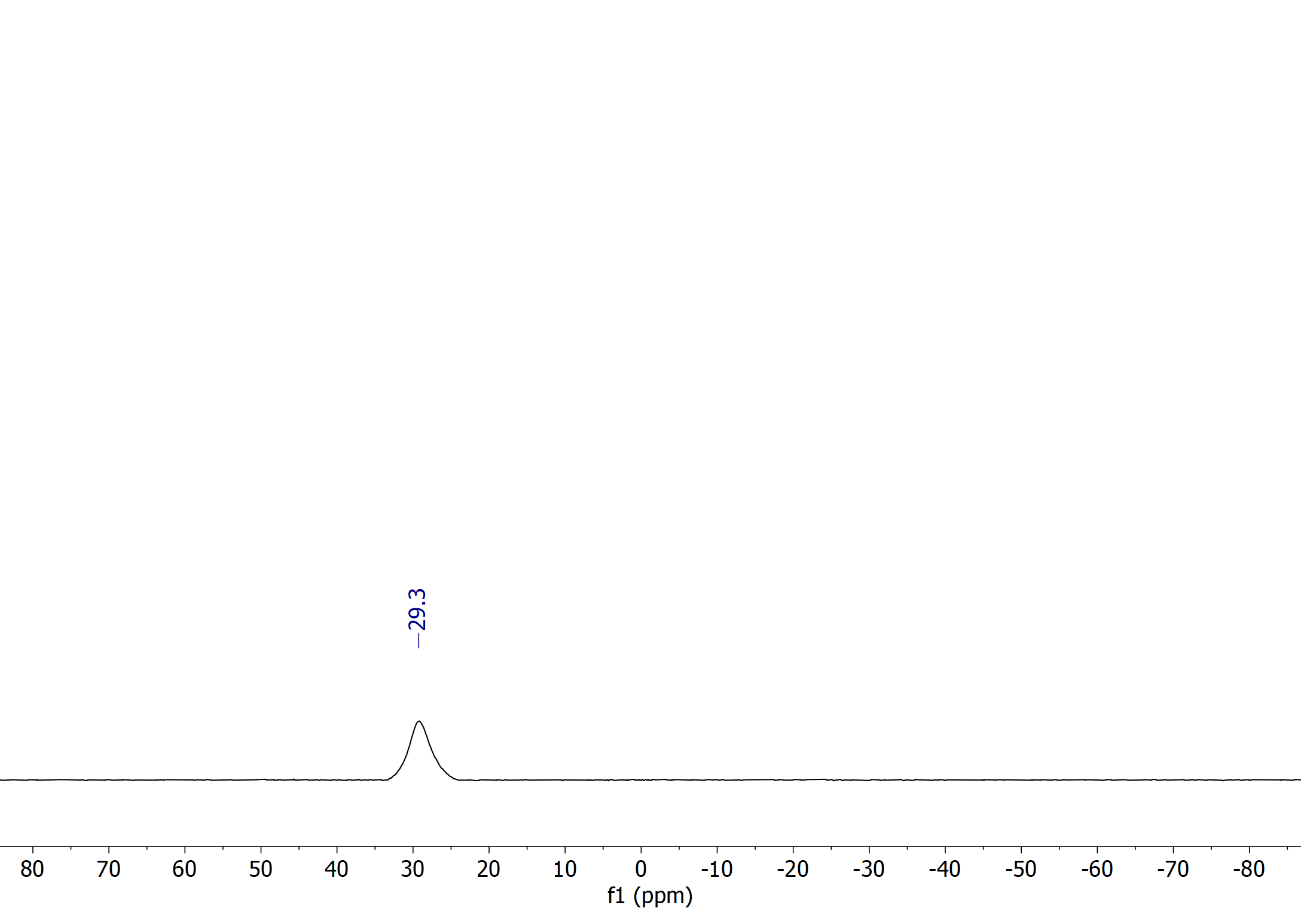


^1^H NMR (600 MHz, CDCl_3_)


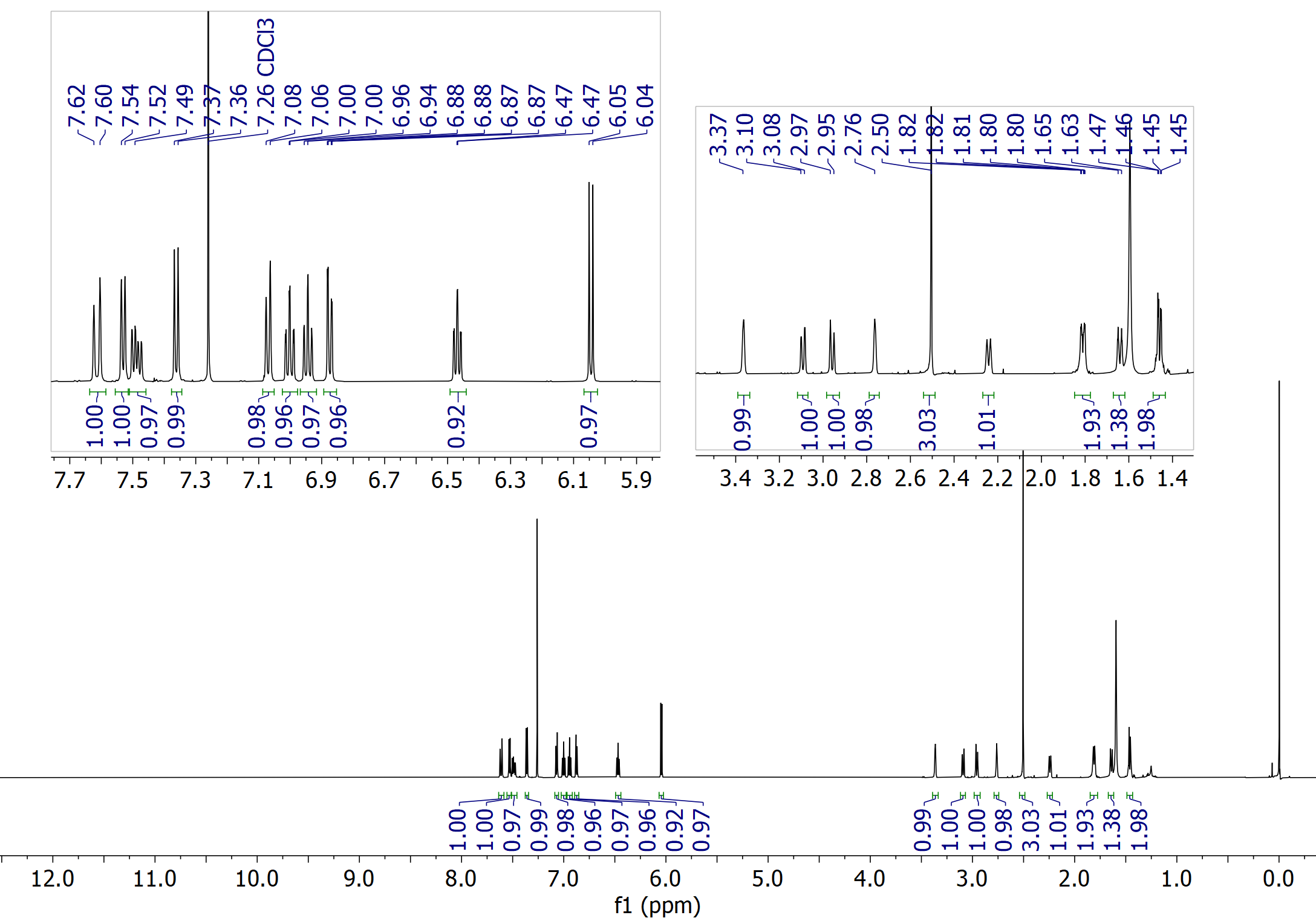


^13^C NMR (100.5 MHz, CDCl_3_)


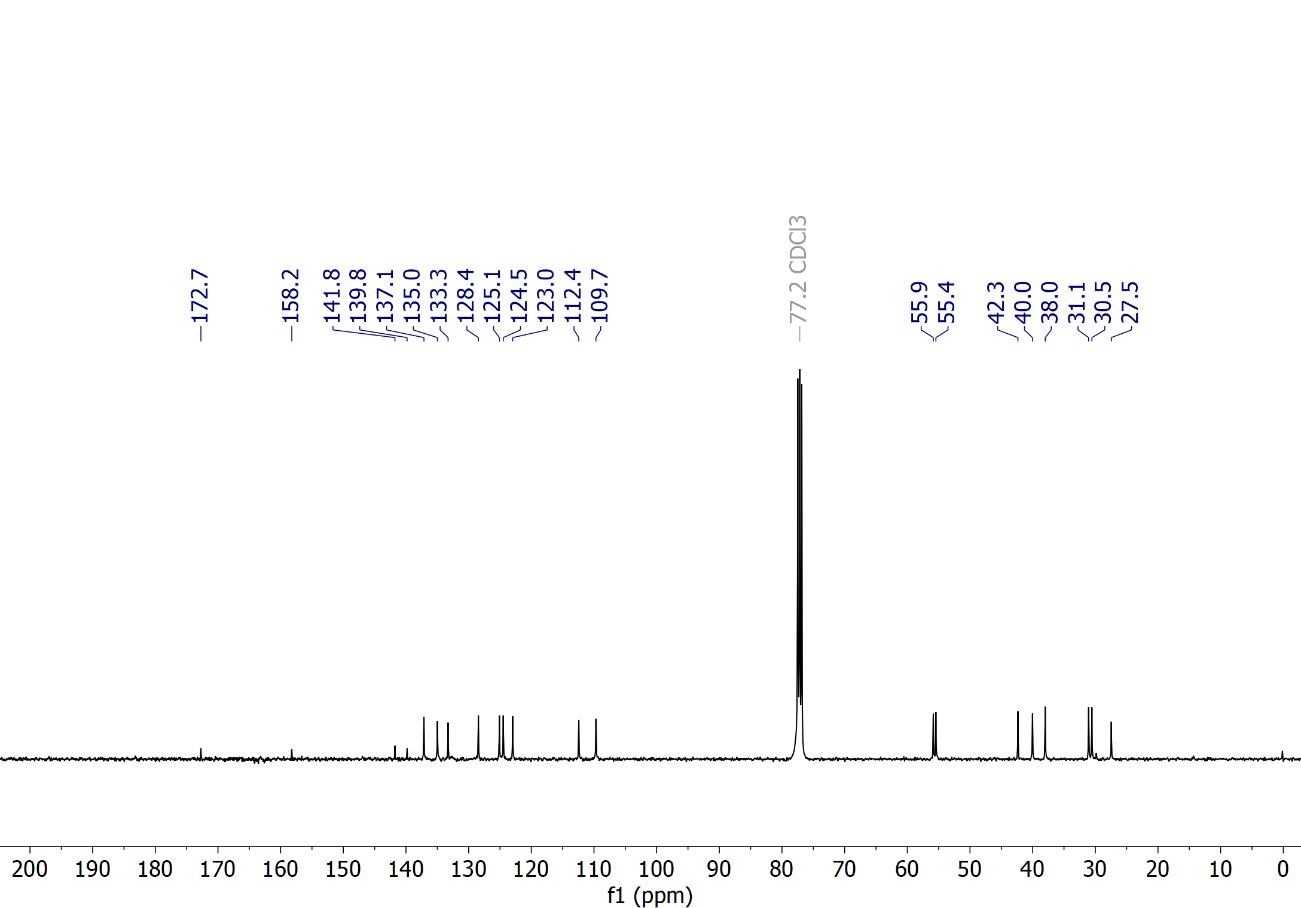


^11^B NMR (193 MHz, CDCl_3_)


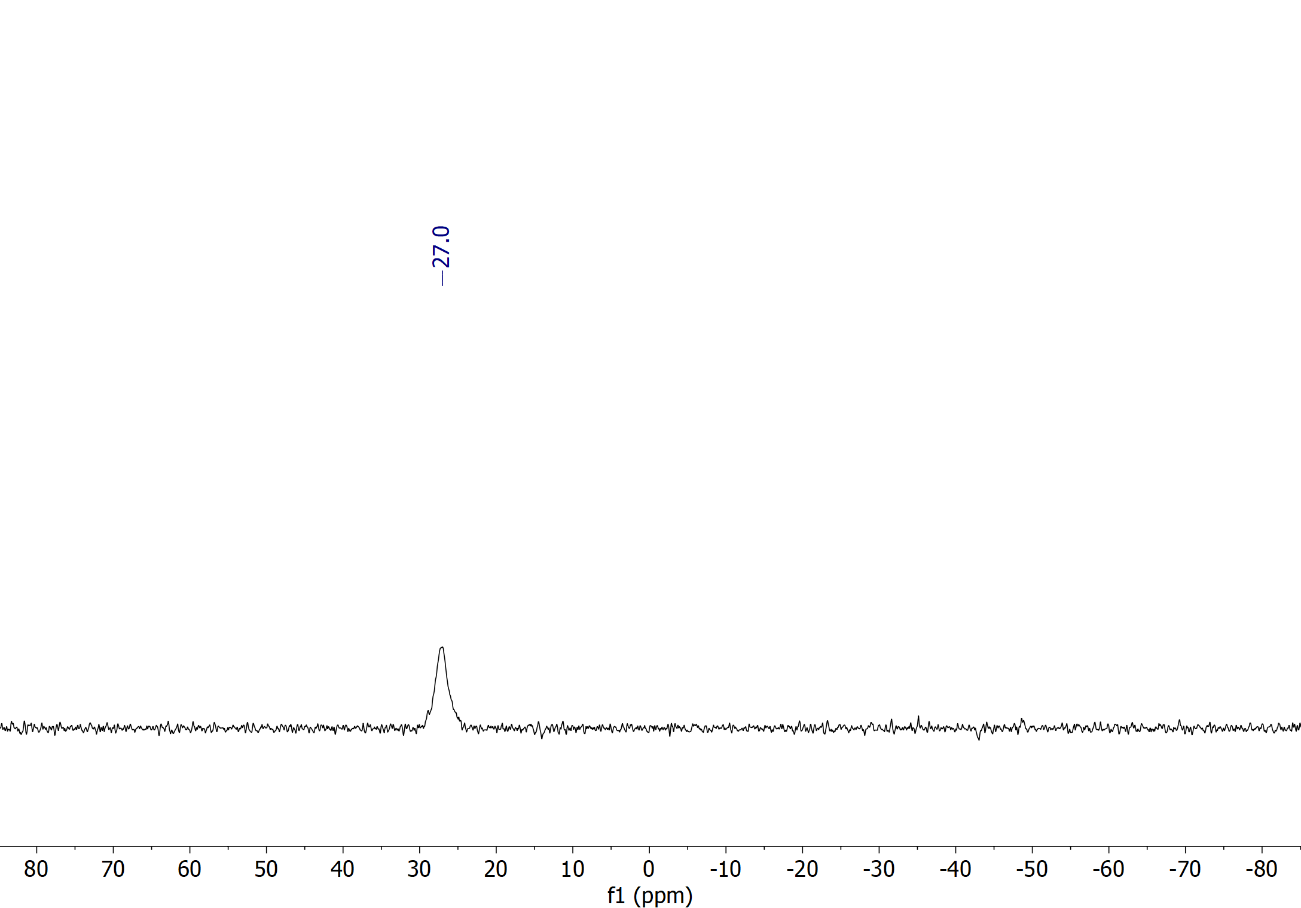


COSY (600, 600 MHz, CDCl_3_)


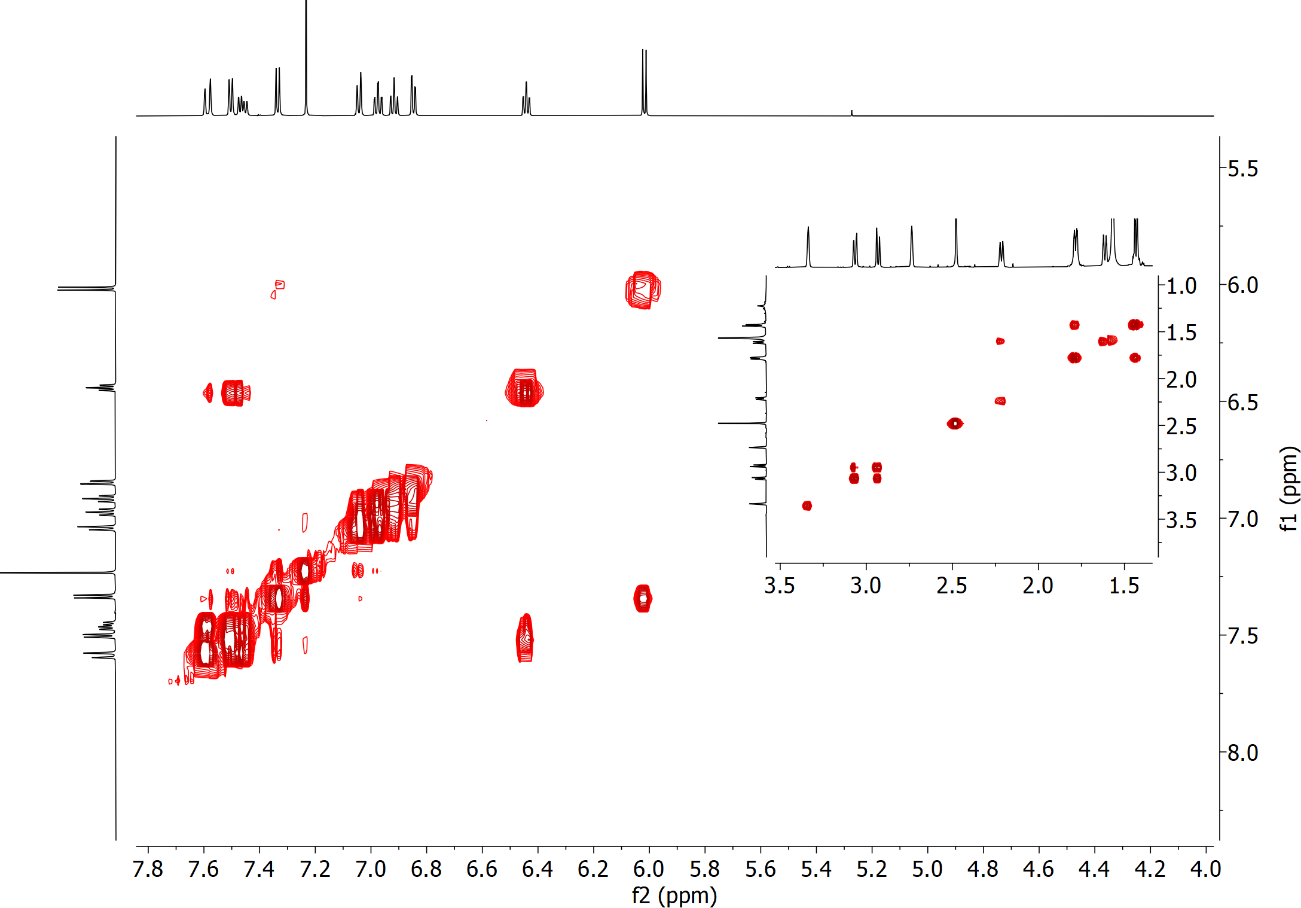


HSQC (600, 151 MHz, CDCl_3_)


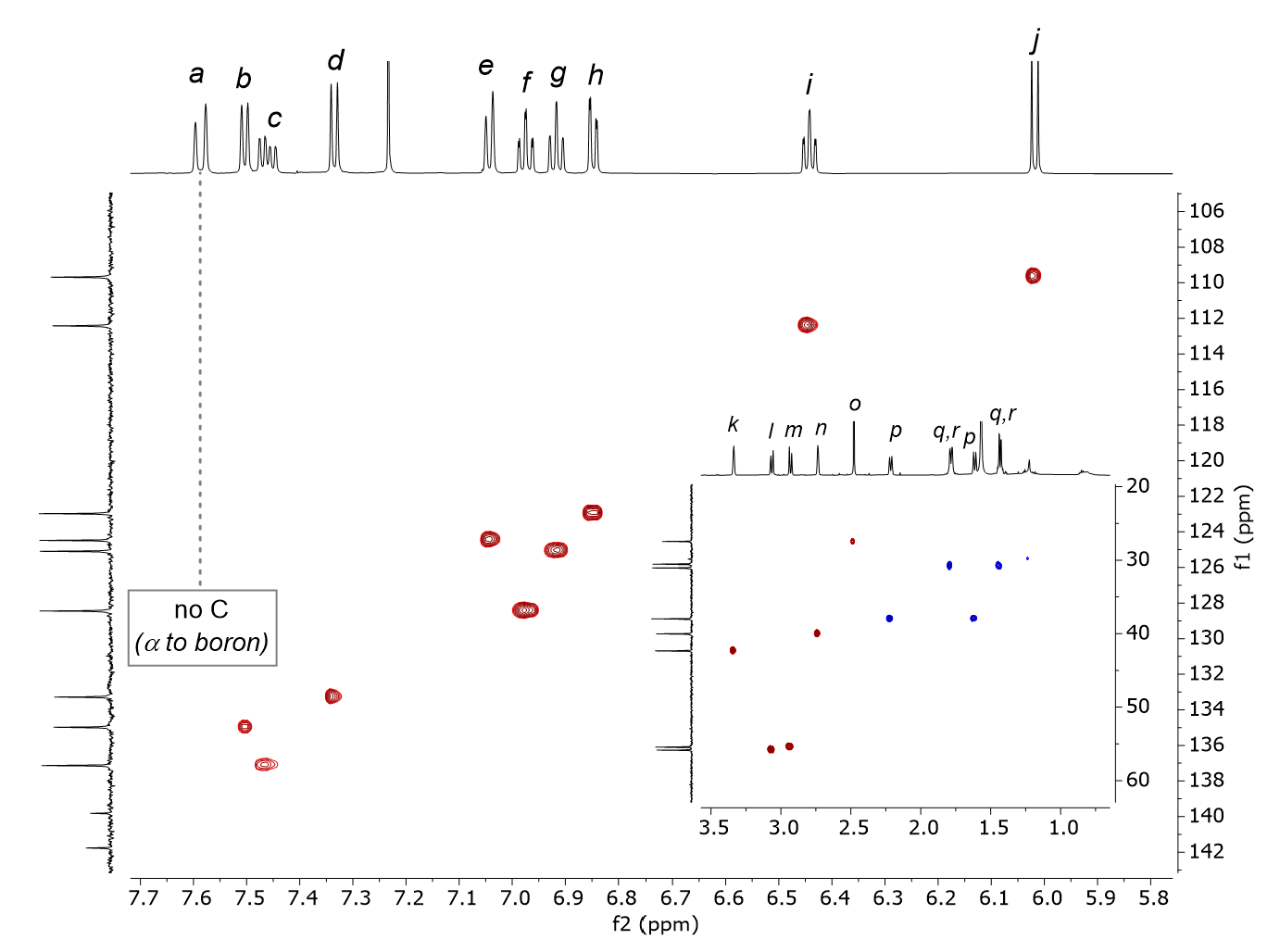


HMBC (600, 151 MHz, CDCl_3_)


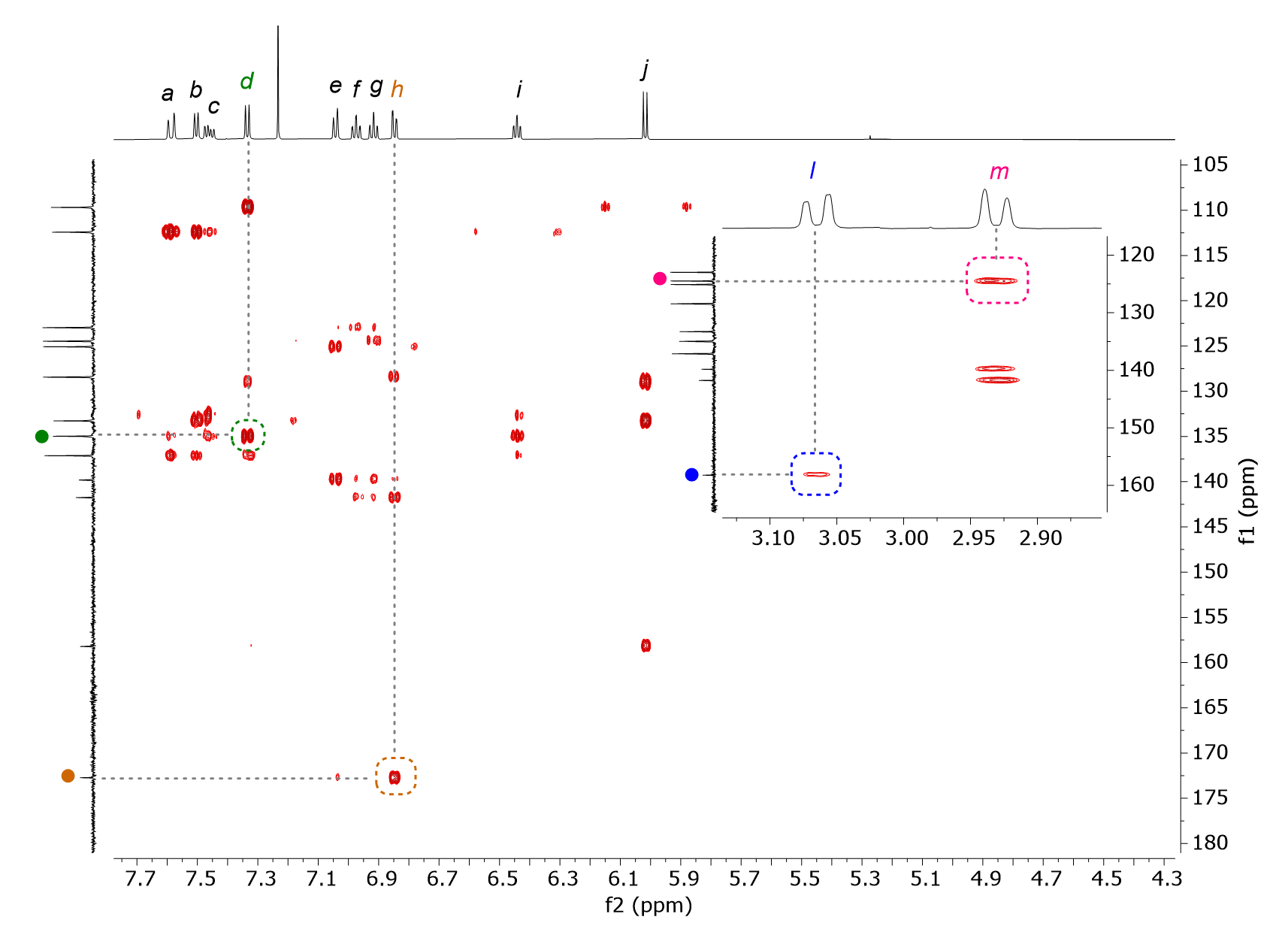


NOESY (600, 600 MHz, CDCl_3_)


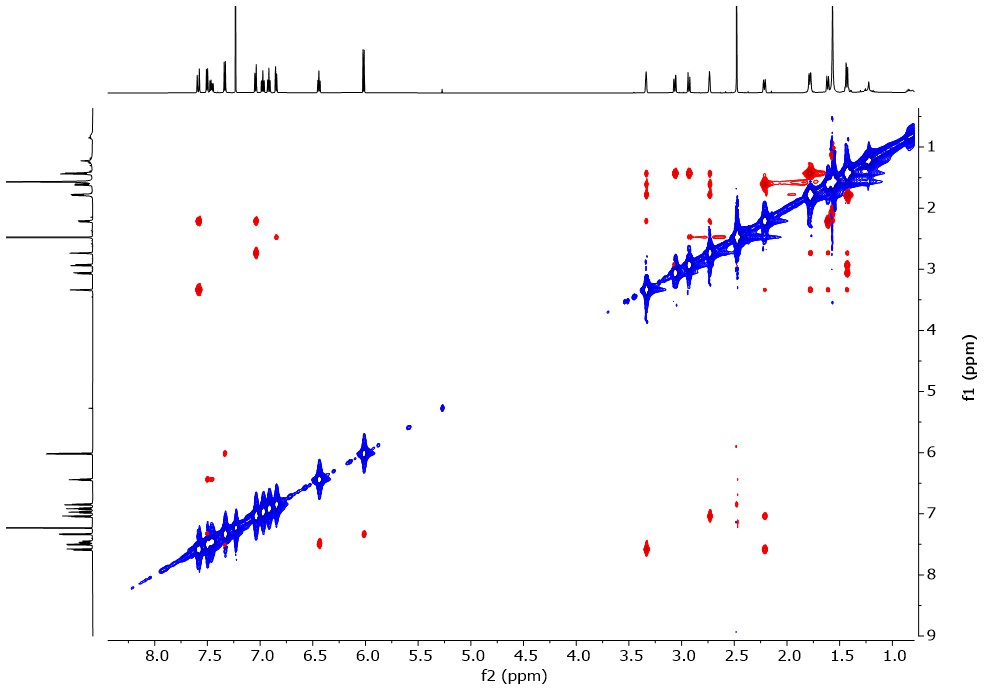


NOESY (600, 600 MHz, CDCl_3_, zoom)


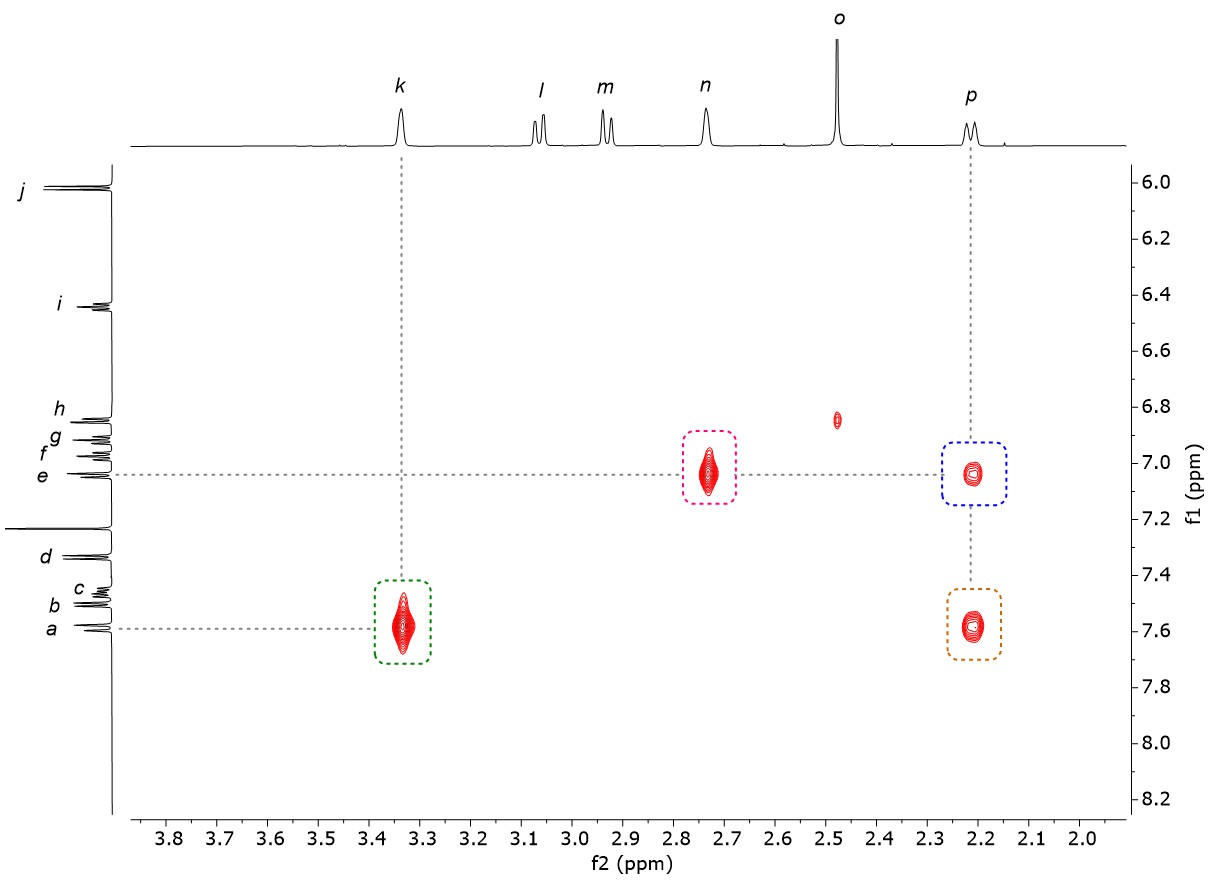


^1^H NMR (400 MHz, CDCl_3_)


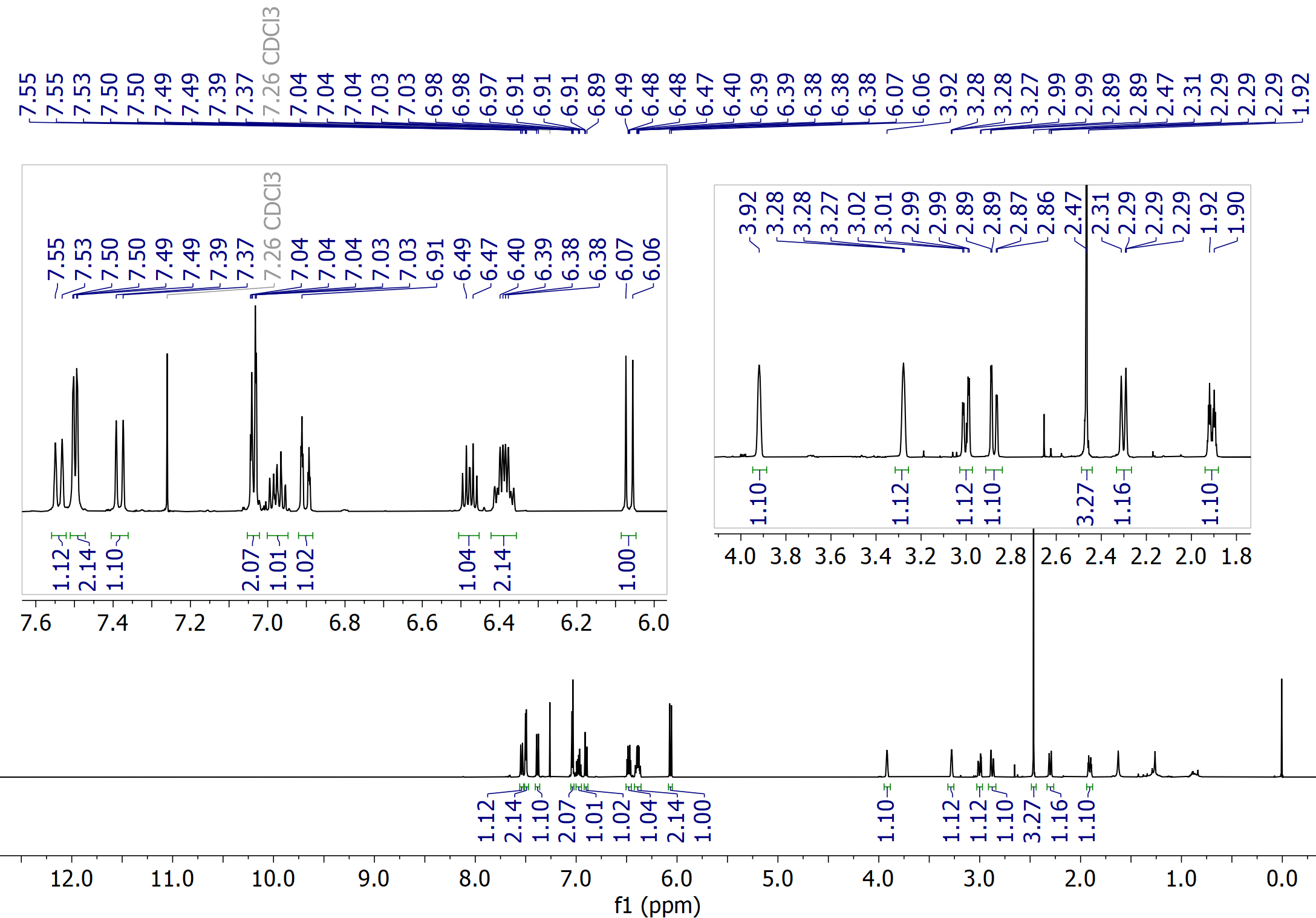


^13^C NMR (100.5 MHz, CDCl_3_)

^11^B NMR (128 MHz, CDCl_3_)

COSY (400, 400 MHz, CDCl_3_)

HSQC (400, 100 MHz, CDCl_3_)

HMBC (600, 151 MHz, CDCl_3_)

NOESY (600, 600 MHz, CDCl_3_)

NOESY (600, 600 MHz, CDCl_3_, zoom)

^1^H NMR (400 MHz, CDCl_3_)

^13^C NMR (100.5 MHz, CDCl_3_)

^11^B NMR (193 MHz, CDCl_3_)

^1^H NMR (400 MHz, CDCl_3_)

^13^C NMR (100.5 MHz, CDCl_3_)

^11^B NMR (128 MHz, CDCl_3_)

COSY (400, 400 MHz, CDCl_3_)

HSQC (400, 100.5 MHz, CDCl_3_)

HMBC (400, 100.5 MHz, CDCl_3_)

^1^H NMR (400 MHz, CDCl_3_)

^13^C NMR (100.5 MHz, CDCl_3_)

^11^B NMR (128 MHz, CDCl_3_)

COSY (400, 400 MHz, CDCl_3_)

HSQC (400, 100.5 MHz, CDCl_3_)

HMBC (400, 100.5 MHz, CDCl_3_)

^1^H NMR (400 MHz, CDCl_3_)

^13^C NMR (100.5 MHz, CDCl_3_)

^11^B NMR (128 MHz, CDCl_3_)

COSY (400, 400 MHz, CDCl_3_)

HSQC (400, 100.5 MHz, CDCl_3_)

HMBC (400, 100.5 MHz, CDCl_3_)

^1^H NMR (400 MHz, CDCl_3_)

^13^C NMR (100.5 MHz, CDCl_3_)

^11^B NMR (128 MHz, CDCl_3_)

^1^H NMR (400 MHz, CDCl_3_)

^13^C NMR (151 MHz, CDCl_3_)

^11^B NMR (128 MHz, CDCl_3_)

^19^F NMR (376 MHz, CDCl_3_)

^1^H NMR (400 MHz, CDCl_3_)

^13^C NMR (100.5 MHz, CDCl_3_)

^11^B NMR (128 MHz, CDCl_3_)

^1^H NMR (400 MHz, DMSO-*d*_6_)

^13^C NMR (100.5 MHz, DMSO-*d*_6_)

^11^B NMR (128 MHz, CDCl_3_)

^1^H NMR (400 MHz, CDCl_3_)

^13^C NMR (100.5 MHz, CDCl_3_)

^11^B NMR (128 MHz, CDCl_3_)

^1^H NMR (400 MHz, CD_2_Cl_2_)

^13^C NMR (100.5 MHz, CD_2_Cl_2_)

^11^B NMR (128 MHz, CD_2_Cl_2_)

^1^H NMR (400 MHz, CDCl_3_)

^13^C NMR (100.5 MHz, CDCl_3_)

^11^B NMR (128 MHz, CDCl_3_)

^1^H NMR (400 MHz, CDCl_3_)

^13^C NMR (151 MHz, CDCl_3_)

^11^B NMR (193 MHz, CDCl_3_)

^1^H NMR (400 MHz, CDCl_3_)

^13^C NMR (100.5 MHz, CDCl_3_)

^11^B NMR (128 MHz, CDCl_3_)

^1^H NMR (400 MHz, CDCl_3_)

^13^C NMR (100.5 MHz, CDCl_3_)

^1^H NMR (400 MHz, CDCl_3_)

^13^C NMR (100.5 MHz, CDCl_3_)

^1^H NMR (400 MHz, CDCl_3_)

^13^C NMR (100.5 MHz, CDCl_3_)

^11^B NMR (128 MHz, CDCl_3_)

^1^H NMR (400 MHz, CDCl_3_)

COSY (400, 400 MHz, CDCl_3_)

^1^H NMR (400 MHz, CDCl_3_)

^13^C NMR (100.5 MHz, CDCl_3_)

^11^B NMR (128 MHz, CDCl_3_)

^1^H NMR (400 MHz, CDCl_3_)

^13^C NMR (100.5 MHz, CDCl_3_)

^11^B NMR (128 MHz, CDCl_3_)

^1^H NMR (400 MHz, CDCl_3_)

^13^C NMR (100.5 MHz, CDCl_3_)

^11^B NMR (128 MHz, CDCl_3_)

^1^H NMR (400 MHz, CDCl_3_)

^13^C NMR (151 MHz, CDCl_3_)

^11^B NMR (193 MHz, CDCl_3_)

^1^H NMR (600 MHz, CDCl_3_)

^13^C NMR (151 MHz, CDCl_3_)

^11^B NMR (193 MHz, CDCl_3_)

COSY (600, 600 MHz, CDCl_3_)

HSQC (600, 151 MHz, CDCl_3_)

HMBC (600, 151 MHz, CDCl_3_)

^1^H NMR (600 MHz, CDCl_3_)

^13^C NMR (151 MHz, CDCl_3_)

^11^B NMR (128 MHz, CDCl_3_)

HSQC (600, 151 MHz, CDCl_3_)

HSQC (600, 151 MHz, CDCl_3_, zoom)

ROESY (600, 600 MHz, CDCl_3_)

ROESY (600, 600 MHz, CDCl_3_, zoom)

HMBC (600, 151 MHz, CDCl_3_)

HMBC (600, 151 MHz, CDCl_3_, zoom)

^1^H NMR (400 MHz, CDCl_3_)

^13^C NMR (100.5 MHz, CDCl_3_)

# 16. Photophysical characterization

*Optical properties* were evaluated in HPLC grade acetonitrile used without further purification. UV-Vis absorption spectra were recorded on a Cary 6000i UV-*vis* spectrophotometer (Varian, Palo Alto, CA, USA) with 1 cm quartz cells. Emission spectra were recorded using a Spex Fluoromax-4 spectrofluorometer (Varian, Palo Alto, CA, USA).

*Fluorescence quantum yields (Φ_F_)* were determined using a comparative method, with quinine sulfate (QS; *Φ*_F_ = 0.546 in 0.5 M H_2_SO_4_ aqueous solution)^[8]^ serving as the external fluorescence standard. The procedure for determining *Φ*_F_ involved measuring the integrated area of the fluorescence band (*I*) for a series of solutions of increasing absorbance and then plotting *I* versus the sample absorption factor (1−10^−A^), which yielded linear plots. Then *Φ*_F_ was obtained by comparison of the slopes of such plots for the quinine sulfate reference and the drug, using Equation 1.

$$\Phi_{F;Sample}=\Phi_{F;QS}\frac{{Slope}_{Sample}}{{Slope}_{QS}} (1)$$

*Fluorescence decays* were recorded with a time-correlated single photon counting system (TimeHarp 260, PicoQuant, Berlin, Germany) equipped with a red sensitive photomultiplier. Excitation was achieved by either a 290-nm picosecond LED working at a 10-MHz repetition rate. The counting frequency was kept always below 1%. Fluorescence decays were analyzed using the EasyTau data analysis software.

*^1^O_2_ photosensitisation* was investigated using time‐resolved near‐infrared phosphorescence,^[9]^ measured with a customised Fluotime 200 time-resolved spectrophotometer (PicoQuant, Berlin, Germany). Excitation at 355 nm was provided by a diode-pumped Nd:YAG laser (FTSS355-Q, Crystal Laser, Berlin, Germany) operating at a 1 kHz repetition rate with a pulse energy of 0.5 μJ. To eliminate residual near-infrared emission, a 1064 nm rugate notch filter (Edmund Optics) and an uncoated SKG-5 filter (CVI Laser Corporation) were placed in the laser beam path. The emitted ^1^O_2_ phosphorescence was selectively detected using a 1100 nm long-pass filter (Edmund Optics) in combination with a narrow-bandpass filter centered at 1275 nm (BK-1270-70-B, bk Interferenzoptik). Detection was carried out using a thermoelectrically cooled, NIR-sensitive photomultiplier tube assembly (H10330C-45-C3, Hamamatsu Photonics, Hamamatsu, Japan). Photon counting was performed with a multichannel scaler (TimeHarp 260, PicoQuant, Berlin, Germany). The amount of ^1^O_2_ generated is proportional to the integrated area of the ^1^O_2_ phosphorescence band.

*Singlet oxygen formation quantum yields (Φ_Δ_)* were determined using a comparative method, with phenalenone (PS; *Φ*_Δ_ = 1 in acetonitrile)^[10]^ serving as the ^1^O_2_ photosensitizer standard. The procedure for determining *Φ*_Δ_ involved measuring the integrated area of the ^1^O_2_ phosphorescence band value for a series of solutions of phenalenone increasing absorbance and then plotting these area values versus the sample absorption factor (1−10^−A^), which yielded a linear plot. Then, *Φ*_Δ_ was obtained by comparison of the integrated area of the ^1^O_2_ phosphorescence band value of a sample with known absorption factor with the slope of the plot for the suitable reference, using Equation 2.

$$\Phi_{\Delta;Sample}=\Phi_{\Delta;PN}\frac{{S(0)}_{Sample}}{{Slope}_{PN}\cdot\left( 1-{10}^{-A;Sample} \right)} (2)$$

**Table S1.** Photophysical data of phenanthridine compounds: *λ*_abs_, absorption band maximum; *ε*, molar absorption coefficient; *λ*_FLUO_, fluorescence band maximum; and *τ*_F_, fluorescence lifetime in air-saturated solutions.

| **Compound** | **λ_ABS_ / nm**  **(ε / M^-1^cm^-1^)** | **λ_FLUO_ / nm** | **τ_F_ / ns** |
| --- | --- | --- | --- |
| **carbo-4** | 254 (56700)  262 (74400) | 378 | 5.0 |
| **4** | 262 (37900)  270 (36900) | 415 | 3.1 |
| **4-inv** | 246 (22600)  270 (22000) | 379 | 4.2 |
| **carbo-6** | 265 (70700) | 384 | 0.3 |
| **6** | 258 (30700)  276 (28300) | 412 | 1.5 |
| ***iodo*-4-inv** | 253 (15000)  277 (17100) | 382 | 2.0 |
| **13** | 240 (27400)  257 (32000)  267 (30600)  278 (26100) | 404 | 0.1 |
| **10** | 272 (39400) | 408 | 0.2 |
| **9** | 221 (40000)  261 (39200)  298 (23600) | 432 | 2.6 |
| **16** | 212 (27100)  259 (30400)  276 (21900) | 452 | 0.3 |
| **18** | 224 (22700)  277 (31000) | 417 | 0.1 |
| **20** | 261 (30800)  281 (31000) | 422 | 0.1 |

**Figure S2**. Absorption spectra of phenanthridine compounds in acetonitrile.

**Figure S3**. Absorption spectra of phenanthridine compounds in acetonitrile.

**Figure S4**. Normalized emission spectra of phenanthridine compounds in acetonitrile.

**Figure S5**. Normalized emission spectra of phenanthridine compounds in acetonitrile.

**Table S2.** Photophysical data of fluorenone compounds: *λ*_abs_, absorption band maximum; *ε*, molar absorption coefficient; *λ*_FLUO_, fluorescence band maximum; *Φ*_F_, fluorescence quantum yield; *τ*_F_, fluorescence lifetime in air-saturated solutions; and *Φ*_Δ_, singlet oxygen quantum yield.

| **Compound** | **λ_ABS_ / nm**  **(ε / M^-1^cm^-1^)** | **λ_FLUO_ / nm** | ***Φ*_F_** | **τ_F_ / ns** | ***Φ*_Δ_** |
| --- | --- | --- | --- | --- | --- |
| **carbo-27** | 268 (40100)  370 (2100) | 549 | 0.004 | 3.5 | 0.05 |
| **27** | 250 (26900)  276 (24700)  367 (3200) | 516 | 0.006 | 1.6 | 0.77 |
| **27-inv** | 252 (22000)  288 (17100) | 406 | <5x10^-4^ | ND | 0.01 |
| **37** | 259 (19100)  289 (15300)  372 (2000) | 508 | - | 1.4 | 0.80 |
| **carbo-28** | 262 (32900)  271 (37900) | 551 | 0.004 | 1.1 | 0.03 |
| **28** | 252 (30000)  284 (28100)  370 (3800) | 526 | 0.005 | 1.4 | 0.22 |
| **39** | 263 (35900)  288 (25400)  374 (3100) | 497 | - | 0.5 | 0.17 |
| **40** | 254 (27500)  278 (24800)  368 (3600) | 513 | - | 0.8 | 0.72 |
| **30** | 256 (23400)  280 (15200)  375 (2700) | 526 | - | 0.6 | 0.12 |
| **29** | 251 (26900)  284 (30100)  376 (4400) | 506 | - | 0.8 | 0.15 |
| **31** | 285 (31700)  376 (2500) | 606 | - | 0.4 | 0.07 |
| **32** | 253 (14700)  280 (15200)  367 (3400) | 439 | - | 0.2 | 0.09 |
| **34** | 246 (24700)  281 (16200)  373 (3300) | 505 | - | 0.5 | 0.20 |
| **33** | 244 (29500)  274 (20700)  371 (4300) | 515 | - | 0.3 | 0.63 |
| **36** | 244 (21900)  279 (13800)  372 (3000) | 503 | - | 0.7 | 0.17 |

**Figure S6**. Absorption spectra of fluorenone compounds in acetonitrile.

**Figure S7**. Absorption spectra of fluorenone compounds in acetonitrile.

**Figure S8**. Normalized emission spectra of fluorenone compounds in acetonitrile.

**Figure S9**. Normalized emission spectra of fluorenone compounds in acetonitrile.

**Figure S10**. Determination of fluorescence quantum yield for different fluorenones derivatives in acetonitrile using a comparative method. Quinine sulphate is used as the fluorescence reference (solvent = H_2_SO_4_ 1N; *Φ*_F_ = 0.54; λ_Exc_ = 355 nm).^[11]^

**Figure S11**. Determination of singlet oxygen formation quantum yield for different fluorenones derivatives in acetonitrile using a comparative method. Phenalenone is used as the ^1^O_2_ reference photosensitizer (solvent = acetonitrile; *Φ*_Δ_ = 1.0; λ_Exc_ = 355 nm).^[12]^

**Table S3.** Estimation of rate constants for singlet-state deactivation (*k*_S_), considering the following deactivation pathways: fluorescence (*k*_F_), intersystem crossing (*k*_ISC_), and internal conversion (*k*_IC_). Notably, we assume that the singlet oxygen formation quantum yield (*Φ*_Δ_) is equivalent to the intersystem crossing quantum yield (*Φ*_ISC_), since the entire triplet-state population is quenched by ground-state molecular oxygen under the present experimental conditions. This assumption is further supported by the short triplet state lifetime (approximately 0.2 μs) observed in the ^1^O_2_ phosphorescence kinetic traces in air-saturated acetonitrile.

| **Compound** | **k_S_ / s^-1^** | **k_F_ / s^-1^** | **k_ISC_ / s^-1^** | **k_IC_ / s^-1^** |
| --- | --- | --- | --- | --- |
| **carbo-27** | 2.86 x 10^8^ | 1.18 x 10^6^ | 2.00 x 10^7^ | 2.65 x 10^8^ |
| **27** | 6.29 x 10^8^ | 3.90 x 10^6^ | 4.72 x 10^8^ | 1.53 x 10^8^ |
| **27-inv** | — | — | — | — |
| **carbo-28** | 8.77 x 10^8^ | 3.51 x 10^6^ | 2.63 x 10^7^ | 8.47 x 10^8^ |
| **28** | 7.14 x 10^8^ | 3.57 x 10^6^ | 1.57 x 10^8^ | 5.54 x 10^8^ |

# 17. DFT calculations

*Geometry optimizations and frequency calculations*.

All ground-state geometry optimizations and frequency calculations were performed using density functional theory (DFT) as implemented in Gaussian 09.^I^ The PBE1PBE (PBE0) hybrid functional^II^ was employed together with the def2-TZVP basis set for all atoms.^III^ An ultrafine integration grid was used throughout (Integral=UltraFine). Empirical dispersion corrections were included using the D3(BJ) scheme of Grimme and co-workers.^IV^ Unless otherwise specified, calculations were carried out using the SMD implicit solvation model for acetonitrile, following the parametrization of Truhlar and co-workers.^V^ Frequency calculations were used to confirm that optimized structures correspond to true minima (no imaginary frequencies). Reported thermochemical data correspond to Gibbs free energies at 298 K within the SMD solvent model.

*Single-point energy calculations*

Single-point electronic energies were computed at the CAM-B3LYP range-separated hybrid functional level^VI^ using the def2-TZVP basis set. The same SMD(acetonitrile) solvation model and D3(BJ) dispersion correction were applied. Wavefunctions from the optimization stage were used as initial guesses (guess=read) to ensure consistent electronic state assignment.

*Excited-State Calculations (TD-DFT)*

Vertical excitation energies for both singlet and triplet excited states were obtained using time-dependent density functional theory (TD-DFT) at the CAM-B3LYP/def2-TZVP level of theory. All calculations employed an ultrafine integration grid, and empirical dispersion was included using the D3(BJ) correction. The SMD implicit solvation model for acetonitrile was used throughout. Excited states were computed as vertical transitions from the optimized ground-state geometry. For each compound, the lowest 20 singlet states TD(NStates=20, Singlets) and the lowest 20 triplet states TD(NStates=20, Triplets) were calculated. Wavefunctions from the ground-state calculations were read in (guess=read) to ensure consistent electronic structure descriptions across the workflow. Molecular orbitals were visualized using GaussView and Avogadro (v. *1*), packages. The reported properties correspond to vertical excitations evaluated at the ground-state geometry.

**Optimized geometries**

**Species Benzofluorenone carbo-27**

Stoichiometry: C_17_H_10_O

Charge: 0 Multiplicity: 1

*Coordinates (xyz)*

C 3.81150213 -1.40028612 -0.00000007

C 4.47434062 -0.15683676 0.00000157

C 3.75310455 1.00593525 0.00000244

C 2.34061775 0.97717121 0.00000173

C 1.67119205 -0.28472210 0.00000014

C 2.44349766 -1.46814405 -0.00000078

C 1.58631113 2.17281519 0.00000267

C 0.21302365 2.16428267 0.00000211

C -0.44540959 0.93035254 0.00000062

C 0.26139140 -0.26198038 -0.00000035

C -1.89159486 0.64104008 -0.00000012

C -2.06033471 -0.74734615 -0.00000162

C -0.71183510 -1.38757191 -0.00000150

C -2.98843588 1.47773406 0.00000057

C -4.25964796 0.89713812 -0.00000025

C -4.42305691 -0.48114839 -0.00000176

C -3.31073620 -1.32458024 -0.00000247

O -0.48043663 -2.57757977 -0.00000293

H 4.39489221 -2.31450279 -0.00000077

H 5.55806819 -0.12663014 0.00000213

H 4.25329257 1.96877413 0.00000371

H 1.93653683 -2.42422892 -0.00000205

H 2.12362973 3.11553803 0.00000387

H -0.34696700 3.09216537 0.00000286

H -2.87629309 2.55605035 0.00000175

H -5.13509351 1.53699013 0.00000032

H -5.42109145 -0.90374217 -0.00000234

H -3.42705980 -2.40289401 -0.00000355

*Imaginary frequencies: none*

E(RCAM-B3LYP)= -728.962289156 Hartrees

Thermal correction to Gibbs Free Energy= 0.178997

Sum of electronic and thermal Free Energies= -728.783292

**Species BN-Benzofluorenone 27**

Stoichiometry: C_15_H_10_BNO

Charge: 0 Multiplicity: 1

*Coordinates (xyz)*

C -4.00926153 1.34617030 -0.00000122

C -4.52571397 0.01854626 -0.00000230

C -3.71126752 -1.06151493 -0.00000184

C -2.66823683 1.57892920 0.00000023

C -1.59151689 -2.07759031 0.00000006

C -0.23013998 -2.06831461 0.00000123

C 0.45997990 -0.84217516 0.00000232

C -0.20540414 0.36915405 0.00000240

C 1.92109315 -0.62497184 0.00000153

C 2.14595226 0.75418056 0.00000137

C 0.81395729 1.44281876 0.00000578

C 2.97924942 -1.51047176 -0.00000031

C 4.27449969 -0.98612219 -0.00000258

C 4.49501938 0.38422059 -0.00000314

C 3.42001903 1.27594027 -0.00000123

O 0.63695756 2.64383273 0.00000047

H -4.72502922 2.16422073 -0.00000155

H -5.59568428 -0.14852579 -0.00000344

H -4.09700200 -2.07429812 -0.00000256

H -2.31734663 2.60631918 0.00000110

H -2.14661762 -3.00858238 -0.00000094

H 0.29836093 -3.01303920 0.00000090

H 2.81938607 -2.58292309 -0.00000051

H 5.12206671 -1.66245278 -0.00000422

H 5.51044467 0.76366274 -0.00000494

H 3.58472017 2.34807606 -0.00000116

B -1.71376655 0.40191078 0.00000053

N -2.34135744 -0.93506687 -0.00000039

*Imaginary frequencies: none*

E(RCAM-B3LYP)= -732.388742740 Hartrees

Thermal correction to Gibbs Free Energy= 0.176683

Sum of electronic and thermal Free Energies= -732.212060

**Species NB-Benzofluorenone 27-inv**

Stoichiometry: C_15_H_10_BNO

Charge: 0 Multiplicity: 1

*Coordinates (xyz)*

C -3.70157052 -1.43351723 0.00000269

C -4.48200810 -0.24949079 -0.00000146

C -3.88341694 0.97650616 -0.00000473

C -2.34353016 -1.40516505 0.00000354

C -1.52618631 2.32390579 -0.00000680

C -0.16419923 2.24220915 -0.00000550

C 0.44104781 0.96690481 -0.00000155

C -0.28230780 -0.20223604 0.00000121

C 1.87926100 0.63825513 -0.00000042

C 2.01848200 -0.75238360 0.00000291

C 0.66023231 -1.36081885 0.00000462

C 2.99293568 1.44914572 -0.00000242

C 4.25241964 0.83844539 -0.00000126

C 4.38577554 -0.54141168 0.00000187

C 3.25288376 -1.35975649 0.00000405

O 0.40068029 -2.54459309 0.00000627

H -4.18537933 -2.40273430 0.00000532

H -5.56382362 -0.35385487 -0.00000194

H -4.51184433 1.86336641 -0.00000792

H -1.75082365 -2.30804172 0.00000667

H -1.98270959 3.31033852 -0.00001017

H 0.47089464 3.12356768 -0.00000767

H 2.90610612 2.52963523 -0.00000505

H 5.14143563 1.45933195 -0.00000295

H 5.37358287 -0.98707272 0.00000259

H 3.34364817 -2.44041986 0.00000650

N -1.65268614 -0.21973896 0.00000044

B -2.37532768 1.06344933 -0.00000382

*Imaginary frequencies: none*

E(RCAM-B3LYP)= -732.377356679 Hartrees

Thermal correction to Gibbs Free Energy= 0.176198

Sum of electronic and thermal Free Energies= -732.201157

**Frontier orbitals shapes and energies**

**Energies of the 10 lowest excited states (TD-DFT)**

*Singlets*

**In all three cases, S1 corresponds to the π-π* HOMO-LUMO excitation.**

*Triplets*

**In all three cases, T_1_ corresponds to the n-π* excitation.**

**DFT References**

I) Frisch, M. J.; et al. *Gaussian 09*; Gaussian, Inc.: Wallingford, CT, **2009**.

II) Adamo, C.; Barone, V. Toward Reliable Density Functional Methods without Adjustable Parameters: The PBE0 Model. *J. Chem. Phys.* **1999**, *110*, 6158–6170.

III) Weigend, F.; Ahlrichs, R. Balanced Basis Sets of Split Valence, Triple Zeta Valence and Quadruple Zeta Valence Quality for H to Rn: Design and Assessment of Accuracy. *Phys. Chem. Chem. Phys.* **2005**, *7*, 3297–3305.

IV) Grimme, S.; Antony, J.; Ehrlich, S.; Krieg, H. A Consistent and Accurate Ab Initio Parametrization of Density Functional Dispersion Correction (DFT-D) for the 94 Elements H–Pu. *J. Chem. Phys.* **2010**, *132*, 154104.

V) Marenich, A. V.; Cramer, C. J.; Truhlar, D. G. Universal Solvation Model Based on Solute Electron Density and a Continuum Model of the Solvent Defined by the Bulk Dielectric Constant and Atomic Surface Tensions. *J. Phys. Chem. B* **2009**, *113*, 6378–6396.

VI) Yanai, T.; Tew, D. P.; Handy, N. C. A New Hybrid Exchange–Correlation Functional Using the Coulomb-Attenuating Method (CAM-B3LYP). *Chem. Phys. Lett.* **2004**, *393*, 51–57.

# 18. Cyclic voltammetry measurements

Cyclic voltammograms were recorded using the SP-50e Potentiostat from BioLogic and controlled through the BioLogic EC-Lab software. The measurements were conducted in a conventional 2.5 mL three-electrode cell containing a glassy-carbon disk working electrode, a Saturated Calomel reference electrode, with a platinum-wire used counter electrode. Experiments were conducted in CH_3_CN using TBAPF_6_ (0.1 M) as electrolyte. Unless specified otherwise, the reported CV’s correspond to a scan rate of 100 mV/sec. For reversible couples, E₁/₂ was taken as the average of the anodic and cathodic peak potentials (E_1/2_ = (E_pa + E_pc)/2).

| **Set up validation.** The described set up was initially validated by obtaining a well-behaved reversible redox process for the ferrocene reference sample (Fc/Fc^+^).  The obtained E_1/2_ (Fc/Fc^+^) of +**0.39** V, consistent with the 0.38-0.42 V *vs* SCE range typically reported for this process. |  |
| --- | --- |

**Voltammograms**

All benzo-fluorenones show two quasi-reversible reduction waves, presumably representing the formation of the anionic forms ***[Benz]^·-^*** and a second doubly reduced forms. No clear oxidation wave could be reached withing the experimental solvent oxidation limit. The first reduction wave appears to be fully chemically reversible, while for the 2^nd^ wave the reversibility was compound-dependent, presumably representing a competing EC-type sequence.

| E_1/2_, mV vs SCE |  | | | | |
| --- | --- | --- | --- | --- | --- |
| Red 1, E_1/2_, mV | -1139 | -1215 | -1029 | -1066 | -1199 |
| Red 2, E_1/2_, mV | -1600 | -1683 | -1655* | -1570 | -1655****** |

*** Partially irreversible at 100 mV/s scan**

As seen in the table, BN-doping causes the displacement of the reduction waves with respect to parent benzofluorenone, with easier/earlier reduction for the B-down isomer and a more difficult/later reduction for the B-up isomer.

# 19. Chemical shift calculations for compound 22-b

DELTA50^[13]^ ^1^H chemical shift predictions were performed at the WP04/6-311++g(2D,P)//B3LYP-D3/6-311G (d, p) level of theory, and ^13^C chemical shift predictions were performed at ωB97X-D/def2SVP//B3LYP-D3/6-311G(d, p) level of theory – both using polarizable continuum model (PCM) for chloroform at the optimization and NMR prediction stages of the calculations.

**Table S4**. DELTA50 based chemical shift (δ) predictions for **22-b**.

| **13C** | **DFT** | **EXP** | **error** | **1H** | **DFT** | **EXP** | **error** |
| --- | --- | --- | --- | --- | --- | --- | --- |
| 1 | 131.6 | 132.77 | 1.3689 | 1 | 7.47 | 7.61 | 0.0196 |
| 2 | 137.5 | 137.11 | 0.1521 | 2 | 7.42 | 7.49 | 0.0049 |
| 3 | 111.7 | 112.4 | 0.49 | 3 | 6.52 | 6.47 | 0.0025 |
| 4 | 136.1 | 134.96 | 1.2996 | 4 | 7.57 | 7.53 | 0.0016 |
| 5 | 128.9 | 129.02 | 0.0144 | 7 | 6.02 | 6.04 | 0.0004 |
| 6 | 160.6 | 158.2 | 5.76 | 8 | 7.47 | 7.36 | 0.0121 |
| 7 | 110 | 109.67 | 0.1089 | 15 | 3.12 | 2.96 | 0.0256 |
| 8 | 134.5 | 133.26 | 1.5376 | 16 | 3.18 | 3.09 | 0.0081 |
| 12 | 175.6 | 172.7 | 8.41 | 17 | 6.8 | 6.87 | 0.0049 |
| 13 | 140.5 | 139.8 | 0.49 | 18 | 6.87 | 6.94 | 0.0049 |
| 14 | 143.5 | 141.74 | 3.0976 | 19 | 6.97 | 7.00 | 0.0009 |
| 15 | 56.2 | 55.42 | 0.6084 | 20 | 7.06 | 7.07 | 0.0001 |
| 16 | 56.9 | 55.83 | 1.1449 | 21 | 2.62 | 2.76 | 0.0196 |
| 17 | 123.4 | 122.96 | 0.1936 | 22 | 1.55 | 1.46 | 0.0081 |
| 18 | 124.1 | 125.07 | 0.9409 |  | 1.85 | 1.80 | 0.0025 |
| 19 | 127.7 | 128.42 | 0.5184 | 23 | 1.5 | 1.46 | 0.0016 |
| 20 | 124.6 | 124.46 | 0.0196 |  | 1.81 | 1.81 | 0 |
| 21 | 42.1 | 40.01 | 4.3681 | 24 | 3.15 | 3.36 | 0.0441 |
| 22 | 32.6 | 30.53 | 4.2849 | 25 | 2.43 | 2.24 | 0.0361 |
| 23 | 32.1 | 31.05 | 1.1025 |  | 1.71 | 1.65 | 0.0036 |
| 24 | 44.2 | 42.33 | 3.4969 | 26 | 2.56 | 2.5 | 0.0036 |
| 25 | 40.2 | 37.97 | 4.9729 |  |  |  |  |
| 26 | 28.7 | 27.43 | 1.6129 |  |  |  |  |
|  |  |  |  |  |  |  |  |
|  |  | Sum(errors)/N | 1.9997 |  |  | Sum(errors)/N | 0.0098 |
|  |  | rmsd | 1.4141 |  |  | rmsd | 0.0988 |
|  |  |  |  |  |  |  |  |

**Table S5**. DFT coordinates for **22-b**.

| Boltzmann population 100% | | | | | |
| --- | --- | --- | --- | --- | --- |
| Atom type | Carbon # | ID | x | y | z |
| C | 1 | 1 | -2.77517 | 1.358012 | 0.423133 |
| C | 2 | 2 | -4.05439 | 1.224083 | 0.886839 |
| C | 3 | 3 | -4.78063 | 0.008728 | 0.716675 |
| C | 4 | 4 | -4.21299 | -1.05333 | 0.091555 |
| C | 5 | 5 | -0.65439 | 0.1049 | -0.8765 |
| C | 6 | 6 | -0.28745 | -1.11173 | -1.42506 |
| C | 7 | 7 | -1.18909 | -2.21834 | -1.51445 |
| C | 8 | 8 | -2.44447 | -2.15766 | -1.01621 |
| N | 9 | 9 | -2.92216 | -1.01577 | -0.40609 |
| B | 10 | 10 | -2.07384 | 0.201246 | -0.29256 |
| N | 11 | 11 | 0.98619 | -1.35597 | -2.0004 |
| C | 12 | 12 | 1.986595 | -1.57421 | -1.23727 |
| C | 13 | 13 | 1.875659 | -1.51257 | 0.256206 |
| C | 14 | 14 | 1.659545 | -0.26507 | 0.875272 |
| C | 15 | 15 | 1.69133 | 0.984065 | 0.014471 |
| C | 16 | 16 | 0.413661 | 1.193306 | -0.94216 |
| C | 17 | 17 | 1.924629 | -2.68632 | 1.008358 |
| C | 18 | 18 | 1.728819 | -2.64736 | 2.387327 |
| C | 19 | 19 | 1.493661 | -1.42442 | 3.008404 |
| C | 20 | 20 | 1.465402 | -0.24863 | 2.258458 |
| C | 21 | 21 | 1.77742 | 2.335569 | 0.765879 |
| C | 22 | 22 | 2.28272 | 3.40759 | -0.22537 |
| C | 23 | 23 | 1.058198 | 3.610463 | -1.17173 |
| C | 24 | 24 | -0.00129 | 2.642491 | -0.59456 |
| C | 25 | 25 | 0.294471 | 2.725491 | 0.918437 |
| C | 26 | 26 | 3.330246 | -1.8916 | -1.82797 |
| H | 1 | 27 | -2.29936 | 2.312618 | 0.597089 |
| H | 2 | 28 | -4.55107 | 2.044829 | 1.398221 |
| H | 3 | 29 | -5.79454 | -0.08545 | 1.084933 |
| H | 4 | 30 | -4.74646 | -1.98661 | -0.04556 |
| H | 7 | 31 | -0.8498 | -3.13026 | -1.98824 |
| H | 8 | 32 | -3.12357 | -2.99975 | -1.07287 |
| H | 15 | 33 | 2.569702 | 0.887607 | -0.63098 |
| H | 16 | 34 | 0.791173 | 1.175818 | -1.96959 |
| H | 17 | 35 | 2.096914 | -3.63467 | 0.510594 |
| H | 18 | 36 | 1.757025 | -3.56275 | 2.967103 |
| H | 19 | 37 | 1.336405 | -1.38009 | 4.080324 |
| H | 20 | 38 | 1.290632 | 0.690913 | 2.766752 |
| H | 21 | 39 | 2.366371 | 2.289015 | 1.682237 |
| H | 22 | 40 | 3.18346 | 3.086368 | -0.75467 |
| H | 22 | 41 | 2.527714 | 4.330411 | 0.306888 |
| H | 23 | 42 | 1.287655 | 3.387687 | -2.2167 |
| H | 23 | 43 | 0.694739 | 4.640462 | -1.12929 |
| H | 24 | 44 | -1.01109 | 2.887298 | -0.91604 |
| H | 25 | 45 | -0.27841 | 2.010307 | 1.509659 |
| H | 25 | 46 | 0.152239 | 3.727256 | 1.33425 |
| H | 26 | 47 | 3.273821 | -1.91997 | -2.91602 |
| H | 26 | 48 | 3.689379 | -2.85523 | -1.45283 |
| H | 26 | 49 | 4.063179 | -1.14076 | -1.51546 |

# 20. X-Ray diffraction structural determination details

In all cases, a prismatic crystal was selected and used for the X-ray crystallographic analysis. The X-ray intensity data were measured on a D8 Venture system equipped with a multilayer monochromator and a Mo microfocus (λ = 0.71073 Å). The frames were integrated with the Bruker SAINT software package^[11]^ using a narrow-frame algorithm. Data were corrected for absorption effects using the multi-scan method (SADABS),^[12]^ and the structure was solved and refined using the Bruker SHELXTL software package.^[13]^

**Table S6**. Crystal data and structure refinement for 3ANABB145.

_____________________________________________________________________

Identification code: 3ANABB145

Chemical formula: C_15_H_11_BN_2_

Formula weight: 230.07 g/mol

Temperature: 100(2) K

Wavelength: 0.71073 Å

Crystal system: monoclinic

Space group: P 21/c

Unit cell dimensions: a = 9.5092(14) Å α = 90°

b = 8.8712(13) Å β = 95.571(6)°

c = 13.372(2) Å γ = 90°

Volume: 1122.7(3) Å^3^

Z: 4

Density (calculated): 1.361 g/cm^3^

Absorption coefficient: 0.080 mm^-1^

F(000): 480.0

Crystal size: 0.200 mm x 0.121 mm x 0.114 mm

Theta range for data collection: 2.15 to 26.43°

Index ranges: -11<=h<=11, -11<=k<=11, -16<=l<=15

Reflections collected: 18072

Independent reflections: 2306 [R(int) = 0.0775]

Refinement method: Full-matrix least-squares on F^2^

Refinement program SHELXL-2019/1 (Sheldrick, 2019)

Function minimized Σ w(Fo^2^ - Fc^2^)^2^

Data / restraints / parameters: 2306 / 0 / 163

Goodness-of-fit on F^2^: 1.067

Final R indices [I>2σ(I)]: 1661 data; I>2σ(I) R1 = 0.0691, wR2 = 0.1452

all data R1 = 0.0983, wR2 = 0.1597

Weighting scheme: w=1/[σ^2^(F_o_^2^)+(0.0368P)^2^+1.6481P]

where P=(F_o_^2^+2F_c_^2^)/3

Largest diff. peak and hole: 0.267 and -0.302 eÅ^-3^

R.M.S. deviation from mean 0.055 eÅ^-3^

_____________________________________________________________________

**Figure S12**. Detail of the crystal packing pattern in 3ANABB145.

**Table S7**. Crystal data and structure refinement for 3ANABB66.

_____________________________________________________________________

Identification code: 3ANABB66

Chemical formula: C_17_H_12_BNO_3_

Formula weight: 289.09 g/mol

Temperature: 100(2) K

Wavelength: 0.71073 Å

Crystal system: orthorhombic

Space group: P 21 21 21

Unit cell dimensions: a = 5.4123(2) Å α = 90°

b = 15.2251(6) Å β = 90°

c = 15.9429(6) Å γ = 90°

Volume: 1313.74(9) Å^3^

Z: 4

Density (calculated): 1.462 g/cm^3^

Absorption coefficient: 0.099 mm^-1^

F(000): 600

Crystal size: 0.180 mm x 0.057 mm x 0.035 mm

Theta range for data collection: 1.85 to 26.36°

Index ranges: -6<=h<=5, -19<=k<=19, -19<=l<=18

Reflections collected: 8843

Independent reflections: 2664 [R(int) = 0.0433]

Refinement method: Full-matrix least-squares on F^2^

Refinement program SHELXL-2019/1 (Sheldrick, 2019)

Function minimized Σ w(Fo^2^ - Fc^2^)^2^

Data / restraints / parameters: 2664 / 0 / 200

Goodness-of-fit on F^2^: 1.063

Final R indices: 2414 data; I>2σ(I) R1 = 0.0385, wR2 = 0.0875 all data R1 = 0.0439, wR2 = 0.0901

Weighting scheme: w=1/[σ^2^(Fo^2^)+(0.0384P)^2^+0.2873P]

where P=(F_o_^2^+2F_c_^2^)/3

Absolute structure parameter -1.0(7)

Largest diff. peak and hole: 0.186 and -0.220 eÅ^-3^

R.M.S. deviation from mean 0.046 eÅ^-3^

**Figure S13**. Detail of the crystal packing pattern in 3ANABB66.

# 21. References

[1] For a revisited synthesis of [1,2]azaborinino[1,2-*a*][1,2]azaborinine **1** see: **Rulli, F.; Sanz-Liarte, G.; Roca, P.; Martínez, N.; Medina, V.; Puig de la Bellacasa, R.; Shafir, A.; Cuenca, A. B. From propenolysis to enyne metathesis: tools for expedited assembly of 4a,8a-azaboranaphthalene and extended polycycles with embedded BN.** *Chem. Science*, **2024**, *15*, 5674-5680.

[2] (a) Sun, F. ; Lv, L.; Huang, M.; Zhou, Z.; Fang, X. Palladium-catalyzed cross-coupling reactions of 4a,8a‑azaboranaphthalene. *Org. Lett.*, **2014**, *16*, 5024−5027. (b) Zhang, P.-F.; Zeng, J.-C.; Zhuang, F.-D.; Zhao, K.-X.; Sun, Z.-F.; Lu, Y.; Wang, X.-Y.; Wang, J.-Y.; Pei, J. Parent B_2_N_2_-perylenes with different BN orientations. *Angew. Chem.* *Int. Ed.*, **2021**, *60*, 23313-23319.

[3] Candito, D. A.; Lautens, M. Palladium-catalyzed domino direct arylation/N-arylation: convenient synthesis of phenanthridines. *Angew. Chem. Int. Ed.*, **2009**, *48*, 6713−6716.

[4] (a) Zengqi, X.; Seong-Jun, Y.; Soo Young, P. Synthesis of highly fluorescent and soluble 1,2,4-linking hyperbranched poly(arylenevinylene) featuring intramolecular energy funneling. *Adv. Functional Materials*, **2010**, *20*, 1638−1644. (b) Chugunov, D. B.; Okina, E. V.; Timonina, A. S.; Klimaeva, L. A.; Selivanova, Y. M. Synthesis and electrochemical properties of 2,5-disubstituted 1,4-bis(4,5-diphenyl-1H-imidazol-2-yl)benzene derivatives. *Russian Journal of Org. Chemistry*, **2020**, *56*, 1222−1227.

[5] Su, X.; Li, C.; Du, Q.; Tao, K.; Wang, S.; Yu, P. Atomically precise synthesis and characterization of heptauthrene with triplet ground state. *Nano Lett.*, **2020**, *20*, 9, 6859−6864.

[6] Joost, M.; Zeineddine, A.; Estévez, L.; Mallet-Ladeira, S.; Miqueu, K.; Amgoune, A.; Bourissou, D*.* Facile oxidative addition of aryl iodides to gold (I) by ligand design: bending turns on reactivity*. J. Am. Chem. Soc.*, **2014**, *136*, 42, 14654–14657

[7] Zhao, Y.-B.; Mariampillai, B.; Candito, D. A.; Laleu, B.; Li, M.; Lautens, M. Exploiting the divergent reactivity of aryl–Palladium intermediates for the rapid assembly of fluorene and phenanthrene derivatives. *Angew. Chem. Int. Ed.,* **2009**, *48*, 1849-1852.

[8] Fletcher, A. A. Quinine sulfate as a fluorescence quantum yield standard. *Photochem. Photobiol.*, **1969**, *9*, 439-444.

[9] Martí, C.; Jürgens, O.; Cuenca, O.; Casals, M.; Nonell, S. Aromatic ketones as standards for singlet molecular oxygen O2 (1Δg) photosensitization. Time-resolved photoacoustic and near-IR emission studies. *J. Photochem. Photobiol. A Chem.*, **1996**, *97*, 11–18.

[10] Cohen, R. D.; Wood, J. S.; Lam, Y.-H.; Buevich, A. V.; Sherer, E. C.; Reibarkh, M.; Williamson, R. T.; Martin, G. E. DELTA50: a highly accurate database of experimental ^1^H and ^13^C NMR chemical shifts applied to DFT benchmarking. *Molecules*, **2023**, *28*, 2449.

[11] Data reduction with Bruker SAINT versions V7.60A. Bruker 2007. Bruker AXS Inc., Madison, Wisconsin, USA.

[12] G. M. Sheldrick, (1996). SADABS. Program for Empirical Absorption Correction. University of Gottingen, Germany.

[13] G. M. Sheldrick, Acta Crystallographica Section C-Structural Chemistry 2015, 71, 3-8.
